# Bioactivity-Guided Identification of Botanical Inhibitors of Ketohexokinase

**DOI:** 10.1371/journal.pone.0157458

**Published:** 2016-06-20

**Authors:** MyPhuong T. Le, Miguel A. Lanaspa, Christina M. Cicerchi, Jatinder Rana, Jeffrey D. Scholten, Brandi L. Hunter, Christopher J. Rivard, R. Keith Randolph, Richard J. Johnson

**Affiliations:** 1 Division of Renal Diseases and Hypertension, Department of Medicine, University of Colorado Anschutz Medical Campus, Aurora, Colorado, United States of America; 2 Amway Research and Development, Ada, Michigan, United States of America; Louisiana State University Health Sciences Center, UNITED STATES

## Abstract

**Objective:**

In developed countries with westernized diets, the excessive consumption of added sugar in beverages and highly refined and processed foods is associated with increased risk for obesity, diabetes, and cardiovascular diseases. As a major constituent of added sugars, fructose has been shown to cause a variety of adverse metabolic effects, such as impaired insulin sensitivity, hypertriglyceridemia, and oxidative stress. Recent studies have shown that ketohexokinase isoform C is the key enzyme responsible in fructose metabolism that drive’s fructose's adverse effects. The objective of this study was to identify botanical ingredients with potential for inhibitory activity against ketohexokinase-C and fructose-induced metabolic effects by using a series of *in vitro* model systems.

**Methods:**

Extracts from 406 botanicals and 1200 purified phytochemicals were screened (initial concentration of 50 μg/mL and 50 μM, respectively) for their inhibitory activity using a cell free, recombinant human ketohexokinase-C assay. Dose response evaluations were conducted on botanical extracts and phytochemicals that inhibited ketohexokinase-C by > 30% and > 40%, respectively. Two different extract lots of the top botanical candidates were further evaluated in lysates of HepG2 cells overexpressing ketohexokinase-C for inhibition of fructose-induced ATP depletion. In addition, extracts were evaluated in intact Hep G2 cells for inhibition of fructose-induced elevation of triglyceride and uric acid production.

**Results:**

Among the botanical extracts, phloretin (*Malus domestica)* extracts were the most potent (IC_50_: 8.9–9.2 μg/mL) followed by extracts of *Angelica archangelica* (IC_50:_ 22.6 μg/mL—57.3 μg/mL). Among the purified phytochemicals, methoxy-isobavachalcone (*Psoralea corylifolia*, IC_50_ = 0.2 μM) exhibited the highest potency against ketohexokinase isoform C activity followed by osthole (*Angelica archangelica*, IC_50_ = 0.7 μM), cratoxyarborenone E (*Cratoxylum prunifolium*, IC_50_ = 1.0 μM), and α-/γ-mangostin (*Cratoxylum prunifolium*, IC_50_ = 1.5 μM). Extracts of *Angelica archangelica*, *Garcinia mangostana*, *Petroselinum crispum*, and *Scutellaria baicalensis* exhibited ketohexokinase inhibitory activity and blocked fructose-induced ATP depletion and fructose-induced elevation in triglyerides and uric acid.

**Conclusions:**

*Angelica archangelica*, *Garcinia mangostana*, *Petroselinum crispum*, and *Scutellaria baicalensis* were the top four botanical candidiates identified with inhibitory activity against ketohexokinase-C. Future studies are needed to show proof of mechanism and the efficacy of these botanical extracts in humans to blunt the negative metabolic effects of fructose-containing added sugars.

## Introduction

The excessive consumption of added sugars in westernized diets is epidemiologically associated with rising prevalence of obesity, metabolic syndrome, and cardiovascular diseases in the United States [1–4]. It is estimated that over 70% of adults consume ≥10% of their total calories from added sugars and approximately 10% of adults consume ≥ 25% [1]. Although the consumption of added sugars has decreased during the past few years, the average intake remains high at about 75 g/day [5]. As a major component of added sugars, high intake of fructose has been shown to cause numerous adverse metabolic effects, suggesting that it has a contributory role in the development of obesity and metabolic syndrome [6–8]. Administration of added sugars or fructose has been shown to induce all of the features of metabolic syndrome in rats and in humans, such as hypertriglyceridemia and lipogenesis, increased blood pressure, fatty liver, and visceral fat accumulation [9–11]. Fructose has also been shown to impair insulin sensitivity, injure β-islet cells, and cause lactic acidosis, oxidative stress, and kidney injuries in animals [12–19].

**Fig 1:**
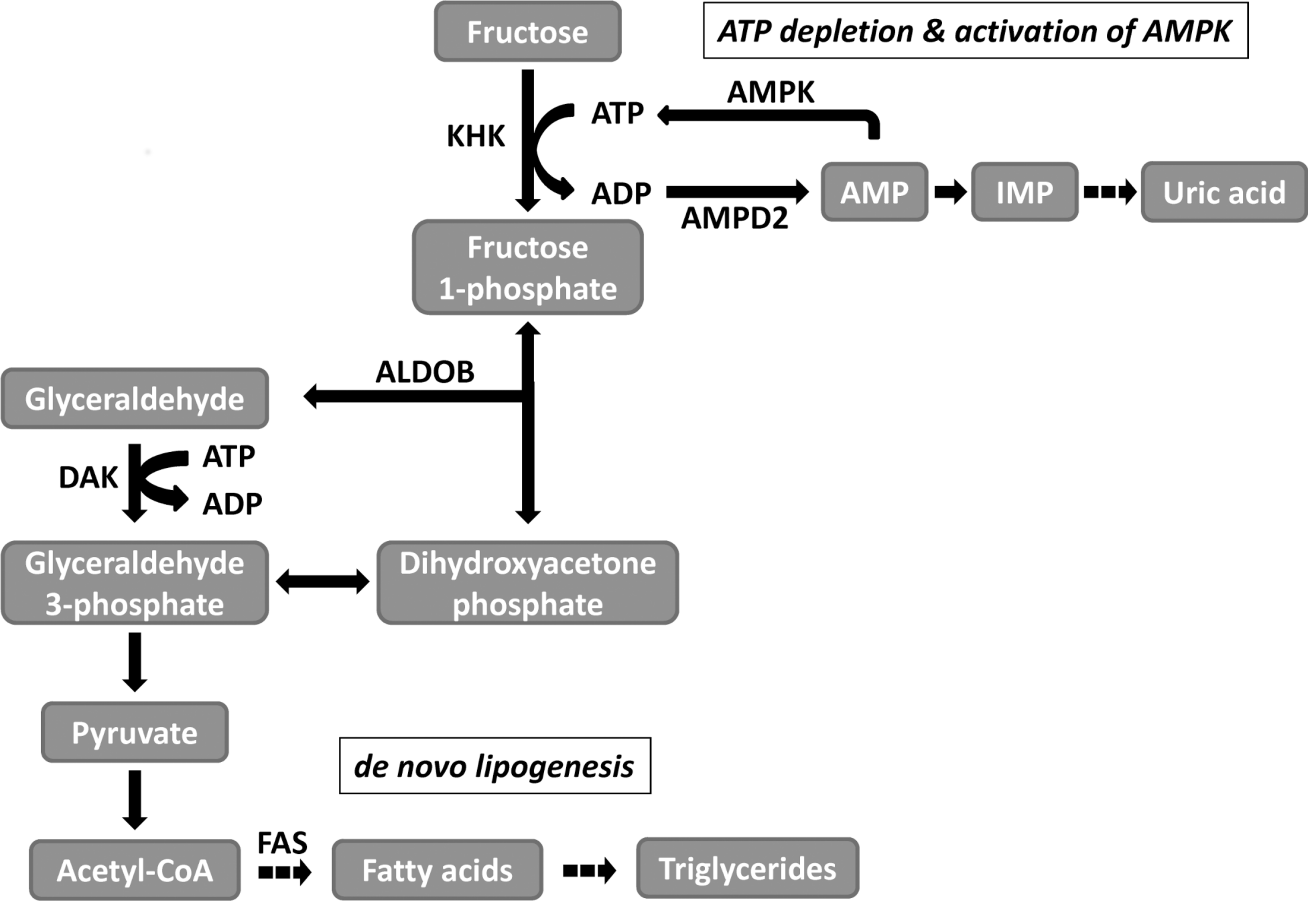
Fructose metabolism and downstream effects. ALDOB aldolase B, AMPD2 adenosine monophosphate deaminase 2, AMPK AMP-activated protein kinase, DAK dihydroxyacetone kinase, FAS fatty acid synthase, KHK ketohexokinase.

The liver, which metabolizes 50 to 70% of ingested fructose, has the highest expression of ketohexokinase (KHK, fructokinase) among body tissues and organs. KHK is an enzyme that specifically initiates the metabolism of fructose and phosphorylates it to generate fructose 1-phosphate (Fig 1) [20]. The metabolism of fructose by KHK is rapid, resulting in a reduction in hepatic ATP and promoting the accumulation of uric acid [21, 22]. Recent studies have highlighted the importance of KHK as the key mechanism stimulating the various adverse effects of fructose. For instance, fructose-induced production of reactive oxidative species was dramatically reduced when the expression of KHK was knocked down in proximal tubular cells [13]. Furthermore, studies utilizing KHK-knockout mice on a high fructose diet have shown that they are protected from developing metabolic syndrome and fatty liver compared to wild type control animals [11, 23].

There are two isoforms of KHK: KHKA and KHKC. Due to alternative splicing, the expressions of the isoforms are tissue dependent. While KHKA is expressed at low levels in most tissues, high levels of KHKC are primarily expressed in the liver, kidneys, and small intestine [24, 25]. Notably, only recombinant human KHKC, but not KHKA, was capable of rapidly metabolizing fructose and causing acute depletion of hepatic ATP [11]. In a study evaluating the effects of a high fructose diet, KHKA and KHKC double-knockout mice were protected against weight gain and fatty liver compared to wild type mice. However, KHKA knockout mice, which express only KHKC, developed similar or worsening metabolic effects to the fructose diet compared to wild type mice despite equal fructose intake. Thus, the data suggests that KHKC is the isoform responsible for driving fructose-induced adverse metabolic effects [11].

The inhibition of KHKC provides a unique approach for ameliorating the effects of fructose and added sugars in the diet. A botanical inhibitor of KHKC may help mitigate the metabolic consequences from the consumption of added sugars on human health. Botanicals are widely used for therapeutic purposes and it is estimated that two-thirds of the world’s population utilize botanicals and their extracts in traditional medicine [26]. Importantly, the potential value of botanicals to ameliorate the adverse metabolic effects of dietary fructose has been shown in several animal studies. For instance, fructose-fed diabetic rats treated with diabegon, a polyherbal preparation, had lower levels of plasma insulin, liver glycogen, and total cholesterol [27]. Furthermore, fructose-induced insulin resistant rats treated with extracts of *Angelica keiskei*, *Panax ginseng* root, and the root of *Acanthopanax senticosus* were found to have improved insulin sensitivity and hypertriglyceridemia [28–30]. Although these studies showed that botanicals can ameliorate the adverse effects of dietary fructose, they did not identify a mechanism of action for these botanicals.

Because KHKC is essential to the rapid metabolism of fructose and its adverse effects, this specific isoform was the main target for inhibition. Thus, the objective of this study was to develop assays that could be utilized to identify botanicals with bioactivity against KHKC and fructose-induced elevation in triglycerides and uric acid. Potentially, top botanical candidates with strong inhibitory activity against KHKC could be developed into therapeutics to prevent or treat the adverse effects of high dietary sugar intake, such as metabolic imbalance, weight gain, and cardiovascular disease.

## Materials and Methods

### Botanicals and Phytochemicals

#### Botanical extract library

Extracts from dry milled powders of 406 botanicals were obtained from Amway’s internal botanical library. Hydrophilic, lipophilic, and combination extracts were prepared for each botanical. For the hydrophilic extractions, 50 g of a powdered botanical was mixed with 300 mL of methanol (Fisher Scientific, Inc., Pittsburgh, PA). For the lipophilic extractions, 50 g of a powdered botanical was mixed with 300 mL of chloroform (Fisher Scientific, Inc.). After a 12 hr incubation, the mixtures were sonicated for 1 hr at room temperature and filtered through GF/A grade filter paper. For the combination extracts, 100 mL aliquots of the hydrophilic and lipophilic solutions of a particular botanical were mixed together. To evaporate the solvents, the extractions were placed in a rotary evaporator, then a nitrogen evaporator, and finally a vacuum desiccator. Final dry weights of the botanical extracts were determined and fractions were stored at -80°C.

#### Phytochemical library

From AnalytiCon Discovery GmbH (Potsdam, Germany), 1200 phytochemicals from the MEGx library were purchased by Amway. Each phytochemical consisted of 100 μg and were extracted from plants with an approximate 90% purity. The phytochemicals were stored at -80°C.

#### Top botanical candidates

Dry powdered preparations of the four top botanical candidates were purchased: *Angelica archangelica* was obtained from NATUREX (Avignon, France), *Scutellaria baicalensis* from PLT Health Solutions (Morristown, New Jersey), *Petroselinum crispum* from ABG Farms (Ada, MI), and *Garcinia mangostana* from BI Nutraceuticals (Long Beach, CA). The plant materials were extracted with a combination of ethanol and water (50–70% ethanol:50–30% water). Two separate extractions from different lots of plant material of each botanical were performed (Lot #1 and Lot #2). Dried extracts were stored at -80°C.

#### HPLC fingerprinting of botanical extracts

Botanical extracts of *Angelica archangelica*, *Scutellaria baicalensis*, *Petroselinum crispum*, and *Garcinia mangostana* were analyzed by HPLC fingerprinting. Approximately 300 mg of extracts were mixed with 40 mL of 80% methanol in deionized water and sonicated in a water bath for ten minutes. Additional solvent was added to a final volume of 100 mL. The solution was filtered with a 0.45 μm syringe into an auto sampler vial. 10 μL of the sample was injected into the HPLC. HPLC separation was achieved using a C18 Nova-Pak column (4μm, 250 X 4.6 mm, Waters Corporation, Milford, MA) on an HP1100 system (Agilent Technologies Inc., Santa Clara, CA) equipped with photodiode-array detection and Chemstation software. Botanical extracts were fingerprinted with 0.2% ortho-phosphoric acid (OPA) v/v with deionized water and acetonitrile (ACN) elution gradient as outlined in Table 1. Flow rate was 1.0 mL/min and the detection wavelengths were 210–370 nm. All solvents were HPLC grade and purchased from Fisher Scientific, Inc.

**Table 1:** HPLC gradient conditions.

Time (min)	0.2% o-Phosphoric acid (%)	Acetonitrile (%)
0	92	8
12	90	10
14	88	12
26	76	24
34	60	40
37	60	40
40	92	8
42	92	8

The following marker compounds were utilized for the HPLC fingerprinting of the botanicals: osthole and osthenol were used for *Angelica archangelica*, baicalin for *Scutellaria baicalensis*, apiin for *Petroselinum crispum*, and α-mangostin for *Garcinia mangostana* [31–34]. The analytical standards were purchased from ChromaDex Corporation (Irvine, CA).

### Assays

#### Inhibition of KHKC activity

**Fig 2:**
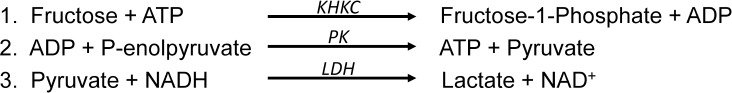
3-Step recombinant ketohexokinase enzymatic assay. KHKC Ketohexokinase C isoform. PK pyruvate kinase. LDH lactate dehydrogenase.

Botanical extracts and phytochemicals were screened for their inhibitory activity using a high throughput cell-free enzymatic assay that utilized recombinant KHKC. KHKC activity was assayed with a 3-step reaction (Fig 2) [25, 35, 36]. The Synergy 2 multi-mode microplate reader was used to measure the decrease in NADH at A_340nm_ (BioTek Instruments, Inc., Winooski, VT). For screening of the botanical extracts and phytochemicals, the KHKC enzymatic assay was carried out at 37°C in a total reaction volume of 200 ul containing 50 mM PIPES, 6 mM MgCl_2_, 100 mM KCl, 5 mM ATP, 2 mM phosphoenolpyruvate, 0.3 mM NADH, 15 U of pyruvate kinase, 15 U of lactate dehydrogenase, and 75 ng KHKC. The botanical extracts (50 μg/mL) and phytochemicals (50 μM) were preincubated in the reaction mixture for 15 min at 37°C. Afterwards, 1 mM fructose was added to the reactions, except for the no fructose controls, and A_340nm_ data were collected every minute for 1 hr. The changes in absorbance were calculated for all samples based on the linear phase of the reactions and adjusted for the no fructose controls. Compared to the fructose only controls (1 mM fructose, no inhibitors), percentage inhibition of KHKC for each sample was calculated. Botanical extracts exhibiting KHKC inhibition by at least 30% were followed up with dose response evaluations (0.1 μg/mL to 200 μg/mL) in the same recombinant KHKC enzymatic assay. For phytochemicals that inhibited KHKC by at least 40%, dose responses were conducted from 0.05 to 100 μM. IC_50_s against KHKC were calculated. 4-(hydroxymercuri) benzoic acid sodium salt was used as a positive inhibitory control of KHKC [36]. All chemicals were purchased from Sigma-Aldrich (St. Louis, MO).

Recombinant human KHKC proteins: Recombinant human KHKC was produced using the Profinity eXact fusion-tag system (Bio-Rad Laboratories, Hercules, CA). RNA were isolated from Hep G2 cells. CDNA was generated using 1 μg of RNA (iScript cDNA synthesis kit, Bio-Rad Laboratories). The coding sequences of KHKC were amplified using iProof high-fidelity DNA polymerase (Bio-Rad Laboratories). The following primers were used: forward primer = 5’…gaagataagctcttcaaagctttggaagagaagcagatcctgtgcgtgg…3’ and reverse primer = 5’…GCTGGATATCTGCAGAATTCTCACACGATGCCATCAAAGCCC…3’. The amplicons were ligated into pPAL8, a bacterial expression vector. Sequencing, performed by Beckman Coulter Genomics (Danvers, MA), confirmed that the coding sequences of the KHKC clones were a match to the National Center for Biotechnology Information’s reference sequence. The clones were transformed into BL21 (DE3) chemi-competent expression cells (Bio-Rad Laboratories). *E*. *coli* cultures were grown at 37°C in terrific broth media containing 100 μg/mL ampicillin. When the cell density (OD_600_) reached 0.5–0.7, the expression of KHKC was induced by adding 1 mM isopropyl β-D-1-thiogalactopyranoside. After 3–4 hr incubation, the growth culture was centrifuged at 5,000 g for 10 min at 4°C and the pellet was stored at -80°C.

For protein purification, the bacterial cell pellet was lysed using B-PER in phosphate buffer (Thermo Fisher Scientific Inc., Rockford, IL) containing AEBSF protease inhibitor (EMD Chemicals, Gibbstown, NJ). The lysate was centrifuged at 30,000 x g for 30 min at 4°C to remove cell debris. The supernatant was filtered using a 0.45 uM syringe filter and then loaded (2.5 mL/min) onto the Bio-Scale Mini Profinity eXact cartridges using the Profinia protein purification instrument (Bio-Rad Laboratories). The column was first washed with 100 mL sodium phosphate, pH 7.0 (10 ml/min, CV = 10) followed by 400 mL sodium phosphate, pH 7.0 (10 ml/min, CV = 15). The protein was eluted with 100 mM sodium phosphate and 100 mM sodium fluoride, pH 7.2 and incubated for 4 hr at 4°C. The tag-less protein was stored in 20% glycerol and frozen at -80°C until use.

#### Inhibition of fructose-induced ATP depletion

Fructose-induced ATP depletion was measured in cell lysates of Hep G2 cells overexpressing KHKC. As previously described, lentiviral particles were utilized to generate KHKC-overexpressing Hep G2 cells [37]. Cells were grown in RPMI 1640 media supplemented with 10% FBS and 1% penicillin and streptomycin and were maintained in a 5% CO_2_ humidified incubator at 37°C. Hep G2 cells were lysed using cold buffer containing 150 mM KCl, 20 mM Tris (pH 7.5), 1 mM EDTA (pH 8), and 1 mM DTT. The protein content of the lysate was determined using Pierce BCA protein assay (Thermo Fisher Scientific Inc., Rockford, IL). The ATP depletion cell lysate reaction contained 50 μg of lysate protein, 50 mM imidazol, 4 mM magnesium chloride, 1 M potassium acetate (pH 7.5), 20 mM n-acetylglucosamine, 40 mM sodium fluoride, 5 mM fructose, and 5 mM ATP in a total reaction volume of 200 μL. Botanical extracts of the top botanical candidates were preincubated with the reaction mixture for 15 min at 37°C. After adding in the fructose (except for no fructose controls), the reaction was continuously shaken and incubated at 37°C for 120 min. Samples of each reaction were taken after 2 min and 120 min for measuring ATP levels. ATP levels were measured using an ATP assay kit (BioVision, Inc., Milpitas, CA). 5 uL of sample was added to 95 uL of the ATP reaction mixture and readings were taken at 570 nm. For each sample, the change in ATP levels was calculated and the percentage ATP inhibition was determined. IC_50_s were calculated for inhibiting ATP depletion from a dose response curve (0.1 to 500 μg/mL) of the extracts from the top four botanical candidates.

#### Inhibition of fructose-induced triglyceride and uric acid production

Whole cell experiments were conducted in regular Hep G2 cells. Cells at 80% confluency were treated with different doses of extracts (0.1 ug/mL to 500 ug/mL) of the top botanical candidates in the presence or absence of 5 mM fructose for 72 hr. Control treatments included cells that were treated with only fructose or with DMSO only (no fructose controls). The media was changed twice daily. Cell lysates were obtained with MAP kinase lysis buffer (50 mM β-glycerophosphate, 2 mM MgCl_2_, 100 mM sodium vanadate, 1 mM EGTA, 1 mM DTT, and 0.5% (v/v) triton X-100) [38]. Triglyceride (TG) and uric acid (UA) levels were measured by VetAce autoanalyzer (Alfa Wassermann, West Caldwell, NJ). The levels were normalized to the amount of soluble protein in the lysates. The percentage inhibition in TG and UA were then compared to the relative change between the fructose only and no fructose controls and IC_50_s were determined.

#### MTT assay

Percentage cell viability was determined using MTT Cell Proliferation Assay according to the manufacturer’s protocol (ATCC, Manassas, VA). Hep G2 cells were grown in 96-well plates at a density of ~10,000 cells/well. The cells were exposed for 72 hr to 5 mM fructose with or without the botanical extracts from 0 to 500 μg/mL. Extracts from Lot #1 of *Angelica archangelica*, *Scutellaria baicalensis*, *Petroselinum crispum*, and *Garcinia mangostana* were used. Experiments were done in triplicates and the absorbance was read at 570 nm.

### Data Analysis

GraphPad Prism 5.03 (GraphPad Software, La Jolla, CA) was utilized for the data and statistical analyses. To generate a best fit, an upper concentration (10,000 μg/mL at 100% inhibition) and a lower concentration (0.001 μg/mL at 0% inhibition) were added in order to calculate the IC_50_s for the inhibition of KHKC and for the inhibition of fructose-induced ATP depletion. No placeholders were used for the calculation of the IC_50_s for the inhibition of fructose-induced elevation in triglycerides and uric acid in the whole cell studies. Nonlinear regression, three parameters, was used as the analysis method. For statistical analyses, unpaired t-tests were conducted and p-values < 0.05 were considered significant.

## Results

### Validation of Recombinant KHKC Enzymatic Assay and Fructose-induced ATP Depletion Assay

To evaluate the overall quality of the assays, Z’-factor values were calculated utilizing data from fructose only and no fructose controls [39]. For the KHKC enzymatic assay, the Z’-factor was 0.906 ± 0.016. For the fructose-induced ATP depletion assay, the Z’-factor was 0.665 ± 0.112. Both assays were shown to be robust (Z’-factor > 0.5).

### Effects of Botanical Extracts and Phytochemicals on Inhibition of KHKC Activity

The botanical extracts and phytochemicals were tested for their ability to inhibit KHKC activity using the high throughput recombinant KHKC enzymatic assay. From the screening of the botanical extract library, over 20 extracts were determined to have KHKC inhibitory activity > 30% (Table 2, S1 and S2 Tables). The lipophilic and combination extracts of phloretin, a phenolic compound found in *Malus domestica*, were the most potent, with KHKC IC_50_s of 8.9 μg/mL and 9.2 μg/mL, respectively. The lipophilic, hydrophilic, and combination extracts of *Angelica archangelica* were all determined to have inhibitory activity against KHKC, with IC_50_s ranging from of 22.6 μg/mL—57.3 μg/mL. From the screening of the phytochemical library, 15 phytochemicals were identified that had KHKC inhibitory activity > 40% (Table 3, S3 and S4 Tables). Methoxy-isobavachalcone (*Psoralea2 corylifolia*) was found to be the most potent compound (IC_50_ = 0.2 μM) followed by osthole (*Angelica archangelica*, IC_50_ = 0.7 μM), cratoxyarborenone E (*Cratoxylum prunifolium*, IC_50_ = 1.0 μM), and α-/γ-mangostin (*Cratoxylum prunifolium*, IC_50_ = 1.5 μM). From the screenings of the botanical extracts and phytochemicals, 12 unique plant genera were identified to have strong inhibitory activity against KHKC.

**Table 2:** Inhibitory KHKC Activity of Candidate Botanical Extracts.

Fraction	Botanical	Genus	Species	Botanical Plate		KHKC Inhibition (%)	KHKC IC_50_ (μg/mL)
				Plate ID	Well		
Lipophilic	Phloretin**	*Malus*	*domestica*	FTL Plate 09	G11	87.9	8.9
Combination	Phloretin**	*Malus*	*domestica*	FTL Plate 09	E10	84.3	9.2
Lipophilic	Angelica Root(1 yr)	*Angelica*	*archangelica*	FTL Plate 26	B4	61.8	22.6
Hydrophilic	Angelica Root(1 yr)	*Angelica*	*archangelica*	FTL Plate 26	D4	77.6	23.2
Combination	Angelica Root (mature > 2yr)	*Angelica*	*archangelica*	FTL Plate 03	C3	64.6	30.3
Hydrophilic	Angelica Root (mature > 2yr)	*Angelica*	*archangelica*	FTL Plate 03	D2	57.9	30.4
Lipophilic	Rhodiola Kirilowii	*Rhodiola*	*Kirilowii*	FTL Plate 15	B9	70.3	32.2
Combination	Angelica Root(1 yr)	*Angelica*	*archangelica*	FTL Plate 26	F4	51.4	39.2
Hydrophilic	Troical Fruit Extract*	*Garcinia*	*mangostana*	FTL Plate 08	C11	53.7	50.9
Lipophilic	Angelica Root (mature > 2yr)	*Angelica*	*archangelica*	FTL Plate 03	B1	44.0	57.3
Lipophilic	Peppermint	*Mentha*	*lavanduliodora*	FTL Plate 24	D2	39.2	76.4
Combination	Troical Fruit Extract	*Garcinia*	*mangostana*	FTL Plate 08	B9	35.5	106.9
Hydrophilic	Onion Extract: Querline Allium cepa	*Allium*	*cepa*	FTL Plate 07	C7	32.2	109.2
Lipophilic	Baikal Skullcap	*Scutellaria*	*baicalensis*	FTL Plate 24	C6	31.7	120.9
Lipophilic	Chinese Mint	*Mentha*	*haplocalyx*	FTL Plate 23	G10	41.3	126.5
Lipophilic	Chocolate Mint	*Mentha*	*piperita*	FTL Plate 23	F9	36.5	129.5
Combination	Spearmint Mentha spicata	*Mentha*	*spicata*	FTL Plate 04	G9	36.2	141.4
Combination	Chinese Mint	*Mentha*	*haplocalyx*	FTL Plate 23	F11	31.6	164.2
Lipophilic	Double Mint	*Mentha*	*gracilis*	FTL Plate 23	B11	31.3	170.3
Combination	Chocolate Mint	*Mentha*	*piperita*	FTL Plate 23	E10	36.5	209.1

FTL: functional target library; proprietary library of Amway Corp. IC50: half maximal inhibitory concentration. KHKC: ketohexokinase isoform C.

* Tropical Fruit Extract is commercially available from Cyvex Nutrition, Inc. The major component is mangosteen fruit extract from Garcinia mangostana.

** Phloretin is commercially available from Cyvex Nutrition, Inc., and is a natural phenolic compound found in apple tree leaves.

**Table 3:** Inhibitory KHKC Activity of Candidate Phytochemicals.

Phytochemical	Genus	Species	Phytochemical Plate		KHKC Inhibition (%)	KHKC IC_50_ (μM)
			Plate ID	Well		
Methoxy isobavachalcone	*Psoralea*	*corylifolia*	AnalytiCon Plate #4	C9	70.8	0.2
Osthole	*Angelica*	*archangelica*	AnalytiCon Plate #2	G10	93.4	0.7
Cratoxyarborenone E	*Cratoxylum*	*prunifolium*	AnalytiCon Plate #2	C8	61.7	1.0
α-/γ-Mangostin	*Cratoxylum*	*prunifolium*	AnalytiCon Plate #4	F2	90.4	1.5
3,4',5,7-THMethoxy3'-O-B-D-Xylopyranoside	*Nymphaea*	*lotus*	AnalytiCon Plate #1	D10	63.3	3.5
Isobavachalcone	*Psoralea*	*corylifolia*	AnalytiCon Plate #10	B11	44.2	5.7
Osthenol	*Angelica*	*archangelica*	AnalytiCon Plate #5	G10	74.1	7.8
Oroxylin A	*Scutellaria*	*baicalensis*	AnalytiCon Plate #3	E1	42.1	8.2
5,7-Dimethoxy-8-prenylcoumarin	*Diospyros*	*attenuata*	AnalytiCon Plate #1	G7	70.4	10.3
Polyketide type molecule	*Myrica*	*cerifera*	AnalytiCon Plate #2	B8	61.2	11.3
Apigenin 7-glucuronide	*Andrographis*	*paniculata*	AnalytiCon Plate #4	F3	53.1	16.1
Apiin	*Petroselinum*	*crispum*	AnalytiCon Plate #6	E1	47.0	21.5
Flavaspidic acid AB	*Pteris*	*wallichiana*	AnalytiCon Plate #2	F8	48.7	29.5
Mulberrin	*Morus*	*alba*	AnalytiCon Plate #3	D7	61.2	31.2
Swietenocoumarin B	*Chloroxylon*	*swietenia*	AnalytiCon Plate #13	G6	62.5	58.4

AnalytiCon: Phytochemicals from MEGx library (AnalytiCon Discovery GmbH, Potsdam, Germany) were selected and purchased by Amway Corp. IC50: half maximal inhibitory concentration. KHKC: ketohexokinase isoform C.

**Fig 3:**
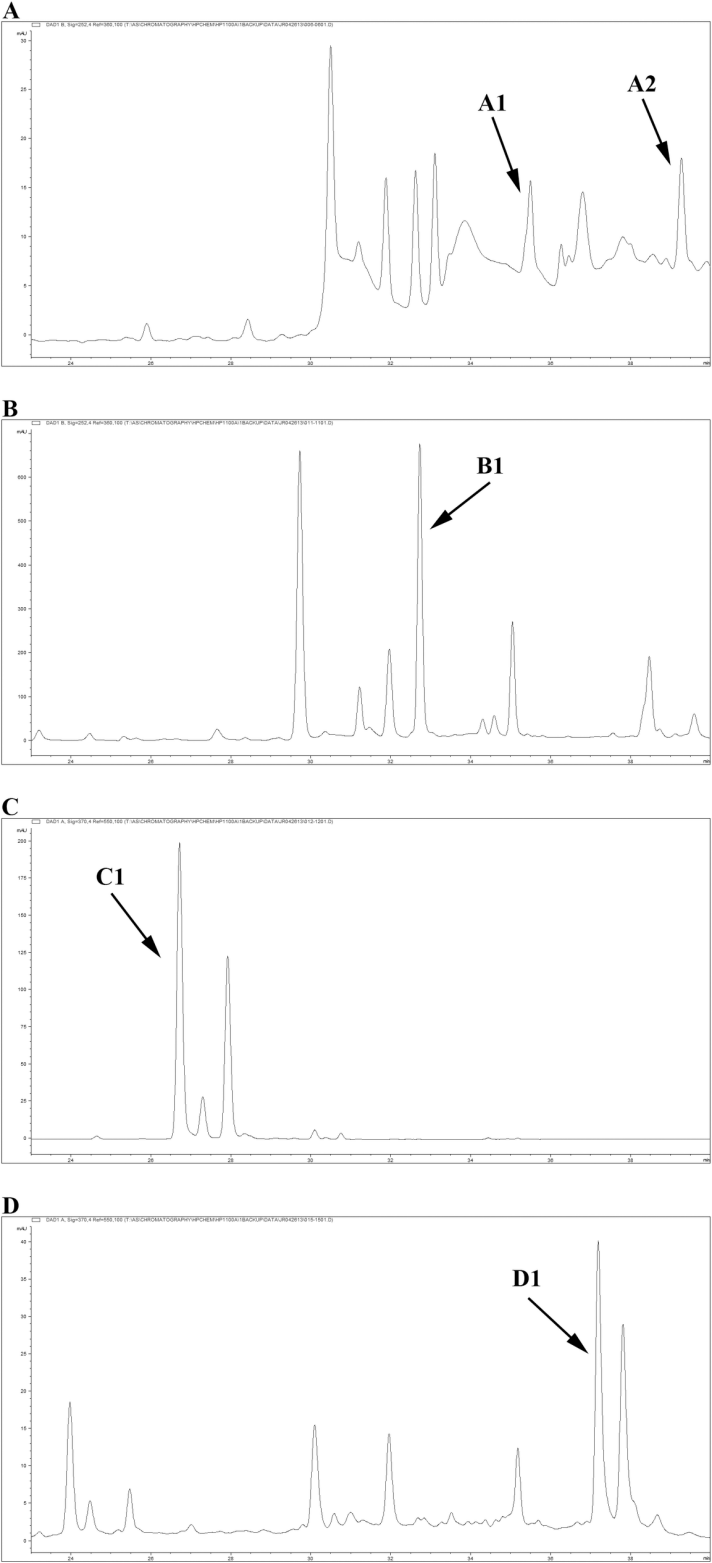
HPLC chromatograms of extracts of top botanical candidates. (A) *Angelica archangelica*, (B) *Scutellaria baicalensis*, (C) *Petroselinum crispum*, and (D) *Garcinia mangostana*. The arrows indicate the marker compounds. A1: osthenol, A2: osthole, B1: baicalin, C1: apiin, and D1: α-/γ-mangostin.

Based on factors such as robustness of commercial supply, safety, and reproducibility of KHKC inhibitory activity, several plant genera were eliminated from further analyses. *Cratoxylum prunifolium*, a plant native to tropical Asia and is in short supply, was replaced with *Garcinia mangostana* which was identified as also being enriched with α-/γ-mangostin. Thus, *Angelica archangelica*, *Scutellaria baicalensis*, *Petroselinum crispum*, and *Garcinia mangostana* were identified as the top four botanical candidates with strong inhibitory activity against KHKC (Table 4, S5–S8 Tables). HPLC fingerprint profiles of these four botanical extracts are shown in Fig 3.

**Table 4:** Inhibitory Activity of Extracts of Top Botanical Candidates.

Genus	Species	Lot #1			Lot #2			
		KHKC IC_50_ (μg/mL)	ATP Depletion IC_50_ (μg/mL)	TG IC_50_ (μg/mL)	KHKC IC_50_ (μg/mL)	ATP Depletion IC_50_ (μg/mL)	TG IC_50_ (μg/mL)	UA IC_50_ (μg/mL)
*Angelica*	*archangelica*	94.8 ± 4.2***	74.5 ± 13.2**	92.2 ± 76.8^NS^	505.6 ± 18.8***	778.1 ± 270.9*	58.8 ± 23.8^NS^	17.5 ± 22.5^NS^
*Scutellaria*	*baicalensis*	121.3 ± 4.6***	73.3 ± 6.4***	52.6 ± 31.4^NS^	23.4 ± 1.5*	28.2 ± 1.8***	16.3 ± 4.5^NS^	25.6 ± 35.9^NS^
*Petroselinum*	*crispum*	185.4 ± 4.7***	149.4 ± 72*	399.8 ± 20.2**	287.5 ± 16.8***	217.7 ± 4.9***	266.1 ± 35.7*	28.8 ± 3.5*
*Garcinia*	*mangostana*	18.7 ± 0.6	30.4 ± 2.7	73 ± 40.8	27.5 ± 2.1	63.1 ± 5.3	17.5 ± 7.1	3.8 ± 4.4

IC50: half maximal inhibitory concentration. KHKC: ketohexokinase isoform C. TG: triglycerides. UA: uric acid. Compared to Garcinia mangostana, p-value: * < 0.05, ** < 0.01, and *** < 0.0001. Data shown as mean ± standard deviation.

### Effects of Top Botanical Candidates on Cell Viability

**Fig 4:**
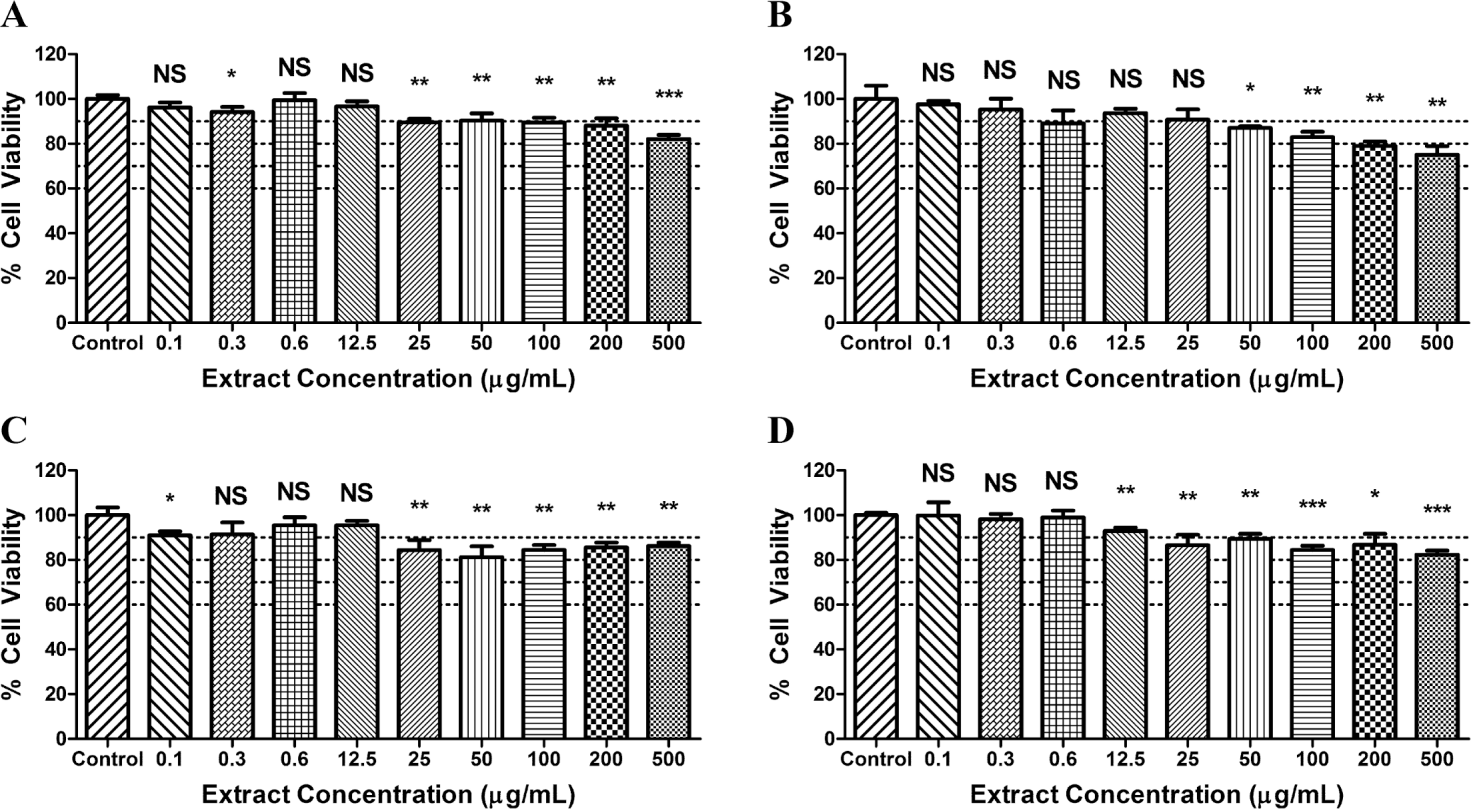
Cell viability after exposure to top botanical candidates. MTT assay was utilized to determine % cell viability of Hep G2 cells after 72 hr exposure to increasing doses of the top four botanical extracts (Lot #1). (A) *Angelica archangelica*, (B) *Scutellaria baicalensis*, (C) *Petroselinum crispum*, and (D) *Garcinia mangostana*.

A decrease in cell viability was generally seen with increasing doses of the botanical extracts (Fig 4). The most severe effect on cell viability was typically seen in cells exposed to 500 μg/ml. For *Angelica archangelica*, there was about an 18% decrease in cell viability. Meanwhile, there was a 25% decrease for *Scutellaria baicalensis*, an 18% decrease for *Garcinia* mangostana, and a 14% decrease in cell viability for *Petroselinum crispum*.

### Extracts of Top Botanical Candidates Inhibit KHKC Activity, Fructose-induced ATP depletion, and Fructose-induced elevation in TG and UA

Two lots of extracts from each of the top four botanical candidates were prepared and inhibitory activity were measured in the following assays.

#### Inhibition of KHKC activity and fructose-induced ATP depletion

Although there were differences in KHKC inhibitory activity between the two lots of botanical extracts, all extracts from Lot #1 and Lot #2 showed inhibitory activity against KHKC and fructose-induced ATP depletion (Table 4). *Garcinia mangostana* extract was significantly the most potent inhibitor from Lot#1 (KHKC IC_50_ = 18.7 μg/mL, ATP depletion IC_50_ = 30.4 μg/mL). *Scutellaria baicalensis* extract was the strongest inhibitor from Lot #2 (KHKC IC_50_ = 23.4 μg/mL, ATP depletion IC_50_ = 28.2 μg/mL).

#### Inhibition of fructose-induced elevation in triglycerides and uric acid

The metabolism of fructose by KHK is known to generate both TG and UA, the latter as a product of the depletion of ATP [40]. From the whole cell experiments, it was determined that all four botanicals had the ability to inhibit fructose-induced elevation of TG and UA. For Lot #1 extracts, *Scutellaria baicalensis* was the most potent inhibitor and had a TG IC_50_ of 52.6 μg/mL. For Lot #2 extracts, *Garcinia mangostana* had the strongest inhibitory activity against fructose-induced elevation in TG (IC_50_ = 17.5 μg/mL) and UA (IC_50_ = 3.8 μg/mL).

## Discussion

The intake of sugar, especially in the form of sugary beverages, is strongly linked with the development of obesity, diabetes, and cardiovascular disease [1, 3, 41]. For instance, higher intake of added sugars has been shown to impair glucose homeostasis and induce insulin resistance in children [42]. In the Framingham Heart Study, middle-aged adults consuming ≥ 1 sugar-sweetened beverages (SSBs) per day were at greater risk of developing metabolic syndrome and obesity than individuals consuming < 1 SSB per day [43]. Furthermore, in the Nurses’ Health Study, individuals who consumed ≥ 2 SSBs per day had a 35% greater risk of developing coronary heart disease compared to those who consumed < 1 SSB per month [44]. The consequences of added sugars has also reached remote rural areas of South Africa where increased intake of added sugars has been linked to increases in waist circumference and body mass index and decreases in HDL levels [45]. Thus, the harmful effects of added sugars have raised concerns over its excessive consumption and its nutritional and metabolic impacts on human health.

As major constituents of added sugars, the metabolic effects of glucose and fructose have been extensively studied. However, several studies have highlighted that fructose can cause worse metabolic effects than glucose. Stanhope et al. found that the consumption of fructose-sweetened beverages, but not glucose-sweetened beverages, for 10 weeks increased visceral adiposity and decreased insulin sensitivity in overweight and obese individuals [9]. Cox et al. also showed that individuals consuming fructose-sweetened versus glucose-sweetened beverages had significant increases in factors that contribute to the development of metabolic syndrome, such as uric acid levels, activation of gamma-glutamyl transferase, and monocyte chemoattractant protein-1 [46, 47]. Interestingly, Page et al. found that the ingestion of fructose generated significantly different hypothalamic brain signals than after the ingestion of glucose [48]. Unlike glucose, fructose ingestion did not result in an increase in the sensation of fullness and satiety. Because of the differences in severity of metabolic effects between these two carbohydrates, we focused on specifically inhibiting fructose activity.

Reducing the consumption of added sugars is a commonly recommended approach for managing obesity, diabetes, and other metabolic diseases. Studies have shown that vigorous physical activity and a healthier diet are effective at controlling and reducing weight [49]. However, the sustainability of these intensive lifestyle modifications is difficult, resulting in individuals regaining their initial weight loss [50, 51]. Furthermore, our lab has recently shown that glucose can be converted into endogeneous fructose by the polyol pathway [52]. Mice treated for 14 weeks with 10% glucose had higher intrahepatic fructose levels and developed fatty liver compared to controls. KHKA and KHKC double knockout mice, as well as aldose reductase (the first enzyme in the polyol pathway) knock-out mice, were protected from the development of fatty liver and hyperinsulinemia. Thus, the conversion of glucose into endogenous fructose may be the key mechanism by which glucose stimulates the development of metabolic syndrome. In addition, individuals with diabetes have been reported to have higher serum fructose levels, which was associated with increased risk for retinopathy [53]. These studies highlight the difficulty of controlling for the adverse effects of added sugars by reducing its consumption. Therefore, as a complementary approach to lifestyle modification, the development of a plant-based therapy that inhibits fructose metabolism is a promising translational approach to mitigate the adverse effects of sugar consumption in our diet.

For this study, we identified botanicals that inhibit the metabolism of fructose by KHKC. Although the study screened a limited number of botanical extracts, the botanical library was diverse and over 75% of the 406 plants were from different genera. Furthermore, the library represents most of the plants currently used in the investigation of phytomedicines by the herbal industry. Because sustainability is an important concern in the utilization of botanicals for the development of a phytomedicine, this botanical library also consisted of plants that are available from either Amway’s farms in Brazil, Mexico, and USA or from other commercial sources.

In order to identify botanicals with inhibitory activity against fructose metabolism, we developed a high-throughput enzymatic assay that utilized recombinant human KHKC. From the screenings of the botanical extract library and the phytochemical library, 12 botanical genera were determined to have strong inhibitory activity against KHKC. Of these botanicals, *Psoralea corylifolia* and *Morus alba* were eliminated because their inhibitory activity against KHKC was not reproducible. Other botanicals were eliminated due to supply chain issues and/or safety concerns. For instance, *Chloroxylon swietenia* is a threatened species and there are limited supplies of *Diospyros attenuate* which comes from a tree in South Africa and *Cratoxylum prunifolium* which is specific to the tropical region in Asia. Although we had eliminated *Cratoxylum prunifolium*, we were able to replace it with *Garcinia mangostana* which also contained the bioactive phytochemicals α-/γ-mangostin. *Garcinia*, an evergreen tree in Southeast Asia, is rich in antioxidant xanthones and is considered safe for human use [54]. *Nymphaea lotus*, *Pteris wallichiana*, *Andrographis paniculata* and, *Myrica cerifera* were also eliminated because of safety concerns. For instance, *Myrica* has been shown to have potential anaphylaxis and anticoagulant effects [55, 56]. Overall, *Angelica archangelica*, *Scutellaria baicalensis*, *Petroselinum crispum*, and *Garcinia mangostana* remained as the top botanical candidates.

To further characterize the abilities of these four botanicals to inhibit downstream effects of fructose metabolism, we developed two other assays. To determine the ability of KHKC-inhibiting botanicals to inhibit fructose-induced ATP depletion, we utilized lysates of Hep G2 cells that were transformed to stably over express KHKC. The second assay was a whole cell assay that utilized Hep G2 cells to determine if the botanical extract could inhibit fructose-induced elevation of TG or UA. We determined that all four botanicals were able to inhibit fructose-induced ATP depletion and fructose-induced elevation in TG and UA.

In summary, after screening libraries containing 406 botanical extracts and 1200 phytochemicals, we identified four botanicals with inhibitory activity against KHKC that demonstrated protective effects against fructose-induced ATP depletion and elevation in TG and UA. These four leading candidates–A*ngelica archangelica*, *Scutellaria baicalensis*, *Petroselinum crispum*, and *Garcinia mangostana*–are also safe for human consumption. In addition, their relative low cost and sustainability makes them excellent botanical candidates for taking to clinical trial to determine if they can block the adverse effects of added sugars in humans.

Significantly, this is the first study to identify botanicals and phytochemicals that directly inhibit KHKC and can block downstream effects of fructose to increase triglycerides and uric acid. Further studies are necessary to determine the safety, specificity, and efficacy of these botanicals to block fructose-dependent disorders, with the hope that these botanical ingredients may one day be useful to help arrest the obesity and diabetes epidemic.

## Supporting Information

S1 Table Data from Screening of Botanical Extract Library for Inhibition of KHKC Activity. FTL: functional target library; proprietary library of Amway Corp. KHKC: ketohexokinase isoform C. FOC: fructose only control. NFC: no fructose control. OD: optical density.
(PDF)

S2 Table Data from Titrations of Candidate Botanical Extracts for Inhibition of KHKC Activity. FTL: functional target library; proprietary library of Amway Corp. IC50: half maximal inhibitory concentration. OD: optical density. *KHKC IC50s were calculated using nonlinear regression (three parameters) in GraphPad Prism 5.03. To generate a best fit, an upper concentration (10,000 μg/mL at 100% inhibition) and a lower concentration (0.001 μg/mL at 0% inhibition) were added.
(PDF)

S3 Table Data from Screening of Phytochemical Library for Inhibition of KHKC Activity. AnalytiCon: Phytochemicals from MEGx library (AnalytiCon Discovery GmbH, Potsdam, Germany) were selected and purchased by Amway Corp. KHKC: ketohexokinase isoform C. FOC: fructose only control. NFC: no fructose control. OD: optical density.
(PDF)

S4 Table Data from Titrations of Candidate Phytochemicals for Inhibition of KHKC Activity. AnalytiCon: Phytochemicals from MEGx library (AnalytiCon Discovery GmbH, Potsdam, Germany) were selected and purchased by Amway Corp. IC50: half maximal inhibitory concentration. OD: optical density. *KHKC IC50s were calculated using nonlinear regression (three parameters) in GraphPad Prism 5.03. To generate a best fit, an upper concentration (10,000 μg/mL at 100% inhibition) and a lower concentration (0.001 μg/mL at 0% inhibition) were added.
(PDF)

S5 Table Data from Titrations of Top Botanical Candidates for Inhibition of KHKC Activity. IC50: half maximal inhibitory concentration. OD: optical density. *KHKC IC50s were calculated using nonlinear regression (three parameters) in GraphPad Prism 5.03. To generate a best fit, an upper concentration (10,000 μg/mL at 100% inhibition) and a lower concentration (0.001 μg/mL at 0% inhibition) were added.
(PDF)

S6 Table Data from Titrations of Top Botanical Candidates for Inhibition of Fructose-Induced ATP Depletion. IC50: half maximal inhibitory concentration. OD: optical density. *ATP Depletion IC50s were calculated using nonlinear regression (three parameters) in GraphPad Prism 5.03. To generate a best fit, an upper concentration (10,000 μg/mL at 100% inhibition) and a lower concentration (0.001 μg/mL at 0% inhibition) were added.
(PDF)

S7 Table Data from Titrations of Top Botanical Candidates for Inhibition of Fructose-induced Elevation in TG Levels. IC50: half maximal inhibitory concentration. OD: optical density. TG: triglycerides. *TG IC50s were calculated using nonlinear regression (three parameters) in GraphPad Prism 5.03. To generate a best fit, an upper concentration (10,000 μg/mL at 100% inhibition) and a lower concentration (0.001 μg/mL at 0% inhibition) were added.
(PDF)

S8 Table Data from Titrations of Top Botanical Candidates for Inhibition of Fructose-induced Elevation in UA Levels. IC50: half maximal inhibitory concentration. OD: optical density. UA: uric acid. *UA IC50s were calculated using nonlinear regression (three parameters) in GraphPad Prism 5.03. To generate a best fit, an upper concentration (10,000 μg/mL at 100% inhibition) and a lower concentration (0.001 μg/mL at 0% inhibition) were added.
(PDF)
