# Supplementary material for: Bioactivity-Guided Identification of Botanical Inhibitors of Ketohexokinase
Source: PLoS One. 2016 Jun 20;11(6):e0157458. doi: 10.1371/journal.pone.0157458 (PMC4913896; doi:10.1371/journal.pone.0157458)
Supplement: S1 Table — FTL: functional target library; proprietary library of Amway Corp. KHKC: ketohexokinase isoform C. FOC: fructose only control. NFC: no fructose control. OD: optical density. (PDF) [file pone.0157458.s001.pdf]

S1 Table. Data from Screening of Botanical Extract Library for Inhibition of KHKC Activity.

Rxn ID	Sample			Sample	Ave. No Fructose Controls	Ave. Fructose Only Controls	KHKC
	Botanical Plate	Well	Conc (μg/mL)	(ΔOD_{340nm})	(ΔOD_{340nm})	(ΔOD_{340nm})	Inhibition (%)
Rxn #02	FTL Plate 21	A1: NFC	-	0.062	0.0626	0.557	100.1
Rxn #02	FTL Plate 21	B1	50	0.475	0.0626	0.557	16.6
Rxn #02	FTL Plate 21	C1	50	0.506	0.0626	0.557	10.3
Rxn #02	FTL Plate 21	D1	50	0.476	0.0626	0.557	16.4
Rxn #02	FTL Plate 21	E1	50	0.493	0.0626	0.557	12.9
Rxn #02	FTL Plate 21	F1	50	0.520	0.0626	0.557	7.5
Rxn #02	FTL Plate 21	G1	50	0.484	0.0626	0.557	14.8
Rxn #02	FTL Plate 21	H1: FOC	-	0.555	0.0626	0.557	0.4
Rxn #02	FTL Plate 21	A2: NFC	-	0.067	0.0626	0.557	99.1
Rxn #02	FTL Plate 21	B2	50	0.559	0.0626	0.557	-0.4
Rxn #02	FTL Plate 21	C2	50	0.580	0.0626	0.557	-4.7
Rxn #02	FTL Plate 21	D2	50	0.541	0.0626	0.557	3.2
Rxn #02	FTL Plate 21	E2	50	0.546	0.0626	0.557	2.2
Rxn #02	FTL Plate 21	F2	50	0.522	0.0626	0.557	7.1
Rxn #02	FTL Plate 21	G2	50	0.534	0.0626	0.557	4.7
Rxn #02	FTL Plate 21	H2: FOC	-	0.577	0.0626	0.557	-4.0
Rxn #02	FTL Plate 21	A3: NFC	-	0.062	0.0626	0.557	100.1
Rxn #02	FTL Plate 21	B3	50	0.609	0.0626	0.557	-10.5
Rxn #02	FTL Plate 21	C3	50	0.558	0.0626	0.557	-0.2
Rxn #02	FTL Plate 21	D3	50	0.589	0.0626	0.557	-6.5
Rxn #02	FTL Plate 21	E3	50	0.570	0.0626	0.557	-2.6
Rxn #02	FTL Plate 21	F3	50	0.557	0.0626	0.557	0.0
Rxn #02	FTL Plate 21	G3	50	0.547	0.0626	0.557	2.0
Rxn #02	FTL Plate 21	H3: FOC	-	0.573	0.0626	0.557	-3.2
Rxn #02	FTL Plate 21	A4: NFC	-	0.062	0.0626	0.557	100.1
Rxn #02	FTL Plate 21	B4	50	0.592	0.0626	0.557	-7.1
Rxn #02	FTL Plate 21	C4	50	0.577	0.0626	0.557	-4.0
Rxn #02	FTL Plate 21	D4	50	0.580	0.0626	0.557	-4.7
Rxn #02	FTL Plate 21	E4	50	0.567	0.0626	0.557	-2.0
Rxn #02	FTL Plate 21	F4	50	0.556	0.0626	0.557	0.2
Rxn #02	FTL Plate 21	G4	50	0.547	0.0626	0.557	2.0
Rxn #02	FTL Plate 21	H4: FOC	-	0.563	0.0626	0.557	-1.2
Rxn #02	FTL Plate 21	A5: NFC	-	0.056	0.0626	0.557	101.3
Rxn #02	FTL Plate 21	B5: NFC	-	0.058	0.0626	0.557	100.9
Rxn #02	FTL Plate 21	C5: NFC	-	0.058	0.0626	0.557	100.9
Rxn #02	FTL Plate 21	D5: NFC	-	0.053	0.0626	0.557	101.9
Rxn #02	FTL Plate 21	E5: FOC	-	0.566	0.0626	0.557	-1.8
Rxn #02	FTL Plate 21	F5: FOC	-	0.552	0.0626	0.557	1.0
Rxn #02	FTL Plate 21	G5: FOC	-	0.556	0.0626	0.557	0.2
Rxn #02	FTL Plate 21	H5: FOC	-	0.555	0.0626	0.557	0.4
Rxn #02	FTL Plate 21	A6: NFC	-	0.055	0.0626	0.557	101.5
Rxn #02	FTL Plate 21	B6	50	0.579	0.0626	0.557	-4.4
Rxn #02	FTL Plate 21	C6	50	0.561	0.0626	0.557	-0.8
Rxn #02	FTL Plate 21	D6	50	0.579	0.0626	0.557	-4.4
Rxn #02	FTL Plate 21	E6	50	0.460	0.0626	0.557	19.6
Rxn #02	FTL Plate 21	F6	50	0.537	0.0626	0.557	4.0
Rxn #02	FTL Plate 21	G6	50	0.541	0.0626	0.557	3.2
Rxn #02	FTL Plate 21	H6: FOC	-	0.558	0.0626	0.557	-0.2
Rxn #02	FTL Plate 21	A7: NFC	-	0.055	0.0626	0.557	101.5
Rxn #02	FTL Plate 21	B7	50	0.553	0.0626	0.557	0.8
Rxn #02	FTL Plate 21	C7	50	0.526	0.0626	0.557	6.3
Rxn #02	FTL Plate 21	D7	50	0.531	0.0626	0.557	5.3
Rxn #02	FTL Plate 21	E7	50	0.564	0.0626	0.557	-1.4
Rxn #02	FTL Plate 21	F7	50	0.544	0.0626	0.557	2.6
Rxn #02	FTL Plate 21	G7	50	0.554	0.0626	0.557	0.6
Rxn #02	FTL Plate 21	H7: FOC	-	0.564	0.0626	0.557	-1.4
Rxn #02	FTL Plate 21	A8: NFC	-	0.061	0.0626	0.557	100.3
Rxn #02	FTL Plate 21	B8	50	0.542	0.0626	0.557	3.0
Rxn #02	FTL Plate 21	C8	50	0.570	0.0626	0.557	-2.6
Rxn #02	FTL Plate 21	D8	50	0.541	0.0626	0.557	3.2
Rxn #02	FTL Plate 21	E8	50	0.613	0.0626	0.557	-11.3
Rxn #02	FTL Plate 21	F8	50	0.564	0.0626	0.557	-1.4
Rxn #02	FTL Plate 21	G8	50	0.600	0.0626	0.557	-8.7
Rxn #02	FTL Plate 21	H8: FOC	-	0.551	0.0626	0.557	1.2
Rxn #02	FTL Plate 21	A9: NFC	-	0.071	0.0626	0.557	98.3
Rxn #02	FTL Plate 21	B9	50	0.577	0.0626	0.557	-4.0
Rxn #02	FTL Plate 21	C9	50	0.525	0.0626	0.557	6.5
Rxn #02	FTL Plate 21	D9	50	0.561	0.0626	0.557	-0.8
Rxn #02	FTL Plate 21	E9	50	0.548	0.0626	0.557	1.8
Rxn #02	FTL Plate 21	F9	50	0.560	0.0626	0.557	-0.6

S1 Table. Data from Screening of Botanical Extract Library for Inhibition of KHKC Activity.

Rxn ID	Sample			Sample	Ave. No Fructose Controls	Ave. Fructose Only Controls	KHKC
	Botanical Plate	Well	Conc (µg/mL)	(ΔOD_{340nm})	(ΔOD_{340nm})	(ΔOD_{340nm})	Inhibition (%)
Rxn #02	FTL Plate 21	G9	50	0.533	0.0626	0.557	4.9
Rxn #02	FTL Plate 21	H9: FOC	-	0.545	0.0626	0.557	2.4
Rxn #02	FTL Plate 21	A10: NFC	-	0.069	0.0626	0.557	98.7
Rxn #02	FTL Plate 21	B10	50	0.549	0.0626	0.557	1.6
Rxn #02	FTL Plate 21	C10	50	0.546	0.0626	0.557	2.2
Rxn #02	FTL Plate 21	D10	50	0.549	0.0626	0.557	1.6
Rxn #02	FTL Plate 21	E10	50	0.535	0.0626	0.557	4.4
Rxn #02	FTL Plate 21	F10	50	0.560	0.0626	0.557	-0.6
Rxn #02	FTL Plate 21	G10	50	0.539	0.0626	0.557	3.6
Rxn #02	FTL Plate 21	H10: FOC	-	0.549	0.0626	0.557	1.6
Rxn #02	FTL Plate 21	A11: NFC	-	0.074	0.0626	0.557	97.7
Rxn #02	FTL Plate 21	B11	50	0.574	0.0626	0.557	-3.4
Rxn #02	FTL Plate 21	C11	50	0.578	0.0626	0.557	-4.2
Rxn #02	FTL Plate 21	D11	50	0.552	0.0626	0.557	1.0
Rxn #02	FTL Plate 21	E11	50	0.510	0.0626	0.557	9.5
Rxn #02	FTL Plate 21	F11	50	0.538	0.0626	0.557	3.8
Rxn #02	FTL Plate 21	G11	50	0.542	0.0626	0.557	3.0
Rxn #02	FTL Plate 21	H11: FOC	-	0.545	0.0626	0.557	2.4
Rxn #02	FTL Plate 21	A12: NFC	-	0.076	0.0626	0.557	97.3
Rxn #02	FTL Plate 21	H12: FOC	-	0.546	0.0626	0.557	2.2
Rxn #04	FTL Plate 4	A1: NFC	-	0.058	0.0563	0.504	99.6
Rxn #04	FTL Plate 4	B1	50	0.474	0.0563	0.504	6.7
Rxn #04	FTL Plate 4	C1	50	0.448	0.0563	0.504	12.5
Rxn #04	FTL Plate 4	D1	50	0.439	0.0563	0.504	14.5
Rxn #04	FTL Plate 4	E1	50	0.468	0.0563	0.504	8.0
Rxn #04	FTL Plate 4	F1	50	0.439	0.0563	0.504	14.5
Rxn #04	FTL Plate 4	G1	50	0.453	0.0563	0.504	11.4
Rxn #04	FTL Plate 4	H1: FOC	-	0.481	0.0563	0.504	5.1
Rxn #04	FTL Plate 4	A2: NFC	-	0.064	0.0563	0.504	98.3
Rxn #04	FTL Plate 4	B2	50	0.469	0.0563	0.504	7.8
Rxn #04	FTL Plate 4	C2	50	0.456	0.0563	0.504	10.7
Rxn #04	FTL Plate 4	D2	50	0.485	0.0563	0.504	4.2
Rxn #04	FTL Plate 4	E2	50	0.448	0.0563	0.504	12.5
Rxn #04	FTL Plate 4	F2	50	0.486	0.0563	0.504	4.0
Rxn #04	FTL Plate 4	G2	50	0.449	0.0563	0.504	12.3
Rxn #04	FTL Plate 4	H2: FOC	-	0.509	0.0563	0.504	-1.1
Rxn #04	FTL Plate 4	A3: NFC	-	0.054	0.0563	0.504	100.5
Rxn #04	FTL Plate 4	B3	50	0.484	0.0563	0.504	4.5
Rxn #04	FTL Plate 4	C3	50	0.511	0.0563	0.504	-1.6
Rxn #04	FTL Plate 4	D3	50	0.478	0.0563	0.504	5.8
Rxn #04	FTL Plate 4	E3	50	0.522	0.0563	0.504	-4.0
Rxn #04	FTL Plate 4	F3	50	0.495	0.0563	0.504	2.0
Rxn #04	FTL Plate 4	G3	50	0.432	0.0563	0.504	16.1
Rxn #04	FTL Plate 4	H3: FOC	-	0.504	0.0563	0.504	0.0
Rxn #04	FTL Plate 4	A4: NFC	-	0.063	0.0563	0.504	98.5
Rxn #04	FTL Plate 4	B4	50	0.512	0.0563	0.504	-1.8
Rxn #04	FTL Plate 4	C4	50	0.467	0.0563	0.504	8.3
Rxn #04	FTL Plate 4	D4	50	0.504	0.0563	0.504	0.0
Rxn #04	FTL Plate 4	E4	50	0.468	0.0563	0.504	8.0
Rxn #04	FTL Plate 4	F4	50	0.515	0.0563	0.504	-2.5
Rxn #04	FTL Plate 4	G4	50	0.453	0.0563	0.504	11.4
Rxn #04	FTL Plate 4	H4: FOC	-	0.515	0.0563	0.504	-2.5
Rxn #04	FTL Plate 4	A5: NFC	-	0.051	0.0563	0.504	101.2
Rxn #04	FTL Plate 4	B5: NFC	-	0.057	0.0563	0.504	99.8
Rxn #04	FTL Plate 4	C5: NFC	-	0.052	0.0563	0.504	101.0
Rxn #04	FTL Plate 4	D5: NFC	-	0.043	0.0563	0.504	103.0
Rxn #04	FTL Plate 4	E5: FOC	-	0.522	0.0563	0.504	-4.0
Rxn #04	FTL Plate 4	F5: FOC	-	0.506	0.0563	0.504	-0.4
Rxn #04	FTL Plate 4	G5: FOC	-	0.496	0.0563	0.504	1.8
Rxn #04	FTL Plate 4	H5: FOC	-	0.508	0.0563	0.504	-0.9
Rxn #04	FTL Plate 4	A6: NFC	-	0.057	0.0563	0.504	99.8
Rxn #04	FTL Plate 4	B6	50	0.508	0.0563	0.504	-0.9
Rxn #04	FTL Plate 4	C6	50	0.504	0.0563	0.504	0.0
Rxn #04	FTL Plate 4	D6	50	0.471	0.0563	0.504	7.4
Rxn #04	FTL Plate 4	E6	50	0.505	0.0563	0.504	-0.2
Rxn #04	FTL Plate 4	F6	50	0.497	0.0563	0.504	1.6
Rxn #04	FTL Plate 4	G6	50	0.445	0.0563	0.504	13.2
Rxn #04	FTL Plate 4	H6: FOC	-	0.506	0.0563	0.504	-0.4
Rxn #04	FTL Plate 4	A7: NFC	-	0.051	0.0563	0.504	101.2
Rxn #04	FTL Plate 4	B7	50	0.503	0.0563	0.504	0.2

S1 Table. Data from Screening of Botanical Extract Library for Inhibition of KHKC Activity.

Rxn ID	Sample			Sample	Ave. No Fructose Controls	Ave. Fructose Only Controls	KHKC
	Botanical Plate	Well	Conc (μg/mL)	(ΔOD_{340nm})	(ΔOD_{340nm})	(ΔOD_{340nm})	Inhibition (%)
Rxn #04	FTL Plate 4	C7	50	0.474	0.0563	0.504	6.7
Rxn #04	FTL Plate 4	D7	50	0.501	0.0563	0.504	0.7
Rxn #04	FTL Plate 4	E7	50	0.512	0.0563	0.504	-1.8
Rxn #04	FTL Plate 4	F7	50	0.468	0.0563	0.504	8.0
Rxn #04	FTL Plate 4	G7	50	0.482	0.0563	0.504	4.9
Rxn #04	FTL Plate 4	H7: FOC	-	0.507	0.0563	0.504	-0.7
Rxn #04	FTL Plate 4	A8: NFC	-	0.052	0.0563	0.504	101.0
Rxn #04	FTL Plate 4	B8	50	0.495	0.0563	0.504	2.0
Rxn #04	FTL Plate 4	C8	50	0.494	0.0563	0.504	2.2
Rxn #04	FTL Plate 4	D8	50	0.468	0.0563	0.504	8.0
Rxn #04	FTL Plate 4	E8	50	0.486	0.0563	0.504	4.0
Rxn #04	FTL Plate 4	F8	50	0.479	0.0563	0.504	5.6
Rxn #04	FTL Plate 4	G8	50	0.448	0.0563	0.504	12.5
Rxn #04	FTL Plate 4	H8: FOC	-	0.506	0.0563	0.504	-0.4
Rxn #04	FTL Plate 4	A9: NFC	-	0.057	0.0563	0.504	99.8
Rxn #04	FTL Plate 4	B9	50	0.507	0.0563	0.504	-0.7
Rxn #04	FTL Plate 4	C9	50	0.504	0.0563	0.504	0.0
Rxn #04	FTL Plate 4	D9	50	0.503	0.0563	0.504	0.2
Rxn #04	FTL Plate 4	E9	50	0.518	0.0563	0.504	-3.1
Rxn #04	FTL Plate 4	F9	50	0.479	0.0563	0.504	5.6
Rxn #04	FTL Plate 4	G9	50	0.342	0.0563	0.504	36.2
Rxn #04	FTL Plate 4	H9: FOC	-	0.502	0.0563	0.504	0.4
Rxn #04	FTL Plate 4	A10: NFC	-	0.059	0.0563	0.504	99.4
Rxn #04	FTL Plate 4	B10	50	0.484	0.0563	0.504	4.5
Rxn #04	FTL Plate 4	C10	50	0.519	0.0563	0.504	-3.4
Rxn #04	FTL Plate 4	D10	50	0.499	0.0563	0.504	1.1
Rxn #04	FTL Plate 4	E10	50	0.513	0.0563	0.504	-2.0
Rxn #04	FTL Plate 4	F10	50	0.417	0.0563	0.504	19.4
Rxn #04	FTL Plate 4	G10	50	0.474	0.0563	0.504	6.7
Rxn #04	FTL Plate 4	H10: FOC	-	0.508	0.0563	0.504	-0.9
Rxn #04	FTL Plate 4	A11: NFC	-	0.065	0.0563	0.504	98.1
Rxn #04	FTL Plate 4	B11	50	0.500	0.0563	0.504	0.9
Rxn #04	FTL Plate 4	C11	50	0.507	0.0563	0.504	-0.7
Rxn #04	FTL Plate 4	D11	50	0.501	0.0563	0.504	0.7
Rxn #04	FTL Plate 4	E11	50	0.398	0.0563	0.504	23.7
Rxn #04	FTL Plate 4	F11	50	0.502	0.0563	0.504	0.4
Rxn #04	FTL Plate 4	G11	50	0.486	0.0563	0.504	4.0
Rxn #04	FTL Plate 4	H11: FOC	-	0.498	0.0563	0.504	1.3
Rxn #04	FTL Plate 4	A12: NFC	-	0.061	0.0563	0.504	99.0
Rxn #04	FTL Plate 4	H12: FOC	-	0.495	0.0563	0.504	2.0
Rxn #05	FTL Plate 16	A1: NFC	-	0.062	0.0541	0.554	98.4
Rxn #05	FTL Plate 16	B1	50	0.545	0.0541	0.554	1.8
Rxn #05	FTL Plate 16	C1	50	0.511	0.0541	0.554	8.6
Rxn #05	FTL Plate 16	D1	50	0.561	0.0541	0.554	-1.4
Rxn #05	FTL Plate 16	E1	50	0.543	0.0541	0.554	2.2
Rxn #05	FTL Plate 16	F1	50	0.526	0.0541	0.554	5.6
Rxn #05	FTL Plate 16	G1	50	0.524	0.0541	0.554	6.0
Rxn #05	FTL Plate 16	H1: FOC	-	0.534	0.0541	0.554	4.0
Rxn #05	FTL Plate 16	A2: NFC	-	0.052	0.0541	0.554	100.4
Rxn #05	FTL Plate 16	B2	50	0.566	0.0541	0.554	-2.4
Rxn #05	FTL Plate 16	C2	50	0.569	0.0541	0.554	-3.0
Rxn #05	FTL Plate 16	D2	50	0.554	0.0541	0.554	0.0
Rxn #05	FTL Plate 16	E2	50	0.529	0.0541	0.554	5.0
Rxn #05	FTL Plate 16	F2	50	0.562	0.0541	0.554	-1.6
Rxn #05	FTL Plate 16	G2	50	0.546	0.0541	0.554	1.6
Rxn #05	FTL Plate 16	H2: FOC	-	0.562	0.0541	0.554	-1.6
Rxn #05	FTL Plate 16	A3: NFC	-	0.058	0.0541	0.554	99.2
Rxn #05	FTL Plate 16	B3	50	0.552	0.0541	0.554	0.4
Rxn #05	FTL Plate 16	C3	50	0.560	0.0541	0.554	-1.2
Rxn #05	FTL Plate 16	D3	50	0.565	0.0541	0.554	-2.2
Rxn #05	FTL Plate 16	E3	50	0.557	0.0541	0.554	-0.6
Rxn #05	FTL Plate 16	F3	50	0.545	0.0541	0.554	1.8
Rxn #05	FTL Plate 16	G3	50	0.536	0.0541	0.554	3.6
Rxn #05	FTL Plate 16	H3: FOC	-	0.577	0.0541	0.554	-4.6
Rxn #05	FTL Plate 16	A4: NFC	-	0.064	0.0541	0.554	98.0
Rxn #05	FTL Plate 16	B4	50	0.562	0.0541	0.554	-1.6
Rxn #05	FTL Plate 16	C4	50	0.511	0.0541	0.554	8.6
Rxn #05	FTL Plate 16	D4	50	0.549	0.0541	0.554	1.0
Rxn #05	FTL Plate 16	E4	50	0.511	0.0541	0.554	8.6
Rxn #05	FTL Plate 16	F4	50	0.541	0.0541	0.554	2.6

S1 Table. Data from Screening of Botanical Extract Library for Inhibition of KHKC Activity.

Rxn ID	Sample			Sample	Ave. No Fructose Controls	Ave. Fructose Only Controls	KHKC
	Botanical Plate	Well	Conc (µg/mL)	(ΔOD_{340nm})	(ΔOD_{340nm})	(ΔOD_{340nm})	Inhibition (%)
Rxn #05	FTL Plate 16	G4	50	0.491	0.0541	0.554	12.6
Rxn #05	FTL Plate 16	H4: FOC	-	0.553	0.0541	0.554	0.2
Rxn #05	FTL Plate 16	A5: NFC	-	0.060	0.0541	0.554	98.8
Rxn #05	FTL Plate 16	B5: NFC	-	0.047	0.0541	0.554	101.4
Rxn #05	FTL Plate 16	C5: NFC	-	0.044	0.0541	0.554	102.0
Rxn #05	FTL Plate 16	D5: NFC	-	0.045	0.0541	0.554	101.8
Rxn #05	FTL Plate 16	E5: FOC	-	0.550	0.0541	0.554	0.8
Rxn #05	FTL Plate 16	F5: FOC	-	0.549	0.0541	0.554	1.0
Rxn #05	FTL Plate 16	G5: FOC	-	0.568	0.0541	0.554	-2.8
Rxn #05	FTL Plate 16	H5: FOC	-	0.545	0.0541	0.554	1.8
Rxn #05	FTL Plate 16	A6: NFC	-	0.059	0.0541	0.554	99.0
Rxn #05	FTL Plate 16	B6	50	0.611	0.0541	0.554	-11.4
Rxn #05	FTL Plate 16	C6	50	0.571	0.0541	0.554	-3.4
Rxn #05	FTL Plate 16	D6	50	0.581	0.0541	0.554	-5.4
Rxn #05	FTL Plate 16	E6	50	0.462	0.0541	0.554	18.4
Rxn #05	FTL Plate 16	F6	50	0.558	0.0541	0.554	-0.8
Rxn #05	FTL Plate 16	G6	50	0.540	0.0541	0.554	2.8
Rxn #05	FTL Plate 16	H6: FOC	-	0.567	0.0541	0.554	-2.6
Rxn #05	FTL Plate 16	A7: NFC	-	0.044	0.0541	0.554	102.0
Rxn #05	FTL Plate 16	B7	50	0.563	0.0541	0.554	-1.8
Rxn #05	FTL Plate 16	C7	50	0.583	0.0541	0.554	-5.8
Rxn #05	FTL Plate 16	D7	50	0.542	0.0541	0.554	2.4
Rxn #05	FTL Plate 16	E7	50	0.546	0.0541	0.554	1.6
Rxn #05	FTL Plate 16	F7	50	0.520	0.0541	0.554	6.8
Rxn #05	FTL Plate 16	G7	50	0.533	0.0541	0.554	4.2
Rxn #05	FTL Plate 16	H7: FOC	-	0.556	0.0541	0.554	-0.4
Rxn #05	FTL Plate 16	A8: NFC	-	0.045	0.0541	0.554	101.8
Rxn #05	FTL Plate 16	B8	50	0.567	0.0541	0.554	-2.6
Rxn #05	FTL Plate 16	C8	50	0.558	0.0541	0.554	-0.8
Rxn #05	FTL Plate 16	D8	50	0.580	0.0541	0.554	-5.2
Rxn #05	FTL Plate 16	E8	50	0.547	0.0541	0.554	1.4
Rxn #05	FTL Plate 16	F8	50	0.531	0.0541	0.554	4.6
Rxn #05	FTL Plate 16	G8	50	0.569	0.0541	0.554	-3.0
Rxn #05	FTL Plate 16	H8: FOC	-	0.557	0.0541	0.554	-0.6
Rxn #05	FTL Plate 16	A9: NFC	-	0.056	0.0541	0.554	99.6
Rxn #05	FTL Plate 16	B9	50	0.609	0.0541	0.554	-11.0
Rxn #05	FTL Plate 16	C9	50	0.549	0.0541	0.554	1.0
Rxn #05	FTL Plate 16	D9	50	0.565	0.0541	0.554	-2.2
Rxn #05	FTL Plate 16	E9	50	0.545	0.0541	0.554	1.8
Rxn #05	FTL Plate 16	F9	50	0.551	0.0541	0.554	0.6
Rxn #05	FTL Plate 16	G9	50	0.555	0.0541	0.554	-0.2
Rxn #05	FTL Plate 16	H9: FOC	-	0.561	0.0541	0.554	-1.4
Rxn #05	FTL Plate 16	A10: NFC	-	0.052	0.0541	0.554	100.4
Rxn #05	FTL Plate 16	B10	50	0.560	0.0541	0.554	-1.2
Rxn #05	FTL Plate 16	C10	50	0.550	0.0541	0.554	0.8
Rxn #05	FTL Plate 16	D10	50	0.528	0.0541	0.554	5.2
Rxn #05	FTL Plate 16	E10	50	0.529	0.0541	0.554	5.0
Rxn #05	FTL Plate 16	F10	50	0.545	0.0541	0.554	1.8
Rxn #05	FTL Plate 16	G10	50	0.555	0.0541	0.554	-0.2
Rxn #05	FTL Plate 16	H10: FOC	-	0.536	0.0541	0.554	3.6
Rxn #05	FTL Plate 16	A11: NFC	-	0.065	0.0541	0.554	97.8
Rxn #05	FTL Plate 16	B11	50	0.563	0.0541	0.554	-1.8
Rxn #05	FTL Plate 16	C11	50	0.576	0.0541	0.554	-4.4
Rxn #05	FTL Plate 16	D11	50	0.555	0.0541	0.554	-0.2
Rxn #05	FTL Plate 16	E11	50	0.549	0.0541	0.554	1.0
Rxn #05	FTL Plate 16	F11	50	0.548	0.0541	0.554	1.2
Rxn #05	FTL Plate 16	G11	50	0.539	0.0541	0.554	3.0
Rxn #05	FTL Plate 16	H11: FOC	-	0.546	0.0541	0.554	1.6
Rxn #05	FTL Plate 16	A12: NFC	-	0.058	0.0541	0.554	99.2
Rxn #05	FTL Plate 16	H12: FOC	-	0.542	0.0541	0.554	2.4
Rxn #06	FTL Plate 19	A1: NFC	-	0.043	0.0458	0.513	100.6
Rxn #06	FTL Plate 19	B1	50	0.543	0.0458	0.513	-6.4
Rxn #06	FTL Plate 19	C1	50	0.523	0.0458	0.513	-2.1
Rxn #06	FTL Plate 19	D1	50	0.451	0.0458	0.513	13.3
Rxn #06	FTL Plate 19	E1	50	0.445	0.0458	0.513	14.6
Rxn #06	FTL Plate 19	F1	50	0.449	0.0458	0.513	13.7
Rxn #06	FTL Plate 19	G1	50	0.423	0.0458	0.513	19.3
Rxn #06	FTL Plate 19	H1: FOC	-	0.495	0.0458	0.513	3.9
Rxn #06	FTL Plate 19	A2: NFC	-	0.054	0.0458	0.513	98.2
Rxn #06	FTL Plate 19	B2	50	0.492	0.0458	0.513	4.5

S1 Table. Data from Screening of Botanical Extract Library for Inhibition of KHKC Activity.

Rxn ID	Sample			Sample	Ave. No Fructose Controls	Ave. Fructose Only Controls	KHKC
	Botanical Plate	Well	Conc (μg/mL)	(ΔOD_{340nm})	(ΔOD_{340nm})	(ΔOD_{340nm})	Inhibition (%)
Rxn #06	FTL Plate 19	C2	50	0.492	0.0458	0.513	4.5
Rxn #06	FTL Plate 19	D2	50	0.524	0.0458	0.513	-2.4
Rxn #06	FTL Plate 19	E2	50	0.531	0.0458	0.513	-3.9
Rxn #06	FTL Plate 19	F2	50	0.485	0.0458	0.513	6.0
Rxn #06	FTL Plate 19	G2	50	0.483	0.0458	0.513	6.4
Rxn #06	FTL Plate 19	H2: FOC	-	0.520	0.0458	0.513	-1.5
Rxn #06	FTL Plate 19	A3: NFC	-	0.044	0.0458	0.513	100.4
Rxn #06	FTL Plate 19	B3	50	0.530	0.0458	0.513	-3.6
Rxn #06	FTL Plate 19	C3	50	0.537	0.0458	0.513	-5.1
Rxn #06	FTL Plate 19	D3	50	0.501	0.0458	0.513	2.6
Rxn #06	FTL Plate 19	E3	50	0.532	0.0458	0.513	-4.1
Rxn #06	FTL Plate 19	F3	50	0.496	0.0458	0.513	3.6
Rxn #06	FTL Plate 19	G3	50	0.491	0.0458	0.513	4.7
Rxn #06	FTL Plate 19	H3: FOC	-	0.509	0.0458	0.513	0.9
Rxn #06	FTL Plate 19	A4: NFC	-	0.045	0.0458	0.513	100.2
Rxn #06	FTL Plate 19	B4	50	0.499	0.0458	0.513	3.0
Rxn #06	FTL Plate 19	C4	50	0.488	0.0458	0.513	5.4
Rxn #06	FTL Plate 19	D4	50	0.486	0.0458	0.513	5.8
Rxn #06	FTL Plate 19	E4	50	0.486	0.0458	0.513	5.8
Rxn #06	FTL Plate 19	F4	50	0.455	0.0458	0.513	12.4
Rxn #06	FTL Plate 19	G4	50	0.475	0.0458	0.513	8.1
Rxn #06	FTL Plate 19	H4: FOC	-	0.509	0.0458	0.513	0.9
Rxn #06	FTL Plate 19	A5: NFC	-	0.044	0.0458	0.513	100.4
Rxn #06	FTL Plate 19	B5: NFC	-	0.046	0.0458	0.513	100.0
Rxn #06	FTL Plate 19	C5: NFC	-	0.039	0.0458	0.513	101.5
Rxn #06	FTL Plate 19	D5: NFC	-	0.044	0.0458	0.513	100.4
Rxn #06	FTL Plate 19	E5: FOC	-	0.531	0.0458	0.513	-3.9
Rxn #06	FTL Plate 19	F5: FOC	-	0.524	0.0458	0.513	-2.4
Rxn #06	FTL Plate 19	G5: FOC	-	0.523	0.0458	0.513	-2.1
Rxn #06	FTL Plate 19	H5: FOC	-	0.517	0.0458	0.513	-0.9
Rxn #06	FTL Plate 19	A6: NFC	-	0.038	0.0458	0.513	101.7
Rxn #06	FTL Plate 19	B6	50	0.484	0.0458	0.513	6.2
Rxn #06	FTL Plate 19	C6	50	0.459	0.0458	0.513	11.6
Rxn #06	FTL Plate 19	D6	50	0.475	0.0458	0.513	8.1
Rxn #06	FTL Plate 19	E6	50	0.507	0.0458	0.513	1.3
Rxn #06	FTL Plate 19	F6	50	0.499	0.0458	0.513	3.0
Rxn #06	FTL Plate 19	G6	50	0.505	0.0458	0.513	1.7
Rxn #06	FTL Plate 19	H6: FOC	-	0.502	0.0458	0.513	2.4
Rxn #06	FTL Plate 19	A7: NFC	-	0.054	0.0458	0.513	98.2
Rxn #06	FTL Plate 19	B7	50	0.492	0.0458	0.513	4.5
Rxn #06	FTL Plate 19	C7	50	0.504	0.0458	0.513	1.9
Rxn #06	FTL Plate 19	D7	50	0.497	0.0458	0.513	3.4
Rxn #06	FTL Plate 19	E7	50	0.490	0.0458	0.513	4.9
Rxn #06	FTL Plate 19	F7	50	0.501	0.0458	0.513	2.6
Rxn #06	FTL Plate 19	G7	50	0.513	0.0458	0.513	0.0
Rxn #06	FTL Plate 19	H7: FOC	-	0.520	0.0458	0.513	-1.5
Rxn #06	FTL Plate 19	A8: NFC	-	0.052	0.0458	0.513	98.7
Rxn #06	FTL Plate 19	B8	50	0.505	0.0458	0.513	1.7
Rxn #06	FTL Plate 19	C8	50	0.528	0.0458	0.513	-3.2
Rxn #06	FTL Plate 19	D8	50	0.518	0.0458	0.513	-1.1
Rxn #06	FTL Plate 19	E8	50	0.511	0.0458	0.513	0.4
Rxn #06	FTL Plate 19	F8	50	0.499	0.0458	0.513	3.0
Rxn #06	FTL Plate 19	G8	50	0.462	0.0458	0.513	10.9
Rxn #06	FTL Plate 19	H8: FOC	-	0.513	0.0458	0.513	0.0
Rxn #06	FTL Plate 19	A9: NFC	-	0.046	0.0458	0.513	100.0
Rxn #06	FTL Plate 19	B9	50	0.478	0.0458	0.513	7.5
Rxn #06	FTL Plate 19	C9	50	0.458	0.0458	0.513	11.8
Rxn #06	FTL Plate 19	D9	50	0.435	0.0458	0.513	16.7
Rxn #06	FTL Plate 19	E9	50	0.446	0.0458	0.513	14.3
Rxn #06	FTL Plate 19	F9	50	0.469	0.0458	0.513	9.4
Rxn #06	FTL Plate 19	G9	50	0.467	0.0458	0.513	9.8
Rxn #06	FTL Plate 19	H9: FOC	-	0.518	0.0458	0.513	-1.1
Rxn #06	FTL Plate 19	A10: NFC	-	0.043	0.0458	0.513	100.6
Rxn #06	FTL Plate 19	B10	50	0.465	0.0458	0.513	10.3
Rxn #06	FTL Plate 19	C10	50	0.489	0.0458	0.513	5.1
Rxn #06	FTL Plate 19	D10	50	0.524	0.0458	0.513	-2.4
Rxn #06	FTL Plate 19	E10	50	0.486	0.0458	0.513	5.8
Rxn #06	FTL Plate 19	F10	50	0.526	0.0458	0.513	-2.8
Rxn #06	FTL Plate 19	G10	50	0.487	0.0458	0.513	5.6
Rxn #06	FTL Plate 19	H10: FOC	-	0.513	0.0458	0.513	0.0

S1 Table. Data from Screening of Botanical Extract Library for Inhibition of KHKC Activity.

Rxn ID	Sample			Sample	Ave. No Fructose Controls	Ave. Fructose Only Controls	KHKC
	Botanical Plate	Well	Conc (μg/mL)	(ΔOD_{340nm})	(ΔOD_{340nm})	(ΔOD_{340nm})	Inhibition (%)
Rxn #06	FTL Plate 19	A11: NFC	-	0.047	0.0458	0.513	99.7
Rxn #06	FTL Plate 19	B11	50	0.507	0.0458	0.513	1.3
Rxn #06	FTL Plate 19	C11	50	0.502	0.0458	0.513	2.4
Rxn #06	FTL Plate 19	D11	50	0.511	0.0458	0.513	0.4
Rxn #06	FTL Plate 19	E11	50	0.501	0.0458	0.513	2.6
Rxn #06	FTL Plate 19	F11	50	0.501	0.0458	0.513	2.6
Rxn #06	FTL Plate 19	G11	50	0.521	0.0458	0.513	-1.7
Rxn #06	FTL Plate 19	H11: FOC	-	0.514	0.0458	0.513	-0.2
Rxn #06	FTL Plate 19	A12: NFC	-	0.048	0.0458	0.513	99.5
Rxn #06	FTL Plate 19	H12: FOC	-	0.490	0.0458	0.513	4.9
Rxn #07	FTL Plate 1	A1: NFC	-	0.063	0.0597	0.532	99.3
Rxn #07	FTL Plate 1	B1	50	0.441	0.0597	0.532	19.3
Rxn #07	FTL Plate 1	C1	50	0.511	0.0597	0.532	4.4
Rxn #07	FTL Plate 1	D1	50	0.426	0.0597	0.532	22.4
Rxn #07	FTL Plate 1	E1	50	0.539	0.0597	0.532	-1.5
Rxn #07	FTL Plate 1	F1	50	0.479	0.0597	0.532	11.2
Rxn #07	FTL Plate 1	G1	50	0.473	0.0597	0.532	12.5
Rxn #07	FTL Plate 1	H1: FOC	-	0.534	0.0597	0.532	-0.4
Rxn #07	FTL Plate 1	A2: NFC	-	0.065	0.0597	0.532	98.9
Rxn #07	FTL Plate 1	B2	50	0.553	0.0597	0.532	-4.4
Rxn #07	FTL Plate 1	C2	50	0.452	0.0597	0.532	16.9
Rxn #07	FTL Plate 1	D2	50	0.525	0.0597	0.532	1.5
Rxn #07	FTL Plate 1	E2	50	0.490	0.0597	0.532	8.9
Rxn #07	FTL Plate 1	F2	50	0.415	0.0597	0.532	24.8
Rxn #07	FTL Plate 1	G2	50	0.519	0.0597	0.532	2.8
Rxn #07	FTL Plate 1	H2: FOC	-	0.500	0.0597	0.532	6.8
Rxn #07	FTL Plate 1	A3: NFC	-	0.065	0.0597	0.532	98.9
Rxn #07	FTL Plate 1	B3	50	0.521	0.0597	0.532	2.3
Rxn #07	FTL Plate 1	C3	50	0.557	0.0597	0.532	-5.3
Rxn #07	FTL Plate 1	D3	50	0.541	0.0597	0.532	-1.9
Rxn #07	FTL Plate 1	E3	50	0.385	0.0597	0.532	31.1
Rxn #07	FTL Plate 1	F3	50	0.523	0.0597	0.532	1.9
Rxn #07	FTL Plate 1	G3	50	0.487	0.0597	0.532	9.5
Rxn #07	FTL Plate 1	H3: FOC	-	0.548	0.0597	0.532	-3.4
Rxn #07	FTL Plate 1	A4: NFC	-	0.069	0.0597	0.532	98.0
Rxn #07	FTL Plate 1	B4	50	0.577	0.0597	0.532	-9.5
Rxn #07	FTL Plate 1	C4	50	0.578	0.0597	0.532	-9.7
Rxn #07	FTL Plate 1	D4	50	0.545	0.0597	0.532	-2.8
Rxn #07	FTL Plate 1	E4	50	0.499	0.0597	0.532	7.0
Rxn #07	FTL Plate 1	F4	50	0.567	0.0597	0.532	-7.4
Rxn #07	FTL Plate 1	G4	50	0.555	0.0597	0.532	-4.9
Rxn #07	FTL Plate 1	H4: FOC	-	0.562	0.0597	0.532	-6.4
Rxn #07	FTL Plate 1	A5: NFC	-	0.061	0.0597	0.532	99.7
Rxn #07	FTL Plate 1	B5: NFC	-	0.057	0.0597	0.532	100.6
Rxn #07	FTL Plate 1	C5: NFC	-	0.046	0.0597	0.532	102.9
Rxn #07	FTL Plate 1	D5: NFC	-	0.046	0.0597	0.532	102.9
Rxn #07	FTL Plate 1	E5: FOC	-	0.420	0.0597	0.532	23.7
Rxn #07	FTL Plate 1	F5: FOC	-	0.555	0.0597	0.532	-4.9
Rxn #07	FTL Plate 1	G5: FOC	-	0.553	0.0597	0.532	-4.4
Rxn #07	FTL Plate 1	H5: FOC	-	0.552	0.0597	0.532	-4.2
Rxn #07	FTL Plate 1	A6: NFC	-	0.059	0.0597	0.532	100.1
Rxn #07	FTL Plate 1	B6	50	0.545	0.0597	0.532	-2.8
Rxn #07	FTL Plate 1	C6	50	0.544	0.0597	0.532	-2.5
Rxn #07	FTL Plate 1	D6	50	0.534	0.0597	0.532	-0.4
Rxn #07	FTL Plate 1	E6	50	0.496	0.0597	0.532	7.6
Rxn #07	FTL Plate 1	F6	50	0.434	0.0597	0.532	20.7
Rxn #07	FTL Plate 1	G6	50	0.488	0.0597	0.532	9.3
Rxn #07	FTL Plate 1	H6: FOC	-	0.533	0.0597	0.532	-0.2
Rxn #07	FTL Plate 1	A7: NFC	-	0.053	0.0597	0.532	101.4
Rxn #07	FTL Plate 1	B7	50	0.553	0.0597	0.532	-4.4
Rxn #07	FTL Plate 1	C7	50	0.544	0.0597	0.532	-2.5
Rxn #07	FTL Plate 1	D7	50	0.572	0.0597	0.532	-8.5
Rxn #07	FTL Plate 1	E7	50	0.384	0.0597	0.532	31.3
Rxn #07	FTL Plate 1	F7	50	0.554	0.0597	0.532	-4.7
Rxn #07	FTL Plate 1	G7	50	0.554	0.0597	0.532	-4.7
Rxn #07	FTL Plate 1	H7: FOC	-	0.560	0.0597	0.532	-5.9
Rxn #07	FTL Plate 1	A8: NFC	-	0.074	0.0597	0.532	97.0
Rxn #07	FTL Plate 1	B8	50	0.558	0.0597	0.532	-5.5
Rxn #07	FTL Plate 1	C8	50	0.561	0.0597	0.532	-6.1
Rxn #07	FTL Plate 1	D8	50	0.560	0.0597	0.532	-5.9

S1 Table. Data from Screening of Botanical Extract Library for Inhibition of KHKC Activity.

Rxn ID	Sample			Sample	Ave. No Fructose Controls	Ave. Fructose Only Controls	KHKC
	Botanical Plate	Well	Conc (μg/mL)	(ΔOD_{340nm})	(ΔOD_{340nm})	(ΔOD_{340nm})	Inhibition (%)
Rxn #07	FTL Plate 1	E8	50	0.505	0.0597	0.532	5.7
Rxn #07	FTL Plate 1	F8	50	0.583	0.0597	0.532	-10.8
Rxn #07	FTL Plate 1	G8	50	0.495	0.0597	0.532	7.8
Rxn #07	FTL Plate 1	H8: FOC	-	0.576	0.0597	0.532	-9.3
Rxn #07	FTL Plate 1	A9: NFC	-	0.059	0.0597	0.532	100.1
Rxn #07	FTL Plate 1	B9	50	0.539	0.0597	0.532	-1.5
Rxn #07	FTL Plate 1	C9	50	0.507	0.0597	0.532	5.3
Rxn #07	FTL Plate 1	D9	50	0.528	0.0597	0.532	0.8
Rxn #07	FTL Plate 1	E9	50	0.515	0.0597	0.532	3.6
Rxn #07	FTL Plate 1	F9	50	0.499	0.0597	0.532	7.0
Rxn #07	FTL Plate 1	G9	50	0.531	0.0597	0.532	0.2
Rxn #07	FTL Plate 1	H9: FOC	-	0.497	0.0597	0.532	7.4
Rxn #07	FTL Plate 1	A10: NFC	-	0.056	0.0597	0.532	100.8
Rxn #07	FTL Plate 1	B10	50	0.540	0.0597	0.532	-1.7
Rxn #07	FTL Plate 1	C10	50	0.548	0.0597	0.532	-3.4
Rxn #07	FTL Plate 1	D10	50	0.561	0.0597	0.532	-6.1
Rxn #07	FTL Plate 1	E10	50	0.539	0.0597	0.532	-1.5
Rxn #07	FTL Plate 1	F10	50	0.543	0.0597	0.532	-2.3
Rxn #07	FTL Plate 1	G10	50	0.510	0.0597	0.532	4.7
Rxn #07	FTL Plate 1	H10: FOC	-	0.517	0.0597	0.532	3.2
Rxn #07	FTL Plate 1	A11: NFC	-	0.058	0.0597	0.532	100.4
Rxn #07	FTL Plate 1	B11	50	0.527	0.0597	0.532	1.1
Rxn #07	FTL Plate 1	C11	50	0.555	0.0597	0.532	-4.9
Rxn #07	FTL Plate 1	D11	50	0.575	0.0597	0.532	-9.1
Rxn #07	FTL Plate 1	E11	50	0.571	0.0597	0.532	-8.3
Rxn #07	FTL Plate 1	F11	50	0.545	0.0597	0.532	-2.8
Rxn #07	FTL Plate 1	G11	50	0.533	0.0597	0.532	-0.2
Rxn #07	FTL Plate 1	H11: FOC	-	0.539	0.0597	0.532	-1.5
Rxn #07	FTL Plate 1	A12: NFC	-	0.064	0.0597	0.532	99.1
Rxn #07	FTL Plate 1	H12: FOC	-	0.527	0.0597	0.532	1.1
Rxn #08	FTL Plate 13	A1: NFC	-	0.056	0.0487	0.505	98.4
Rxn #08	FTL Plate 13	B1	50	0.495	0.0487	0.505	2.2
Rxn #08	FTL Plate 13	C1	50	0.477	0.0487	0.505	6.1
Rxn #08	FTL Plate 13	D1	50	0.495	0.0487	0.505	2.2
Rxn #08	FTL Plate 13	E1	50	0.479	0.0487	0.505	5.7
Rxn #08	FTL Plate 13	F1	50	0.480	0.0487	0.505	5.5
Rxn #08	FTL Plate 13	G1	50	0.471	0.0487	0.505	7.5
Rxn #08	FTL Plate 13	H1: FOC	-	0.493	0.0487	0.505	2.6
Rxn #08	FTL Plate 13	A2: NFC	-	0.062	0.0487	0.505	97.1
Rxn #08	FTL Plate 13	B2	50	0.507	0.0487	0.505	-0.4
Rxn #08	FTL Plate 13	C2	50	0.526	0.0487	0.505	-4.6
Rxn #08	FTL Plate 13	D2	50	0.500	0.0487	0.505	1.1
Rxn #08	FTL Plate 13	E2	50	0.516	0.0487	0.505	-2.4
Rxn #08	FTL Plate 13	F2	50	0.507	0.0487	0.505	-0.4
Rxn #08	FTL Plate 13	G2	50	0.498	0.0487	0.505	1.5
Rxn #08	FTL Plate 13	H2: FOC	-	0.512	0.0487	0.505	-1.5
Rxn #08	FTL Plate 13	A3: NFC	-	0.044	0.0487	0.505	101.0
Rxn #08	FTL Plate 13	B3	50	0.509	0.0487	0.505	-0.9
Rxn #08	FTL Plate 13	C3	50	0.528	0.0487	0.505	-5.0
Rxn #08	FTL Plate 13	D3	50	0.516	0.0487	0.505	-2.4
Rxn #08	FTL Plate 13	E3	50	0.488	0.0487	0.505	3.7
Rxn #08	FTL Plate 13	F3	50	0.498	0.0487	0.505	1.5
Rxn #08	FTL Plate 13	G3	50	0.501	0.0487	0.505	0.9
Rxn #08	FTL Plate 13	H3: FOC	-	0.506	0.0487	0.505	-0.2
Rxn #08	FTL Plate 13	A4: NFC	-	0.047	0.0487	0.505	100.4
Rxn #08	FTL Plate 13	B4	50	0.505	0.0487	0.505	0.0
Rxn #08	FTL Plate 13	C4	50	0.501	0.0487	0.505	0.9
Rxn #08	FTL Plate 13	D4	50	0.513	0.0487	0.505	-1.8
Rxn #08	FTL Plate 13	E4	50	0.460	0.0487	0.505	9.9
Rxn #08	FTL Plate 13	F4	50	0.490	0.0487	0.505	3.3
Rxn #08	FTL Plate 13	G4	50	0.463	0.0487	0.505	9.2
Rxn #08	FTL Plate 13	H4: FOC	-	0.509	0.0487	0.505	-0.9
Rxn #08	FTL Plate 13	A5: NFC	-	0.053	0.0487	0.505	99.1
Rxn #08	FTL Plate 13	B5: NFC	-	0.041	0.0487	0.505	101.7
Rxn #08	FTL Plate 13	C5: NFC	-	0.038	0.0487	0.505	102.3
Rxn #08	FTL Plate 13	D5: NFC	-	0.036	0.0487	0.505	102.8
Rxn #08	FTL Plate 13	E5: FOC	-	0.507	0.0487	0.505	-0.4
Rxn #08	FTL Plate 13	F5: FOC	-	0.496	0.0487	0.505	2.0
Rxn #08	FTL Plate 13	G5: FOC	-	0.500	0.0487	0.505	1.1
Rxn #08	FTL Plate 13	H5: FOC	-	0.508	0.0487	0.505	-0.7

S1 Table. Data from Screening of Botanical Extract Library for Inhibition of KHKC Activity.

Rxn ID	Sample			Sample	Ave. No Fructose Controls	Ave. Fructose Only Controls	KHKC
	Botanical Plate	Well	Conc (μg/mL)	(ΔOD_{340nm})	(ΔOD_{340nm})	(ΔOD_{340nm})	Inhibition (%)
Rxn #08	FTL Plate 13	A6: NFC	-	0.046	0.0487	0.505	100.6
Rxn #08	FTL Plate 13	B6	50	0.524	0.0487	0.505	-4.2
Rxn #08	FTL Plate 13	C6	50	0.512	0.0487	0.505	-1.5
Rxn #08	FTL Plate 13	D6	50	0.500	0.0487	0.505	1.1
Rxn #08	FTL Plate 13	E6	50	0.505	0.0487	0.505	0.0
Rxn #08	FTL Plate 13	F6	50	0.481	0.0487	0.505	5.3
Rxn #08	FTL Plate 13	G6	50	0.516	0.0487	0.505	-2.4
Rxn #08	FTL Plate 13	H6: FOC	-	0.505	0.0487	0.505	0.0
Rxn #08	FTL Plate 13	A7: NFC	-	0.045	0.0487	0.505	100.8
Rxn #08	FTL Plate 13	B7	50	0.531	0.0487	0.505	-5.7
Rxn #08	FTL Plate 13	C7	50	0.512	0.0487	0.505	-1.5
Rxn #08	FTL Plate 13	D7	50	0.522	0.0487	0.505	-3.7
Rxn #08	FTL Plate 13	E7	50	0.528	0.0487	0.505	-5.0
Rxn #08	FTL Plate 13	F7	50	0.519	0.0487	0.505	-3.1
Rxn #08	FTL Plate 13	G7	50	0.510	0.0487	0.505	-1.1
Rxn #08	FTL Plate 13	H7: FOC	-	0.502	0.0487	0.505	0.7
Rxn #08	FTL Plate 13	A8: NFC	-	0.048	0.0487	0.505	100.2
Rxn #08	FTL Plate 13	B8	50	0.497	0.0487	0.505	1.8
Rxn #08	FTL Plate 13	C8	50	0.509	0.0487	0.505	-0.9
Rxn #08	FTL Plate 13	D8	50	0.510	0.0487	0.505	-1.1
Rxn #08	FTL Plate 13	E8	50	0.508	0.0487	0.505	-0.7
Rxn #08	FTL Plate 13	F8	50	0.520	0.0487	0.505	-3.3
Rxn #08	FTL Plate 13	G8	50	0.520	0.0487	0.505	-3.3
Rxn #08	FTL Plate 13	H8: FOC	-	0.502	0.0487	0.505	0.7
Rxn #08	FTL Plate 13	A9: NFC	-	0.059	0.0487	0.505	97.7
Rxn #08	FTL Plate 13	B9	50	0.518	0.0487	0.505	-2.8
Rxn #08	FTL Plate 13	C9	50	0.495	0.0487	0.505	2.2
Rxn #08	FTL Plate 13	D9	50	0.473	0.0487	0.505	7.0
Rxn #08	FTL Plate 13	E9	50	0.510	0.0487	0.505	-1.1
Rxn #08	FTL Plate 13	F9	50	0.462	0.0487	0.505	9.4
Rxn #08	FTL Plate 13	G9	50	0.512	0.0487	0.505	-1.5
Rxn #08	FTL Plate 13	H9: FOC	-	0.515	0.0487	0.505	-2.2
Rxn #08	FTL Plate 13	A10: NFC	-	0.051	0.0487	0.505	99.5
Rxn #08	FTL Plate 13	B10	50	0.505	0.0487	0.505	0.0
Rxn #08	FTL Plate 13	C10	50	0.499	0.0487	0.505	1.3
Rxn #08	FTL Plate 13	D10	50	0.490	0.0487	0.505	3.3
Rxn #08	FTL Plate 13	E10	50	0.471	0.0487	0.505	7.5
Rxn #08	FTL Plate 13	F10	50	0.508	0.0487	0.505	-0.7
Rxn #08	FTL Plate 13	G10	50	0.481	0.0487	0.505	5.3
Rxn #08	FTL Plate 13	H10: FOC	-	0.507	0.0487	0.505	-0.4
Rxn #08	FTL Plate 13	A11: NFC	-	0.048	0.0487	0.505	100.2
Rxn #08	FTL Plate 13	B11	50	0.503	0.0487	0.505	0.4
Rxn #08	FTL Plate 13	C11	50	0.509	0.0487	0.505	-0.9
Rxn #08	FTL Plate 13	D11	50	0.497	0.0487	0.505	1.8
Rxn #08	FTL Plate 13	E11	50	0.501	0.0487	0.505	0.9
Rxn #08	FTL Plate 13	F11	50	0.491	0.0487	0.505	3.1
Rxn #08	FTL Plate 13	G11	50	0.477	0.0487	0.505	6.1
Rxn #08	FTL Plate 13	H11: FOC	-	0.502	0.0487	0.505	0.7
Rxn #08	FTL Plate 13	A12: NFC	-	0.056	0.0487	0.505	98.4
Rxn #08	FTL Plate 13	H12: FOC	-	0.506	0.0487	0.505	-0.2
Rxn #09	FTL Plate 14	A1: NFC	-	0.045	0.0423	0.479	99.4
Rxn #09	FTL Plate 14	B1	50	0.493	0.0423	0.479	-3.2
Rxn #09	FTL Plate 14	C1	50	0.478	0.0423	0.479	0.2
Rxn #09	FTL Plate 14	D1	50	0.500	0.0423	0.479	-4.8
Rxn #09	FTL Plate 14	E1	50	0.495	0.0423	0.479	-3.7
Rxn #09	FTL Plate 14	F1	50	0.473	0.0423	0.479	1.4
Rxn #09	FTL Plate 14	G1	50	0.498	0.0423	0.479	-4.4
Rxn #09	FTL Plate 14	H1: FOC	-	0.437	0.0423	0.479	9.6
Rxn #09	FTL Plate 14	A2: NFC	-	0.039	0.0423	0.479	100.8
Rxn #09	FTL Plate 14	B2	50	0.550	0.0423	0.479	-16.3
Rxn #09	FTL Plate 14	C2	50	0.535	0.0423	0.479	-12.8
Rxn #09	FTL Plate 14	D2	50	0.500	0.0423	0.479	-4.8
Rxn #09	FTL Plate 14	E2	50	0.519	0.0423	0.479	-9.2
Rxn #09	FTL Plate 14	F2	50	0.513	0.0423	0.479	-7.8
Rxn #09	FTL Plate 14	G2	50	0.491	0.0423	0.479	-2.7
Rxn #09	FTL Plate 14	H2: FOC	-	0.462	0.0423	0.479	3.9
Rxn #09	FTL Plate 14	A3: NFC	-	0.039	0.0423	0.479	100.8
Rxn #09	FTL Plate 14	B3	50	0.504	0.0423	0.479	-5.7
Rxn #09	FTL Plate 14	C3	50	0.520	0.0423	0.479	-9.4
Rxn #09	FTL Plate 14	D3	50	0.530	0.0423	0.479	-11.7

S1 Table. Data from Screening of Botanical Extract Library for Inhibition of KHKC Activity.

Rxn ID	Sample			Sample	Ave. No Fructose Controls	Ave. Fructose Only Controls	KHKC
	Botanical Plate	Well	Conc (μg/mL)	(ΔOD_{340nm})	(ΔOD_{340nm})	(ΔOD_{340nm})	Inhibition (%)
Rxn #09	FTL Plate 14	E3	50	0.501	0.0423	0.479	-5.0
Rxn #09	FTL Plate 14	F3	50	0.512	0.0423	0.479	-7.6
Rxn #09	FTL Plate 14	G3	50	0.518	0.0423	0.479	-8.9
Rxn #09	FTL Plate 14	H3: FOC	-	0.474	0.0423	0.479	1.1
Rxn #09	FTL Plate 14	A4: NFC	-	0.041	0.0423	0.479	100.3
Rxn #09	FTL Plate 14	B4	50	0.534	0.0423	0.479	-12.6
Rxn #09	FTL Plate 14	C4	50	0.526	0.0423	0.479	-10.8
Rxn #09	FTL Plate 14	D4	50	0.512	0.0423	0.479	-7.6
Rxn #09	FTL Plate 14	E4	50	0.501	0.0423	0.479	-5.0
Rxn #09	FTL Plate 14	F4	50	0.515	0.0423	0.479	-8.2
Rxn #09	FTL Plate 14	G4	50	0.496	0.0423	0.479	-3.9
Rxn #09	FTL Plate 14	H4: FOC	-	0.459	0.0423	0.479	4.6
Rxn #09	FTL Plate 14	A5: NFC	-	0.046	0.0423	0.479	99.2
Rxn #09	FTL Plate 14	B5: NFC	-	0.037	0.0423	0.479	101.2
Rxn #09	FTL Plate 14	C5: NFC	-	0.039	0.0423	0.479	100.8
Rxn #09	FTL Plate 14	D5: NFC	-	0.037	0.0423	0.479	101.2
Rxn #09	FTL Plate 14	E5: FOC	-	0.526	0.0423	0.479	-10.8
Rxn #09	FTL Plate 14	F5: FOC	-	0.513	0.0423	0.479	-7.8
Rxn #09	FTL Plate 14	G5: FOC	-	0.512	0.0423	0.479	-7.6
Rxn #09	FTL Plate 14	H5: FOC	-	0.467	0.0423	0.479	2.7
Rxn #09	FTL Plate 14	A6: NFC	-	0.040	0.0423	0.479	100.5
Rxn #09	FTL Plate 14	B6	50	0.531	0.0423	0.479	-11.9
Rxn #09	FTL Plate 14	C6	50	0.504	0.0423	0.479	-5.7
Rxn #09	FTL Plate 14	D6	50	0.525	0.0423	0.479	-10.5
Rxn #09	FTL Plate 14	E6	50	0.517	0.0423	0.479	-8.7
Rxn #09	FTL Plate 14	F6	50	0.505	0.0423	0.479	-6.0
Rxn #09	FTL Plate 14	G6	50	0.526	0.0423	0.479	-10.8
Rxn #09	FTL Plate 14	H6: FOC	-	0.472	0.0423	0.479	1.6
Rxn #09	FTL Plate 14	A7: NFC	-	0.041	0.0423	0.479	100.3
Rxn #09	FTL Plate 14	B7	50	0.530	0.0423	0.479	-11.7
Rxn #09	FTL Plate 14	C7	50	0.535	0.0423	0.479	-12.8
Rxn #09	FTL Plate 14	D7	50	0.487	0.0423	0.479	-1.8
Rxn #09	FTL Plate 14	E7	50	0.489	0.0423	0.479	-2.3
Rxn #09	FTL Plate 14	F7	50	0.528	0.0423	0.479	-11.2
Rxn #09	FTL Plate 14	G7	50	0.489	0.0423	0.479	-2.3
Rxn #09	FTL Plate 14	H7: FOC	-	0.484	0.0423	0.479	-1.1
Rxn #09	FTL Plate 14	A8: NFC	-	0.038	0.0423	0.479	101.0
Rxn #09	FTL Plate 14	B8	50	0.517	0.0423	0.479	-8.7
Rxn #09	FTL Plate 14	C8	50	0.527	0.0423	0.479	-11.0
Rxn #09	FTL Plate 14	D8	50	0.528	0.0423	0.479	-11.2
Rxn #09	FTL Plate 14	E8	50	0.509	0.0423	0.479	-6.9
Rxn #09	FTL Plate 14	F8	50	0.524	0.0423	0.479	-10.3
Rxn #09	FTL Plate 14	G8	50	0.507	0.0423	0.479	-6.4
Rxn #09	FTL Plate 14	H8: FOC	-	0.461	0.0423	0.479	4.1
Rxn #09	FTL Plate 14	A9: NFC	-	0.046	0.0423	0.479	99.2
Rxn #09	FTL Plate 14	B9	50	0.543	0.0423	0.479	-14.7
Rxn #09	FTL Plate 14	C9	50	0.507	0.0423	0.479	-6.4
Rxn #09	FTL Plate 14	D9	50	0.538	0.0423	0.479	-13.5
Rxn #09	FTL Plate 14	E9	50	0.542	0.0423	0.479	-14.4
Rxn #09	FTL Plate 14	F9	50	0.512	0.0423	0.479	-7.6
Rxn #09	FTL Plate 14	G9	50	0.521	0.0423	0.479	-9.6
Rxn #09	FTL Plate 14	H9: FOC	-	0.496	0.0423	0.479	-3.9
Rxn #09	FTL Plate 14	A10: NFC	-	0.047	0.0423	0.479	98.9
Rxn #09	FTL Plate 14	B10	50	0.532	0.0423	0.479	-12.1
Rxn #09	FTL Plate 14	C10	50	0.544	0.0423	0.479	-14.9
Rxn #09	FTL Plate 14	D10	50	0.502	0.0423	0.479	-5.3
Rxn #09	FTL Plate 14	E10	50	0.507	0.0423	0.479	-6.4
Rxn #09	FTL Plate 14	F10	50	0.515	0.0423	0.479	-8.2
Rxn #09	FTL Plate 14	G10	50	0.507	0.0423	0.479	-6.4
Rxn #09	FTL Plate 14	H10: FOC	-	0.473	0.0423	0.479	1.4
Rxn #09	FTL Plate 14	A11: NFC	-	0.049	0.0423	0.479	98.5
Rxn #09	FTL Plate 14	B11	50	0.513	0.0423	0.479	-7.8
Rxn #09	FTL Plate 14	C11	50	0.531	0.0423	0.479	-11.9
Rxn #09	FTL Plate 14	D11	50	0.539	0.0423	0.479	-13.7
Rxn #09	FTL Plate 14	E11	50	0.493	0.0423	0.479	-3.2
Rxn #09	FTL Plate 14	F11	50	0.523	0.0423	0.479	-10.1
Rxn #09	FTL Plate 14	G11	50	0.529	0.0423	0.479	-11.4
Rxn #09	FTL Plate 14	H11: FOC	-	0.467	0.0423	0.479	2.7
Rxn #09	FTL Plate 14	A12: NFC	-	0.050	0.0423	0.479	98.2
Rxn #09	FTL Plate 14	H12: FOC	-	0.480	0.0423	0.479	-0.2

S1 Table. Data from Screening of Botanical Extract Library for Inhibition of KHKC Activity.

Rxn ID	Sample			Sample	Ave. No Fructose Controls	Ave. Fructose Only Controls	KHKC
	Botanical Plate	Well	Conc (μg/mL)	(ΔOD_{340nm})	(ΔOD_{340nm})	(ΔOD_{340nm})	Inhibition (%)
Rxn #10	FTL Plate 15	A1: NFC	-	0.043	0.0350	0.469	98.2
Rxn #10	FTL Plate 15	B1	50	0.454	0.0350	0.469	3.5
Rxn #10	FTL Plate 15	C1	50	0.448	0.0350	0.469	4.8
Rxn #10	FTL Plate 15	D1	50	0.473	0.0350	0.469	-0.9
Rxn #10	FTL Plate 15	E1	50	0.495	0.0350	0.469	-6.0
Rxn #10	FTL Plate 15	F1	50	0.466	0.0350	0.469	0.7
Rxn #10	FTL Plate 15	G1	50	0.509	0.0350	0.469	-9.2
Rxn #10	FTL Plate 15	H1: FOC	-	0.473	0.0350	0.469	-0.9
Rxn #10	FTL Plate 15	A2: NFC	-	0.034	0.0350	0.469	100.2
Rxn #10	FTL Plate 15	B2	50	0.477	0.0350	0.469	-1.8
Rxn #10	FTL Plate 15	C2	50	0.463	0.0350	0.469	1.4
Rxn #10	FTL Plate 15	D2	50	0.436	0.0350	0.469	7.6
Rxn #10	FTL Plate 15	E2	50	0.424	0.0350	0.469	10.4
Rxn #10	FTL Plate 15	F2	50	0.472	0.0350	0.469	-0.7
Rxn #10	FTL Plate 15	G2	50	0.458	0.0350	0.469	2.5
Rxn #10	FTL Plate 15	H2: FOC	-	0.471	0.0350	0.469	-0.5
Rxn #10	FTL Plate 15	A3: NFC	-	0.039	0.0350	0.469	99.1
Rxn #10	FTL Plate 15	B3	50	0.449	0.0350	0.469	4.6
Rxn #10	FTL Plate 15	C3	50	0.435	0.0350	0.469	7.8
Rxn #10	FTL Plate 15	D3	50	0.487	0.0350	0.469	-4.1
Rxn #10	FTL Plate 15	E3	50	0.456	0.0350	0.469	3.0
Rxn #10	FTL Plate 15	F3	50	0.484	0.0350	0.469	-3.5
Rxn #10	FTL Plate 15	G3	50	0.421	0.0350	0.469	11.1
Rxn #10	FTL Plate 15	H3: FOC	-	0.474	0.0350	0.469	-1.2
Rxn #10	FTL Plate 15	A4: NFC	-	0.034	0.0350	0.469	100.2
Rxn #10	FTL Plate 15	B4	50	0.463	0.0350	0.469	1.4
Rxn #10	FTL Plate 15	C4	50	0.416	0.0350	0.469	12.2
Rxn #10	FTL Plate 15	D4	50	0.454	0.0350	0.469	3.5
Rxn #10	FTL Plate 15	E4	50	0.459	0.0350	0.469	2.3
Rxn #10	FTL Plate 15	F4	50	0.452	0.0350	0.469	3.9
Rxn #10	FTL Plate 15	G4	50	0.406	0.0350	0.469	14.5
Rxn #10	FTL Plate 15	H4: FOC	-	0.509	0.0350	0.469	-9.2
Rxn #10	FTL Plate 15	A5: NFC	-	0.031	0.0350	0.469	100.9
Rxn #10	FTL Plate 15	B5: NFC	-	0.034	0.0350	0.469	100.2
Rxn #10	FTL Plate 15	C5: NFC	-	0.030	0.0350	0.469	101.2
Rxn #10	FTL Plate 15	D5: NFC	-	0.035	0.0350	0.469	100.0
Rxn #10	FTL Plate 15	E5: FOC	-	0.467	0.0350	0.469	0.5
Rxn #10	FTL Plate 15	F5: FOC	-	0.470	0.0350	0.469	-0.2
Rxn #10	FTL Plate 15	G5: FOC	-	0.469	0.0350	0.469	0.0
Rxn #10	FTL Plate 15	H5: FOC	-	0.462	0.0350	0.469	1.6
Rxn #10	FTL Plate 15	A6: NFC	-	0.035	0.0350	0.469	100.0
Rxn #10	FTL Plate 15	B6	50	0.494	0.0350	0.469	-5.8
Rxn #10	FTL Plate 15	C6	50	0.463	0.0350	0.469	1.4
Rxn #10	FTL Plate 15	D6	50	0.458	0.0350	0.469	2.5
Rxn #10	FTL Plate 15	E6	50	0.413	0.0350	0.469	12.9
Rxn #10	FTL Plate 15	F6	50	0.458	0.0350	0.469	2.5
Rxn #10	FTL Plate 15	G6	50	0.462	0.0350	0.469	1.6
Rxn #10	FTL Plate 15	H6: FOC	-	0.450	0.0350	0.469	4.4
Rxn #10	FTL Plate 15	A7: NFC	-	0.034	0.0350	0.469	100.2
Rxn #10	FTL Plate 15	B7	50	0.435	0.0350	0.469	7.8
Rxn #10	FTL Plate 15	C7	50	0.462	0.0350	0.469	1.6
Rxn #10	FTL Plate 15	D7	50	0.454	0.0350	0.469	3.5
Rxn #10	FTL Plate 15	E7	50	0.452	0.0350	0.469	3.9
Rxn #10	FTL Plate 15	F7	50	0.460	0.0350	0.469	2.1
Rxn #10	FTL Plate 15	G7	50	0.455	0.0350	0.469	3.2
Rxn #10	FTL Plate 15	H7: FOC	-	0.437	0.0350	0.469	7.4
Rxn #10	FTL Plate 15	A8: NFC	-	0.036	0.0350	0.469	99.8
Rxn #10	FTL Plate 15	B8	50	0.435	0.0350	0.469	7.8
Rxn #10	FTL Plate 15	C8	50	0.444	0.0350	0.469	5.8
Rxn #10	FTL Plate 15	D8	50	0.412	0.0350	0.469	13.1
Rxn #10	FTL Plate 15	E8	50	0.436	0.0350	0.469	7.6
Rxn #10	FTL Plate 15	F8	50	0.379	0.0350	0.469	20.7
Rxn #10	FTL Plate 15	G8	50	0.418	0.0350	0.469	11.8
Rxn #10	FTL Plate 15	H8: FOC	-	0.445	0.0350	0.469	5.5
Rxn #10	FTL Plate 15	A9: NFC	-	0.031	0.0350	0.469	100.9
Rxn #10	FTL Plate 15	B9	50	0.164	0.0350	0.469	70.3
Rxn #10	FTL Plate 15	C9	50	0.459	0.0350	0.469	2.3
Rxn #10	FTL Plate 15	D9	50	0.476	0.0350	0.469	-1.6
Rxn #10	FTL Plate 15	E9	50	0.426	0.0350	0.469	9.9
Rxn #10	FTL Plate 15	F9	50	0.452	0.0350	0.469	3.9

S1 Table. Data from Screening of Botanical Extract Library for Inhibition of KHKC Activity.

Rxn ID	Sample			Sample	Ave. No Fructose Controls	Ave. Fructose Only Controls	KHKC
	Botanical Plate	Well	Conc (µg/mL)	(ΔOD_{340nm})	(ΔOD_{340nm})	(ΔOD_{340nm})	Inhibition (%)
Rxn #10	FTL Plate 15	G9	50	0.469	0.0350	0.469	0.0
Rxn #10	FTL Plate 15	H9: FOC	-	0.476	0.0350	0.469	-1.6
Rxn #10	FTL Plate 15	A10: NFC	-	0.037	0.0350	0.469	99.5
Rxn #10	FTL Plate 15	B10	50	0.478	0.0350	0.469	-2.1
Rxn #10	FTL Plate 15	C10	50	0.452	0.0350	0.469	3.9
Rxn #10	FTL Plate 15	D10	50	0.451	0.0350	0.469	4.1
Rxn #10	FTL Plate 15	E10	50	0.458	0.0350	0.469	2.5
Rxn #10	FTL Plate 15	F10	50	0.485	0.0350	0.469	-3.7
Rxn #10	FTL Plate 15	G10	50	0.469	0.0350	0.469	0.0
Rxn #10	FTL Plate 15	H10: FOC	-	0.471	0.0350	0.469	-0.5
Rxn #10	FTL Plate 15	A11: NFC	-	0.034	0.0350	0.469	100.2
Rxn #10	FTL Plate 15	B11	50	0.455	0.0350	0.469	3.2
Rxn #10	FTL Plate 15	C11	50	0.459	0.0350	0.469	2.3
Rxn #10	FTL Plate 15	D11	50	0.505	0.0350	0.469	-8.3
Rxn #10	FTL Plate 15	E11	50	0.509	0.0350	0.469	-9.2
Rxn #10	FTL Plate 15	F11	50	0.446	0.0350	0.469	5.3
Rxn #10	FTL Plate 15	G11	50	0.464	0.0350	0.469	1.2
Rxn #10	FTL Plate 15	H11: FOC	-	0.472	0.0350	0.469	-0.7
Rxn #10	FTL Plate 15	A12: NFC	-	0.038	0.0350	0.469	99.3
Rxn #10	FTL Plate 15	H12: FOC	-	0.492	0.0350	0.469	-5.3
Rxn #11	FTL Plate 2	A1: NFC	-	0.057	0.0521	0.492	98.9
Rxn #11	FTL Plate 2	B1	50	0.486	0.0521	0.492	1.4
Rxn #11	FTL Plate 2	C1	50	0.452	0.0521	0.492	9.1
Rxn #11	FTL Plate 2	D1	50	0.476	0.0521	0.492	3.6
Rxn #11	FTL Plate 2	E1	50	0.459	0.0521	0.492	7.5
Rxn #11	FTL Plate 2	F1	50	0.428	0.0521	0.492	14.5
Rxn #11	FTL Plate 2	G1	50	0.356	0.0521	0.492	30.9
Rxn #11	FTL Plate 2	H1: FOC	-	0.465	0.0521	0.492	6.1
Rxn #11	FTL Plate 2	A2: NFC	-	0.062	0.0521	0.492	97.7
Rxn #11	FTL Plate 2	B2	50	0.478	0.0521	0.492	3.2
Rxn #11	FTL Plate 2	C2	50	0.509	0.0521	0.492	-3.9
Rxn #11	FTL Plate 2	D2	50	0.466	0.0521	0.492	5.9
Rxn #11	FTL Plate 2	E2	50	0.496	0.0521	0.492	-0.9
Rxn #11	FTL Plate 2	F2	50	0.422	0.0521	0.492	15.9
Rxn #11	FTL Plate 2	G2	50	0.479	0.0521	0.492	3.0
Rxn #11	FTL Plate 2	H2: FOC	-	0.494	0.0521	0.492	-0.5
Rxn #11	FTL Plate 2	A3: NFC	-	0.062	0.0521	0.492	97.7
Rxn #11	FTL Plate 2	B3	50	0.508	0.0521	0.492	-3.6
Rxn #11	FTL Plate 2	C3	50	0.500	0.0521	0.492	-1.8
Rxn #11	FTL Plate 2	D3	50	0.462	0.0521	0.492	6.8
Rxn #11	FTL Plate 2	E3	50	0.479	0.0521	0.492	3.0
Rxn #11	FTL Plate 2	F3	50	0.496	0.0521	0.492	-0.9
Rxn #11	FTL Plate 2	G3	50	0.504	0.0521	0.492	-2.7
Rxn #11	FTL Plate 2	H3: FOC	-	0.492	0.0521	0.492	0.0
Rxn #11	FTL Plate 2	A4: NFC	-	0.051	0.0521	0.492	100.3
Rxn #11	FTL Plate 2	B4	50	0.500	0.0521	0.492	-1.8
Rxn #11	FTL Plate 2	C4	50	0.522	0.0521	0.492	-6.8
Rxn #11	FTL Plate 2	D4	50	0.487	0.0521	0.492	1.1
Rxn #11	FTL Plate 2	E4	50	0.477	0.0521	0.492	3.4
Rxn #11	FTL Plate 2	F4	50	0.484	0.0521	0.492	1.8
Rxn #11	FTL Plate 2	G4	50	0.502	0.0521	0.492	-2.3
Rxn #11	FTL Plate 2	H4: FOC	-	0.484	0.0521	0.492	1.8
Rxn #11	FTL Plate 2	A5: NFC	-	0.054	0.0521	0.492	99.6
Rxn #11	FTL Plate 2	B5: NFC	-	0.047	0.0521	0.492	101.2
Rxn #11	FTL Plate 2	C5: NFC	-	0.039	0.0521	0.492	103.0
Rxn #11	FTL Plate 2	D5: NFC	-	0.037	0.0521	0.492	103.4
Rxn #11	FTL Plate 2	E5: FOC	-	0.510	0.0521	0.492	-4.1
Rxn #11	FTL Plate 2	F5: FOC	-	0.489	0.0521	0.492	0.7
Rxn #11	FTL Plate 2	G5: FOC	-	0.513	0.0521	0.492	-4.8
Rxn #11	FTL Plate 2	H5: FOC	-	0.498	0.0521	0.492	-1.4
Rxn #11	FTL Plate 2	A6: NFC	-	0.065	0.0521	0.492	97.1
Rxn #11	FTL Plate 2	B6	50	0.426	0.0521	0.492	15.0
Rxn #11	FTL Plate 2	C6	50	0.504	0.0521	0.492	-2.7
Rxn #11	FTL Plate 2	D6	50	0.525	0.0521	0.492	-7.5
Rxn #11	FTL Plate 2	E6	50	0.504	0.0521	0.492	-2.7
Rxn #11	FTL Plate 2	F6	50	0.497	0.0521	0.492	-1.1
Rxn #11	FTL Plate 2	G6	50	0.507	0.0521	0.492	-3.4
Rxn #11	FTL Plate 2	H6: FOC	-	0.512	0.0521	0.492	-4.5
Rxn #11	FTL Plate 2	A7: NFC	-	0.055	0.0521	0.492	99.3
Rxn #11	FTL Plate 2	B7	50	0.533	0.0521	0.492	-9.3

S1 Table. Data from Screening of Botanical Extract Library for Inhibition of KHKC Activity.

Rxn ID	Sample			Sample	Ave. No Fructose Controls	Ave. Fructose Only Controls	KHKC
	Botanical Plate	Well	Conc (μg/mL)	(ΔOD_{340nm})	(ΔOD_{340nm})	(ΔOD_{340nm})	Inhibition (%)
Rxn #11	FTL Plate 2	C7	50	0.513	0.0521	0.492	-4.8
Rxn #11	FTL Plate 2	D7	50	0.515	0.0521	0.492	-5.2
Rxn #11	FTL Plate 2	E7	50	0.517	0.0521	0.492	-5.7
Rxn #11	FTL Plate 2	F7	50	0.523	0.0521	0.492	-7.0
Rxn #11	FTL Plate 2	G7	50	0.483	0.0521	0.492	2.0
Rxn #11	FTL Plate 2	H7: FOC	-	0.494	0.0521	0.492	-0.5
Rxn #11	FTL Plate 2	A8: NFC	-	0.057	0.0521	0.492	98.9
Rxn #11	FTL Plate 2	B8	50	0.493	0.0521	0.492	-0.2
Rxn #11	FTL Plate 2	C8	50	0.474	0.0521	0.492	4.1
Rxn #11	FTL Plate 2	D8	50	0.514	0.0521	0.492	-5.0
Rxn #11	FTL Plate 2	E8	50	0.505	0.0521	0.492	-3.0
Rxn #11	FTL Plate 2	F8	50	0.481	0.0521	0.492	2.5
Rxn #11	FTL Plate 2	G8	50	0.479	0.0521	0.492	3.0
Rxn #11	FTL Plate 2	H8: FOC	-	0.493	0.0521	0.492	-0.2
Rxn #11	FTL Plate 2	A9: NFC	-	0.046	0.0521	0.492	101.4
Rxn #11	FTL Plate 2	B9	50	0.495	0.0521	0.492	-0.7
Rxn #11	FTL Plate 2	C9	50	0.503	0.0521	0.492	-2.5
Rxn #11	FTL Plate 2	D9	50	0.551	0.0521	0.492	-13.4
Rxn #11	FTL Plate 2	E9	50	0.506	0.0521	0.492	-3.2
Rxn #11	FTL Plate 2	F9	50	0.554	0.0521	0.492	-14.1
Rxn #11	FTL Plate 2	G9	50	0.381	0.0521	0.492	25.2
Rxn #11	FTL Plate 2	H9: FOC	-	0.488	0.0521	0.492	0.9
Rxn #11	FTL Plate 2	A10: NFC	-	0.052	0.0521	0.492	100.0
Rxn #11	FTL Plate 2	B10	50	0.512	0.0521	0.492	-4.5
Rxn #11	FTL Plate 2	C10	50	0.467	0.0521	0.492	5.7
Rxn #11	FTL Plate 2	D10	50	0.497	0.0521	0.492	-1.1
Rxn #11	FTL Plate 2	E10	50	0.499	0.0521	0.492	-1.6
Rxn #11	FTL Plate 2	F10	50	0.510	0.0521	0.492	-4.1
Rxn #11	FTL Plate 2	G10	50	0.506	0.0521	0.492	-3.2
Rxn #11	FTL Plate 2	H10: FOC	-	0.561	0.0521	0.492	-15.7
Rxn #11	FTL Plate 2	A11: NFC	-	0.040	0.0521	0.492	102.8
Rxn #11	FTL Plate 2	B11	50	0.505	0.0521	0.492	-3.0
Rxn #11	FTL Plate 2	C11	50	0.498	0.0521	0.492	-1.4
Rxn #11	FTL Plate 2	D11	50	0.490	0.0521	0.492	0.5
Rxn #11	FTL Plate 2	E11	50	0.506	0.0521	0.492	-3.2
Rxn #11	FTL Plate 2	F11	50	0.495	0.0521	0.492	-0.7
Rxn #11	FTL Plate 2	G11	50	0.510	0.0521	0.492	-4.1
Rxn #11	FTL Plate 2	H11: FOC	-	0.492	0.0521	0.492	0.0
Rxn #11	FTL Plate 2	A12: NFC	-	0.057	0.0521	0.492	98.9
Rxn #11	FTL Plate 2	H12: FOC	-	0.391	0.0521	0.492	23.0
Rxn #12	FTL Plate 3	A1: NFC	-	0.072	0.0641	0.555	98.4
Rxn #12	FTL Plate 3	B1	50	0.339	0.0641	0.555	44.0
Rxn #12	FTL Plate 3	C1	50	0.523	0.0641	0.555	6.5
Rxn #12	FTL Plate 3	D1	50	0.502	0.0641	0.555	10.8
Rxn #12	FTL Plate 3	E1	50	0.533	0.0641	0.555	4.5
Rxn #12	FTL Plate 3	F1	50	0.518	0.0641	0.555	7.5
Rxn #12	FTL Plate 3	G1	50	0.536	0.0641	0.555	3.9
Rxn #12	FTL Plate 3	H1: FOC	-	0.541	0.0641	0.555	2.9
Rxn #12	FTL Plate 3	A2: NFC	-	0.066	0.0641	0.555	99.6
Rxn #12	FTL Plate 3	B2	50	0.568	0.0641	0.555	-2.6
Rxn #12	FTL Plate 3	C2	50	0.545	0.0641	0.555	2.0
Rxn #12	FTL Plate 3	D2	50	0.271	0.0641	0.555	57.9
Rxn #12	FTL Plate 3	E2	50	0.549	0.0641	0.555	1.2
Rxn #12	FTL Plate 3	F2	50	0.558	0.0641	0.555	-0.6
Rxn #12	FTL Plate 3	G2	50	0.537	0.0641	0.555	3.7
Rxn #12	FTL Plate 3	H2: FOC	-	0.555	0.0641	0.555	0.0
Rxn #12	FTL Plate 3	A3: NFC	-	0.072	0.0641	0.555	98.4
Rxn #12	FTL Plate 3	B3	50	0.551	0.0641	0.555	0.8
Rxn #12	FTL Plate 3	C3	50	0.238	0.0641	0.555	64.6
Rxn #12	FTL Plate 3	D3	50	0.571	0.0641	0.555	-3.3
Rxn #12	FTL Plate 3	E3	50	0.563	0.0641	0.555	-1.6
Rxn #12	FTL Plate 3	F3	50	0.566	0.0641	0.555	-2.2
Rxn #12	FTL Plate 3	G3	50	0.561	0.0641	0.555	-1.2
Rxn #12	FTL Plate 3	H3: FOC	-	0.563	0.0641	0.555	-1.6
Rxn #12	FTL Plate 3	A4: NFC	-	0.065	0.0641	0.555	99.8
Rxn #12	FTL Plate 3	B4	50	0.580	0.0641	0.555	-5.1
Rxn #12	FTL Plate 3	C4	50	0.552	0.0641	0.555	0.6
Rxn #12	FTL Plate 3	D4	50	0.544	0.0641	0.555	2.2
Rxn #12	FTL Plate 3	E4	50	0.556	0.0641	0.555	-0.2
Rxn #12	FTL Plate 3	F4	50	0.564	0.0641	0.555	-1.8

S1 Table. Data from Screening of Botanical Extract Library for Inhibition of KHKC Activity.

Rxn ID	Sample			Sample	Ave. No Fructose Controls	Ave. Fructose Only Controls	KHKC
	Botanical Plate	Well	Conc (μg/mL)	(ΔOD_{340nm})	(ΔOD_{340nm})	(ΔOD_{340nm})	Inhibition (%)
Rxn #12	FTL Plate 3	G4	50	0.589	0.0641	0.555	-6.9
Rxn #12	FTL Plate 3	H4: FOC	-	0.576	0.0641	0.555	-4.3
Rxn #12	FTL Plate 3	A5: NFC	-	0.057	0.0641	0.555	101.4
Rxn #12	FTL Plate 3	B5: NFC	-	0.059	0.0641	0.555	101.0
Rxn #12	FTL Plate 3	C5: NFC	-	0.065	0.0641	0.555	99.8
Rxn #12	FTL Plate 3	D5: NFC	-	0.061	0.0641	0.555	100.6
Rxn #12	FTL Plate 3	E5: FOC	-	0.540	0.0641	0.555	3.1
Rxn #12	FTL Plate 3	F5: FOC	-	0.555	0.0641	0.555	0.0
Rxn #12	FTL Plate 3	G5: FOC	-	0.547	0.0641	0.555	1.6
Rxn #12	FTL Plate 3	H5: FOC	-	0.565	0.0641	0.555	-2.0
Rxn #12	FTL Plate 3	A6: NFC	-	0.062	0.0641	0.555	100.4
Rxn #12	FTL Plate 3	B6	50	0.583	0.0641	0.555	-5.7
Rxn #12	FTL Plate 3	C6	50	0.502	0.0641	0.555	10.8
Rxn #12	FTL Plate 3	D6	50	0.576	0.0641	0.555	-4.3
Rxn #12	FTL Plate 3	E6	50	0.575	0.0641	0.555	-4.1
Rxn #12	FTL Plate 3	F6	50	0.562	0.0641	0.555	-1.4
Rxn #12	FTL Plate 3	G6	50	0.533	0.0641	0.555	4.5
Rxn #12	FTL Plate 3	H6: FOC	-	0.554	0.0641	0.555	0.2
Rxn #12	FTL Plate 3	A7: NFC	-	0.054	0.0641	0.555	102.1
Rxn #12	FTL Plate 3	B7	50	0.549	0.0641	0.555	1.2
Rxn #12	FTL Plate 3	C7	50	0.572	0.0641	0.555	-3.5
Rxn #12	FTL Plate 3	D7	50	0.561	0.0641	0.555	-1.2
Rxn #12	FTL Plate 3	E7	50	0.579	0.0641	0.555	-4.9
Rxn #12	FTL Plate 3	F7	50	0.499	0.0641	0.555	11.4
Rxn #12	FTL Plate 3	G7	50	0.556	0.0641	0.555	-0.2
Rxn #12	FTL Plate 3	H7: FOC	-	0.560	0.0641	0.555	-1.0
Rxn #12	FTL Plate 3	A8: NFC	-	0.063	0.0641	0.555	100.2
Rxn #12	FTL Plate 3	B8	50	0.565	0.0641	0.555	-2.0
Rxn #12	FTL Plate 3	C8	50	0.559	0.0641	0.555	-0.8
Rxn #12	FTL Plate 3	D8	50	0.567	0.0641	0.555	-2.4
Rxn #12	FTL Plate 3	E8	50	0.540	0.0641	0.555	3.1
Rxn #12	FTL Plate 3	F8	50	0.550	0.0641	0.555	1.0
Rxn #12	FTL Plate 3	G8	50	0.549	0.0641	0.555	1.2
Rxn #12	FTL Plate 3	H8: FOC	-	0.551	0.0641	0.555	0.8
Rxn #12	FTL Plate 3	A9: NFC	-	0.072	0.0641	0.555	98.4
Rxn #12	FTL Plate 3	B9	50	0.525	0.0641	0.555	6.1
Rxn #12	FTL Plate 3	C9	50	0.566	0.0641	0.555	-2.2
Rxn #12	FTL Plate 3	D9	50	0.547	0.0641	0.555	1.6
Rxn #12	FTL Plate 3	E9	50	0.553	0.0641	0.555	0.4
Rxn #12	FTL Plate 3	F9	50	0.544	0.0641	0.555	2.2
Rxn #12	FTL Plate 3	G9	50	0.545	0.0641	0.555	2.0
Rxn #12	FTL Plate 3	H9: FOC	-	0.555	0.0641	0.555	0.0
Rxn #12	FTL Plate 3	A10: NFC	-	0.065	0.0641	0.555	99.8
Rxn #12	FTL Plate 3	B10	50	0.552	0.0641	0.555	0.6
Rxn #12	FTL Plate 3	C10	50	0.530	0.0641	0.555	5.1
Rxn #12	FTL Plate 3	D10	50	0.551	0.0641	0.555	0.8
Rxn #12	FTL Plate 3	E10	50	0.546	0.0641	0.555	1.8
Rxn #12	FTL Plate 3	F10	50	0.530	0.0641	0.555	5.1
Rxn #12	FTL Plate 3	G10	50	0.538	0.0641	0.555	3.5
Rxn #12	FTL Plate 3	H10: FOC	-	0.551	0.0641	0.555	0.8
Rxn #12	FTL Plate 3	A11: NFC	-	0.065	0.0641	0.555	99.8
Rxn #12	FTL Plate 3	B11	50	0.525	0.0641	0.555	6.1
Rxn #12	FTL Plate 3	C11	50	0.502	0.0641	0.555	10.8
Rxn #12	FTL Plate 3	D11	50	0.523	0.0641	0.555	6.5
Rxn #12	FTL Plate 3	E11	50	0.532	0.0641	0.555	4.7
Rxn #12	FTL Plate 3	F11	50	0.536	0.0641	0.555	3.9
Rxn #12	FTL Plate 3	G11	50	0.507	0.0641	0.555	9.8
Rxn #12	FTL Plate 3	H11: FOC	-	0.540	0.0641	0.555	3.1
Rxn #12	FTL Plate 3	A12: NFC	-	0.064	0.0641	0.555	100.0
Rxn #12	FTL Plate 3	H12: FOC	-	0.566	0.0641	0.555	-2.2
Rxn #13	FTL Plate 5	A1: NFC	-	0.064	0.0709	0.594	101.3
Rxn #13	FTL Plate 5	B1	50	0.609	0.0709	0.594	-2.9
Rxn #13	FTL Plate 5	C1	50	0.618	0.0709	0.594	-4.6
Rxn #13	FTL Plate 5	D1	50	0.612	0.0709	0.594	-3.4
Rxn #13	FTL Plate 5	E1	50	0.579	0.0709	0.594	2.9
Rxn #13	FTL Plate 5	F1	50	0.597	0.0709	0.594	-0.6
Rxn #13	FTL Plate 5	G1	50	0.600	0.0709	0.594	-1.1
Rxn #13	FTL Plate 5	H1: FOC	-	0.612	0.0709	0.594	-3.4
Rxn #13	FTL Plate 5	A2: NFC	-	0.089	0.0709	0.594	96.5
Rxn #13	FTL Plate 5	B2	50	0.610	0.0709	0.594	-3.1

S1 Table. Data from Screening of Botanical Extract Library for Inhibition of KHKC Activity.

Rxn ID	Sample			Sample	Ave. No Fructose Controls	Ave. Fructose Only Controls	KHKC
	Botanical Plate	Well	Conc (μg/mL)	(ΔOD_{340nm})	(ΔOD_{340nm})	(ΔOD_{340nm})	Inhibition (%)
Rxn #13	FTL Plate 5	C2	50	0.616	0.0709	0.594	-4.2
Rxn #13	FTL Plate 5	D2	50	0.589	0.0709	0.594	1.0
Rxn #13	FTL Plate 5	E2	50	0.596	0.0709	0.594	-0.4
Rxn #13	FTL Plate 5	F2	50	0.601	0.0709	0.594	-1.3
Rxn #13	FTL Plate 5	G2	50	0.575	0.0709	0.594	3.6
Rxn #13	FTL Plate 5	H2: FOC	-	0.600	0.0709	0.594	-1.1
Rxn #13	FTL Plate 5	A3: NFC	-	0.087	0.0709	0.594	96.9
Rxn #13	FTL Plate 5	B3	50	0.615	0.0709	0.594	-4.0
Rxn #13	FTL Plate 5	C3	50	0.600	0.0709	0.594	-1.1
Rxn #13	FTL Plate 5	D3	50	0.614	0.0709	0.594	-3.8
Rxn #13	FTL Plate 5	E3	50	0.598	0.0709	0.594	-0.8
Rxn #13	FTL Plate 5	F3	50	0.564	0.0709	0.594	5.7
Rxn #13	FTL Plate 5	G3	50	0.598	0.0709	0.594	-0.8
Rxn #13	FTL Plate 5	H3: FOC	-	0.600	0.0709	0.594	-1.1
Rxn #13	FTL Plate 5	A4: NFC	-	0.076	0.0709	0.594	99.0
Rxn #13	FTL Plate 5	B4	50	0.648	0.0709	0.594	-10.3
Rxn #13	FTL Plate 5	C4	50	0.573	0.0709	0.594	4.0
Rxn #13	FTL Plate 5	D4	50	0.572	0.0709	0.594	4.2
Rxn #13	FTL Plate 5	E4	50	0.604	0.0709	0.594	-1.9
Rxn #13	FTL Plate 5	F4	50	0.599	0.0709	0.594	-1.0
Rxn #13	FTL Plate 5	G4	50	0.563	0.0709	0.594	5.9
Rxn #13	FTL Plate 5	H4: FOC	-	0.602	0.0709	0.594	-1.5
Rxn #13	FTL Plate 5	A5: NFC	-	0.053	0.0709	0.594	103.4
Rxn #13	FTL Plate 5	B5: NFC	-	0.058	0.0709	0.594	102.5
Rxn #13	FTL Plate 5	C5: NFC	-	0.061	0.0709	0.594	101.9
Rxn #13	FTL Plate 5	D5: NFC	-	0.065	0.0709	0.594	101.1
Rxn #13	FTL Plate 5	E5: FOC	-	0.579	0.0709	0.594	2.9
Rxn #13	FTL Plate 5	F5: FOC	-	0.583	0.0709	0.594	2.1
Rxn #13	FTL Plate 5	G5: FOC	-	0.591	0.0709	0.594	0.6
Rxn #13	FTL Plate 5	H5: FOC	-	0.580	0.0709	0.594	2.7
Rxn #13	FTL Plate 5	A6: NFC	-	0.074	0.0709	0.594	99.4
Rxn #13	FTL Plate 5	B6	50	0.615	0.0709	0.594	-4.0
Rxn #13	FTL Plate 5	C6	50	0.587	0.0709	0.594	1.3
Rxn #13	FTL Plate 5	D6	50	0.619	0.0709	0.594	-4.8
Rxn #13	FTL Plate 5	E6	50	0.496	0.0709	0.594	18.7
Rxn #13	FTL Plate 5	F6	50	0.588	0.0709	0.594	1.1
Rxn #13	FTL Plate 5	G6	50	0.594	0.0709	0.594	0.0
Rxn #13	FTL Plate 5	H6: FOC	-	0.611	0.0709	0.594	-3.2
Rxn #13	FTL Plate 5	A7: NFC	-	0.079	0.0709	0.594	98.5
Rxn #13	FTL Plate 5	B7	50	0.602	0.0709	0.594	-1.5
Rxn #13	FTL Plate 5	C7	50	0.625	0.0709	0.594	-5.9
Rxn #13	FTL Plate 5	D7	50	0.605	0.0709	0.594	-2.1
Rxn #13	FTL Plate 5	E7	50	0.576	0.0709	0.594	3.4
Rxn #13	FTL Plate 5	F7	50	0.588	0.0709	0.594	1.1
Rxn #13	FTL Plate 5	G7	50	0.580	0.0709	0.594	2.7
Rxn #13	FTL Plate 5	H7: FOC	-	0.590	0.0709	0.594	0.8
Rxn #13	FTL Plate 5	A8: NFC	-	0.073	0.0709	0.594	99.6
Rxn #13	FTL Plate 5	B8	50	0.588	0.0709	0.594	1.1
Rxn #13	FTL Plate 5	C8	50	0.599	0.0709	0.594	-1.0
Rxn #13	FTL Plate 5	D8	50	0.577	0.0709	0.594	3.2
Rxn #13	FTL Plate 5	E8	50	0.581	0.0709	0.594	2.5
Rxn #13	FTL Plate 5	F8	50	0.564	0.0709	0.594	5.7
Rxn #13	FTL Plate 5	G8	50	0.549	0.0709	0.594	8.6
Rxn #13	FTL Plate 5	H8: FOC	-	0.585	0.0709	0.594	1.7
Rxn #13	FTL Plate 5	A9: NFC	-	0.063	0.0709	0.594	101.5
Rxn #13	FTL Plate 5	B9	50	0.605	0.0709	0.594	-2.1
Rxn #13	FTL Plate 5	C9	50	0.599	0.0709	0.594	-1.0
Rxn #13	FTL Plate 5	D9	50	0.548	0.0709	0.594	8.8
Rxn #13	FTL Plate 5	E9	50	0.585	0.0709	0.594	1.7
Rxn #13	FTL Plate 5	F9	50	0.562	0.0709	0.594	6.1
Rxn #13	FTL Plate 5	G9	50	0.571	0.0709	0.594	4.4
Rxn #13	FTL Plate 5	H9: FOC	-	0.580	0.0709	0.594	2.7
Rxn #13	FTL Plate 5	A10: NFC	-	0.068	0.0709	0.594	100.6
Rxn #13	FTL Plate 5	B10	50	0.603	0.0709	0.594	-1.7
Rxn #13	FTL Plate 5	C10	50	0.520	0.0709	0.594	14.1
Rxn #13	FTL Plate 5	D10	50	0.579	0.0709	0.594	2.9
Rxn #13	FTL Plate 5	E10	50	0.588	0.0709	0.594	1.1
Rxn #13	FTL Plate 5	F10	50	0.573	0.0709	0.594	4.0
Rxn #13	FTL Plate 5	G10	50	0.577	0.0709	0.594	3.2
Rxn #13	FTL Plate 5	H10: FOC	-	0.600	0.0709	0.594	-1.1

S1 Table. Data from Screening of Botanical Extract Library for Inhibition of KHKC Activity.

Rxn ID	Sample			Sample	Ave. No Fructose Controls	Ave. Fructose Only Controls	KHKC
	Botanical Plate	Well	Conc (μg/mL)	(ΔOD_{340nm})	(ΔOD_{340nm})	(ΔOD_{340nm})	Inhibition (%)
Rxn #13	FTL Plate 5	A11: NFC	-	0.073	0.0709	0.594	99.6
Rxn #13	FTL Plate 5	B11	50	0.570	0.0709	0.594	4.6
Rxn #13	FTL Plate 5	C11	50	0.582	0.0709	0.594	2.3
Rxn #13	FTL Plate 5	D11	50	0.557	0.0709	0.594	7.1
Rxn #13	FTL Plate 5	E11	50	0.583	0.0709	0.594	2.1
Rxn #13	FTL Plate 5	F11	50	0.580	0.0709	0.594	2.7
Rxn #13	FTL Plate 5	G11	50	0.585	0.0709	0.594	1.7
Rxn #13	FTL Plate 5	H11: FOC	-	0.592	0.0709	0.594	0.4
Rxn #13	FTL Plate 5	A12: NFC	-	0.080	0.0709	0.594	98.3
Rxn #13	FTL Plate 5	H12: FOC	-	0.606	0.0709	0.594	-2.3
Rxn #14	FTL Plate 6	A1: NFC	-	0.050	0.0534	0.539	100.7
Rxn #14	FTL Plate 6	B1	50	0.531	0.0534	0.539	1.6
Rxn #14	FTL Plate 6	C1	50	0.497	0.0534	0.539	8.6
Rxn #14	FTL Plate 6	D1	50	0.458	0.0534	0.539	16.7
Rxn #14	FTL Plate 6	E1	50	0.499	0.0534	0.539	8.2
Rxn #14	FTL Plate 6	F1	50	0.499	0.0534	0.539	8.2
Rxn #14	FTL Plate 6	G1	50	0.484	0.0534	0.539	11.3
Rxn #14	FTL Plate 6	H1: FOC	-	0.502	0.0534	0.539	7.6
Rxn #14	FTL Plate 6	A2: NFC	-	0.045	0.0534	0.539	101.7
Rxn #14	FTL Plate 6	B2	50	0.484	0.0534	0.539	11.3
Rxn #14	FTL Plate 6	C2	50	0.531	0.0534	0.539	1.6
Rxn #14	FTL Plate 6	D2	50	0.526	0.0534	0.539	2.7
Rxn #14	FTL Plate 6	E2	50	0.516	0.0534	0.539	4.7
Rxn #14	FTL Plate 6	F2	50	0.504	0.0534	0.539	7.2
Rxn #14	FTL Plate 6	G2	50	0.524	0.0534	0.539	3.1
Rxn #14	FTL Plate 6	H2: FOC	-	0.533	0.0534	0.539	1.2
Rxn #14	FTL Plate 6	A3: NFC	-	0.043	0.0534	0.539	102.1
Rxn #14	FTL Plate 6	B3	50	0.486	0.0534	0.539	10.9
Rxn #14	FTL Plate 6	C3	50	0.569	0.0534	0.539	-6.2
Rxn #14	FTL Plate 6	D3	50	0.497	0.0534	0.539	8.6
Rxn #14	FTL Plate 6	E3	50	0.525	0.0534	0.539	2.9
Rxn #14	FTL Plate 6	F3	50	0.535	0.0534	0.539	0.8
Rxn #14	FTL Plate 6	G3	50	0.535	0.0534	0.539	0.8
Rxn #14	FTL Plate 6	H3: FOC	-	0.537	0.0534	0.539	0.4
Rxn #14	FTL Plate 6	A4: NFC	-	0.054	0.0534	0.539	99.9
Rxn #14	FTL Plate 6	B4	50	0.544	0.0534	0.539	-1.0
Rxn #14	FTL Plate 6	C4	50	0.577	0.0534	0.539	-7.8
Rxn #14	FTL Plate 6	D4	50	0.540	0.0534	0.539	-0.2
Rxn #14	FTL Plate 6	E4	50	0.536	0.0534	0.539	0.6
Rxn #14	FTL Plate 6	F4	50	0.530	0.0534	0.539	1.9
Rxn #14	FTL Plate 6	G4	50	0.571	0.0534	0.539	-6.6
Rxn #14	FTL Plate 6	H4: FOC	-	0.526	0.0534	0.539	2.7
Rxn #14	FTL Plate 6	A5: NFC	-	0.054	0.0534	0.539	99.9
Rxn #14	FTL Plate 6	B5: NFC	-	0.056	0.0534	0.539	99.5
Rxn #14	FTL Plate 6	C5: NFC	-	0.045	0.0534	0.539	101.7
Rxn #14	FTL Plate 6	D5: NFC	-	0.040	0.0534	0.539	102.8
Rxn #14	FTL Plate 6	E5: FOC	-	0.545	0.0534	0.539	-1.2
Rxn #14	FTL Plate 6	F5: FOC	-	0.545	0.0534	0.539	-1.2
Rxn #14	FTL Plate 6	G5: FOC	-	0.537	0.0534	0.539	0.4
Rxn #14	FTL Plate 6	H5: FOC	-	0.546	0.0534	0.539	-1.4
Rxn #14	FTL Plate 6	A6: NFC	-	0.060	0.0534	0.539	98.6
Rxn #14	FTL Plate 6	B6	50	0.548	0.0534	0.539	-1.9
Rxn #14	FTL Plate 6	C6	50	0.533	0.0534	0.539	1.2
Rxn #14	FTL Plate 6	D6	50	0.537	0.0534	0.539	0.4
Rxn #14	FTL Plate 6	E6	50	0.514	0.0534	0.539	5.1
Rxn #14	FTL Plate 6	F6	50	0.571	0.0534	0.539	-6.6
Rxn #14	FTL Plate 6	G6	50	0.516	0.0534	0.539	4.7
Rxn #14	FTL Plate 6	H6: FOC	-	0.542	0.0534	0.539	-0.6
Rxn #14	FTL Plate 6	A7: NFC	-	0.064	0.0534	0.539	97.8
Rxn #14	FTL Plate 6	B7	50	0.532	0.0534	0.539	1.4
Rxn #14	FTL Plate 6	C7	50	0.549	0.0534	0.539	-2.1
Rxn #14	FTL Plate 6	D7	50	0.550	0.0534	0.539	-2.3
Rxn #14	FTL Plate 6	E7	50	0.543	0.0534	0.539	-0.8
Rxn #14	FTL Plate 6	F7	50	0.520	0.0534	0.539	3.9
Rxn #14	FTL Plate 6	G7	50	0.497	0.0534	0.539	8.6
Rxn #14	FTL Plate 6	H7: FOC	-	0.551	0.0534	0.539	-2.5
Rxn #14	FTL Plate 6	A8: NFC	-	0.063	0.0534	0.539	98.0
Rxn #14	FTL Plate 6	B8	50	0.548	0.0534	0.539	-1.9
Rxn #14	FTL Plate 6	C8	50	0.562	0.0534	0.539	-4.7
Rxn #14	FTL Plate 6	D8	50	0.506	0.0534	0.539	6.8

S1 Table. Data from Screening of Botanical Extract Library for Inhibition of KHKC Activity.

Rxn ID	Sample			Sample	Ave. No Fructose Controls	Ave. Fructose Only Controls	KHKC
	Botanical Plate	Well	Conc (μg/mL)	(ΔOD_{340nm})	(ΔOD_{340nm})	(ΔOD_{340nm})	Inhibition (%)
Rxn #14	FTL Plate 6	E8	50	0.548	0.0534	0.539	-1.9
Rxn #14	FTL Plate 6	F8	50	0.511	0.0534	0.539	5.8
Rxn #14	FTL Plate 6	G8	50	0.541	0.0534	0.539	-0.4
Rxn #14	FTL Plate 6	H8: FOC	-	0.556	0.0534	0.539	-3.5
Rxn #14	FTL Plate 6	A9: NFC	-	0.053	0.0534	0.539	100.1
Rxn #14	FTL Plate 6	B9	50	0.521	0.0534	0.539	3.7
Rxn #14	FTL Plate 6	C9	50	0.550	0.0534	0.539	-2.3
Rxn #14	FTL Plate 6	D9	50	0.528	0.0534	0.539	2.3
Rxn #14	FTL Plate 6	E9	50	0.553	0.0534	0.539	-2.9
Rxn #14	FTL Plate 6	F9	50	0.546	0.0534	0.539	-1.4
Rxn #14	FTL Plate 6	G9	50	0.540	0.0534	0.539	-0.2
Rxn #14	FTL Plate 6	H9: FOC	-	0.540	0.0534	0.539	-0.2
Rxn #14	FTL Plate 6	A10: NFC	-	0.053	0.0534	0.539	100.1
Rxn #14	FTL Plate 6	B10	50	0.570	0.0534	0.539	-6.4
Rxn #14	FTL Plate 6	C10	50	0.556	0.0534	0.539	-3.5
Rxn #14	FTL Plate 6	D10	50	0.521	0.0534	0.539	3.7
Rxn #14	FTL Plate 6	E10	50	0.570	0.0534	0.539	-6.4
Rxn #14	FTL Plate 6	F10	50	0.541	0.0534	0.539	-0.4
Rxn #14	FTL Plate 6	G10	50	0.557	0.0534	0.539	-3.7
Rxn #14	FTL Plate 6	H10: FOC	-	0.543	0.0534	0.539	-0.8
Rxn #14	FTL Plate 6	A11: NFC	-	0.062	0.0534	0.539	98.2
Rxn #14	FTL Plate 6	B11	50	0.584	0.0534	0.539	-9.3
Rxn #14	FTL Plate 6	C11	50	0.523	0.0534	0.539	3.3
Rxn #14	FTL Plate 6	D11	50	0.569	0.0534	0.539	-6.2
Rxn #14	FTL Plate 6	E11	50	0.557	0.0534	0.539	-3.7
Rxn #14	FTL Plate 6	F11	50	0.552	0.0534	0.539	-2.7
Rxn #14	FTL Plate 6	G11	50	0.568	0.0534	0.539	-6.0
Rxn #14	FTL Plate 6	H11: FOC	-	0.544	0.0534	0.539	-1.0
Rxn #14	FTL Plate 6	A12: NFC	-	0.059	0.0534	0.539	98.8
Rxn #14	FTL Plate 6	H12: FOC	-	0.541	0.0534	0.539	-0.4
Rxn #15	FTL Plate 7	A1: NFC	-	0.067	0.0661	0.520	99.8
Rxn #15	FTL Plate 7	B1	50	0.472	0.0661	0.520	10.6
Rxn #15	FTL Plate 7	C1	50	0.485	0.0661	0.520	7.7
Rxn #15	FTL Plate 7	D1	50	0.410	0.0661	0.520	24.2
Rxn #15	FTL Plate 7	E1	50	0.457	0.0661	0.520	13.9
Rxn #15	FTL Plate 7	F1	50	0.483	0.0661	0.520	8.2
Rxn #15	FTL Plate 7	G1	50	0.451	0.0661	0.520	15.2
Rxn #15	FTL Plate 7	H1: FOC	-	0.498	0.0661	0.520	4.8
Rxn #15	FTL Plate 7	A2: NFC	-	0.073	0.0661	0.520	98.5
Rxn #15	FTL Plate 7	B2	50	0.453	0.0661	0.520	14.8
Rxn #15	FTL Plate 7	C2	50	0.485	0.0661	0.520	7.7
Rxn #15	FTL Plate 7	D2	50	0.507	0.0661	0.520	2.9
Rxn #15	FTL Plate 7	E2	50	0.447	0.0661	0.520	16.1
Rxn #15	FTL Plate 7	F2	50	0.484	0.0661	0.520	7.9
Rxn #15	FTL Plate 7	G2	50	0.492	0.0661	0.520	6.2
Rxn #15	FTL Plate 7	H2: FOC	-	0.522	0.0661	0.520	-0.4
Rxn #15	FTL Plate 7	A3: NFC	-	0.062	0.0661	0.520	100.9
Rxn #15	FTL Plate 7	B3	50	0.511	0.0661	0.520	2.0
Rxn #15	FTL Plate 7	C3	50	0.496	0.0661	0.520	5.3
Rxn #15	FTL Plate 7	D3	50	0.493	0.0661	0.520	5.9
Rxn #15	FTL Plate 7	E3	50	0.518	0.0661	0.520	0.4
Rxn #15	FTL Plate 7	F3	50	0.447	0.0661	0.520	16.1
Rxn #15	FTL Plate 7	G3	50	0.489	0.0661	0.520	6.8
Rxn #15	FTL Plate 7	H3: FOC	-	0.525	0.0661	0.520	-1.1
Rxn #15	FTL Plate 7	A4: NFC	-	0.066	0.0661	0.520	100.0
Rxn #15	FTL Plate 7	B4	50	0.492	0.0661	0.520	6.2
Rxn #15	FTL Plate 7	C4	50	0.496	0.0661	0.520	5.3
Rxn #15	FTL Plate 7	D4	50	0.514	0.0661	0.520	1.3
Rxn #15	FTL Plate 7	E4	50	0.533	0.0661	0.520	-2.9
Rxn #15	FTL Plate 7	F4	50	0.488	0.0661	0.520	7.1
Rxn #15	FTL Plate 7	G4	50	0.485	0.0661	0.520	7.7
Rxn #15	FTL Plate 7	H4: FOC	-	0.538	0.0661	0.520	-4.0
Rxn #15	FTL Plate 7	A5: NFC	-	0.064	0.0661	0.520	100.5
Rxn #15	FTL Plate 7	B5: NFC	-	0.063	0.0661	0.520	100.7
Rxn #15	FTL Plate 7	C5: NFC	-	0.058	0.0661	0.520	101.8
Rxn #15	FTL Plate 7	D5: NFC	-	0.054	0.0661	0.520	102.7
Rxn #15	FTL Plate 7	E5: FOC	-	0.534	0.0661	0.520	-3.1
Rxn #15	FTL Plate 7	F5: FOC	-	0.517	0.0661	0.520	0.7
Rxn #15	FTL Plate 7	G5: FOC	-	0.510	0.0661	0.520	2.2
Rxn #15	FTL Plate 7	H5: FOC	-	0.524	0.0661	0.520	-0.9

S1 Table. Data from Screening of Botanical Extract Library for Inhibition of KHKC Activity.

Rxn ID	Sample			Sample	Ave. No Fructose Controls	Ave. Fructose Only Controls	KHKC
	Botanical Plate	Well	Conc (μg/mL)	(ΔOD_{340nm})	(ΔOD_{340nm})	(ΔOD_{340nm})	Inhibition (%)
Rxn #15	FTL Plate 7	A6: NFC	-	0.063	0.0661	0.520	100.7
Rxn #15	FTL Plate 7	B6	50	0.510	0.0661	0.520	2.2
Rxn #15	FTL Plate 7	C6	50	0.517	0.0661	0.520	0.7
Rxn #15	FTL Plate 7	D6	50	0.390	0.0661	0.520	28.6
Rxn #15	FTL Plate 7	E6	50	0.480	0.0661	0.520	8.8
Rxn #15	FTL Plate 7	F6	50	0.538	0.0661	0.520	-4.0
Rxn #15	FTL Plate 7	G6	50	0.499	0.0661	0.520	4.6
Rxn #15	FTL Plate 7	H6: FOC	-	0.535	0.0661	0.520	-3.3
Rxn #15	FTL Plate 7	A7: NFC	-	0.079	0.0661	0.520	97.2
Rxn #15	FTL Plate 7	B7	50	0.527	0.0661	0.520	-1.5
Rxn #15	FTL Plate 7	C7	50	0.374	0.0661	0.520	32.2
Rxn #15	FTL Plate 7	D7	50	0.536	0.0661	0.520	-3.5
Rxn #15	FTL Plate 7	E7	50	0.463	0.0661	0.520	12.6
Rxn #15	FTL Plate 7	F7	50	0.493	0.0661	0.520	5.9
Rxn #15	FTL Plate 7	G7	50	0.495	0.0661	0.520	5.5
Rxn #15	FTL Plate 7	H7: FOC	-	0.527	0.0661	0.520	-1.5
Rxn #15	FTL Plate 7	A8: NFC	-	0.069	0.0661	0.520	99.4
Rxn #15	FTL Plate 7	B8	50	0.506	0.0661	0.520	3.1
Rxn #15	FTL Plate 7	C8	50	0.509	0.0661	0.520	2.4
Rxn #15	FTL Plate 7	D8	50	0.489	0.0661	0.520	6.8
Rxn #15	FTL Plate 7	E8	50	0.532	0.0661	0.520	-2.6
Rxn #15	FTL Plate 7	F8	50	0.484	0.0661	0.520	7.9
Rxn #15	FTL Plate 7	G8	50	0.456	0.0661	0.520	14.1
Rxn #15	FTL Plate 7	H8: FOC	-	0.519	0.0661	0.520	0.2
Rxn #15	FTL Plate 7	A9: NFC	-	0.064	0.0661	0.520	100.5
Rxn #15	FTL Plate 7	B9	50	0.476	0.0661	0.520	9.7
Rxn #15	FTL Plate 7	C9	50	0.502	0.0661	0.520	4.0
Rxn #15	FTL Plate 7	D9	50	0.436	0.0661	0.520	18.5
Rxn #15	FTL Plate 7	E9	50	0.520	0.0661	0.520	0.0
Rxn #15	FTL Plate 7	F9	50	0.523	0.0661	0.520	-0.7
Rxn #15	FTL Plate 7	G9	50	0.500	0.0661	0.520	4.4
Rxn #15	FTL Plate 7	H9: FOC	-	0.518	0.0661	0.520	0.4
Rxn #15	FTL Plate 7	A10: NFC	-	0.065	0.0661	0.520	100.2
Rxn #15	FTL Plate 7	B10	50	0.517	0.0661	0.520	0.7
Rxn #15	FTL Plate 7	C10	50	0.416	0.0661	0.520	22.9
Rxn #15	FTL Plate 7	D10	50	0.450	0.0661	0.520	15.4
Rxn #15	FTL Plate 7	E10	50	0.550	0.0661	0.520	-6.6
Rxn #15	FTL Plate 7	F10	50	0.478	0.0661	0.520	9.3
Rxn #15	FTL Plate 7	G10	50	0.486	0.0661	0.520	7.5
Rxn #15	FTL Plate 7	H10: FOC	-	0.513	0.0661	0.520	1.5
Rxn #15	FTL Plate 7	A11: NFC	-	0.072	0.0661	0.520	98.7
Rxn #15	FTL Plate 7	B11	50	0.516	0.0661	0.520	0.9
Rxn #15	FTL Plate 7	C11	50	0.440	0.0661	0.520	17.6
Rxn #15	FTL Plate 7	D11	50	0.460	0.0661	0.520	13.2
Rxn #15	FTL Plate 7	E11	50	0.492	0.0661	0.520	6.2
Rxn #15	FTL Plate 7	F11	50	0.515	0.0661	0.520	1.1
Rxn #15	FTL Plate 7	G11	50	0.533	0.0661	0.520	-2.9
Rxn #15	FTL Plate 7	H11: FOC	-	0.534	0.0661	0.520	-3.1
Rxn #15	FTL Plate 7	A12: NFC	-	0.072	0.0661	0.520	98.7
Rxn #15	FTL Plate 7	H12: FOC	-	0.491	0.0661	0.520	6.4
Rxn #16	FTL Plate 8	A1: NFC	-	0.067	0.0650	0.538	99.6
Rxn #16	FTL Plate 8	B1	50	0.526	0.0650	0.538	2.5
Rxn #16	FTL Plate 8	C1	50	0.498	0.0650	0.538	8.5
Rxn #16	FTL Plate 8	D1	50	0.466	0.0650	0.538	15.2
Rxn #16	FTL Plate 8	E1	50	0.542	0.0650	0.538	-0.8
Rxn #16	FTL Plate 8	F1	50	0.524	0.0650	0.538	3.0
Rxn #16	FTL Plate 8	G1	50	0.530	0.0650	0.538	1.7
Rxn #16	FTL Plate 8	H1: FOC	-	0.505	0.0650	0.538	7.0
Rxn #16	FTL Plate 8	A2: NFC	-	0.079	0.0650	0.538	97.0
Rxn #16	FTL Plate 8	B2	50	0.599	0.0650	0.538	-12.9
Rxn #16	FTL Plate 8	C2	50	0.506	0.0650	0.538	6.8
Rxn #16	FTL Plate 8	D2	50	0.529	0.0650	0.538	1.9
Rxn #16	FTL Plate 8	E2	50	0.589	0.0650	0.538	-10.8
Rxn #16	FTL Plate 8	F2	50	0.555	0.0650	0.538	-3.6
Rxn #16	FTL Plate 8	G2	50	0.523	0.0650	0.538	3.2
Rxn #16	FTL Plate 8	H2: FOC	-	0.546	0.0650	0.538	-1.7
Rxn #16	FTL Plate 8	A3: NFC	-	0.062	0.0650	0.538	100.6
Rxn #16	FTL Plate 8	B3	50	0.549	0.0650	0.538	-2.3
Rxn #16	FTL Plate 8	C3	50	0.548	0.0650	0.538	-2.1
Rxn #16	FTL Plate 8	D3	50	0.555	0.0650	0.538	-3.6

S1 Table. Data from Screening of Botanical Extract Library for Inhibition of KHKC Activity.

Rxn ID	Sample			Sample	Ave. No Fructose Controls	Ave. Fructose Only Controls	KHKC
	Botanical Plate	Well	Conc (μg/mL)	(ΔOD_{340nm})	(ΔOD_{340nm})	(ΔOD_{340nm})	Inhibition (%)
Rxn #16	FTL Plate 8	E3	50	0.548	0.0650	0.538	-2.1
Rxn #16	FTL Plate 8	F3	50	0.560	0.0650	0.538	-4.7
Rxn #16	FTL Plate 8	G3	50	0.577	0.0650	0.538	-8.2
Rxn #16	FTL Plate 8	H3: FOC	-	0.552	0.0650	0.538	-3.0
Rxn #16	FTL Plate 8	A4: NFC	-	0.065	0.0650	0.538	100.0
Rxn #16	FTL Plate 8	B4	50	0.583	0.0650	0.538	-9.5
Rxn #16	FTL Plate 8	C4	50	0.588	0.0650	0.538	-10.6
Rxn #16	FTL Plate 8	D4	50	0.511	0.0650	0.538	5.7
Rxn #16	FTL Plate 8	E4	50	0.541	0.0650	0.538	-0.6
Rxn #16	FTL Plate 8	F4	50	0.565	0.0650	0.538	-5.7
Rxn #16	FTL Plate 8	G4	50	0.562	0.0650	0.538	-5.1
Rxn #16	FTL Plate 8	H4: FOC	-	0.535	0.0650	0.538	0.6
Rxn #16	FTL Plate 8	A5: NFC	-	0.064	0.0650	0.538	100.2
Rxn #16	FTL Plate 8	B5: NFC	-	0.054	0.0650	0.538	102.3
Rxn #16	FTL Plate 8	C5: NFC	-	0.053	0.0650	0.538	102.5
Rxn #16	FTL Plate 8	D5: NFC	-	0.046	0.0650	0.538	104.0
Rxn #16	FTL Plate 8	E5: FOC	-	0.546	0.0650	0.538	-1.7
Rxn #16	FTL Plate 8	F5: FOC	-	0.571	0.0650	0.538	-7.0
Rxn #16	FTL Plate 8	G5: FOC	-	0.535	0.0650	0.538	0.6
Rxn #16	FTL Plate 8	H5: FOC	-	0.550	0.0650	0.538	-2.5
Rxn #16	FTL Plate 8	A6: NFC	-	0.073	0.0650	0.538	98.3
Rxn #16	FTL Plate 8	B6	50	0.584	0.0650	0.538	-9.7
Rxn #16	FTL Plate 8	C6	50	0.548	0.0650	0.538	-2.1
Rxn #16	FTL Plate 8	D6	50	0.538	0.0650	0.538	0.0
Rxn #16	FTL Plate 8	E6	50	0.557	0.0650	0.538	-4.0
Rxn #16	FTL Plate 8	F6	50	0.519	0.0650	0.538	4.0
Rxn #16	FTL Plate 8	G6	50	0.591	0.0650	0.538	-11.2
Rxn #16	FTL Plate 8	H6: FOC	-	0.525	0.0650	0.538	2.7
Rxn #16	FTL Plate 8	A7: NFC	-	0.069	0.0650	0.538	99.2
Rxn #16	FTL Plate 8	B7	50	0.574	0.0650	0.538	-7.6
Rxn #16	FTL Plate 8	C7	50	0.571	0.0650	0.538	-7.0
Rxn #16	FTL Plate 8	D7	50	0.466	0.0650	0.538	15.2
Rxn #16	FTL Plate 8	E7	50	0.577	0.0650	0.538	-8.2
Rxn #16	FTL Plate 8	F7	50	0.546	0.0650	0.538	-1.7
Rxn #16	FTL Plate 8	G7	50	0.545	0.0650	0.538	-1.5
Rxn #16	FTL Plate 8	H7: FOC	-	0.529	0.0650	0.538	1.9
Rxn #16	FTL Plate 8	A8: NFC	-	0.070	0.0650	0.538	98.9
Rxn #16	FTL Plate 8	B8	50	0.520	0.0650	0.538	3.8
Rxn #16	FTL Plate 8	C8	50	0.549	0.0650	0.538	-2.3
Rxn #16	FTL Plate 8	D8	50	0.539	0.0650	0.538	-0.2
Rxn #16	FTL Plate 8	E8	50	0.550	0.0650	0.538	-2.5
Rxn #16	FTL Plate 8	F8	50	0.544	0.0650	0.538	-1.3
Rxn #16	FTL Plate 8	G8	50	0.553	0.0650	0.538	-3.2
Rxn #16	FTL Plate 8	H8: FOC	-	0.544	0.0650	0.538	-1.3
Rxn #16	FTL Plate 8	A9: NFC	-	0.068	0.0650	0.538	99.4
Rxn #16	FTL Plate 8	B9	50	0.370	0.0650	0.538	35.5
Rxn #16	FTL Plate 8	C9	50	0.535	0.0650	0.538	0.6
Rxn #16	FTL Plate 8	D9	50	0.536	0.0650	0.538	0.4
Rxn #16	FTL Plate 8	E9	50	0.483	0.0650	0.538	11.6
Rxn #16	FTL Plate 8	F9	50	0.529	0.0650	0.538	1.9
Rxn #16	FTL Plate 8	G9	50	0.519	0.0650	0.538	4.0
Rxn #16	FTL Plate 8	H9: FOC	-	0.529	0.0650	0.538	1.9
Rxn #16	FTL Plate 8	A10: NFC	-	0.062	0.0650	0.538	100.6
Rxn #16	FTL Plate 8	B10	50	0.567	0.0650	0.538	-6.1
Rxn #16	FTL Plate 8	C10	50	0.559	0.0650	0.538	-4.4
Rxn #16	FTL Plate 8	D10	50	0.521	0.0650	0.538	3.6
Rxn #16	FTL Plate 8	E10	50	0.555	0.0650	0.538	-3.6
Rxn #16	FTL Plate 8	F10	50	0.527	0.0650	0.538	2.3
Rxn #16	FTL Plate 8	G10	50	0.531	0.0650	0.538	1.5
Rxn #16	FTL Plate 8	H10: FOC	-	0.528	0.0650	0.538	2.1
Rxn #16	FTL Plate 8	A11: NFC	-	0.063	0.0650	0.538	100.4
Rxn #16	FTL Plate 8	B11	50	0.552	0.0650	0.538	-3.0
Rxn #16	FTL Plate 8	C11	50	0.284	0.0650	0.538	53.7
Rxn #16	FTL Plate 8	D11	50	0.537	0.0650	0.538	0.2
Rxn #16	FTL Plate 8	E11	50	0.528	0.0650	0.538	2.1
Rxn #16	FTL Plate 8	F11	50	0.504	0.0650	0.538	7.2
Rxn #16	FTL Plate 8	G11	50	0.531	0.0650	0.538	1.5
Rxn #16	FTL Plate 8	H11: FOC	-	0.536	0.0650	0.538	0.4
Rxn #16	FTL Plate 8	A12: NFC	-	0.080	0.0650	0.538	96.8
Rxn #16	FTL Plate 8	H12: FOC	-	0.538	0.0650	0.538	0.0

S1 Table. Data from Screening of Botanical Extract Library for Inhibition of KHKC Activity.

Rxn ID	Sample			Sample	Ave. No Fructose Controls	Ave. Fructose Only Controls	KHKC
	Botanical Plate	Well	Conc (μg/mL)	(ΔOD_{340nm})	(ΔOD_{340nm})	(ΔOD_{340nm})	Inhibition (%)
Rxn #17	FTL Plate 23	A1: NFC	-	0.085	0.0783	0.560	98.6
Rxn #17	FTL Plate 23	B1	50	0.500	0.0783	0.560	12.5
Rxn #17	FTL Plate 23	C1	50	0.484	0.0783	0.560	15.8
Rxn #17	FTL Plate 23	D1	50	0.513	0.0783	0.560	9.8
Rxn #17	FTL Plate 23	E1	50	0.502	0.0783	0.560	12.0
Rxn #17	FTL Plate 23	F1	50	0.424	0.0783	0.560	28.2
Rxn #17	FTL Plate 23	G1	50	0.525	0.0783	0.560	7.3
Rxn #17	FTL Plate 23	H1: FOC	-	0.559	0.0783	0.560	0.2
Rxn #17	FTL Plate 23	A2: NFC	-	0.086	0.0783	0.560	98.4
Rxn #17	FTL Plate 23	B2	50	0.523	0.0783	0.560	7.7
Rxn #17	FTL Plate 23	C2	50	0.534	0.0783	0.560	5.4
Rxn #17	FTL Plate 23	D2	50	0.469	0.0783	0.560	18.9
Rxn #17	FTL Plate 23	E2	50	0.445	0.0783	0.560	23.9
Rxn #17	FTL Plate 23	F2	50	0.516	0.0783	0.560	9.1
Rxn #17	FTL Plate 23	G2	50	0.502	0.0783	0.560	12.0
Rxn #17	FTL Plate 23	H2: FOC	-	0.574	0.0783	0.560	-2.9
Rxn #17	FTL Plate 23	A3: NFC	-	0.087	0.0783	0.560	98.2
Rxn #17	FTL Plate 23	B3	50	0.553	0.0783	0.560	1.5
Rxn #17	FTL Plate 23	C3	50	0.506	0.0783	0.560	11.2
Rxn #17	FTL Plate 23	D3	50	0.547	0.0783	0.560	2.7
Rxn #17	FTL Plate 23	E3	50	0.536	0.0783	0.560	5.0
Rxn #17	FTL Plate 23	F3	50	0.525	0.0783	0.560	7.3
Rxn #17	FTL Plate 23	G3	50	0.648	0.0783	0.560	-18.3
Rxn #17	FTL Plate 23	H3: FOC	-	0.575	0.0783	0.560	-3.1
Rxn #17	FTL Plate 23	A4: NFC	-	0.093	0.0783	0.560	96.9
Rxn #17	FTL Plate 23	B4	50	0.575	0.0783	0.560	-3.1
Rxn #17	FTL Plate 23	C4	50	0.544	0.0783	0.560	3.3
Rxn #17	FTL Plate 23	D4	50	0.548	0.0783	0.560	2.5
Rxn #17	FTL Plate 23	E4	50	0.556	0.0783	0.560	0.8
Rxn #17	FTL Plate 23	F4	50	0.562	0.0783	0.560	-0.4
Rxn #17	FTL Plate 23	G4	50	0.568	0.0783	0.560	-1.7
Rxn #17	FTL Plate 23	H4: FOC	-	0.567	0.0783	0.560	-1.5
Rxn #17	FTL Plate 23	A5: NFC	-	0.091	0.0783	0.560	97.4
Rxn #17	FTL Plate 23	B5: NFC	-	0.075	0.0783	0.560	100.7
Rxn #17	FTL Plate 23	C5: NFC	-	0.076	0.0783	0.560	100.5
Rxn #17	FTL Plate 23	D5: NFC	-	0.073	0.0783	0.560	101.1
Rxn #17	FTL Plate 23	E5: FOC	-	0.561	0.0783	0.560	-0.2
Rxn #17	FTL Plate 23	F5: FOC	-	0.556	0.0783	0.560	0.8
Rxn #17	FTL Plate 23	G5: FOC	-	0.551	0.0783	0.560	1.9
Rxn #17	FTL Plate 23	H5: FOC	-	0.542	0.0783	0.560	3.7
Rxn #17	FTL Plate 23	A6: NFC	-	0.083	0.0783	0.560	99.0
Rxn #17	FTL Plate 23	B6	50	0.554	0.0783	0.560	1.2
Rxn #17	FTL Plate 23	C6	50	0.394	0.0783	0.560	34.5
Rxn #17	FTL Plate 23	D6	50	0.560	0.0783	0.560	0.0
Rxn #17	FTL Plate 23	E6	50	0.537	0.0783	0.560	4.8
Rxn #17	FTL Plate 23	F6	50	0.551	0.0783	0.560	1.9
Rxn #17	FTL Plate 23	G6	50	0.566	0.0783	0.560	-1.2
Rxn #17	FTL Plate 23	H6: FOC	-	0.568	0.0783	0.560	-1.7
Rxn #17	FTL Plate 23	A7: NFC	-	0.089	0.0783	0.560	97.8
Rxn #17	FTL Plate 23	B7	50	0.458	0.0783	0.560	21.2
Rxn #17	FTL Plate 23	C7	50	0.551	0.0783	0.560	1.9
Rxn #17	FTL Plate 23	D7	50	0.549	0.0783	0.560	2.3
Rxn #17	FTL Plate 23	E7	50	0.545	0.0783	0.560	3.1
Rxn #17	FTL Plate 23	F7	50	0.530	0.0783	0.560	6.2
Rxn #17	FTL Plate 23	G7	50	0.524	0.0783	0.560	7.5
Rxn #17	FTL Plate 23	H7: FOC	-	0.569	0.0783	0.560	-1.9
Rxn #17	FTL Plate 23	A8: NFC	-	0.080	0.0783	0.560	99.6
Rxn #17	FTL Plate 23	B8	50	0.549	0.0783	0.560	2.3
Rxn #17	FTL Plate 23	C8	50	0.638	0.0783	0.560	-16.2
Rxn #17	FTL Plate 23	D8	50	0.449	0.0783	0.560	23.0
Rxn #17	FTL Plate 23	E8	50	0.548	0.0783	0.560	2.5
Rxn #17	FTL Plate 23	F8	50	0.529	0.0783	0.560	6.4
Rxn #17	FTL Plate 23	G8	50	0.543	0.0783	0.560	3.5
Rxn #17	FTL Plate 23	H8: FOC	-	0.549	0.0783	0.560	2.3
Rxn #17	FTL Plate 23	A9: NFC	-	0.072	0.0783	0.560	101.3
Rxn #17	FTL Plate 23	B9	50	0.536	0.0783	0.560	5.0
Rxn #17	FTL Plate 23	C9	50	0.418	0.0783	0.560	29.5
Rxn #17	FTL Plate 23	D9	50	0.439	0.0783	0.560	25.1
Rxn #17	FTL Plate 23	E9	50	0.473	0.0783	0.560	18.1
Rxn #17	FTL Plate 23	F9	50	0.384	0.0783	0.560	36.5

S1 Table. Data from Screening of Botanical Extract Library for Inhibition of KHKC Activity.

Rxn ID	Sample			Sample	Ave. No Fructose Controls	Ave. Fructose Only Controls	KHKC
	Botanical Plate	Well	Conc (μg/mL)	(ΔOD_{340nm})	(ΔOD_{340nm})	(ΔOD_{340nm})	Inhibition (%)
Rxn #17	FTL Plate 23	G9	50	0.424	0.0783	0.560	28.2
Rxn #17	FTL Plate 23	H9: FOC	-	0.552	0.0783	0.560	1.7
Rxn #17	FTL Plate 23	A10: NFC	-	0.050	0.0783	0.560	105.9
Rxn #17	FTL Plate 23	B10	50	0.430	0.0783	0.560	27.0
Rxn #17	FTL Plate 23	C10	50	0.454	0.0783	0.560	22.0
Rxn #17	FTL Plate 23	D10	50	0.480	0.0783	0.560	16.6
Rxn #17	FTL Plate 23	E10	50	0.384	0.0783	0.560	36.5
Rxn #17	FTL Plate 23	F10	50	0.424	0.0783	0.560	28.2
Rxn #17	FTL Plate 23	G10	50	0.361	0.0783	0.560	41.3
Rxn #17	FTL Plate 23	H10: FOC	-	0.580	0.0783	0.560	-4.2
Rxn #17	FTL Plate 23	A11: NFC	-	0.075	0.0783	0.560	100.7
Rxn #17	FTL Plate 23	B11	50	0.409	0.0783	0.560	31.3
Rxn #17	FTL Plate 23	C11	50	0.524	0.0783	0.560	7.5
Rxn #17	FTL Plate 23	D11	50	0.485	0.0783	0.560	15.6
Rxn #17	FTL Plate 23	E11	50	0.394	0.0783	0.560	34.5
Rxn #17	FTL Plate 23	F11	50	0.408	0.0783	0.560	31.6
Rxn #17	FTL Plate 23	G11	50	0.472	0.0783	0.560	18.3
Rxn #17	FTL Plate 23	H11: FOC	-	0.530	0.0783	0.560	6.2
Rxn #17	FTL Plate 23	A12: NFC	-	0.060	0.0783	0.560	103.8
Rxn #17	FTL Plate 23	H12: FOC	-	0.567	0.0783	0.560	-1.5
Rxn #18	FTL Plate 24	A1: NFC	-	0.075	0.0731	0.509	99.6
Rxn #18	FTL Plate 24	B1	50	0.464	0.0731	0.509	10.3
Rxn #18	FTL Plate 24	C1	50	0.481	0.0731	0.509	6.4
Rxn #18	FTL Plate 24	D1	50	0.429	0.0731	0.509	18.4
Rxn #18	FTL Plate 24	E1	50	0.474	0.0731	0.509	8.0
Rxn #18	FTL Plate 24	F1	50	0.471	0.0731	0.509	8.7
Rxn #18	FTL Plate 24	G1	50	0.430	0.0731	0.509	18.1
Rxn #18	FTL Plate 24	H1: FOC	-	0.507	0.0731	0.509	0.5
Rxn #18	FTL Plate 24	A2: NFC	-	0.077	0.0731	0.509	99.1
Rxn #18	FTL Plate 24	B2	50	0.502	0.0731	0.509	1.6
Rxn #18	FTL Plate 24	C2	50	0.446	0.0731	0.509	14.5
Rxn #18	FTL Plate 24	D2	50	0.338	0.0731	0.509	39.2
Rxn #18	FTL Plate 24	E2	50	0.519	0.0731	0.509	-2.3
Rxn #18	FTL Plate 24	F2	50	0.460	0.0731	0.509	11.2
Rxn #18	FTL Plate 24	G2	50	0.472	0.0731	0.509	8.5
Rxn #18	FTL Plate 24	H2: FOC	-	0.501	0.0731	0.509	1.8
Rxn #18	FTL Plate 24	A3: NFC	-	0.071	0.0731	0.509	100.5
Rxn #18	FTL Plate 24	B3	50	0.437	0.0731	0.509	16.5
Rxn #18	FTL Plate 24	C3	50	0.422	0.0731	0.509	20.0
Rxn #18	FTL Plate 24	D3	50	0.482	0.0731	0.509	6.2
Rxn #18	FTL Plate 24	E3	50	0.381	0.0731	0.509	29.4
Rxn #18	FTL Plate 24	F3	50	0.477	0.0731	0.509	7.3
Rxn #18	FTL Plate 24	G3	50	0.496	0.0731	0.509	3.0
Rxn #18	FTL Plate 24	H3: FOC	-	0.509	0.0731	0.509	0.0
Rxn #18	FTL Plate 24	A4: NFC	-	0.074	0.0731	0.509	99.8
Rxn #18	FTL Plate 24	B4	50	0.512	0.0731	0.509	-0.7
Rxn #18	FTL Plate 24	C4	50	0.516	0.0731	0.509	-1.6
Rxn #18	FTL Plate 24	D4	50	0.481	0.0731	0.509	6.4
Rxn #18	FTL Plate 24	E4	50	0.444	0.0731	0.509	14.9
Rxn #18	FTL Plate 24	F4	50	0.492	0.0731	0.509	3.9
Rxn #18	FTL Plate 24	G4	50	0.499	0.0731	0.509	2.3
Rxn #18	FTL Plate 24	H4: FOC	-	0.507	0.0731	0.509	0.5
Rxn #18	FTL Plate 24	A5: NFC	-	0.075	0.0731	0.509	99.6
Rxn #18	FTL Plate 24	B5: NFC	-	0.061	0.0731	0.509	102.8
Rxn #18	FTL Plate 24	C5: NFC	-	0.066	0.0731	0.509	101.6
Rxn #18	FTL Plate 24	D5: NFC	-	0.055	0.0731	0.509	104.2
Rxn #18	FTL Plate 24	E5: FOC	-	0.496	0.0731	0.509	3.0
Rxn #18	FTL Plate 24	F5: FOC	-	0.503	0.0731	0.509	1.4
Rxn #18	FTL Plate 24	G5: FOC	-	0.507	0.0731	0.509	0.5
Rxn #18	FTL Plate 24	H5: FOC	-	0.507	0.0731	0.509	0.5
Rxn #18	FTL Plate 24	A6: NFC	-	0.065	0.0731	0.509	101.9
Rxn #18	FTL Plate 24	B6	50	0.515	0.0731	0.509	-1.4
Rxn #18	FTL Plate 24	C6	50	0.371	0.0731	0.509	31.7
Rxn #18	FTL Plate 24	D6	50	0.508	0.0731	0.509	0.2
Rxn #18	FTL Plate 24	E6	50	0.488	0.0731	0.509	4.8
Rxn #18	FTL Plate 24	F6	50	0.510	0.0731	0.509	-0.2
Rxn #18	FTL Plate 24	G6	50	0.504	0.0731	0.509	1.1
Rxn #18	FTL Plate 24	H6: FOC	-	0.513	0.0731	0.509	-0.9
Rxn #18	FTL Plate 24	A7: NFC	-	0.072	0.0731	0.509	100.3
Rxn #18	FTL Plate 24	B7	50	0.491	0.0731	0.509	4.1

S1 Table. Data from Screening of Botanical Extract Library for Inhibition of KHKC Activity.

Rxn ID	Sample			Sample	Ave. No Fructose Controls	Ave. Fructose Only Controls	KHKC
	Botanical Plate	Well	Conc (μg/mL)	(ΔOD_{340nm})	(ΔOD_{340nm})	(ΔOD_{340nm})	Inhibition (%)
Rxn #18	FTL Plate 24	C7	50	0.514	0.0731	0.509	-1.1
Rxn #18	FTL Plate 24	D7	50	0.452	0.0731	0.509	13.1
Rxn #18	FTL Plate 24	E7	50	0.485	0.0731	0.509	5.5
Rxn #18	FTL Plate 24	F7	50	0.503	0.0731	0.509	1.4
Rxn #18	FTL Plate 24	G7	50	0.410	0.0731	0.509	22.7
Rxn #18	FTL Plate 24	H7: FOC	-	0.525	0.0731	0.509	-3.7
Rxn #18	FTL Plate 24	A8: NFC	-	0.080	0.0731	0.509	98.4
Rxn #18	FTL Plate 24	B8	50	0.484	0.0731	0.509	5.7
Rxn #18	FTL Plate 24	C8	50	0.509	0.0731	0.509	0.0
Rxn #18	FTL Plate 24	D8	50	0.457	0.0731	0.509	11.9
Rxn #18	FTL Plate 24	E8	50	0.470	0.0731	0.509	8.9
Rxn #18	FTL Plate 24	F8	50	0.496	0.0731	0.509	3.0
Rxn #18	FTL Plate 24	G8	50	0.523	0.0731	0.509	-3.2
Rxn #18	FTL Plate 24	H8: FOC	-	0.514	0.0731	0.509	-1.1
Rxn #18	FTL Plate 24	A9: NFC	-	0.077	0.0731	0.509	99.1
Rxn #18	FTL Plate 24	B9	50	0.505	0.0731	0.509	0.9
Rxn #18	FTL Plate 24	C9	50	0.482	0.0731	0.509	6.2
Rxn #18	FTL Plate 24	D9	50	0.480	0.0731	0.509	6.7
Rxn #18	FTL Plate 24	E9	50	0.506	0.0731	0.509	0.7
Rxn #18	FTL Plate 24	F9	50	0.447	0.0731	0.509	14.2
Rxn #18	FTL Plate 24	G9	50	0.511	0.0731	0.509	-0.5
Rxn #18	FTL Plate 24	H9: FOC	-	0.512	0.0731	0.509	-0.7
Rxn #18	FTL Plate 24	A10: NFC	-	0.076	0.0731	0.509	99.3
Rxn #18	FTL Plate 24	B10	50	0.495	0.0731	0.509	3.2
Rxn #18	FTL Plate 24	C10	50	0.470	0.0731	0.509	8.9
Rxn #18	FTL Plate 24	D10	50	0.484	0.0731	0.509	5.7
Rxn #18	FTL Plate 24	E10	50	0.460	0.0731	0.509	11.2
Rxn #18	FTL Plate 24	F10	50	0.467	0.0731	0.509	9.6
Rxn #18	FTL Plate 24	G10	50	0.473	0.0731	0.509	8.3
Rxn #18	FTL Plate 24	H10: FOC	-	0.519	0.0731	0.509	-2.3
Rxn #18	FTL Plate 24	A11: NFC	-	0.084	0.0731	0.509	97.5
Rxn #18	FTL Plate 24	B11	50	0.487	0.0731	0.509	5.0
Rxn #18	FTL Plate 24	C11	50	0.476	0.0731	0.509	7.6
Rxn #18	FTL Plate 24	D11	50	0.518	0.0731	0.509	-2.1
Rxn #18	FTL Plate 24	E11	50	0.499	0.0731	0.509	2.3
Rxn #18	FTL Plate 24	F11	50	0.505	0.0731	0.509	0.9
Rxn #18	FTL Plate 24	G11	50	0.456	0.0731	0.509	12.2
Rxn #18	FTL Plate 24	H11: FOC	-	0.509	0.0731	0.509	0.0
Rxn #18	FTL Plate 24	A12: NFC	-	0.089	0.0731	0.509	96.4
Rxn #18	FTL Plate 24	H12: FOC	-	0.506	0.0731	0.509	0.7
Rxn #19	FTL Plate 9	A1: NFC	-	0.079	0.0699	0.549	98.1
Rxn #19	FTL Plate 9	B1	50	0.528	0.0699	0.549	4.4
Rxn #19	FTL Plate 9	C1	50	0.532	0.0699	0.549	3.5
Rxn #19	FTL Plate 9	D1	50	0.492	0.0699	0.549	11.9
Rxn #19	FTL Plate 9	E1	50	0.444	0.0699	0.549	21.9
Rxn #19	FTL Plate 9	F1	50	0.502	0.0699	0.549	9.8
Rxn #19	FTL Plate 9	G1	50	0.522	0.0699	0.549	5.6
Rxn #19	FTL Plate 9	H1: FOC	-	0.536	0.0699	0.549	2.7
Rxn #19	FTL Plate 9	A2: NFC	-	0.071	0.0699	0.549	99.8
Rxn #19	FTL Plate 9	B2	50	0.534	0.0699	0.549	3.1
Rxn #19	FTL Plate 9	C2	50	0.516	0.0699	0.549	6.9
Rxn #19	FTL Plate 9	D2	50	0.572	0.0699	0.549	-4.8
Rxn #19	FTL Plate 9	E2	50	0.545	0.0699	0.549	0.8
Rxn #19	FTL Plate 9	F2	50	0.543	0.0699	0.549	1.3
Rxn #19	FTL Plate 9	G2	50	0.548	0.0699	0.549	0.2
Rxn #19	FTL Plate 9	H2: FOC	-	0.568	0.0699	0.549	-4.0
Rxn #19	FTL Plate 9	A3: NFC	-	0.075	0.0699	0.549	98.9
Rxn #19	FTL Plate 9	B3	50	0.575	0.0699	0.549	-5.4
Rxn #19	FTL Plate 9	C3	50	0.504	0.0699	0.549	9.4
Rxn #19	FTL Plate 9	D3	50	0.559	0.0699	0.549	-2.1
Rxn #19	FTL Plate 9	E3	50	0.550	0.0699	0.549	-0.2
Rxn #19	FTL Plate 9	F3	50	0.549	0.0699	0.549	0.0
Rxn #19	FTL Plate 9	G3	50	0.514	0.0699	0.549	7.3
Rxn #19	FTL Plate 9	H3: FOC	-	0.548	0.0699	0.549	0.2
Rxn #19	FTL Plate 9	A4: NFC	-	0.070	0.0699	0.549	100.0
Rxn #19	FTL Plate 9	B4	50	0.530	0.0699	0.549	4.0
Rxn #19	FTL Plate 9	C4	50	0.537	0.0699	0.549	2.5
Rxn #19	FTL Plate 9	D4	50	0.505	0.0699	0.549	9.2
Rxn #19	FTL Plate 9	E4	50	0.523	0.0699	0.549	5.4
Rxn #19	FTL Plate 9	F4	50	0.524	0.0699	0.549	5.2

S1 Table. Data from Screening of Botanical Extract Library for Inhibition of KHKC Activity.

Rxn ID	Sample			Sample	Ave. No Fructose Controls	Ave. Fructose Only Controls	KHKC
	Botanical Plate	Well	Conc (μg/mL)	(ΔOD_{340nm})	(ΔOD_{340nm})	(ΔOD_{340nm})	Inhibition (%)
Rxn #19	FTL Plate 9	G4	50	0.526	0.0699	0.549	4.8
Rxn #19	FTL Plate 9	H4: FOC	-	0.541	0.0699	0.549	1.7
Rxn #19	FTL Plate 9	A5: NFC	-	0.070	0.0699	0.549	100.0
Rxn #19	FTL Plate 9	B5: NFC	-	0.056	0.0699	0.549	102.9
Rxn #19	FTL Plate 9	C5: NFC	-	0.051	0.0699	0.549	103.9
Rxn #19	FTL Plate 9	D5: NFC	-	0.056	0.0699	0.549	102.9
Rxn #19	FTL Plate 9	E5: FOC	-	0.542	0.0699	0.549	1.5
Rxn #19	FTL Plate 9	F5: FOC	-	0.536	0.0699	0.549	2.7
Rxn #19	FTL Plate 9	G5: FOC	-	0.539	0.0699	0.549	2.1
Rxn #19	FTL Plate 9	H5: FOC	-	0.545	0.0699	0.549	0.8
Rxn #19	FTL Plate 9	A6: NFC	-	0.072	0.0699	0.549	99.6
Rxn #19	FTL Plate 9	B6	50	0.465	0.0699	0.549	17.5
Rxn #19	FTL Plate 9	C6	50	0.526	0.0699	0.549	4.8
Rxn #19	FTL Plate 9	D6	50	0.540	0.0699	0.549	1.9
Rxn #19	FTL Plate 9	E6	50	0.509	0.0699	0.549	8.3
Rxn #19	FTL Plate 9	F6	50	0.513	0.0699	0.549	7.5
Rxn #19	FTL Plate 9	G6	50	0.511	0.0699	0.549	7.9
Rxn #19	FTL Plate 9	H6: FOC	-	0.571	0.0699	0.549	-4.6
Rxn #19	FTL Plate 9	A7: NFC	-	0.073	0.0699	0.549	99.4
Rxn #19	FTL Plate 9	B7	50	0.558	0.0699	0.549	-1.9
Rxn #19	FTL Plate 9	C7	50	0.529	0.0699	0.549	4.2
Rxn #19	FTL Plate 9	D7	50	0.502	0.0699	0.549	9.8
Rxn #19	FTL Plate 9	E7	50	0.528	0.0699	0.549	4.4
Rxn #19	FTL Plate 9	F7	50	0.524	0.0699	0.549	5.2
Rxn #19	FTL Plate 9	G7	50	0.536	0.0699	0.549	2.7
Rxn #19	FTL Plate 9	H7: FOC	-	0.560	0.0699	0.549	-2.3
Rxn #19	FTL Plate 9	A8: NFC	-	0.070	0.0699	0.549	100.0
Rxn #19	FTL Plate 9	B8	50	0.541	0.0699	0.549	1.7
Rxn #19	FTL Plate 9	C8	50	0.473	0.0699	0.549	15.9
Rxn #19	FTL Plate 9	D8	50	0.534	0.0699	0.549	3.1
Rxn #19	FTL Plate 9	E8	50	0.523	0.0699	0.549	5.4
Rxn #19	FTL Plate 9	F8	50	0.539	0.0699	0.549	2.1
Rxn #19	FTL Plate 9	G8	50	0.490	0.0699	0.549	12.3
Rxn #19	FTL Plate 9	H8: FOC	-	0.565	0.0699	0.549	-3.3
Rxn #19	FTL Plate 9	A9: NFC	-	0.070	0.0699	0.549	100.0
Rxn #19	FTL Plate 9	B9	50	0.540	0.0699	0.549	1.9
Rxn #19	FTL Plate 9	C9	50	0.558	0.0699	0.549	-1.9
Rxn #19	FTL Plate 9	D9	50	0.554	0.0699	0.549	-1.0
Rxn #19	FTL Plate 9	E9	50	0.485	0.0699	0.549	13.4
Rxn #19	FTL Plate 9	F9	50	0.562	0.0699	0.549	-2.7
Rxn #19	FTL Plate 9	G9	50	0.551	0.0699	0.549	-0.4
Rxn #19	FTL Plate 9	H9: FOC	-	0.549	0.0699	0.549	0.0
Rxn #19	FTL Plate 9	A10: NFC	-	0.083	0.0699	0.549	97.3
Rxn #19	FTL Plate 9	B10	50	0.554	0.0699	0.549	-1.0
Rxn #19	FTL Plate 9	C10	50	0.530	0.0699	0.549	4.0
Rxn #19	FTL Plate 9	D10	50	0.542	0.0699	0.549	1.5
Rxn #19	FTL Plate 9	E10	50	0.145	0.0699	0.549	84.3
Rxn #19	FTL Plate 9	F10	50	0.470	0.0699	0.549	16.5
Rxn #19	FTL Plate 9	G10	50	0.562	0.0699	0.549	-2.7
Rxn #19	FTL Plate 9	H10: FOC	-	0.556	0.0699	0.549	-1.5
Rxn #19	FTL Plate 9	A11: NFC	-	0.072	0.0699	0.549	99.6
Rxn #19	FTL Plate 9	B11	50	0.526	0.0699	0.549	4.8
Rxn #19	FTL Plate 9	C11	50	0.518	0.0699	0.549	6.5
Rxn #19	FTL Plate 9	D11	50	0.550	0.0699	0.549	-0.2
Rxn #19	FTL Plate 9	E11	50	0.514	0.0699	0.549	7.3
Rxn #19	FTL Plate 9	F11	50	0.509	0.0699	0.549	8.3
Rxn #19	FTL Plate 9	G11	50	0.128	0.0699	0.549	87.9
Rxn #19	FTL Plate 9	H11: FOC	-	0.559	0.0699	0.549	-2.1
Rxn #19	FTL Plate 9	A12: NFC	-	0.080	0.0699	0.549	97.9
Rxn #19	FTL Plate 9	H12: FOC	-	0.514	0.0699	0.549	7.3
Rxn #20	FTL Plate 11	A1: NFC	-	0.070	0.0689	0.580	99.8
Rxn #20	FTL Plate 11	B1	50	0.543	0.0689	0.580	7.2
Rxn #20	FTL Plate 11	C1	50	0.503	0.0689	0.580	15.1
Rxn #20	FTL Plate 11	D1	50	0.529	0.0689	0.580	10.0
Rxn #20	FTL Plate 11	E1	50	0.537	0.0689	0.580	8.4
Rxn #20	FTL Plate 11	F1	50	0.557	0.0689	0.580	4.5
Rxn #20	FTL Plate 11	G1	50	0.471	0.0689	0.580	21.3
Rxn #20	FTL Plate 11	H1: FOC	-	0.601	0.0689	0.580	-4.1
Rxn #20	FTL Plate 11	A2: NFC	-	0.079	0.0689	0.580	98.0
Rxn #20	FTL Plate 11	B2	50	0.508	0.0689	0.580	14.1

S1 Table. Data from Screening of Botanical Extract Library for Inhibition of KHKC Activity.

Rxn ID	Sample			Sample	Ave. No Fructose Controls	Ave. Fructose Only Controls	KHKC
	Botanical Plate	Well	Conc (μg/mL)	(ΔOD_{340nm})	(ΔOD_{340nm})	(ΔOD_{340nm})	Inhibition (%)
Rxn #20	FTL Plate 11	C2	50	0.505	0.0689	0.580	14.7
Rxn #20	FTL Plate 11	D2	50	0.542	0.0689	0.580	7.4
Rxn #20	FTL Plate 11	E2	50	0.577	0.0689	0.580	0.6
Rxn #20	FTL Plate 11	F2	50	0.472	0.0689	0.580	21.1
Rxn #20	FTL Plate 11	G2	50	0.550	0.0689	0.580	5.9
Rxn #20	FTL Plate 11	H2: FOC	-	0.590	0.0689	0.580	-2.0
Rxn #20	FTL Plate 11	A3: NFC	-	0.074	0.0689	0.580	99.0
Rxn #20	FTL Plate 11	B3	50	0.556	0.0689	0.580	4.7
Rxn #20	FTL Plate 11	C3	50	0.571	0.0689	0.580	1.8
Rxn #20	FTL Plate 11	D3	50	0.491	0.0689	0.580	17.4
Rxn #20	FTL Plate 11	E3	50	0.505	0.0689	0.580	14.7
Rxn #20	FTL Plate 11	F3	50	0.559	0.0689	0.580	4.1
Rxn #20	FTL Plate 11	G3	50	0.568	0.0689	0.580	2.3
Rxn #20	FTL Plate 11	H3: FOC	-	0.573	0.0689	0.580	1.4
Rxn #20	FTL Plate 11	A4: NFC	-	0.067	0.0689	0.580	100.4
Rxn #20	FTL Plate 11	B4	50	0.558	0.0689	0.580	4.3
Rxn #20	FTL Plate 11	C4	50	0.565	0.0689	0.580	2.9
Rxn #20	FTL Plate 11	D4	50	0.541	0.0689	0.580	7.6
Rxn #20	FTL Plate 11	E4	50	0.582	0.0689	0.580	-0.4
Rxn #20	FTL Plate 11	F4	50	0.503	0.0689	0.580	15.1
Rxn #20	FTL Plate 11	G4	50	0.583	0.0689	0.580	-0.6
Rxn #20	FTL Plate 11	H4: FOC	-	0.581	0.0689	0.580	-0.2
Rxn #20	FTL Plate 11	A5: NFC	-	0.071	0.0689	0.580	99.6
Rxn #20	FTL Plate 11	B5: NFC	-	0.054	0.0689	0.580	102.9
Rxn #20	FTL Plate 11	C5: NFC	-	0.062	0.0689	0.580	101.4
Rxn #20	FTL Plate 11	D5: NFC	-	0.056	0.0689	0.580	102.5
Rxn #20	FTL Plate 11	E5: FOC	-	0.557	0.0689	0.580	4.5
Rxn #20	FTL Plate 11	F5: FOC	-	0.573	0.0689	0.580	1.4
Rxn #20	FTL Plate 11	G5: FOC	-	0.580	0.0689	0.580	0.0
Rxn #20	FTL Plate 11	H5: FOC	-	0.580	0.0689	0.580	0.0
Rxn #20	FTL Plate 11	A6: NFC	-	0.075	0.0689	0.580	98.8
Rxn #20	FTL Plate 11	B6	50	0.468	0.0689	0.580	21.9
Rxn #20	FTL Plate 11	C6	50	0.540	0.0689	0.580	7.8
Rxn #20	FTL Plate 11	D6	50	0.562	0.0689	0.580	3.5
Rxn #20	FTL Plate 11	E6	50	0.564	0.0689	0.580	3.1
Rxn #20	FTL Plate 11	F6	50	0.542	0.0689	0.580	7.4
Rxn #20	FTL Plate 11	G6	50	0.551	0.0689	0.580	5.7
Rxn #20	FTL Plate 11	H6: FOC	-	0.582	0.0689	0.580	-0.4
Rxn #20	FTL Plate 11	A7: NFC	-	0.071	0.0689	0.580	99.6
Rxn #20	FTL Plate 11	B7	50	0.538	0.0689	0.580	8.2
Rxn #20	FTL Plate 11	C7	50	0.560	0.0689	0.580	3.9
Rxn #20	FTL Plate 11	D7	50	0.510	0.0689	0.580	13.7
Rxn #20	FTL Plate 11	E7	50	0.526	0.0689	0.580	10.6
Rxn #20	FTL Plate 11	F7	50	0.545	0.0689	0.580	6.8
Rxn #20	FTL Plate 11	G7	50	0.561	0.0689	0.580	3.7
Rxn #20	FTL Plate 11	H7: FOC	-	0.567	0.0689	0.580	2.5
Rxn #20	FTL Plate 11	A8: NFC	-	0.062	0.0689	0.580	101.4
Rxn #20	FTL Plate 11	B8	50	0.563	0.0689	0.580	3.3
Rxn #20	FTL Plate 11	C8	50	0.494	0.0689	0.580	16.8
Rxn #20	FTL Plate 11	D8	50	0.564	0.0689	0.580	3.1
Rxn #20	FTL Plate 11	E8	50	0.531	0.0689	0.580	9.6
Rxn #20	FTL Plate 11	F8	50	0.565	0.0689	0.580	2.9
Rxn #20	FTL Plate 11	G8	50	0.546	0.0689	0.580	6.7
Rxn #20	FTL Plate 11	H8: FOC	-	0.585	0.0689	0.580	-1.0
Rxn #20	FTL Plate 11	A9: NFC	-	0.067	0.0689	0.580	100.4
Rxn #20	FTL Plate 11	B9	50	0.548	0.0689	0.580	6.3
Rxn #20	FTL Plate 11	C9	50	0.562	0.0689	0.580	3.5
Rxn #20	FTL Plate 11	D9	50	0.572	0.0689	0.580	1.6
Rxn #20	FTL Plate 11	E9	50	0.572	0.0689	0.580	1.6
Rxn #20	FTL Plate 11	F9	50	0.558	0.0689	0.580	4.3
Rxn #20	FTL Plate 11	G9	50	0.565	0.0689	0.580	2.9
Rxn #20	FTL Plate 11	H9: FOC	-	0.575	0.0689	0.580	1.0
Rxn #20	FTL Plate 11	A10: NFC	-	0.075	0.0689	0.580	98.8
Rxn #20	FTL Plate 11	B10	50	0.577	0.0689	0.580	0.6
Rxn #20	FTL Plate 11	C10	50	0.530	0.0689	0.580	9.8
Rxn #20	FTL Plate 11	D10	50	0.529	0.0689	0.580	10.0
Rxn #20	FTL Plate 11	E10	50	0.583	0.0689	0.580	-0.6
Rxn #20	FTL Plate 11	F10	50	0.577	0.0689	0.580	0.6
Rxn #20	FTL Plate 11	G10	50	0.567	0.0689	0.580	2.5
Rxn #20	FTL Plate 11	H10: FOC	-	0.579	0.0689	0.580	0.2

S1 Table. Data from Screening of Botanical Extract Library for Inhibition of KHKC Activity.

Rxn ID	Sample			Sample	Ave. No Fructose Controls	Ave. Fructose Only Controls	KHKC
	Botanical Plate	Well	Conc (µg/mL)	(ΔOD_{340nm})	(ΔOD_{340nm})	(ΔOD_{340nm})	Inhibition (%)
Rxn #20	FTL Plate 11	A11: NFC	-	0.076	0.0689	0.580	98.6
Rxn #20	FTL Plate 11	B11	50	0.563	0.0689	0.580	3.3
Rxn #20	FTL Plate 11	C11	50	0.544	0.0689	0.580	7.0
Rxn #20	FTL Plate 11	D11	50	0.565	0.0689	0.580	2.9
Rxn #20	FTL Plate 11	E11	50	0.543	0.0689	0.580	7.2
Rxn #20	FTL Plate 11	F11	50	0.524	0.0689	0.580	11.0
Rxn #20	FTL Plate 11	G11	50	0.583	0.0689	0.580	-0.6
Rxn #20	FTL Plate 11	H11: FOC	-	0.589	0.0689	0.580	-1.8
Rxn #20	FTL Plate 11	A12: NFC	-	0.074	0.0689	0.580	99.0
Rxn #20	FTL Plate 11	H12: FOC	-	0.582	0.0689	0.580	-0.4
Rxn #21	FTL Plate 12	A1: NFC	-	0.053	0.0535	0.516	100.1
Rxn #21	FTL Plate 12	B1	50	0.504	0.0535	0.516	2.6
Rxn #21	FTL Plate 12	C1	50	0.431	0.0535	0.516	18.4
Rxn #21	FTL Plate 12	D1	50	0.494	0.0535	0.516	4.8
Rxn #21	FTL Plate 12	E1	50	0.522	0.0535	0.516	-1.3
Rxn #21	FTL Plate 12	F1	50	0.504	0.0535	0.516	2.6
Rxn #21	FTL Plate 12	G1	50	0.471	0.0535	0.516	9.7
Rxn #21	FTL Plate 12	H1: FOC	-	0.491	0.0535	0.516	5.4
Rxn #21	FTL Plate 12	A2: NFC	-	0.053	0.0535	0.516	100.1
Rxn #21	FTL Plate 12	B2	50	0.447	0.0535	0.516	14.9
Rxn #21	FTL Plate 12	C2	50	0.523	0.0535	0.516	-1.5
Rxn #21	FTL Plate 12	D2	50	0.497	0.0535	0.516	4.1
Rxn #21	FTL Plate 12	E2	50	0.518	0.0535	0.516	-0.4
Rxn #21	FTL Plate 12	F2	50	0.460	0.0535	0.516	12.1
Rxn #21	FTL Plate 12	G2	50	0.509	0.0535	0.516	1.5
Rxn #21	FTL Plate 12	H2: FOC	-	0.509	0.0535	0.516	1.5
Rxn #21	FTL Plate 12	A3: NFC	-	0.059	0.0535	0.516	98.8
Rxn #21	FTL Plate 12	B3	50	0.509	0.0535	0.516	1.5
Rxn #21	FTL Plate 12	C3	50	0.530	0.0535	0.516	-3.0
Rxn #21	FTL Plate 12	D3	50	0.478	0.0535	0.516	8.2
Rxn #21	FTL Plate 12	E3	50	0.498	0.0535	0.516	3.9
Rxn #21	FTL Plate 12	F3	50	0.512	0.0535	0.516	0.9
Rxn #21	FTL Plate 12	G3	50	0.539	0.0535	0.516	-5.0
Rxn #21	FTL Plate 12	H3: FOC	-	0.521	0.0535	0.516	-1.1
Rxn #21	FTL Plate 12	A4: NFC	-	0.053	0.0535	0.516	100.1
Rxn #21	FTL Plate 12	B4	50	0.505	0.0535	0.516	2.4
Rxn #21	FTL Plate 12	C4	50	0.512	0.0535	0.516	0.9
Rxn #21	FTL Plate 12	D4	50	0.503	0.0535	0.516	2.8
Rxn #21	FTL Plate 12	E4	50	0.430	0.0535	0.516	18.6
Rxn #21	FTL Plate 12	F4	50	0.498	0.0535	0.516	3.9
Rxn #21	FTL Plate 12	G4	50	0.490	0.0535	0.516	5.6
Rxn #21	FTL Plate 12	H4: FOC	-	0.527	0.0535	0.516	-2.4
Rxn #21	FTL Plate 12	A5: NFC	-	0.054	0.0535	0.516	99.9
Rxn #21	FTL Plate 12	B5: NFC	-	0.042	0.0535	0.516	102.5
Rxn #21	FTL Plate 12	C5: NFC	-	0.045	0.0535	0.516	101.8
Rxn #21	FTL Plate 12	D5: NFC	-	0.036	0.0535	0.516	103.8
Rxn #21	FTL Plate 12	E5: FOC	-	0.517	0.0535	0.516	-0.2
Rxn #21	FTL Plate 12	F5: FOC	-	0.496	0.0535	0.516	4.3
Rxn #21	FTL Plate 12	G5: FOC	-	0.525	0.0535	0.516	-1.9
Rxn #21	FTL Plate 12	H5: FOC	-	0.510	0.0535	0.516	1.3
Rxn #21	FTL Plate 12	A6: NFC	-	0.054	0.0535	0.516	99.9
Rxn #21	FTL Plate 12	B6	50	0.505	0.0535	0.516	2.4
Rxn #21	FTL Plate 12	C6	50	0.473	0.0535	0.516	9.3
Rxn #21	FTL Plate 12	D6	50	0.496	0.0535	0.516	4.3
Rxn #21	FTL Plate 12	E6	50	0.509	0.0535	0.516	1.5
Rxn #21	FTL Plate 12	F6	50	0.460	0.0535	0.516	12.1
Rxn #21	FTL Plate 12	G6	50	0.445	0.0535	0.516	15.4
Rxn #21	FTL Plate 12	H6: FOC	-	0.504	0.0535	0.516	2.6
Rxn #21	FTL Plate 12	A7: NFC	-	0.054	0.0535	0.516	99.9
Rxn #21	FTL Plate 12	B7	50	0.502	0.0535	0.516	3.0
Rxn #21	FTL Plate 12	C7	50	0.483	0.0535	0.516	7.1
Rxn #21	FTL Plate 12	D7	50	0.507	0.0535	0.516	1.9
Rxn #21	FTL Plate 12	E7	50	0.470	0.0535	0.516	9.9
Rxn #21	FTL Plate 12	F7	50	0.542	0.0535	0.516	-5.6
Rxn #21	FTL Plate 12	G7	50	0.517	0.0535	0.516	-0.2
Rxn #21	FTL Plate 12	H7: FOC	-	0.542	0.0535	0.516	-5.6
Rxn #21	FTL Plate 12	A8: NFC	-	0.057	0.0535	0.516	99.2
Rxn #21	FTL Plate 12	B8	50	0.508	0.0535	0.516	1.7
Rxn #21	FTL Plate 12	C8	50	0.498	0.0535	0.516	3.9
Rxn #21	FTL Plate 12	D8	50	0.507	0.0535	0.516	1.9

S1 Table. Data from Screening of Botanical Extract Library for Inhibition of KHKC Activity.

Rxn ID	Sample			Sample	Ave. No Fructose Controls	Ave. Fructose Only Controls	KHKC
	Botanical Plate	Well	Conc (μg/mL)	(ΔOD_{340nm})	(ΔOD_{340nm})	(ΔOD_{340nm})	Inhibition (%)
Rxn #21	FTL Plate 12	E8	50	0.409	0.0535	0.516	23.1
Rxn #21	FTL Plate 12	F8	50	0.502	0.0535	0.516	3.0
Rxn #21	FTL Plate 12	G8	50	0.463	0.0535	0.516	11.5
Rxn #21	FTL Plate 12	H8: FOC	-	0.527	0.0535	0.516	-2.4
Rxn #21	FTL Plate 12	A9: NFC	-	0.068	0.0535	0.516	96.9
Rxn #21	FTL Plate 12	B9	50	0.481	0.0535	0.516	7.6
Rxn #21	FTL Plate 12	C9	50	0.505	0.0535	0.516	2.4
Rxn #21	FTL Plate 12	D9	50	0.483	0.0535	0.516	7.1
Rxn #21	FTL Plate 12	E9	50	0.470	0.0535	0.516	9.9
Rxn #21	FTL Plate 12	F9	50	0.509	0.0535	0.516	1.5
Rxn #21	FTL Plate 12	G9	50	0.481	0.0535	0.516	7.6
Rxn #21	FTL Plate 12	H9: FOC	-	0.520	0.0535	0.516	-0.9
Rxn #21	FTL Plate 12	A10: NFC	-	0.059	0.0535	0.516	98.8
Rxn #21	FTL Plate 12	B10	50	0.485	0.0535	0.516	6.7
Rxn #21	FTL Plate 12	C10	50	0.513	0.0535	0.516	0.6
Rxn #21	FTL Plate 12	D10	50	0.516	0.0535	0.516	0.0
Rxn #21	FTL Plate 12	E10	50	0.489	0.0535	0.516	5.8
Rxn #21	FTL Plate 12	F10	50	0.481	0.0535	0.516	7.6
Rxn #21	FTL Plate 12	G10	50	0.519	0.0535	0.516	-0.6
Rxn #21	FTL Plate 12	H10: FOC	-	0.539	0.0535	0.516	-5.0
Rxn #21	FTL Plate 12	A11: NFC	-	0.059	0.0535	0.516	98.8
Rxn #21	FTL Plate 12	B11	50	0.494	0.0535	0.516	4.8
Rxn #21	FTL Plate 12	C11	50	0.515	0.0535	0.516	0.2
Rxn #21	FTL Plate 12	D11	50	0.481	0.0535	0.516	7.6
Rxn #21	FTL Plate 12	E11	50	0.524	0.0535	0.516	-1.7
Rxn #21	FTL Plate 12	F11	50	0.489	0.0535	0.516	5.8
Rxn #21	FTL Plate 12	G11	50	0.490	0.0535	0.516	5.6
Rxn #21	FTL Plate 12	H11: FOC	-	0.525	0.0535	0.516	-1.9
Rxn #21	FTL Plate 12	A12: NFC	-	0.056	0.0535	0.516	99.5
Rxn #21	FTL Plate 12	H12: FOC	-	0.494	0.0535	0.516	4.8
Rxn #22	FTL Plate 26	A1: NFC	-	0.063	0.0611	0.524	99.6
Rxn #22	FTL Plate 26	B1	50	0.467	0.0611	0.524	12.3
Rxn #22	FTL Plate 26	C1	50	0.472	0.0611	0.524	11.2
Rxn #22	FTL Plate 26	D1	50	0.518	0.0611	0.524	1.3
Rxn #22	FTL Plate 26	E1	50	0.488	0.0611	0.524	7.8
Rxn #22	FTL Plate 26	F1	50	0.502	0.0611	0.524	4.8
Rxn #22	FTL Plate 26	G1	50	0.464	0.0611	0.524	13.0
Rxn #22	FTL Plate 26	H1: FOC	-	0.521	0.0611	0.524	0.6
Rxn #22	FTL Plate 26	A2: NFC	-	0.062	0.0611	0.524	99.8
Rxn #22	FTL Plate 26	B2	50	0.513	0.0611	0.524	2.4
Rxn #22	FTL Plate 26	C2	50	0.531	0.0611	0.524	-1.5
Rxn #22	FTL Plate 26	D2	50	0.497	0.0611	0.524	5.8
Rxn #22	FTL Plate 26	E2	50	0.484	0.0611	0.524	8.6
Rxn #22	FTL Plate 26	F2	50	0.484	0.0611	0.524	8.6
Rxn #22	FTL Plate 26	G2	50	0.417	0.0611	0.524	23.1
Rxn #22	FTL Plate 26	H2: FOC	-	0.521	0.0611	0.524	0.6
Rxn #22	FTL Plate 26	A3: NFC	-	0.061	0.0611	0.524	100.0
Rxn #22	FTL Plate 26	B3	50	0.519	0.0611	0.524	1.1
Rxn #22	FTL Plate 26	C3	50	0.526	0.0611	0.524	-0.4
Rxn #22	FTL Plate 26	D3	50	0.518	0.0611	0.524	1.3
Rxn #22	FTL Plate 26	E3	50	0.488	0.0611	0.524	7.8
Rxn #22	FTL Plate 26	F3	50	0.493	0.0611	0.524	6.7
Rxn #22	FTL Plate 26	G3	50	0.503	0.0611	0.524	4.5
Rxn #22	FTL Plate 26	H3: FOC	-	0.520	0.0611	0.524	0.9
Rxn #22	FTL Plate 26	A4: NFC	-	0.061	0.0611	0.524	100.0
Rxn #22	FTL Plate 26	B4	50	0.238	0.0611	0.524	61.8
Rxn #22	FTL Plate 26	C4	50	0.455	0.0611	0.524	14.9
Rxn #22	FTL Plate 26	D4	50	0.165	0.0611	0.524	77.6
Rxn #22	FTL Plate 26	E4	50	0.482	0.0611	0.524	9.1
Rxn #22	FTL Plate 26	F4	50	0.286	0.0611	0.524	51.4
Rxn #22	FTL Plate 26	G4	50	0.444	0.0611	0.524	17.3
Rxn #22	FTL Plate 26	H4: FOC	-	0.540	0.0611	0.524	-3.5
Rxn #22	FTL Plate 26	A5: NFC	-	0.059	0.0611	0.524	100.5
Rxn #22	FTL Plate 26	B5: NFC	-	0.061	0.0611	0.524	100.0
Rxn #22	FTL Plate 26	C5: NFC	-	0.047	0.0611	0.524	103.0
Rxn #22	FTL Plate 26	D5: NFC	-	0.053	0.0611	0.524	101.7
Rxn #22	FTL Plate 26	E5: FOC	-	0.515	0.0611	0.524	1.9
Rxn #22	FTL Plate 26	F5: FOC	-	0.497	0.0611	0.524	5.8
Rxn #22	FTL Plate 26	G5: FOC	-	0.514	0.0611	0.524	2.2
Rxn #22	FTL Plate 26	H5: FOC	-	0.513	0.0611	0.524	2.4

S1 Table. Data from Screening of Botanical Extract Library for Inhibition of KHKC Activity.

Rxn ID	Sample			Sample	Ave. No Fructose Controls	Ave. Fructose Only Controls	KHKC
	Botanical Plate	Well	Conc (μg/mL)	(ΔOD_{340nm})	(ΔOD_{340nm})	(ΔOD_{340nm})	Inhibition (%)
Rxn #22	FTL Plate 26	A6: NFC	-	0.062	0.0611	0.524	99.8
Rxn #22	FTL Plate 26	B6	50	0.539	0.0611	0.524	-3.2
Rxn #22	FTL Plate 26	C6	50	0.495	0.0611	0.524	6.3
Rxn #22	FTL Plate 26	D6	50	0.472	0.0611	0.524	11.2
Rxn #22	FTL Plate 26	E6	50	0.487	0.0611	0.524	8.0
Rxn #22	FTL Plate 26	F6	50	0.519	0.0611	0.524	1.1
Rxn #22	FTL Plate 26	G6	50	0.532	0.0611	0.524	-1.7
Rxn #22	FTL Plate 26	H6: FOC	-	0.531	0.0611	0.524	-1.5
Rxn #22	FTL Plate 26	A7: NFC	-	0.068	0.0611	0.524	98.5
Rxn #22	FTL Plate 26	B7	50	0.510	0.0611	0.524	3.0
Rxn #22	FTL Plate 26	C7	50	0.477	0.0611	0.524	10.2
Rxn #22	FTL Plate 26	D7	50	0.520	0.0611	0.524	0.9
Rxn #22	FTL Plate 26	E7	50	0.523	0.0611	0.524	0.2
Rxn #22	FTL Plate 26	F7	50	0.518	0.0611	0.524	1.3
Rxn #22	FTL Plate 26	G7	50	0.474	0.0611	0.524	10.8
Rxn #22	FTL Plate 26	H7: FOC	-	0.543	0.0611	0.524	-4.1
Rxn #22	FTL Plate 26	A8: NFC	-	0.063	0.0611	0.524	99.6
Rxn #22	FTL Plate 26	B8	50	0.446	0.0611	0.524	16.9
Rxn #22	FTL Plate 26	C8	50	0.533	0.0611	0.524	-1.9
Rxn #22	FTL Plate 26	D8	50	0.503	0.0611	0.524	4.5
Rxn #22	FTL Plate 26	E8	50	0.518	0.0611	0.524	1.3
Rxn #22	FTL Plate 26	F8	50	0.451	0.0611	0.524	15.8
Rxn #22	FTL Plate 26	G8	50	0.548	0.0611	0.524	-5.2
Rxn #22	FTL Plate 26	H8: FOC	-	0.538	0.0611	0.524	-3.0
Rxn #22	FTL Plate 26	A9: NFC	-	0.062	0.0611	0.524	99.8
Rxn #22	FTL Plate 26	B9	50	0.422	0.0611	0.524	22.0
Rxn #22	FTL Plate 26	C9	50	0.496	0.0611	0.524	6.0
Rxn #22	FTL Plate 26	D9	50	0.496	0.0611	0.524	6.0
Rxn #22	FTL Plate 26	E9	50	0.476	0.0611	0.524	10.4
Rxn #22	FTL Plate 26	F9	50	0.522	0.0611	0.524	0.4
Rxn #22	FTL Plate 26	G9	50	0.526	0.0611	0.524	-0.4
Rxn #22	FTL Plate 26	H9: FOC	-	0.526	0.0611	0.524	-0.4
Rxn #22	FTL Plate 26	A10: NFC	-	0.059	0.0611	0.524	100.5
Rxn #22	FTL Plate 26	B10	50	0.485	0.0611	0.524	8.4
Rxn #22	FTL Plate 26	C10	50	0.501	0.0611	0.524	5.0
Rxn #22	FTL Plate 26	D10	50	0.511	0.0611	0.524	2.8
Rxn #22	FTL Plate 26	E10	50	0.468	0.0611	0.524	12.1
Rxn #22	FTL Plate 26	F10	50	0.505	0.0611	0.524	4.1
Rxn #22	FTL Plate 26	G10	50	0.501	0.0611	0.524	5.0
Rxn #22	FTL Plate 26	H10: FOC	-	0.538	0.0611	0.524	-3.0
Rxn #22	FTL Plate 26	A11: NFC	-	0.067	0.0611	0.524	98.7
Rxn #22	FTL Plate 26	B11	50	0.523	0.0611	0.524	0.2
Rxn #22	FTL Plate 26	C11	50	0.416	0.0611	0.524	23.3
Rxn #22	FTL Plate 26	D11	50	0.495	0.0611	0.524	6.3
Rxn #22	FTL Plate 26	E11	50	0.505	0.0611	0.524	4.1
Rxn #22	FTL Plate 26	F11	50	0.497	0.0611	0.524	5.8
Rxn #22	FTL Plate 26	G11	50	0.504	0.0611	0.524	4.3
Rxn #22	FTL Plate 26	H11: FOC	-	0.528	0.0611	0.524	-0.9
Rxn #22	FTL Plate 26	A12: NFC	-	0.068	0.0611	0.524	98.5
Rxn #22	FTL Plate 26	H12: FOC	-	0.513	0.0611	0.524	2.4
Rxn #29	FTL Plate 27	A1: NFC	-	0.027	0.0258	0.459	99.7
Rxn #29	FTL Plate 27	B1	50	0.399	0.0258	0.459	13.9
Rxn #29	FTL Plate 27	C1	50	0.408	0.0258	0.459	11.8
Rxn #29	FTL Plate 27	D1	50	0.458	0.0258	0.459	0.2
Rxn #29	FTL Plate 27	E1	50	0.435	0.0258	0.459	5.5
Rxn #29	FTL Plate 27	F1	50	0.458	0.0258	0.459	0.2
Rxn #29	FTL Plate 27	G1	50	0.448	0.0258	0.459	2.5
Rxn #29	FTL Plate 27	A2: NFC	-	0.026	0.0258	0.459	100.0
Rxn #29	FTL Plate 27	B2	50	0.413	0.0258	0.459	10.6
Rxn #29	FTL Plate 27	C2	50	0.480	0.0258	0.459	-4.8
Rxn #29	FTL Plate 27	D2	50	0.474	0.0258	0.459	-3.5
Rxn #29	FTL Plate 27	E2	50	0.469	0.0258	0.459	-2.3
Rxn #29	FTL Plate 27	F2	50	0.482	0.0258	0.459	-5.3
Rxn #29	FTL Plate 27	G2	50	0.474	0.0258	0.459	-3.5
Rxn #29	FTL Plate 27	A3: NFC	-	0.028	0.0258	0.459	99.5
Rxn #29	FTL Plate 27	B3	50	0.454	0.0258	0.459	1.2
Rxn #29	FTL Plate 27	C3	50	0.444	0.0258	0.459	3.5
Rxn #29	FTL Plate 27	D3	50	0.425	0.0258	0.459	7.8
Rxn #29	FTL Plate 27	E3	50	0.478	0.0258	0.459	-4.4
Rxn #29	FTL Plate 27	F3	50	0.449	0.0258	0.459	2.3

S1 Table. Data from Screening of Botanical Extract Library for Inhibition of KHKC Activity.

Rxn ID	Sample			Sample	Ave. No Fructose Controls	Ave. Fructose Only Controls	KHKC
	Botanical Plate	Well	Conc (μg/mL)	(ΔOD_{340nm})	(ΔOD_{340nm})	(ΔOD_{340nm})	Inhibition (%)
Rxn #29	FTL Plate 27	G3	50	0.459	0.0258	0.459	0.0
Rxn #29	FTL Plate 27	A4: NFC	-	0.024	0.0258	0.459	100.4
Rxn #29	FTL Plate 27	B4	50	0.451	0.0258	0.459	1.8
Rxn #29	FTL Plate 27	C4	50	0.511	0.0258	0.459	-12.0
Rxn #29	FTL Plate 27	D4	50	0.453	0.0258	0.459	1.4
Rxn #29	FTL Plate 27	E4	50	0.475	0.0258	0.459	-3.7
Rxn #29	FTL Plate 27	F4	50	0.465	0.0258	0.459	-1.4
Rxn #29	FTL Plate 27	G4	50	0.486	0.0258	0.459	-6.2
Rxn #29	FTL Plate 27	A5: NFC	-	0.027	0.0258	0.459	99.7
Rxn #29	FTL Plate 27	B5: NFC	-	0.019	0.0258	0.459	101.6
Rxn #29	FTL Plate 27	C5: NFC	-	0.019	0.0258	0.459	101.6
Rxn #29	FTL Plate 27	D5: NFC	-	0.024	0.0258	0.459	100.4
Rxn #29	FTL Plate 27	E5: FOC	-	0.469	0.0258	0.459	-2.3
Rxn #29	FTL Plate 27	F5: FOC	-	0.460	0.0258	0.459	-0.2
Rxn #29	FTL Plate 27	G5: FOC	-	0.454	0.0258	0.459	1.2
Rxn #29	FTL Plate 27	A6: NFC	-	0.032	0.0258	0.459	98.6
Rxn #29	FTL Plate 27	B6	50	0.438	0.0258	0.459	4.8
Rxn #29	FTL Plate 27	C6	50	0.466	0.0258	0.459	-1.6
Rxn #29	FTL Plate 27	D6	50	0.426	0.0258	0.459	7.6
Rxn #29	FTL Plate 27	E6	50	0.458	0.0258	0.459	0.2
Rxn #29	FTL Plate 27	F6	50	0.466	0.0258	0.459	-1.6
Rxn #29	FTL Plate 27	G6	50	0.486	0.0258	0.459	-6.2
Rxn #29	FTL Plate 27	A7: NFC	-	0.031	0.0258	0.459	98.8
Rxn #29	FTL Plate 27	B7	50	0.474	0.0258	0.459	-3.5
Rxn #29	FTL Plate 27	C7	50	0.432	0.0258	0.459	6.2
Rxn #29	FTL Plate 27	D7	50	0.462	0.0258	0.459	-0.7
Rxn #29	FTL Plate 27	E7	50	0.495	0.0258	0.459	-8.3
Rxn #29	FTL Plate 27	F7	50	0.479	0.0258	0.459	-4.6
Rxn #29	FTL Plate 27	G7	50	0.438	0.0258	0.459	4.8
Rxn #29	FTL Plate 27	B8	50	0.433	0.0258	0.459	6.0
Rxn #29	FTL Plate 27	C8	50	0.450	0.0258	0.459	2.1
Rxn #29	FTL Plate 27	D8	50	0.513	0.0258	0.459	-12.5
Rxn #29	FTL Plate 27	E8	50	0.470	0.0258	0.459	-2.5
Rxn #29	FTL Plate 27	F8	50	0.453	0.0258	0.459	1.4
Rxn #29	FTL Plate 27	G8	50	0.486	0.0258	0.459	-6.2
Rxn #29	FTL Plate 27	B9	50	0.454	0.0258	0.459	1.2
Rxn #29	FTL Plate 27	C9	50	0.436	0.0258	0.459	5.3
Rxn #29	FTL Plate 27	D9	50	0.461	0.0258	0.459	-0.5
Rxn #29	FTL Plate 27	E9	50	0.364	0.0258	0.459	21.9
Rxn #29	FTL Plate 27	F9	50	0.393	0.0258	0.459	15.2
Rxn #29	FTL Plate 27	G9	50	0.392	0.0258	0.459	15.5
Rxn #29	FTL Plate 27	A10: NFC	-	0.027	0.0258	0.459	99.7
Rxn #29	FTL Plate 27	B10	50	0.437	0.0258	0.459	5.1
Rxn #29	FTL Plate 27	C10	50	0.478	0.0258	0.459	-4.4
Rxn #29	FTL Plate 27	D10	50	0.518	0.0258	0.459	-13.6
Rxn #29	FTL Plate 27	E10	50	0.415	0.0258	0.459	10.2
Rxn #29	FTL Plate 27	F10	50	0.384	0.0258	0.459	17.3
Rxn #29	FTL Plate 27	G10	50	0.375	0.0258	0.459	19.4
Rxn #29	FTL Plate 27	H10: FOC	-	0.463	0.0258	0.459	-0.9
Rxn #29	FTL Plate 27	A11: NFC	-	0.020	0.0258	0.459	101.3
Rxn #29	FTL Plate 27	B11	50	0.406	0.0258	0.459	12.2
Rxn #29	FTL Plate 27	C11	50	0.460	0.0258	0.459	-0.2
Rxn #29	FTL Plate 27	D11	50	0.469	0.0258	0.459	-2.3
Rxn #29	FTL Plate 27	E11	50	0.423	0.0258	0.459	8.3
Rxn #29	FTL Plate 27	F11	50	0.371	0.0258	0.459	20.3
Rxn #29	FTL Plate 27	G11	50	0.437	0.0258	0.459	5.1
Rxn #29	FTL Plate 27	H11: FOC	-	0.448	0.0258	0.459	2.5
Rxn #29	FTL Plate 27	A12: NFC	-	0.031	0.0258	0.459	98.8

FTL: functional target library; proprietary library of Amway Corp. KHKC: ketohexokinase isoform C. FOC: fructose only control. NFC: no fructose control. OD: optical density.
