# Supplementary material for: Bioactivity-Guided Identification of Botanical Inhibitors of Ketohexokinase
Source: PLoS One. 2016 Jun 20;11(6):e0157458. doi: 10.1371/journal.pone.0157458 (PMC4913896; doi:10.1371/journal.pone.0157458)
Supplement: S2 Table — FTL: functional target library; proprietary library of Amway Corp. IC50: half maximal inhibitory concentration. OD: optical density. *KHKC IC50s were calculated using nonlinear regression (three parameters) in GraphPad Prism 5.03. To generate a best fit, an upper concentration (10,000 μg/mL at 100% inhibition) and a lower concentration (0.001 μg/mL at 0% inhibition) were added. (PDF) [file pone.0157458.s002.pdf]

S2 Table. Data from Titrations of Candidate Botanical Extracts for Inhibition of KHKC Activity.

Rxn		Sample			Sample	Ave. No Fructose Controls	Ave. Fructose Only Controls	KHKC	*KHKC IC_{50}
ID	Well	Botanical Plate	Well	Conc (μg/mL)	(ΔOD_{340nm})	(ΔOD_{340nm})	(ΔOD_{340nm})	Inhibition (%)	(μg/mL)
Rxn #23	A1	FTL Plate 15	B9	200	0.066	0.0772	0.422	103.2	32.15
Rxn #23	A2	FTL Plate 15	B9	150	0.117	0.0772	0.422	88.5	
Rxn #23	A3	FTL Plate 15	B9	100	0.029	0.0772	0.422	114.0	
Rxn #23	A4	FTL Plate 15	B9	50	0.074	0.0772	0.422	100.9	
Rxn #23	A5	FTL Plate 15	B9	25	0.378	0.0772	0.422	12.8	
Rxn #23	A6	FTL Plate 15	B9	10	0.441	0.0772	0.422	-5.5	
Rxn #23	A7	FTL Plate 15	B9	5	0.444	0.0772	0.422	-6.4	
Rxn #23	A8	FTL Plate 15	B9	1	0.462	0.0772	0.422	-11.6	
Rxn #23	A9	FTL Plate 15	B9	0.5	0.462	0.0772	0.422	-11.6	
Rxn #23	A10	FTL Plate 15	B9	0.1	0.454	0.0772	0.422	-9.3	
Rxn #23	G1	FTL Plate 26	F4	200	0.142	0.0772	0.422	81.2	39.24
Rxn #23	G2	FTL Plate 26	F4	150	0.156	0.0772	0.422	77.1	
Rxn #23	G3	FTL Plate 26	F4	100	0.182	0.0772	0.422	69.6	
Rxn #23	G4	FTL Plate 26	F4	50	0.222	0.0772	0.422	58.0	
Rxn #23	G5	FTL Plate 26	F4	25	0.323	0.0772	0.422	28.7	
Rxn #23	G6	FTL Plate 26	F4	10	0.404	0.0772	0.422	5.2	
Rxn #23	G7	FTL Plate 26	F4	5	0.421	0.0772	0.422	0.3	
Rxn #23	G8	FTL Plate 26	F4	1	0.476	0.0772	0.422	-15.7	
Rxn #23	G9	FTL Plate 26	F4	0.5	0.475	0.0772	0.422	-15.4	
Rxn #23	G10	FTL Plate 26	F4	0.1	0.471	0.0772	0.422	-14.2	
Rxn #23	F1	FTL Plate 26	D4	200	0.114	0.0772	0.422	89.3	23.22
Rxn #23	F2	FTL Plate 26	D4	150	0.115	0.0772	0.422	89.0	
Rxn #23	F3	FTL Plate 26	D4	100	0.136	0.0772	0.422	82.9	
Rxn #23	F4	FTL Plate 26	D4	50	0.179	0.0772	0.422	70.5	
Rxn #23	F5	FTL Plate 26	D4	25	0.265	0.0772	0.422	45.5	
Rxn #23	F6	FTL Plate 26	D4	10	0.344	0.0772	0.422	22.6	
Rxn #23	F7	FTL Plate 26	D4	5	0.394	0.0772	0.422	8.1	
Rxn #23	F8	FTL Plate 26	D4	1	0.451	0.0772	0.422	-8.4	
Rxn #23	F9	FTL Plate 26	D4	0.5	0.458	0.0772	0.422	-10.4	
Rxn #23	F10	FTL Plate 26	D4	0.1	0.474	0.0772	0.422	-15.1	
Rxn #23	E1	FTL Plate 26	B4	200	0.133	0.0772	0.422	83.8	22.56
Rxn #23	E2	FTL Plate 26	B4	150	0.119	0.0772	0.422	87.9	
Rxn #23	E3	FTL Plate 26	B4	100	0.141	0.0772	0.422	81.5	
Rxn #23	E4	FTL Plate 26	B4	50	0.181	0.0772	0.422	69.9	
Rxn #23	E5	FTL Plate 26	B4	25	0.248	0.0772	0.422	50.5	
Rxn #23	E6	FTL Plate 26	B4	10	0.345	0.0772	0.422	22.3	
Rxn #23	E7	FTL Plate 26	B4	5	0.391	0.0772	0.422	9.0	
Rxn #23	E8	FTL Plate 26	B4	1	0.446	0.0772	0.422	-7.0	
Rxn #23	E9	FTL Plate 26	B4	0.5	0.455	0.0772	0.422	-9.6	
Rxn #23	E10	FTL Plate 26	B4	0.1	0.448	0.0772	0.422	-7.5	
Rxn #23	C1	FTL Plate 3	D2	200	0.115	0.0772	0.422	89.0	30.41
Rxn #23	C2	FTL Plate 3	D2	150	0.136	0.0772	0.422	82.9	
Rxn #23	C3	FTL Plate 3	D2	100	0.156	0.0772	0.422	77.1	
Rxn #23	C4	FTL Plate 3	D2	50	0.200	0.0772	0.422	64.4	
Rxn #23	C5	FTL Plate 3	D2	25	0.289	0.0772	0.422	38.6	
Rxn #23	C6	FTL Plate 3	D2	10	0.360	0.0772	0.422	18.0	
Rxn #23	C7	FTL Plate 3	D2	5	0.408	0.0772	0.422	4.1	
Rxn #23	C8	FTL Plate 3	D2	1	0.454	0.0772	0.422	-9.3	
Rxn #23	C9	FTL Plate 3	D2	0.5	0.450	0.0772	0.422	-8.1	
Rxn #23	C10	FTL Plate 3	D2	0.1	0.461	0.0772	0.422	-11.3	
Rxn #23	D1	FTL Plate 3	C3	200	0.140	0.0772	0.422	81.8	30.31
Rxn #23	D2	FTL Plate 3	C3	150	0.146	0.0772	0.422	80.0	
Rxn #23	D3	FTL Plate 3	C3	100	0.159	0.0772	0.422	76.3	
Rxn #23	D4	FTL Plate 3	C3	50	0.200	0.0772	0.422	64.4	
Rxn #23	D5	FTL Plate 3	C3	25	0.291	0.0772	0.422	38.0	
Rxn #23	D6	FTL Plate 3	C3	10	0.373	0.0772	0.422	14.2	
Rxn #23	D7	FTL Plate 3	C3	5	0.403	0.0772	0.422	5.5	
Rxn #23	D8	FTL Plate 3	C3	1	0.459	0.0772	0.422	-10.7	
Rxn #23	D9	FTL Plate 3	C3	0.5	0.466	0.0772	0.422	-12.8	
Rxn #23	D10	FTL Plate 3	C3	0.1	0.455	0.0772	0.422	-9.6	
Rxn #23	B1	FTL Plate 3	B1	200	0.166	0.0772	0.422	74.2	57.27
Rxn #23	B2	FTL Plate 3	B1	150	0.178	0.0772	0.422	70.8	
Rxn #23	B3	FTL Plate 3	B1	100	0.208	0.0772	0.422	62.1	
Rxn #23	B4	FTL Plate 3	B1	50	0.265	0.0772	0.422	45.5	
Rxn #23	B5	FTL Plate 3	B1	25	0.345	0.0772	0.422	22.3	
Rxn #23	B6	FTL Plate 3	B1	10	0.414	0.0772	0.422	2.3	
Rxn #23	B7	FTL Plate 3	B1	5	0.419	0.0772	0.422	0.9	
Rxn #23	B8	FTL Plate 3	B1	1	0.463	0.0772	0.422	-11.9	
Rxn #23	B9	FTL Plate 3	B1	0.5	0.458	0.0772	0.422	-10.4	
Rxn #23	B10	FTL Plate 3	B1	0.1	0.458	0.0772	0.422	-10.4	
Rxn #23	H1	FTL Plate 8	C11	200	0.159	0.0772	0.422	76.3	50.85
Rxn #23	H2	FTL Plate 8	C11	150	0.177	0.0772	0.422	71.1	

S2 Table. Data from Titrations of Candidate Botanical Extracts for Inhibition of KHKC Activity.

Rxn		Sample			Sample	Ave. No Fructose Controls	Ave. Fructose Only Controls	KHKC	*KHKC IC_{50}
ID	Well	Botanical Plate	Well	Conc (μg/mL)	(ΔOD_{340nm})	(ΔOD_{340nm})	(ΔOD_{340nm})	Inhibition (%)	(μg/mL)
Rxn #23	H3	FTL Plate 8	C11	100	0.209	0.0772	0.422	61.8	
Rxn #23	H4	FTL Plate 8	C11	50	0.258	0.0772	0.422	47.6	
Rxn #23	H5	FTL Plate 8	C11	25	0.316	0.0772	0.422	30.7	
Rxn #23	H6	FTL Plate 8	C11	10	0.428	0.0772	0.422	-1.7	
Rxn #23	H7	FTL Plate 8	C11	5	0.454	0.0772	0.422	-9.3	
Rxn #23	H8	FTL Plate 8	C11	1	0.478	0.0772	0.422	-16.2	
Rxn #23	H9	FTL Plate 8	C11	0.5	0.460	0.0772	0.422	-11.0	
Rxn #23	H10	FTL Plate 8	C11	0.1	0.458	0.0772	0.422	-10.4	
Rxn #23	D12	No Fructose Control			0.076	0.0772	0.422	100.3	
Rxn #23	E12	No Fructose Control			0.073	0.0772	0.422	101.2	
Rxn #23	F12	No Fructose Control			0.073	0.0772	0.422	101.2	
Rxn #23	G12	No Fructose Control			0.085	0.0772	0.422	97.7	
Rxn #23	H12	No Fructose Control			0.079	0.0772	0.422	99.5	
Rxn #23	A11	Fructose Only Control			0.428	0.0772	0.422	-1.7	
Rxn #23	H11	Fructose Only Control			0.465	0.0772	0.422	-12.5	
Rxn #23	A12	Fructose Only Control			0.407	0.0772	0.422	4.4	
Rxn #23	B12	Fructose Only Control			0.368	0.0772	0.422	15.7	
Rxn #23	C12	Fructose Only Control			0.442	0.0772	0.422	-5.8	
Rxn #25	H1	FTL Plate 23	F9	200	0.272	0.0624	0.550	57.0	129.50
Rxn #25	H2	FTL Plate 23	F9	150	0.312	0.0624	0.550	48.8	
Rxn #25	H3	FTL Plate 23	F9	100	0.321	0.0624	0.550	47.0	
Rxn #25	H4	FTL Plate 23	F9	50	0.412	0.0624	0.550	28.3	
Rxn #25	H5	FTL Plate 23	F9	25	0.443	0.0624	0.550	21.9	
Rxn #25	H6	FTL Plate 23	F9	10	0.497	0.0624	0.550	10.9	
Rxn #25	H7	FTL Plate 23	F9	5	0.539	0.0624	0.550	2.3	
Rxn #25	H8	FTL Plate 23	F9	1	0.538	0.0624	0.550	2.5	
Rxn #25	H9	FTL Plate 23	F9	0.5	0.574	0.0624	0.550	-4.9	
Rxn #25	H10	FTL Plate 23	F9	0.1	0.559	0.0624	0.550	-1.8	
Rxn #25	G1	FTL Plate 23	E10	200	0.321	0.0624	0.550	47.0	209.10
Rxn #25	G2	FTL Plate 23	E10	150	0.357	0.0624	0.550	39.6	
Rxn #25	G3	FTL Plate 23	E10	100	0.366	0.0624	0.550	37.7	
Rxn #25	G4	FTL Plate 23	E10	50	0.442	0.0624	0.550	22.1	
Rxn #25	G5	FTL Plate 23	E10	25	0.463	0.0624	0.550	17.8	
Rxn #25	G6	FTL Plate 23	E10	10	0.531	0.0624	0.550	3.9	
Rxn #25	G7	FTL Plate 23	E10	5	0.540	0.0624	0.550	2.1	
Rxn #25	G8	FTL Plate 23	E10	1	0.544	0.0624	0.550	1.2	
Rxn #25	G9	FTL Plate 23	E10	0.5	0.551	0.0624	0.550	-0.2	
Rxn #25	G10	FTL Plate 23	E10	0.1	0.540	0.0624	0.550	2.1	
Rxn #25	D1	FTL Plate 24	D2	200	0.221	0.0624	0.550	67.5	76.41
Rxn #25	D2	FTL Plate 24	D2	150	0.245	0.0624	0.550	62.6	
Rxn #25	D3	FTL Plate 24	D2	100	0.269	0.0624	0.550	57.6	
Rxn #25	D4	FTL Plate 24	D2	50	0.335	0.0624	0.550	44.1	
Rxn #25	D5	FTL Plate 24	D2	25	0.386	0.0624	0.550	33.6	
Rxn #25	D6	FTL Plate 24	D2	10	0.466	0.0624	0.550	17.2	
Rxn #25	D7	FTL Plate 24	D2	5	0.492	0.0624	0.550	11.9	
Rxn #25	D8	FTL Plate 24	D2	1	0.500	0.0624	0.550	10.3	
Rxn #25	D9	FTL Plate 24	D2	0.5	0.533	0.0624	0.550	3.5	
Rxn #25	D10	FTL Plate 24	D2	0.1	0.521	0.0624	0.550	5.9	
Rxn #25	E1	FTL Plate 24	C6	200	0.240	0.0624	0.550	63.6	120.90
Rxn #25	E2	FTL Plate 24	C6	150	0.301	0.0624	0.550	51.1	
Rxn #25	E3	FTL Plate 24	C6	100	0.323	0.0624	0.550	46.6	
Rxn #25	E4	FTL Plate 24	C6	50	0.374	0.0624	0.550	36.1	
Rxn #25	E5	FTL Plate 24	C6	25	0.423	0.0624	0.550	26.0	
Rxn #25	E6	FTL Plate 24	C6	10	0.493	0.0624	0.550	11.7	
Rxn #25	E7	FTL Plate 24	C6	5	0.496	0.0624	0.550	11.1	
Rxn #25	E8	FTL Plate 24	C6	1	0.513	0.0624	0.550	7.6	
Rxn #25	E9	FTL Plate 24	C6	0.5	0.546	0.0624	0.550	0.8	
Rxn #25	E10	FTL Plate 24	C6	0.1	0.534	0.0624	0.550	3.3	
Rxn #25	A1	FTL Plate 8	B9	200	0.234	0.0624	0.550	64.8	106.90
Rxn #25	A2	FTL Plate 8	B9	150	0.253	0.0624	0.550	60.9	
Rxn #25	A3	FTL Plate 8	B9	100	0.320	0.0624	0.550	47.2	
Rxn #25	A4	FTL Plate 8	B9	50	0.385	0.0624	0.550	33.8	
Rxn #25	A5	FTL Plate 8	B9	25	0.450	0.0624	0.550	20.5	
Rxn #25	A6	FTL Plate 8	B9	10	0.513	0.0624	0.550	7.6	
Rxn #25	A7	FTL Plate 8	B9	5	0.546	0.0624	0.550	0.8	
Rxn #25	A8	FTL Plate 8	B9	1	0.534	0.0624	0.550	3.3	
Rxn #25	A9	FTL Plate 8	B9	0.5	0.545	0.0624	0.550	1.0	
Rxn #25	A10	FTL Plate 8	B9	0.1	0.541	0.0624	0.550	1.8	
Rxn #25	C1	FTL Plate 9	G11	200	0.170	0.0624	0.550	77.9	8.92
Rxn #25	C2	FTL Plate 9	G11	150	0.125	0.0624	0.550	87.2	
Rxn #25	C3	FTL Plate 9	G11	100	0.075	0.0624	0.550	97.4	
Rxn #25	C4	FTL Plate 9	G11	50	0.134	0.0624	0.550	85.3	

S2 Table. Data from Titrations of Candidate Botanical Extracts for Inhibition of KHKC Activity.

Rxn ID	Rxn Well	Sample Botanical Plate	Sample Well	Sample Conc (μg/mL)	Sample (ΔOD_{340nm})	Ave. No Fructose Controls (ΔOD_{340nm})	Ave. Fructose Only Controls (ΔOD_{340nm})	KHKC Inhibition (%)	*KHKC IC_{50} (μg/mL)
Rxn #25	C5	FTL Plate 9	G11	25	0.211	0.0624	0.550	69.5	
Rxn #25	C6	FTL Plate 9	G11	10	0.308	0.0624	0.550	49.6	
Rxn #25	C7	FTL Plate 9	G11	5	0.390	0.0624	0.550	32.8	
Rxn #25	C8	FTL Plate 9	G11	1	0.497	0.0624	0.550	10.9	
Rxn #25	C9	FTL Plate 9	G11	0.5	0.526	0.0624	0.550	4.9	
Rxn #25	C10	FTL Plate 9	G11	0.1	0.548	0.0624	0.550	0.4	
Rxn #25	B1	FTL Plate 9	E10	200	0.153	0.0624	0.550	81.4	9.18
Rxn #25	B2	FTL Plate 9	E10	150	0.141	0.0624	0.550	83.9	
Rxn #25	B3	FTL Plate 9	E10	100	0.079	0.0624	0.550	96.6	
Rxn #25	B4	FTL Plate 9	E10	50	0.153	0.0624	0.550	81.4	
Rxn #25	B5	FTL Plate 9	E10	25	0.207	0.0624	0.550	70.3	
Rxn #25	B6	FTL Plate 9	E10	10	0.313	0.0624	0.550	48.6	
Rxn #25	B7	FTL Plate 9	E10	5	0.384	0.0624	0.550	34.0	
Rxn #25	B8	FTL Plate 9	E10	1	0.498	0.0624	0.550	10.7	
Rxn #25	B9	FTL Plate 9	E10	0.5	0.503	0.0624	0.550	9.6	
Rxn #25	B10	FTL Plate 9	E10	0.1	0.548	0.0624	0.550	0.4	
Rxn #25	D12	No Fructose Control			0.065	0.0624	0.550	99.5	
Rxn #25	E12	No Fructose Control			0.057	0.0624	0.550	101.1	
Rxn #25	F12	No Fructose Control			0.062	0.0624	0.550	100.1	
Rxn #25	G12	No Fructose Control			0.062	0.0624	0.550	100.1	
Rxn #25	H12	No Fructose Control			0.066	0.0624	0.550	99.3	
Rxn #25	A11	Fructose Only Control			0.542	0.0624	0.550	1.6	
Rxn #25	H11	Fructose Only Control			0.548	0.0624	0.550	0.4	
Rxn #25	A12	Fructose Only Control			0.553	0.0624	0.550	-0.6	
Rxn #25	B12	Fructose Only Control			0.556	0.0624	0.550	-1.2	
Rxn #25	C12	Fructose Only Control			0.551	0.0624	0.550	-0.2	
Rxn #27	A1	FTL Plate 23	G10	200	0.254	0.0660	0.514	58.0	126.50
Rxn #27	A2	FTL Plate 23	G10	150	0.279	0.0660	0.514	52.5	
Rxn #27	A3	FTL Plate 23	G10	100	0.314	0.0660	0.514	44.6	
Rxn #27	A4	FTL Plate 23	G10	50	0.369	0.0660	0.514	32.4	
Rxn #27	A5	FTL Plate 23	G10	25	0.431	0.0660	0.514	18.5	
Rxn #27	A6	FTL Plate 23	G10	10	0.489	0.0660	0.514	5.6	
Rxn #27	A7	FTL Plate 23	G10	5	0.487	0.0660	0.514	6.0	
Rxn #27	A8	FTL Plate 23	G10	1	0.508	0.0660	0.514	1.3	
Rxn #27	A9	FTL Plate 23	G10	0.5	0.529	0.0660	0.514	-3.3	
Rxn #27	A10	FTL Plate 23	G10	0.1	0.515	0.0660	0.514	-0.2	
Rxn #27	C1	FTL Plate 23	F11	200	0.265	0.0660	0.514	55.6	164.20
Rxn #27	C2	FTL Plate 23	F11	150	0.297	0.0660	0.514	48.4	
Rxn #27	C3	FTL Plate 23	F11	100	0.333	0.0660	0.514	40.4	
Rxn #27	C4	FTL Plate 23	F11	50	0.383	0.0660	0.514	29.2	
Rxn #27	C5	FTL Plate 23	F11	25	0.432	0.0660	0.514	18.3	
Rxn #27	C6	FTL Plate 23	F11	10	0.478	0.0660	0.514	8.0	
Rxn #27	C7	FTL Plate 23	F11	5	0.474	0.0660	0.514	8.9	
Rxn #27	C8	FTL Plate 23	F11	1	0.503	0.0660	0.514	2.5	
Rxn #27	C9	FTL Plate 23	F11	0.5	0.500	0.0660	0.514	3.1	
Rxn #27	C10	FTL Plate 23	F11	0.1	0.489	0.0660	0.514	5.6	
Rxn #27	B1	FTL Plate 23	B11	200	0.272	0.0660	0.514	54.0	170.30
Rxn #27	B2	FTL Plate 23	B11	150	0.299	0.0660	0.514	48.0	
Rxn #27	B3	FTL Plate 23	B11	100	0.339	0.0660	0.514	39.1	
Rxn #27	B4	FTL Plate 23	B11	50	0.408	0.0660	0.514	23.7	
Rxn #27	B5	FTL Plate 23	B11	25	0.444	0.0660	0.514	15.6	
Rxn #27	B6	FTL Plate 23	B11	10	0.481	0.0660	0.514	7.4	
Rxn #27	B7	FTL Plate 23	B11	5	0.498	0.0660	0.514	3.6	
Rxn #27	B8	FTL Plate 23	B11	1	0.507	0.0660	0.514	1.6	
Rxn #27	B9	FTL Plate 23	B11	0.5	0.509	0.0660	0.514	1.1	
Rxn #27	B10	FTL Plate 23	B11	0.1	0.506	0.0660	0.514	1.8	
Rxn #27	F1	FTL Plate 4	G9	200	0.258	0.0660	0.514	57.1	141.40
Rxn #27	F2	FTL Plate 4	G9	150	0.283	0.0660	0.514	51.6	
Rxn #27	F3	FTL Plate 4	G9	100	0.329	0.0660	0.514	41.3	
Rxn #27	F4	FTL Plate 4	G9	50	0.371	0.0660	0.514	31.9	
Rxn #27	F5	FTL Plate 4	G9	25	0.424	0.0660	0.514	20.1	
Rxn #27	F6	FTL Plate 4	G9	10	0.481	0.0660	0.514	7.4	
Rxn #27	F7	FTL Plate 4	G9	5	0.493	0.0660	0.514	4.7	
Rxn #27	F8	FTL Plate 4	G9	1	0.495	0.0660	0.514	4.2	
Rxn #27	F9	FTL Plate 4	G9	0.5	0.510	0.0660	0.514	0.9	
Rxn #27	F10	FTL Plate 4	G9	0.1	0.507	0.0660	0.514	1.6	
Rxn #27	E1	FTL Plate 7	C7	200	0.204	0.0660	0.514	69.2	109.20
Rxn #27	E2	FTL Plate 7	C7	150	0.302	0.0660	0.514	47.3	
Rxn #27	E3	FTL Plate 7	C7	100	0.291	0.0660	0.514	49.8	
Rxn #27	E4	FTL Plate 7	C7	50	0.348	0.0660	0.514	37.1	
Rxn #27	E5	FTL Plate 7	C7	25	0.405	0.0660	0.514	24.3	
Rxn #27	E6	FTL Plate 7	C7	10	0.476	0.0660	0.514	8.5	

S2 Table. Data from Titrations of Candidate Botanical Extracts for Inhibition of KHKC Activity.

Rxn		Sample			Sample	Ave. No Fructose Controls	Ave. Fructose Only Controls	KHKC	*KHKC IC_{50}
ID	Well	Botanical Plate	Well	Conc (μg/mL)	(ΔOD_{340nm})	(ΔOD_{340nm})	(ΔOD_{340nm})	Inhibition (%)	(μg/mL)
Rxn #27	E7	FTL Plate 7	C7	5	0.481	0.0660	0.514	7.4	
Rxn #27	E8	FTL Plate 7	C7	1	0.501	0.0660	0.514	2.9	
Rxn #27	E9	FTL Plate 7	C7	0.5	0.510	0.0660	0.514	0.9	
Rxn #27	E10	FTL Plate 7	C7	0.1	0.507	0.0660	0.514	1.6	
Rxn #27	A12	No Fructose Control			0.075	0.0660	0.514	98.0	
Rxn #27	B12	No Fructose Control			0.065	0.0660	0.514	100.2	
Rxn #27	C12	No Fructose Control			0.066	0.0660	0.514	100.0	
Rxn #27	D12	No Fructose Control			0.068	0.0660	0.514	99.6	
Rxn #27	E12	No Fructose Control			0.064	0.0660	0.514	100.4	
Rxn #27	F12	No Fructose Control			0.059	0.0660	0.514	101.6	
Rxn #27	G12	No Fructose Control			0.063	0.0660	0.514	100.7	
Rxn #27	H12	No Fructose Control			0.068	0.0660	0.514	99.6	
Rxn #27	G1	Fructose Only Control			0.499	0.0660	0.514	3.3	
Rxn #27	H1	Fructose Only Control			0.513	0.0660	0.514	0.2	
Rxn #27	G2	Fructose Only Control			0.497	0.0660	0.514	3.8	
Rxn #27	H2	Fructose Only Control			0.514	0.0660	0.514	0.0	
Rxn #27	G3	Fructose Only Control			0.521	0.0660	0.514	-1.6	
Rxn #27	H3	Fructose Only Control			0.528	0.0660	0.514	-3.1	
Rxn #27	G4	Fructose Only Control			0.516	0.0660	0.514	-0.4	
Rxn #27	H4	Fructose Only Control			0.532	0.0660	0.514	-4.0	
Rxn #27	G5	Fructose Only Control			0.502	0.0660	0.514	2.7	
Rxn #27	H5	Fructose Only Control			0.513	0.0660	0.514	0.2	
Rxn #27	G6	Fructose Only Control			0.528	0.0660	0.514	-3.1	
Rxn #27	H6	Fructose Only Control			0.537	0.0660	0.514	-5.1	
Rxn #27	G7	Fructose Only Control			0.498	0.0660	0.514	3.6	
Rxn #27	H7	Fructose Only Control			0.512	0.0660	0.514	0.4	
Rxn #27	G8	Fructose Only Control			0.517	0.0660	0.514	-0.7	
Rxn #27	H8	Fructose Only Control			0.509	0.0660	0.514	1.1	
Rxn #27	G9	Fructose Only Control			0.509	0.0660	0.514	1.1	
Rxn #27	H9	Fructose Only Control			0.525	0.0660	0.514	-2.5	
Rxn #27	G10	Fructose Only Control			0.503	0.0660	0.514	2.5	
Rxn #27	H10	Fructose Only Control			0.516	0.0660	0.514	-0.4	
Rxn #27	A11	Fructose Only Control			0.509	0.0660	0.514	1.1	
Rxn #27	H11	Fructose Only Control			0.516	0.0660	0.514	-0.4	

FTL: functional target library; proprietary library of Amway Corp. IC_{50}: half maximal inhibitory concentration. OD: optical density.
*KHKC IC_{50}s were calculated using nonlinear regression (three parameters) in GraphPad Prism 5.03. To generate a best fit, an upper concentration (10,000 μg/mL at 100% inhibition) and a lower concentration (0.001 μg/mL at 0% inhibition) were added.
