# Supplementary material for: Bioactivity-Guided Identification of Botanical Inhibitors of Ketohexokinase
Source: PLoS One. 2016 Jun 20;11(6):e0157458. doi: 10.1371/journal.pone.0157458 (PMC4913896; doi:10.1371/journal.pone.0157458)
Supplement: S3 Table — AnalytiCon: Phytochemicals from MEGx library (AnalytiCon Discovery GmbH, Potsdam, Germany) were selected and purchased by Amway Corp. KHKC: ketohexokinase isoform C. FOC: fructose only control. NFC: no fructose control. OD: optical density. (PDF) [file pone.0157458.s003.pdf]

S3 Table. Data from Screening of Phytochemical Library for Inhibition of KHKC Activity.

Rxn ID	Sample			Sample	Ave. No Fructose Controls	Ave. Fructose Only Controls	KHKC
	Phytochemical Plate	Well	Conc (μM)	(ΔOD_{340nm})	(ΔOD_{340nm})	(ΔOD_{340nm})	Inhibition (%)
Rxn #32	AnalytiCon Plate #1	A1: NFC	-	0.069	0.0619	0.457	98.2
Rxn #32	AnalytiCon Plate #1	B1	50	0.360	0.0619	0.457	24.6
Rxn #32	AnalytiCon Plate #1	C1	50	0.409	0.0619	0.457	12.1
Rxn #32	AnalytiCon Plate #1	D1	50	0.387	0.0619	0.457	17.7
Rxn #32	AnalytiCon Plate #1	E1	50	0.404	0.0619	0.457	13.4
Rxn #32	AnalytiCon Plate #1	F1	50	0.359	0.0619	0.457	24.8
Rxn #32	AnalytiCon Plate #1	G1	50	0.409	0.0619	0.457	12.1
Rxn #32	AnalytiCon Plate #1	H1: FOC	-	0.438	0.0619	0.457	4.8
Rxn #32	AnalytiCon Plate #1	A2: NFC	-	0.057	0.0619	0.457	101.2
Rxn #32	AnalytiCon Plate #1	B2	50	0.397	0.0619	0.457	15.2
Rxn #32	AnalytiCon Plate #1	C2	50	0.471	0.0619	0.457	-3.5
Rxn #32	AnalytiCon Plate #1	D2	50	0.424	0.0619	0.457	8.4
Rxn #32	AnalytiCon Plate #1	E2	50	0.333	0.0619	0.457	31.4
Rxn #32	AnalytiCon Plate #1	F2	50	0.417	0.0619	0.457	10.1
Rxn #32	AnalytiCon Plate #1	G2	50	0.401	0.0619	0.457	14.2
Rxn #32	AnalytiCon Plate #1	H2: FOC	-	0.450	0.0619	0.457	1.8
Rxn #32	AnalytiCon Plate #1	A3: NFC	-	0.060	0.0619	0.457	100.5
Rxn #32	AnalytiCon Plate #1	B3	50	0.452	0.0619	0.457	1.3
Rxn #32	AnalytiCon Plate #1	C3	50	0.447	0.0619	0.457	2.5
Rxn #32	AnalytiCon Plate #1	D3	50	0.380	0.0619	0.457	19.5
Rxn #32	AnalytiCon Plate #1	E3	50	0.412	0.0619	0.457	11.4
Rxn #32	AnalytiCon Plate #1	F3	50	0.437	0.0619	0.457	5.1
Rxn #32	AnalytiCon Plate #1	G3	50	0.413	0.0619	0.457	11.1
Rxn #32	AnalytiCon Plate #1	H3: FOC	-	0.452	0.0619	0.457	1.3
Rxn #32	AnalytiCon Plate #1	A4: NFC	-	0.057	0.0619	0.457	101.2
Rxn #32	AnalytiCon Plate #1	B4	50	0.396	0.0619	0.457	15.4
Rxn #32	AnalytiCon Plate #1	C4	50	0.454	0.0619	0.457	0.8
Rxn #32	AnalytiCon Plate #1	D4	50	0.408	0.0619	0.457	12.4
Rxn #32	AnalytiCon Plate #1	E4	50	0.445	0.0619	0.457	3.0
Rxn #32	AnalytiCon Plate #1	F4	50	0.381	0.0619	0.457	19.2
Rxn #32	AnalytiCon Plate #1	G4	50	0.437	0.0619	0.457	5.1
Rxn #32	AnalytiCon Plate #1	H4: FOC	-	0.454	0.0619	0.457	0.8
Rxn #32	AnalytiCon Plate #1	A5: NFC	-	0.056	0.0619	0.457	101.5
Rxn #32	AnalytiCon Plate #1	B5: NFC	-	0.066	0.0619	0.457	99.0
Rxn #32	AnalytiCon Plate #1	C5: NFC	-	0.066	0.0619	0.457	99.0
Rxn #32	AnalytiCon Plate #1	D5: NFC	-	0.070	0.0619	0.457	97.9
Rxn #32	AnalytiCon Plate #1	E5: FOC	-	0.464	0.0619	0.457	-1.8
Rxn #32	AnalytiCon Plate #1	F5: FOC	-	0.467	0.0619	0.457	-2.5
Rxn #32	AnalytiCon Plate #1	G5: FOC	-	0.463	0.0619	0.457	-1.5
Rxn #32	AnalytiCon Plate #1	H5: FOC	-	0.466	0.0619	0.457	-2.3
Rxn #32	AnalytiCon Plate #1	A6: NFC	-	0.067	0.0619	0.457	98.7
Rxn #32	AnalytiCon Plate #1	B6	50	0.444	0.0619	0.457	3.3
Rxn #32	AnalytiCon Plate #1	C6	50	0.405	0.0619	0.457	13.2
Rxn #32	AnalytiCon Plate #1	D6	50	0.437	0.0619	0.457	5.1
Rxn #32	AnalytiCon Plate #1	E6	50	0.435	0.0619	0.457	5.6
Rxn #32	AnalytiCon Plate #1	F6	50	0.440	0.0619	0.457	4.3
Rxn #32	AnalytiCon Plate #1	G6	50	0.426	0.0619	0.457	7.8
Rxn #32	AnalytiCon Plate #1	H6: FOC	-	0.452	0.0619	0.457	1.3
Rxn #32	AnalytiCon Plate #1	A7: NFC	-	0.063	0.0619	0.457	99.7
Rxn #32	AnalytiCon Plate #1	B7	50	0.451	0.0619	0.457	1.5
Rxn #32	AnalytiCon Plate #1	C7	50	0.400	0.0619	0.457	14.4
Rxn #32	AnalytiCon Plate #1	D7	50	0.461	0.0619	0.457	-1.0
Rxn #32	AnalytiCon Plate #1	E7	50	0.461	0.0619	0.457	-1.0
Rxn #32	AnalytiCon Plate #1	F7	50	0.443	0.0619	0.457	3.5
Rxn #32	AnalytiCon Plate #1	G7	50	0.179	0.0619	0.457	70.4
Rxn #32	AnalytiCon Plate #1	H7: FOC	-	0.461	0.0619	0.457	-1.0
Rxn #32	AnalytiCon Plate #1	A8: NFC	-	0.057	0.0619	0.457	101.2
Rxn #32	AnalytiCon Plate #1	B8	50	0.414	0.0619	0.457	10.9
Rxn #32	AnalytiCon Plate #1	C8	50	0.445	0.0619	0.457	3.0
Rxn #32	AnalytiCon Plate #1	D8	50	0.439	0.0619	0.457	4.6
Rxn #32	AnalytiCon Plate #1	E8	50	0.454	0.0619	0.457	0.8
Rxn #32	AnalytiCon Plate #1	F8	50	0.442	0.0619	0.457	3.8
Rxn #32	AnalytiCon Plate #1	G8	50	0.439	0.0619	0.457	4.6
Rxn #32	AnalytiCon Plate #1	H8: FOC	-	0.440	0.0619	0.457	4.3
Rxn #32	AnalytiCon Plate #1	A9: NFC	-	0.061	0.0619	0.457	100.2
Rxn #32	AnalytiCon Plate #1	B9	50	0.452	0.0619	0.457	1.3
Rxn #32	AnalytiCon Plate #1	C9	50	0.461	0.0619	0.457	-1.0
Rxn #32	AnalytiCon Plate #1	D9	50	0.441	0.0619	0.457	4.0
Rxn #32	AnalytiCon Plate #1	E9	50	0.439	0.0619	0.457	4.6
Rxn #32	AnalytiCon Plate #1	F9	50	0.452	0.0619	0.457	1.3
Rxn #32	AnalytiCon Plate #1	G9	50	0.440	0.0619	0.457	4.3
Rxn #32	AnalytiCon Plate #1	H9: FOC	-	0.452	0.0619	0.457	1.3
Rxn #32	AnalytiCon Plate #1	A10: NFC	-	0.064	0.0619	0.457	99.5
Rxn #32	AnalytiCon Plate #1	B10	50	0.443	0.0619	0.457	3.5

S3 Table. Data from Screening of Phytochemical Library for Inhibition of KHKC Activity.

Rxn ID	Sample			Sample	Ave. No Fructose Controls	Ave. Fructose Only Controls	KHKC
	Phytochemical Plate	Well	Conc (μM)	(ΔOD_{340nm})	(ΔOD_{340nm})	(ΔOD_{340nm})	Inhibition (%)
Rxn #32	AnalytiCon Plate #1	C10	50	0.404	0.0619	0.457	13.4
Rxn #32	AnalytiCon Plate #1	D10	50	0.207	0.0619	0.457	63.3
Rxn #32	AnalytiCon Plate #1	E10	50	0.431	0.0619	0.457	6.6
Rxn #32	AnalytiCon Plate #1	F10	50	0.439	0.0619	0.457	4.6
Rxn #32	AnalytiCon Plate #1	G10	50	0.428	0.0619	0.457	7.3
Rxn #32	AnalytiCon Plate #1	H10: FOC	-	0.457	0.0619	0.457	0.0
Rxn #32	AnalytiCon Plate #1	A11: NFC	-	0.054	0.0619	0.457	102.0
Rxn #32	AnalytiCon Plate #1	B11	50	0.436	0.0619	0.457	5.3
Rxn #32	AnalytiCon Plate #1	C11	50	0.411	0.0619	0.457	11.6
Rxn #32	AnalytiCon Plate #1	D11	50	0.471	0.0619	0.457	-3.5
Rxn #32	AnalytiCon Plate #1	E11	50	0.425	0.0619	0.457	8.1
Rxn #32	AnalytiCon Plate #1	F11	50	0.433	0.0619	0.457	6.1
Rxn #32	AnalytiCon Plate #1	G11	50	0.445	0.0619	0.457	3.0
Rxn #32	AnalytiCon Plate #1	H11: FOC	-	0.437	0.0619	0.457	5.1
Rxn #32	AnalytiCon Plate #1	A12: NFC	-	0.062	0.0619	0.457	100.0
Rxn #32	AnalytiCon Plate #1	H12: FOC	-	0.441	0.0619	0.457	4.0
Rxn #33	AnalytiCon Plate #2	A1: NFC	-	0.045	0.0516	0.499	101.5
Rxn #33	AnalytiCon Plate #2	B1	50	0.423	0.0516	0.499	17.0
Rxn #33	AnalytiCon Plate #2	C1	50	0.446	0.0516	0.499	11.8
Rxn #33	AnalytiCon Plate #2	D1	50	0.424	0.0516	0.499	16.8
Rxn #33	AnalytiCon Plate #2	E1	50	0.438	0.0516	0.499	13.6
Rxn #33	AnalytiCon Plate #2	F1	50	0.379	0.0516	0.499	26.8
Rxn #33	AnalytiCon Plate #2	G1	50	0.451	0.0516	0.499	10.7
Rxn #33	AnalytiCon Plate #2	H1: FOC	-	0.483	0.0516	0.499	3.6
Rxn #33	AnalytiCon Plate #2	A2: NFC	-	0.051	0.0516	0.499	100.1
Rxn #33	AnalytiCon Plate #2	B2	50	0.482	0.0516	0.499	3.8
Rxn #33	AnalytiCon Plate #2	C2	50	0.399	0.0516	0.499	22.4
Rxn #33	AnalytiCon Plate #2	D2	50	0.476	0.0516	0.499	5.1
Rxn #33	AnalytiCon Plate #2	E2	50	0.500	0.0516	0.499	-0.2
Rxn #33	AnalytiCon Plate #2	F2	50	0.488	0.0516	0.499	2.5
Rxn #33	AnalytiCon Plate #2	G2	50	0.490	0.0516	0.499	2.0
Rxn #33	AnalytiCon Plate #2	H2: FOC	-	0.506	0.0516	0.499	-1.6
Rxn #33	AnalytiCon Plate #2	A3: NFC	-	0.050	0.0516	0.499	100.4
Rxn #33	AnalytiCon Plate #2	B3	50	0.449	0.0516	0.499	11.2
Rxn #33	AnalytiCon Plate #2	C3	50	0.481	0.0516	0.499	4.0
Rxn #33	AnalytiCon Plate #2	D3	50	0.367	0.0516	0.499	29.5
Rxn #33	AnalytiCon Plate #2	E3	50	0.397	0.0516	0.499	22.8
Rxn #33	AnalytiCon Plate #2	F3	50	0.484	0.0516	0.499	3.4
Rxn #33	AnalytiCon Plate #2	G3	50	0.488	0.0516	0.499	2.5
Rxn #33	AnalytiCon Plate #2	H3: FOC	-	0.514	0.0516	0.499	-3.4
Rxn #33	AnalytiCon Plate #2	A4: NFC	-	0.047	0.0516	0.499	101.0
Rxn #33	AnalytiCon Plate #2	B4	50	0.474	0.0516	0.499	5.6
Rxn #33	AnalytiCon Plate #2	C4	50	0.453	0.0516	0.499	10.3
Rxn #33	AnalytiCon Plate #2	D4	50	0.508	0.0516	0.499	-2.0
Rxn #33	AnalytiCon Plate #2	E4	50	0.412	0.0516	0.499	19.4
Rxn #33	AnalytiCon Plate #2	F4	50	0.493	0.0516	0.499	1.3
Rxn #33	AnalytiCon Plate #2	G4	50	0.485	0.0516	0.499	3.1
Rxn #33	AnalytiCon Plate #2	H4: FOC	-	0.501	0.0516	0.499	-0.4
Rxn #33	AnalytiCon Plate #2	A5: NFC	-	0.053	0.0516	0.499	99.7
Rxn #33	AnalytiCon Plate #2	B5: NFC	-	0.051	0.0516	0.499	100.1
Rxn #33	AnalytiCon Plate #2	C5: NFC	-	0.058	0.0516	0.499	98.6
Rxn #33	AnalytiCon Plate #2	D5: NFC	-	0.047	0.0516	0.499	101.0
Rxn #33	AnalytiCon Plate #2	E5: FOC	-	0.482	0.0516	0.499	3.8
Rxn #33	AnalytiCon Plate #2	F5: FOC	-	0.493	0.0516	0.499	1.3
Rxn #33	AnalytiCon Plate #2	G5: FOC	-	0.496	0.0516	0.499	0.7
Rxn #33	AnalytiCon Plate #2	H5: FOC	-	0.501	0.0516	0.499	-0.4
Rxn #33	AnalytiCon Plate #2	A6: NFC	-	0.054	0.0516	0.499	99.5
Rxn #33	AnalytiCon Plate #2	B6	50	0.477	0.0516	0.499	4.9
Rxn #33	AnalytiCon Plate #2	C6	50	0.482	0.0516	0.499	3.8
Rxn #33	AnalytiCon Plate #2	D6	50	0.480	0.0516	0.499	4.2
Rxn #33	AnalytiCon Plate #2	E6	50	0.482	0.0516	0.499	3.8
Rxn #33	AnalytiCon Plate #2	F6	50	0.484	0.0516	0.499	3.4
Rxn #33	AnalytiCon Plate #2	G6	50	0.477	0.0516	0.499	4.9
Rxn #33	AnalytiCon Plate #2	H6: FOC	-	0.506	0.0516	0.499	-1.6
Rxn #33	AnalytiCon Plate #2	A7: NFC	-	0.044	0.0516	0.499	101.7
Rxn #33	AnalytiCon Plate #2	B7	50	0.451	0.0516	0.499	10.7
Rxn #33	AnalytiCon Plate #2	C7	50	0.458	0.0516	0.499	9.2
Rxn #33	AnalytiCon Plate #2	D7	50	0.482	0.0516	0.499	3.8
Rxn #33	AnalytiCon Plate #2	E7	50	0.461	0.0516	0.499	8.5
Rxn #33	AnalytiCon Plate #2	F7	50	0.489	0.0516	0.499	2.2
Rxn #33	AnalytiCon Plate #2	G7	50	0.483	0.0516	0.499	3.6
Rxn #33	AnalytiCon Plate #2	H7: FOC	-	0.505	0.0516	0.499	-1.3
Rxn #33	AnalytiCon Plate #2	A8: NFC	-	0.058	0.0516	0.499	98.6
Rxn #33	AnalytiCon Plate #2	B8	50	0.225	0.0516	0.499	61.2

S3 Table. Data from Screening of Phytochemical Library for Inhibition of KHKC Activity.

Rxn ID	Sample			Sample	Ave. No Fructose Controls	Ave. Fructose Only Controls	KHKC
	Phytochemical Plate	Well	Conc (μM)	(ΔOD_{340nm})	(ΔOD_{340nm})	(ΔOD_{340nm})	Inhibition (%)
Rxn #33	AnalytiCon Plate #2	C8	50	0.223	0.0516	0.499	61.7
Rxn #33	AnalytiCon Plate #2	D8	50	0.432	0.0516	0.499	15.0
Rxn #33	AnalytiCon Plate #2	E8	50	0.332	0.0516	0.499	37.3
Rxn #33	AnalytiCon Plate #2	F8	50	0.281	0.0516	0.499	48.7
Rxn #33	AnalytiCon Plate #2	G8	50	0.445	0.0516	0.499	12.1
Rxn #33	AnalytiCon Plate #2	H8: FOC	-	0.502	0.0516	0.499	-0.7
Rxn #33	AnalytiCon Plate #2	A9: NFC	-	0.054	0.0516	0.499	99.5
Rxn #33	AnalytiCon Plate #2	B9	50	0.450	0.0516	0.499	11.0
Rxn #33	AnalytiCon Plate #2	C9	50	0.453	0.0516	0.499	10.3
Rxn #33	AnalytiCon Plate #2	D9	50	0.426	0.0516	0.499	16.3
Rxn #33	AnalytiCon Plate #2	E9	50	0.466	0.0516	0.499	7.4
Rxn #33	AnalytiCon Plate #2	F9	50	0.366	0.0516	0.499	29.7
Rxn #33	AnalytiCon Plate #2	G9	50	0.471	0.0516	0.499	6.3
Rxn #33	AnalytiCon Plate #2	H9: FOC	-	0.488	0.0516	0.499	2.5
Rxn #33	AnalytiCon Plate #2	A10: NFC	-	0.050	0.0516	0.499	100.4
Rxn #33	AnalytiCon Plate #2	B10	50	0.361	0.0516	0.499	30.8
Rxn #33	AnalytiCon Plate #2	C10	50	0.439	0.0516	0.499	13.4
Rxn #33	AnalytiCon Plate #2	D10	50	0.408	0.0516	0.499	20.3
Rxn #33	AnalytiCon Plate #2	E10	50	0.353	0.0516	0.499	32.6
Rxn #33	AnalytiCon Plate #2	F10	50	0.505	0.0516	0.499	-1.3
Rxn #33	AnalytiCon Plate #2	G10	50	0.081	0.0516	0.499	93.4
Rxn #33	AnalytiCon Plate #2	H10: FOC	-	0.487	0.0516	0.499	2.7
Rxn #33	AnalytiCon Plate #2	A11: NFC	-	0.055	0.0516	0.499	99.2
Rxn #33	AnalytiCon Plate #2	B11	50	0.479	0.0516	0.499	4.5
Rxn #33	AnalytiCon Plate #2	C11	50	0.466	0.0516	0.499	7.4
Rxn #33	AnalytiCon Plate #2	D11	50	0.445	0.0516	0.499	12.1
Rxn #33	AnalytiCon Plate #2	E11	50	0.496	0.0516	0.499	0.7
Rxn #33	AnalytiCon Plate #2	F11	50	0.473	0.0516	0.499	5.8
Rxn #33	AnalytiCon Plate #2	G11	50	0.493	0.0516	0.499	1.3
Rxn #33	AnalytiCon Plate #2	H11: FOC	-	0.470	0.0516	0.499	6.5
Rxn #33	AnalytiCon Plate #2	A12: NFC	-	0.057	0.0516	0.499	98.8
Rxn #33	AnalytiCon Plate #2	H12: FOC	-	0.475	0.0516	0.499	5.4
Rxn #34	AnalytiCon Plate #3	A1: NFC	-	0.051	0.0543	0.551	100.7
Rxn #34	AnalytiCon Plate #3	B1	50	0.525	0.0543	0.551	5.2
Rxn #34	AnalytiCon Plate #3	C1	50	0.524	0.0543	0.551	5.4
Rxn #34	AnalytiCon Plate #3	D1	50	0.466	0.0543	0.551	17.1
Rxn #34	AnalytiCon Plate #3	E1	50	0.342	0.0543	0.551	42.1
Rxn #34	AnalytiCon Plate #3	F1	50	0.453	0.0543	0.551	19.7
Rxn #34	AnalytiCon Plate #3	G1	50	0.516	0.0543	0.551	7.0
Rxn #34	AnalytiCon Plate #3	H1: FOC	-	0.520	0.0543	0.551	6.2
Rxn #34	AnalytiCon Plate #3	A2: NFC	-	0.048	0.0543	0.551	101.3
Rxn #34	AnalytiCon Plate #3	B2	50	0.541	0.0543	0.551	2.0
Rxn #34	AnalytiCon Plate #3	C2	50	0.537	0.0543	0.551	2.8
Rxn #34	AnalytiCon Plate #3	D2	50	0.430	0.0543	0.551	24.4
Rxn #34	AnalytiCon Plate #3	E2	50	0.517	0.0543	0.551	6.8
Rxn #34	AnalytiCon Plate #3	F2	50	0.521	0.0543	0.551	6.0
Rxn #34	AnalytiCon Plate #3	G2	50	0.529	0.0543	0.551	4.4
Rxn #34	AnalytiCon Plate #3	H2: FOC	-	0.552	0.0543	0.551	-0.2
Rxn #34	AnalytiCon Plate #3	A3: NFC	-	0.054	0.0543	0.551	100.1
Rxn #34	AnalytiCon Plate #3	B3	50	0.536	0.0543	0.551	3.0
Rxn #34	AnalytiCon Plate #3	C3	50	0.541	0.0543	0.551	2.0
Rxn #34	AnalytiCon Plate #3	D3	50	0.513	0.0543	0.551	7.7
Rxn #34	AnalytiCon Plate #3	E3	50	0.430	0.0543	0.551	24.4
Rxn #34	AnalytiCon Plate #3	F3	50	0.469	0.0543	0.551	16.5
Rxn #34	AnalytiCon Plate #3	G3	50	0.431	0.0543	0.551	24.2
Rxn #34	AnalytiCon Plate #3	H3: FOC	-	0.552	0.0543	0.551	-0.2
Rxn #34	AnalytiCon Plate #3	A4: NFC	-	0.058	0.0543	0.551	99.3
Rxn #34	AnalytiCon Plate #3	B4	50	0.524	0.0543	0.551	5.4
Rxn #34	AnalytiCon Plate #3	C4	50	0.524	0.0543	0.551	5.4
Rxn #34	AnalytiCon Plate #3	D4	50	0.496	0.0543	0.551	11.1
Rxn #34	AnalytiCon Plate #3	E4	50	0.504	0.0543	0.551	9.5
Rxn #34	AnalytiCon Plate #3	F4	50	0.529	0.0543	0.551	4.4
Rxn #34	AnalytiCon Plate #3	G4	50	0.472	0.0543	0.551	15.9
Rxn #34	AnalytiCon Plate #3	H4: FOC	-	0.557	0.0543	0.551	-1.2
Rxn #34	AnalytiCon Plate #3	A5: NFC	-	0.052	0.0543	0.551	100.5
Rxn #34	AnalytiCon Plate #3	B5: NFC	-	0.047	0.0543	0.551	101.5
Rxn #34	AnalytiCon Plate #3	C5: NFC	-	0.049	0.0543	0.551	101.1
Rxn #34	AnalytiCon Plate #3	D5: NFC	-	0.052	0.0543	0.551	100.5
Rxn #34	AnalytiCon Plate #3	E5: FOC	-	0.553	0.0543	0.551	-0.4
Rxn #34	AnalytiCon Plate #3	F5: FOC	-	0.536	0.0543	0.551	3.0
Rxn #34	AnalytiCon Plate #3	G5: FOC	-	0.555	0.0543	0.551	-0.8
Rxn #34	AnalytiCon Plate #3	H5: FOC	-	0.559	0.0543	0.551	-1.6
Rxn #34	AnalytiCon Plate #3	A6: NFC	-	0.058	0.0543	0.551	99.3
Rxn #34	AnalytiCon Plate #3	B6	50	0.523	0.0543	0.551	5.6

S3 Table. Data from Screening of Phytochemical Library for Inhibition of KHKC Activity.

Rxn ID	Sample			Sample	Ave. No Fructose Controls	Ave. Fructose Only Controls	KHKC
	Phytochemical Plate	Well	Conc (μM)	(ΔOD_{340nm})	(ΔOD_{340nm})	(ΔOD_{340nm})	Inhibition (%)
Rxn #34	AnalytiCon Plate #3	C6	50	0.472	0.0543	0.551	15.9
Rxn #34	AnalytiCon Plate #3	D6	50	0.381	0.0543	0.551	34.2
Rxn #34	AnalytiCon Plate #3	E6	50	0.509	0.0543	0.551	8.5
Rxn #34	AnalytiCon Plate #3	F6	50	0.497	0.0543	0.551	10.9
Rxn #34	AnalytiCon Plate #3	G6	50	0.531	0.0543	0.551	4.0
Rxn #34	AnalytiCon Plate #3	H6: FOC	-	0.559	0.0543	0.551	-1.6
Rxn #34	AnalytiCon Plate #3	A7: NFC	-	0.056	0.0543	0.551	99.7
Rxn #34	AnalytiCon Plate #3	B7	50	0.485	0.0543	0.551	13.3
Rxn #34	AnalytiCon Plate #3	C7	50	0.521	0.0543	0.551	6.0
Rxn #34	AnalytiCon Plate #3	D7	50	0.247	0.0543	0.551	61.2
Rxn #34	AnalytiCon Plate #3	E7	50	0.438	0.0543	0.551	22.8
Rxn #34	AnalytiCon Plate #3	F7	50	0.466	0.0543	0.551	17.1
Rxn #34	AnalytiCon Plate #3	G7	50	0.527	0.0543	0.551	4.8
Rxn #34	AnalytiCon Plate #3	H7: FOC	-	0.563	0.0543	0.551	-2.4
Rxn #34	AnalytiCon Plate #3	A8: NFC	-	0.062	0.0543	0.551	98.4
Rxn #34	AnalytiCon Plate #3	B8	50	0.530	0.0543	0.551	4.2
Rxn #34	AnalytiCon Plate #3	C8	50	0.515	0.0543	0.551	7.2
Rxn #34	AnalytiCon Plate #3	D8	50	0.529	0.0543	0.551	4.4
Rxn #34	AnalytiCon Plate #3	E8	50	0.478	0.0543	0.551	14.7
Rxn #34	AnalytiCon Plate #3	F8	50	0.547	0.0543	0.551	0.8
Rxn #34	AnalytiCon Plate #3	G8	50	0.574	0.0543	0.551	-4.6
Rxn #34	AnalytiCon Plate #3	H8: FOC	-	0.563	0.0543	0.551	-2.4
Rxn #34	AnalytiCon Plate #3	A9: NFC	-	0.061	0.0543	0.551	98.7
Rxn #34	AnalytiCon Plate #3	B9	50	0.497	0.0543	0.551	10.9
Rxn #34	AnalytiCon Plate #3	C9	50	0.440	0.0543	0.551	22.3
Rxn #34	AnalytiCon Plate #3	D9	50	0.513	0.0543	0.551	7.7
Rxn #34	AnalytiCon Plate #3	E9	50	0.476	0.0543	0.551	15.1
Rxn #34	AnalytiCon Plate #3	F9	50	0.527	0.0543	0.551	4.8
Rxn #34	AnalytiCon Plate #3	G9	50	0.542	0.0543	0.551	1.8
Rxn #34	AnalytiCon Plate #3	H9: FOC	-	0.573	0.0543	0.551	-4.4
Rxn #34	AnalytiCon Plate #3	A10: NFC	-	0.054	0.0543	0.551	100.1
Rxn #34	AnalytiCon Plate #3	B10	50	0.504	0.0543	0.551	9.5
Rxn #34	AnalytiCon Plate #3	C10	50	0.573	0.0543	0.551	-4.4
Rxn #34	AnalytiCon Plate #3	D10	50	0.577	0.0543	0.551	-5.2
Rxn #34	AnalytiCon Plate #3	E10	50	0.483	0.0543	0.551	13.7
Rxn #34	AnalytiCon Plate #3	F10	50	0.531	0.0543	0.551	4.0
Rxn #34	AnalytiCon Plate #3	G10	50	0.542	0.0543	0.551	1.8
Rxn #34	AnalytiCon Plate #3	H10: FOC	-	0.568	0.0543	0.551	-3.4
Rxn #34	AnalytiCon Plate #3	A11: NFC	-	0.055	0.0543	0.551	99.9
Rxn #34	AnalytiCon Plate #3	B11	50	0.460	0.0543	0.551	18.3
Rxn #34	AnalytiCon Plate #3	C11	50	0.538	0.0543	0.551	2.6
Rxn #34	AnalytiCon Plate #3	D11	50	0.551	0.0543	0.551	0.0
Rxn #34	AnalytiCon Plate #3	E11	50	0.551	0.0543	0.551	0.0
Rxn #34	AnalytiCon Plate #3	F11	50	0.487	0.0543	0.551	12.9
Rxn #34	AnalytiCon Plate #3	G11	50	0.548	0.0543	0.551	0.6
Rxn #34	AnalytiCon Plate #3	H11: FOC	-	0.580	0.0543	0.551	-5.8
Rxn #34	AnalytiCon Plate #3	A12: NFC	-	0.057	0.0543	0.551	99.5
Rxn #34	AnalytiCon Plate #3	H12: FOC	-	0.591	0.0543	0.551	-8.1
Rxn #35	AnalytiCon Plate #4	A1: NFC	-	0.042	0.0465	0.470	101.1
Rxn #35	AnalytiCon Plate #4	B1	50	0.361	0.0465	0.470	25.7
Rxn #35	AnalytiCon Plate #4	C1	50	0.393	0.0465	0.470	18.2
Rxn #35	AnalytiCon Plate #4	D1	50	0.388	0.0465	0.470	19.4
Rxn #35	AnalytiCon Plate #4	E1	50	0.396	0.0465	0.470	17.5
Rxn #35	AnalytiCon Plate #4	F1	50	0.457	0.0465	0.470	3.1
Rxn #35	AnalytiCon Plate #4	G1	50	0.439	0.0465	0.470	7.3
Rxn #35	AnalytiCon Plate #4	H1: FOC	-	0.454	0.0465	0.470	3.8
Rxn #35	AnalytiCon Plate #4	A2: NFC	-	0.045	0.0465	0.470	100.4
Rxn #35	AnalytiCon Plate #4	B2	50	0.446	0.0465	0.470	5.7
Rxn #35	AnalytiCon Plate #4	C2	50	0.431	0.0465	0.470	9.2
Rxn #35	AnalytiCon Plate #4	D2	50	0.452	0.0465	0.470	4.3
Rxn #35	AnalytiCon Plate #4	E2	50	0.395	0.0465	0.470	17.7
Rxn #35	AnalytiCon Plate #4	F2	50	0.087	0.0465	0.470	90.4
Rxn #35	AnalytiCon Plate #4	G2	50	0.461	0.0465	0.470	2.1
Rxn #35	AnalytiCon Plate #4	H2: FOC	-	0.471	0.0465	0.470	-0.2
Rxn #35	AnalytiCon Plate #4	A3: NFC	-	0.043	0.0465	0.470	100.8
Rxn #35	AnalytiCon Plate #4	B3	50	0.429	0.0465	0.470	9.7
Rxn #35	AnalytiCon Plate #4	C3	50	0.439	0.0465	0.470	7.3
Rxn #35	AnalytiCon Plate #4	D3	50	0.458	0.0465	0.470	2.8
Rxn #35	AnalytiCon Plate #4	E3	50	0.443	0.0465	0.470	6.4
Rxn #35	AnalytiCon Plate #4	F3	50	0.245	0.0465	0.470	53.1
Rxn #35	AnalytiCon Plate #4	G3	50	0.430	0.0465	0.470	9.4
Rxn #35	AnalytiCon Plate #4	H3: FOC	-	0.470	0.0465	0.470	0.0
Rxn #35	AnalytiCon Plate #4	A4: NFC	-	0.033	0.0465	0.470	103.2
Rxn #35	AnalytiCon Plate #4	B4	50	0.439	0.0465	0.470	7.3

S3 Table. Data from Screening of Phytochemical Library for Inhibition of KHKC Activity.

Rxn ID	Sample			Sample	Ave. No Fructose Controls	Ave. Fructose Only Controls	KHKC
	Phytochemical Plate	Well	Conc (μM)	(ΔOD_{340nm})	(ΔOD_{340nm})	(ΔOD_{340nm})	Inhibition (%)
Rxn #35	AnalytiCon Plate #4	C4	50	0.318	0.0465	0.470	35.9
Rxn #35	AnalytiCon Plate #4	D4	50	0.427	0.0465	0.470	10.2
Rxn #35	AnalytiCon Plate #4	E4	50	0.395	0.0465	0.470	17.7
Rxn #35	AnalytiCon Plate #4	F4	50	0.446	0.0465	0.470	5.7
Rxn #35	AnalytiCon Plate #4	G4	50	0.448	0.0465	0.470	5.2
Rxn #35	AnalytiCon Plate #4	H4: FOC	-	0.475	0.0465	0.470	-1.2
Rxn #35	AnalytiCon Plate #4	A5: NFC	-	0.040	0.0465	0.470	101.5
Rxn #35	AnalytiCon Plate #4	B5: NFC	-	0.053	0.0465	0.470	98.5
Rxn #35	AnalytiCon Plate #4	C5: NFC	-	0.048	0.0465	0.470	99.6
Rxn #35	AnalytiCon Plate #4	D5: NFC	-	0.053	0.0465	0.470	98.5
Rxn #35	AnalytiCon Plate #4	E5: FOC	-	0.464	0.0465	0.470	1.4
Rxn #35	AnalytiCon Plate #4	F5: FOC	-	0.470	0.0465	0.470	0.0
Rxn #35	AnalytiCon Plate #4	G5: FOC	-	0.473	0.0465	0.470	-0.7
Rxn #35	AnalytiCon Plate #4	H5: FOC	-	0.479	0.0465	0.470	-2.1
Rxn #35	AnalytiCon Plate #4	A6: NFC	-	0.053	0.0465	0.470	98.5
Rxn #35	AnalytiCon Plate #4	B6	50	0.428	0.0465	0.470	9.9
Rxn #35	AnalytiCon Plate #4	C6	50	0.439	0.0465	0.470	7.3
Rxn #35	AnalytiCon Plate #4	D6	50	0.436	0.0465	0.470	8.0
Rxn #35	AnalytiCon Plate #4	E6	50	0.436	0.0465	0.470	8.0
Rxn #35	AnalytiCon Plate #4	F6	50	0.457	0.0465	0.470	3.1
Rxn #35	AnalytiCon Plate #4	G6	50	0.429	0.0465	0.470	9.7
Rxn #35	AnalytiCon Plate #4	H6: FOC	-	0.478	0.0465	0.470	-1.9
Rxn #35	AnalytiCon Plate #4	A7: NFC	-	0.049	0.0465	0.470	99.4
Rxn #35	AnalytiCon Plate #4	B7	50	0.425	0.0465	0.470	10.6
Rxn #35	AnalytiCon Plate #4	C7	50	0.431	0.0465	0.470	9.2
Rxn #35	AnalytiCon Plate #4	D7	50	0.440	0.0465	0.470	7.1
Rxn #35	AnalytiCon Plate #4	E7	50	0.442	0.0465	0.470	6.6
Rxn #35	AnalytiCon Plate #4	F7	50	0.460	0.0465	0.470	2.4
Rxn #35	AnalytiCon Plate #4	G7	50	0.517	0.0465	0.470	-11.1
Rxn #35	AnalytiCon Plate #4	H7: FOC	-	0.467	0.0465	0.470	0.7
Rxn #35	AnalytiCon Plate #4	A8: NFC	-	0.051	0.0465	0.470	98.9
Rxn #35	AnalytiCon Plate #4	B8	50	0.416	0.0465	0.470	12.8
Rxn #35	AnalytiCon Plate #4	C8	50	0.431	0.0465	0.470	9.2
Rxn #35	AnalytiCon Plate #4	D8	50	0.466	0.0465	0.470	0.9
Rxn #35	AnalytiCon Plate #4	E8	50	0.472	0.0465	0.470	-0.5
Rxn #35	AnalytiCon Plate #4	F8	50	0.462	0.0465	0.470	1.9
Rxn #35	AnalytiCon Plate #4	G8	50	0.453	0.0465	0.470	4.0
Rxn #35	AnalytiCon Plate #4	H8: FOC	-	0.485	0.0465	0.470	-3.5
Rxn #35	AnalytiCon Plate #4	A9: NFC	-	0.046	0.0465	0.470	100.1
Rxn #35	AnalytiCon Plate #4	B9	50	0.502	0.0465	0.470	-7.6
Rxn #35	AnalytiCon Plate #4	C9	50	0.170	0.0465	0.470	70.8
Rxn #35	AnalytiCon Plate #4	D9	50	0.429	0.0465	0.470	9.7
Rxn #35	AnalytiCon Plate #4	E9	50	0.453	0.0465	0.470	4.0
Rxn #35	AnalytiCon Plate #4	F9	50	0.461	0.0465	0.470	2.1
Rxn #35	AnalytiCon Plate #4	G9	50	0.412	0.0465	0.470	13.7
Rxn #35	AnalytiCon Plate #4	H9: FOC	-	0.481	0.0465	0.470	-2.6
Rxn #35	AnalytiCon Plate #4	A10: NFC	-	0.046	0.0465	0.470	100.1
Rxn #35	AnalytiCon Plate #4	B10	50	0.441	0.0465	0.470	6.8
Rxn #35	AnalytiCon Plate #4	C10	50	0.466	0.0465	0.470	0.9
Rxn #35	AnalytiCon Plate #4	D10	50	0.465	0.0465	0.470	1.2
Rxn #35	AnalytiCon Plate #4	E10	50	0.313	0.0465	0.470	37.1
Rxn #35	AnalytiCon Plate #4	F10	50	0.463	0.0465	0.470	1.7
Rxn #35	AnalytiCon Plate #4	G10	50	0.469	0.0465	0.470	0.2
Rxn #35	AnalytiCon Plate #4	H10: FOC	-	0.476	0.0465	0.470	-1.4
Rxn #35	AnalytiCon Plate #4	A11: NFC	-	0.042	0.0465	0.470	101.1
Rxn #35	AnalytiCon Plate #4	B11	50	0.439	0.0465	0.470	7.3
Rxn #35	AnalytiCon Plate #4	C11	50	0.453	0.0465	0.470	4.0
Rxn #35	AnalytiCon Plate #4	D11	50	0.463	0.0465	0.470	1.7
Rxn #35	AnalytiCon Plate #4	E11	50	0.470	0.0465	0.470	0.0
Rxn #35	AnalytiCon Plate #4	F11	50	0.466	0.0465	0.470	0.9
Rxn #35	AnalytiCon Plate #4	G11	50	0.427	0.0465	0.470	10.2
Rxn #35	AnalytiCon Plate #4	H11: FOC	-	0.471	0.0465	0.470	-0.2
Rxn #35	AnalytiCon Plate #4	A12: NFC	-	0.054	0.0465	0.470	98.2
Rxn #35	AnalytiCon Plate #4	H12: FOC	-	0.454	0.0465	0.470	3.8
Rxn #36	AnalytiCon Plate #5	A1: NFC	-	0.090	0.0941	0.611	100.8
Rxn #36	AnalytiCon Plate #5	B1	50	0.518	0.0941	0.611	18.0
Rxn #36	AnalytiCon Plate #5	C1	50	0.518	0.0941	0.611	18.0
Rxn #36	AnalytiCon Plate #5	D1	50	0.585	0.0941	0.611	5.0
Rxn #36	AnalytiCon Plate #5	E1	50	0.517	0.0941	0.611	18.2
Rxn #36	AnalytiCon Plate #5	F1	50	0.583	0.0941	0.611	5.4
Rxn #36	AnalytiCon Plate #5	G1	50	0.565	0.0941	0.611	8.9
Rxn #36	AnalytiCon Plate #5	H1: FOC	-	0.583	0.0941	0.611	5.4
Rxn #36	AnalytiCon Plate #5	A2: NFC	-	0.094	0.0941	0.611	100.0
Rxn #36	AnalytiCon Plate #5	B2	50	0.592	0.0941	0.611	3.7

S3 Table. Data from Screening of Phytochemical Library for Inhibition of KHKC Activity.

Rxn ID	Sample			Sample	Ave. No Fructose Controls	Ave. Fructose Only Controls	KHKC
	Phytochemical Plate	Well	Conc (μM)	(ΔOD_{340nm})	(ΔOD_{340nm})	(ΔOD_{340nm})	Inhibition (%)
Rxn #36	AnalytiCon Plate #5	C2	50	0.415	0.0941	0.611	37.9
Rxn #36	AnalytiCon Plate #5	D2	50	0.577	0.0941	0.611	6.6
Rxn #36	AnalytiCon Plate #5	E2	50	0.594	0.0941	0.611	3.3
Rxn #36	AnalytiCon Plate #5	F2	50	0.588	0.0941	0.611	4.4
Rxn #36	AnalytiCon Plate #5	G2	50	0.604	0.0941	0.611	1.4
Rxn #36	AnalytiCon Plate #5	H2: FOC	-	0.615	0.0941	0.611	-0.8
Rxn #36	AnalytiCon Plate #5	A3: NFC	-	0.092	0.0941	0.611	100.4
Rxn #36	AnalytiCon Plate #5	B3	50	0.613	0.0941	0.611	-0.4
Rxn #36	AnalytiCon Plate #5	C3	50	0.617	0.0941	0.611	-1.2
Rxn #36	AnalytiCon Plate #5	D3	50	0.598	0.0941	0.611	2.5
Rxn #36	AnalytiCon Plate #5	E3	50	0.626	0.0941	0.611	-2.9
Rxn #36	AnalytiCon Plate #5	F3	50	0.602	0.0941	0.611	1.7
Rxn #36	AnalytiCon Plate #5	G3	50	0.627	0.0941	0.611	-3.1
Rxn #36	AnalytiCon Plate #5	H3: FOC	-	0.614	0.0941	0.611	-0.6
Rxn #36	AnalytiCon Plate #5	A4: NFC	-	0.084	0.0941	0.611	102.0
Rxn #36	AnalytiCon Plate #5	B4	50	0.624	0.0941	0.611	-2.5
Rxn #36	AnalytiCon Plate #5	C4	50	0.613	0.0941	0.611	-0.4
Rxn #36	AnalytiCon Plate #5	D4	50	0.630	0.0941	0.611	-3.7
Rxn #36	AnalytiCon Plate #5	E4	50	0.613	0.0941	0.611	-0.4
Rxn #36	AnalytiCon Plate #5	F4	50	0.609	0.0941	0.611	0.4
Rxn #36	AnalytiCon Plate #5	G4	50	0.579	0.0941	0.611	6.2
Rxn #36	AnalytiCon Plate #5	H4: FOC	-	0.616	0.0941	0.611	-1.0
Rxn #36	AnalytiCon Plate #5	A5: NFC	-	0.101	0.0941	0.611	98.7
Rxn #36	AnalytiCon Plate #5	B5: NFC	-	0.082	0.0941	0.611	102.3
Rxn #36	AnalytiCon Plate #5	C5: NFC	-	0.079	0.0941	0.611	102.9
Rxn #36	AnalytiCon Plate #5	D5: NFC	-	0.081	0.0941	0.611	102.5
Rxn #36	AnalytiCon Plate #5	E5: FOC	-	0.624	0.0941	0.611	-2.5
Rxn #36	AnalytiCon Plate #5	F5: FOC	-	0.613	0.0941	0.611	-0.4
Rxn #36	AnalytiCon Plate #5	G5: FOC	-	0.604	0.0941	0.611	1.4
Rxn #36	AnalytiCon Plate #5	H5: FOC	-	0.617	0.0941	0.611	-1.2
Rxn #36	AnalytiCon Plate #5	A6: NFC	-	0.093	0.0941	0.611	100.2
Rxn #36	AnalytiCon Plate #5	B6	50	0.602	0.0941	0.611	1.7
Rxn #36	AnalytiCon Plate #5	C6	50	0.545	0.0941	0.611	12.8
Rxn #36	AnalytiCon Plate #5	D6	50	0.605	0.0941	0.611	1.2
Rxn #36	AnalytiCon Plate #5	E6	50	0.619	0.0941	0.611	-1.5
Rxn #36	AnalytiCon Plate #5	F6	50	0.611	0.0941	0.611	0.0
Rxn #36	AnalytiCon Plate #5	G6	50	0.588	0.0941	0.611	4.4
Rxn #36	AnalytiCon Plate #5	H6: FOC	-	0.613	0.0941	0.611	-0.4
Rxn #36	AnalytiCon Plate #5	A7: NFC	-	0.102	0.0941	0.611	98.5
Rxn #36	AnalytiCon Plate #5	B7	50	0.609	0.0941	0.611	0.4
Rxn #36	AnalytiCon Plate #5	C7	50	0.569	0.0941	0.611	8.1
Rxn #36	AnalytiCon Plate #5	D7	50	0.593	0.0941	0.611	3.5
Rxn #36	AnalytiCon Plate #5	E7	50	0.607	0.0941	0.611	0.8
Rxn #36	AnalytiCon Plate #5	F7	50	0.590	0.0941	0.611	4.1
Rxn #36	AnalytiCon Plate #5	G7	50	0.581	0.0941	0.611	5.8
Rxn #36	AnalytiCon Plate #5	H7: FOC	-	0.613	0.0941	0.611	-0.4
Rxn #36	AnalytiCon Plate #5	A8: NFC	-	0.090	0.0941	0.611	100.8
Rxn #36	AnalytiCon Plate #5	B8	50	0.573	0.0941	0.611	7.4
Rxn #36	AnalytiCon Plate #5	C8	50	0.603	0.0941	0.611	1.5
Rxn #36	AnalytiCon Plate #5	D8	50	0.615	0.0941	0.611	-0.8
Rxn #36	AnalytiCon Plate #5	E8	50	0.523	0.0941	0.611	17.0
Rxn #36	AnalytiCon Plate #5	F8	50	0.620	0.0941	0.611	-1.7
Rxn #36	AnalytiCon Plate #5	G8	50	0.615	0.0941	0.611	-0.8
Rxn #36	AnalytiCon Plate #5	H8: FOC	-	0.616	0.0941	0.611	-1.0
Rxn #36	AnalytiCon Plate #5	A9: NFC	-	0.095	0.0941	0.611	99.8
Rxn #36	AnalytiCon Plate #5	B9	50	0.593	0.0941	0.611	3.5
Rxn #36	AnalytiCon Plate #5	C9	50	0.625	0.0941	0.611	-2.7
Rxn #36	AnalytiCon Plate #5	D9	50	0.620	0.0941	0.611	-1.7
Rxn #36	AnalytiCon Plate #5	E9	50	0.620	0.0941	0.611	-1.7
Rxn #36	AnalytiCon Plate #5	F9	50	0.598	0.0941	0.611	2.5
Rxn #36	AnalytiCon Plate #5	G9	50	0.611	0.0941	0.611	0.0
Rxn #36	AnalytiCon Plate #5	H9: FOC	-	0.610	0.0941	0.611	0.2
Rxn #36	AnalytiCon Plate #5	A10: NFC	-	0.108	0.0941	0.611	97.3
Rxn #36	AnalytiCon Plate #5	B10	50	0.617	0.0941	0.611	-1.2
Rxn #36	AnalytiCon Plate #5	C10	50	0.617	0.0941	0.611	-1.2
Rxn #36	AnalytiCon Plate #5	D10	50	0.628	0.0941	0.611	-3.3
Rxn #36	AnalytiCon Plate #5	E10	50	0.615	0.0941	0.611	-0.8
Rxn #36	AnalytiCon Plate #5	F10	50	0.627	0.0941	0.611	-3.1
Rxn #36	AnalytiCon Plate #5	G10	50	0.228	0.0941	0.611	74.1
Rxn #36	AnalytiCon Plate #5	H10: FOC	-	0.639	0.0941	0.611	-5.4
Rxn #36	AnalytiCon Plate #5	A11: NFC	-	0.110	0.0941	0.611	96.9
Rxn #36	AnalytiCon Plate #5	B11	50	0.598	0.0941	0.611	2.5
Rxn #36	AnalytiCon Plate #5	C11	50	0.513	0.0941	0.611	19.0
Rxn #36	AnalytiCon Plate #5	D11	50	0.621	0.0941	0.611	-1.9

S3 Table. Data from Screening of Phytochemical Library for Inhibition of KHKC Activity.

Rxn ID	Sample			Sample	Ave. No Fructose Controls	Ave. Fructose Only Controls	KHKC
	Phytochemical Plate	Well	Conc (μM)	(ΔOD_{340nm})	(ΔOD_{340nm})	(ΔOD_{340nm})	Inhibition (%)
Rxn #36	AnalytiCon Plate #5	E11	50	0.635	0.0941	0.611	-4.6
Rxn #36	AnalytiCon Plate #5	F11	50	0.616	0.0941	0.611	-1.0
Rxn #36	AnalytiCon Plate #5	G11	50	0.610	0.0941	0.611	0.2
Rxn #36	AnalytiCon Plate #5	H11: FOC	-	0.631	0.0941	0.611	-3.9
Rxn #36	AnalytiCon Plate #5	A12: NFC	-	0.110	0.0941	0.611	96.9
Rxn #36	AnalytiCon Plate #5	H12: FOC	-	0.602	0.0941	0.611	1.7
Rxn #37	AnalytiCon Plate #6	A1: NFC	-	0.064	0.0954	0.536	107.1
Rxn #37	AnalytiCon Plate #6	B1	50	0.511	0.0954	0.536	5.7
Rxn #37	AnalytiCon Plate #6	C1	50	0.501	0.0954	0.536	7.9
Rxn #37	AnalytiCon Plate #6	D1	50	0.478	0.0954	0.536	13.2
Rxn #37	AnalytiCon Plate #6	E1	50	0.329	0.0954	0.536	47.0
Rxn #37	AnalytiCon Plate #6	F1	50	0.432	0.0954	0.536	23.6
Rxn #37	AnalytiCon Plate #6	G1	50	0.484	0.0954	0.536	11.8
Rxn #37	AnalytiCon Plate #6	H1: FOC	-	0.505	0.0954	0.536	7.0
Rxn #37	AnalytiCon Plate #6	A2: NFC	-	0.131	0.0954	0.536	91.9
Rxn #37	AnalytiCon Plate #6	B2	50	0.552	0.0954	0.536	-3.6
Rxn #37	AnalytiCon Plate #6	C2	50	0.487	0.0954	0.536	11.1
Rxn #37	AnalytiCon Plate #6	D2	50	0.527	0.0954	0.536	2.0
Rxn #37	AnalytiCon Plate #6	E2	50	0.514	0.0954	0.536	5.0
Rxn #37	AnalytiCon Plate #6	F2	50	0.507	0.0954	0.536	6.6
Rxn #37	AnalytiCon Plate #6	G2	50	0.459	0.0954	0.536	17.5
Rxn #37	AnalytiCon Plate #6	H2: FOC	-	0.538	0.0954	0.536	-0.5
Rxn #37	AnalytiCon Plate #6	A3: NFC	-	0.094	0.0954	0.536	100.3
Rxn #37	AnalytiCon Plate #6	B3	50	0.546	0.0954	0.536	-2.3
Rxn #37	AnalytiCon Plate #6	C3	50	0.511	0.0954	0.536	5.7
Rxn #37	AnalytiCon Plate #6	D3	50	0.496	0.0954	0.536	9.1
Rxn #37	AnalytiCon Plate #6	E3	50	0.463	0.0954	0.536	16.6
Rxn #37	AnalytiCon Plate #6	F3	50	0.529	0.0954	0.536	1.6
Rxn #37	AnalytiCon Plate #6	G3	50	0.503	0.0954	0.536	7.5
Rxn #37	AnalytiCon Plate #6	H3: FOC	-	0.541	0.0954	0.536	-1.1
Rxn #37	AnalytiCon Plate #6	A4: NFC	-	0.087	0.0954	0.536	101.9
Rxn #37	AnalytiCon Plate #6	B4	50	0.544	0.0954	0.536	-1.8
Rxn #37	AnalytiCon Plate #6	C4	50	0.534	0.0954	0.536	0.5
Rxn #37	AnalytiCon Plate #6	D4	50	0.473	0.0954	0.536	14.3
Rxn #37	AnalytiCon Plate #6	E4	50	0.514	0.0954	0.536	5.0
Rxn #37	AnalytiCon Plate #6	F4	50	0.512	0.0954	0.536	5.4
Rxn #37	AnalytiCon Plate #6	G4	50	0.517	0.0954	0.536	4.3
Rxn #37	AnalytiCon Plate #6	H4: FOC	-	0.538	0.0954	0.536	-0.5
Rxn #37	AnalytiCon Plate #6	A5: NFC	-	0.094	0.0954	0.536	100.3
Rxn #37	AnalytiCon Plate #6	B5: NFC	-	0.081	0.0954	0.536	103.3
Rxn #37	AnalytiCon Plate #6	C5: NFC	-	0.072	0.0954	0.536	105.3
Rxn #37	AnalytiCon Plate #6	D5: NFC	-	0.093	0.0954	0.536	100.5
Rxn #37	AnalytiCon Plate #6	E5: FOC	-	0.537	0.0954	0.536	-0.2
Rxn #37	AnalytiCon Plate #6	F5: FOC	-	0.527	0.0954	0.536	2.0
Rxn #37	AnalytiCon Plate #6	G5: FOC	-	0.530	0.0954	0.536	1.4
Rxn #37	AnalytiCon Plate #6	H5: FOC	-	0.552	0.0954	0.536	-3.6
Rxn #37	AnalytiCon Plate #6	A6: NFC	-	0.099	0.0954	0.536	99.2
Rxn #37	AnalytiCon Plate #6	B6	50	0.548	0.0954	0.536	-2.7
Rxn #37	AnalytiCon Plate #6	C6	50	0.543	0.0954	0.536	-1.6
Rxn #37	AnalytiCon Plate #6	D6	50	0.526	0.0954	0.536	2.3
Rxn #37	AnalytiCon Plate #6	E6	50	0.529	0.0954	0.536	1.6
Rxn #37	AnalytiCon Plate #6	F6	50	0.518	0.0954	0.536	4.1
Rxn #37	AnalytiCon Plate #6	G6	50	0.509	0.0954	0.536	6.1
Rxn #37	AnalytiCon Plate #6	H6: FOC	-	0.547	0.0954	0.536	-2.5
Rxn #37	AnalytiCon Plate #6	A7: NFC	-	0.095	0.0954	0.536	100.1
Rxn #37	AnalytiCon Plate #6	B7	50	0.542	0.0954	0.536	-1.4
Rxn #37	AnalytiCon Plate #6	C7	50	0.545	0.0954	0.536	-2.0
Rxn #37	AnalytiCon Plate #6	D7	50	0.508	0.0954	0.536	6.4
Rxn #37	AnalytiCon Plate #6	E7	50	0.530	0.0954	0.536	1.4
Rxn #37	AnalytiCon Plate #6	F7	50	0.462	0.0954	0.536	16.8
Rxn #37	AnalytiCon Plate #6	G7	50	0.495	0.0954	0.536	9.3
Rxn #37	AnalytiCon Plate #6	H7: FOC	-	0.543	0.0954	0.536	-1.6
Rxn #37	AnalytiCon Plate #6	A8: NFC	-	0.091	0.0954	0.536	101.0
Rxn #37	AnalytiCon Plate #6	B8	50	0.530	0.0954	0.536	1.4
Rxn #37	AnalytiCon Plate #6	C8	50	0.543	0.0954	0.536	-1.6
Rxn #37	AnalytiCon Plate #6	D8	50	0.483	0.0954	0.536	12.0
Rxn #37	AnalytiCon Plate #6	E8	50	0.482	0.0954	0.536	12.3
Rxn #37	AnalytiCon Plate #6	F8	50	0.486	0.0954	0.536	11.3
Rxn #37	AnalytiCon Plate #6	G8	50	0.474	0.0954	0.536	14.1
Rxn #37	AnalytiCon Plate #6	H8: FOC	-	0.547	0.0954	0.536	-2.5
Rxn #37	AnalytiCon Plate #6	A9: NFC	-	0.091	0.0954	0.536	101.0
Rxn #37	AnalytiCon Plate #6	B9	50	0.540	0.0954	0.536	-0.9
Rxn #37	AnalytiCon Plate #6	C9	50	0.530	0.0954	0.536	1.4
Rxn #37	AnalytiCon Plate #6	D9	50	0.515	0.0954	0.536	4.8

S3 Table. Data from Screening of Phytochemical Library for Inhibition of KHKC Activity.

Rxn ID	Sample			Sample	Ave. No Fructose Controls	Ave. Fructose Only Controls	KHKC
	Phytochemical Plate	Well	Conc (μM)	(ΔOD_{340nm})	(ΔOD_{340nm})	(ΔOD_{340nm})	Inhibition (%)
Rxn #37	AnalytiCon Plate #6	E9	50	0.512	0.0954	0.536	5.4
Rxn #37	AnalytiCon Plate #6	F9	50	0.521	0.0954	0.536	3.4
Rxn #37	AnalytiCon Plate #6	G9	50	0.471	0.0954	0.536	14.8
Rxn #37	AnalytiCon Plate #6	H9: FOC	-	0.548	0.0954	0.536	-2.7
Rxn #37	AnalytiCon Plate #6	A10: NFC	-	0.095	0.0954	0.536	100.1
Rxn #37	AnalytiCon Plate #6	B10	50	0.458	0.0954	0.536	17.7
Rxn #37	AnalytiCon Plate #6	C10	50	0.526	0.0954	0.536	2.3
Rxn #37	AnalytiCon Plate #6	D10	50	0.517	0.0954	0.536	4.3
Rxn #37	AnalytiCon Plate #6	E10	50	0.514	0.0954	0.536	5.0
Rxn #37	AnalytiCon Plate #6	F10	50	0.519	0.0954	0.536	3.9
Rxn #37	AnalytiCon Plate #6	G10	50	0.478	0.0954	0.536	13.2
Rxn #37	AnalytiCon Plate #6	H10: FOC	-	0.557	0.0954	0.536	-4.8
Rxn #37	AnalytiCon Plate #6	A11: NFC	-	0.142	0.0954	0.536	89.4
Rxn #37	AnalytiCon Plate #6	B11	50	0.549	0.0954	0.536	-3.0
Rxn #37	AnalytiCon Plate #6	C11	50	0.456	0.0954	0.536	18.2
Rxn #37	AnalytiCon Plate #6	D11	50	0.518	0.0954	0.536	4.1
Rxn #37	AnalytiCon Plate #6	E11	50	0.472	0.0954	0.536	14.5
Rxn #37	AnalytiCon Plate #6	F11	50	0.532	0.0954	0.536	0.9
Rxn #37	AnalytiCon Plate #6	G11	50	0.531	0.0954	0.536	1.1
Rxn #37	AnalytiCon Plate #6	H11: FOC	-	0.542	0.0954	0.536	-1.4
Rxn #37	AnalytiCon Plate #6	A12: NFC	-	0.102	0.0954	0.536	98.5
Rxn #37	AnalytiCon Plate #6	H12: FOC	-	0.550	0.0954	0.536	-3.2
Rxn #38	AnalytiCon Plate #7	A1: NFC	-	0.071	0.0664	0.621	99.2
Rxn #38	AnalytiCon Plate #7	B1	50	0.580	0.0664	0.621	7.4
Rxn #38	AnalytiCon Plate #7	C1	50	0.574	0.0664	0.621	8.5
Rxn #38	AnalytiCon Plate #7	D1	50	0.520	0.0664	0.621	18.2
Rxn #38	AnalytiCon Plate #7	E1	50	0.527	0.0664	0.621	16.9
Rxn #38	AnalytiCon Plate #7	F1	50	0.565	0.0664	0.621	10.1
Rxn #38	AnalytiCon Plate #7	G1	50	0.587	0.0664	0.621	6.1
Rxn #38	AnalytiCon Plate #7	H1: FOC	-	0.609	0.0664	0.621	2.2
Rxn #38	AnalytiCon Plate #7	A2: NFC	-	0.086	0.0664	0.621	96.5
Rxn #38	AnalytiCon Plate #7	B2	50	0.636	0.0664	0.621	-2.7
Rxn #38	AnalytiCon Plate #7	C2	50	0.613	0.0664	0.621	1.4
Rxn #38	AnalytiCon Plate #7	D2	50	0.597	0.0664	0.621	4.3
Rxn #38	AnalytiCon Plate #7	E2	50	0.570	0.0664	0.621	9.2
Rxn #38	AnalytiCon Plate #7	F2	50	0.623	0.0664	0.621	-0.4
Rxn #38	AnalytiCon Plate #7	G2	50	0.597	0.0664	0.621	4.3
Rxn #38	AnalytiCon Plate #7	H2: FOC	-	0.617	0.0664	0.621	0.7
Rxn #38	AnalytiCon Plate #7	A3: NFC	-	0.063	0.0664	0.621	100.6
Rxn #38	AnalytiCon Plate #7	B3	50	0.554	0.0664	0.621	12.1
Rxn #38	AnalytiCon Plate #7	C3	50	0.631	0.0664	0.621	-1.8
Rxn #38	AnalytiCon Plate #7	D3	50	0.574	0.0664	0.621	8.5
Rxn #38	AnalytiCon Plate #7	E3	50	0.595	0.0664	0.621	4.7
Rxn #38	AnalytiCon Plate #7	F3	50	0.593	0.0664	0.621	5.0
Rxn #38	AnalytiCon Plate #7	G3	50	0.539	0.0664	0.621	14.8
Rxn #38	AnalytiCon Plate #7	H3: FOC	-	0.622	0.0664	0.621	-0.2
Rxn #38	AnalytiCon Plate #7	A4: NFC	-	0.059	0.0664	0.621	101.3
Rxn #38	AnalytiCon Plate #7	B4	50	0.608	0.0664	0.621	2.3
Rxn #38	AnalytiCon Plate #7	C4	50	0.568	0.0664	0.621	9.6
Rxn #38	AnalytiCon Plate #7	D4	50	0.540	0.0664	0.621	14.6
Rxn #38	AnalytiCon Plate #7	E4	50	0.581	0.0664	0.621	7.2
Rxn #38	AnalytiCon Plate #7	F4	50	0.607	0.0664	0.621	2.5
Rxn #38	AnalytiCon Plate #7	G4	50	0.581	0.0664	0.621	7.2
Rxn #38	AnalytiCon Plate #7	H4: FOC	-	0.616	0.0664	0.621	0.9
Rxn #38	AnalytiCon Plate #7	A5: NFC	-	0.072	0.0664	0.621	99.0
Rxn #38	AnalytiCon Plate #7	B5: NFC	-	0.065	0.0664	0.621	100.3
Rxn #38	AnalytiCon Plate #7	C5: NFC	-	0.076	0.0664	0.621	98.3
Rxn #38	AnalytiCon Plate #7	D5: NFC	-	0.074	0.0664	0.621	98.6
Rxn #38	AnalytiCon Plate #7	E5: FOC	-	0.618	0.0664	0.621	0.5
Rxn #38	AnalytiCon Plate #7	F5: FOC	-	0.613	0.0664	0.621	1.4
Rxn #38	AnalytiCon Plate #7	G5: FOC	-	0.625	0.0664	0.621	-0.7
Rxn #38	AnalytiCon Plate #7	H5: FOC	-	0.630	0.0664	0.621	-1.6
Rxn #38	AnalytiCon Plate #7	A6: NFC	-	0.066	0.0664	0.621	100.1
Rxn #38	AnalytiCon Plate #7	B6	50	0.574	0.0664	0.621	8.5
Rxn #38	AnalytiCon Plate #7	C6	50	0.609	0.0664	0.621	2.2
Rxn #38	AnalytiCon Plate #7	D6	50	0.597	0.0664	0.621	4.3
Rxn #38	AnalytiCon Plate #7	E6	50	0.607	0.0664	0.621	2.5
Rxn #38	AnalytiCon Plate #7	F6	50	0.582	0.0664	0.621	7.0
Rxn #38	AnalytiCon Plate #7	G6	50	0.550	0.0664	0.621	12.8
Rxn #38	AnalytiCon Plate #7	H6: FOC	-	0.629	0.0664	0.621	-1.4
Rxn #38	AnalytiCon Plate #7	A7: NFC	-	0.069	0.0664	0.621	99.5
Rxn #38	AnalytiCon Plate #7	B7	50	0.626	0.0664	0.621	-0.9
Rxn #38	AnalytiCon Plate #7	C7	50	0.573	0.0664	0.621	8.7
Rxn #38	AnalytiCon Plate #7	D7	50	0.542	0.0664	0.621	14.2

S3 Table. Data from Screening of Phytochemical Library for Inhibition of KHKC Activity.

Rxn ID	Sample			Sample	Ave. No Fructose Controls	Ave. Fructose Only Controls	KHKC
	Phytochemical Plate	Well	Conc (μM)	(ΔOD_{340nm})	(ΔOD_{340nm})	(ΔOD_{340nm})	Inhibition (%)
Rxn #38	AnalytiCon Plate #7	E7	50	0.553	0.0664	0.621	12.3
Rxn #38	AnalytiCon Plate #7	F7	50	0.550	0.0664	0.621	12.8
Rxn #38	AnalytiCon Plate #7	G7	50	0.582	0.0664	0.621	7.0
Rxn #38	AnalytiCon Plate #7	H7: FOC	-	0.626	0.0664	0.621	-0.9
Rxn #38	AnalytiCon Plate #7	A8: NFC	-	0.051	0.0664	0.621	102.8
Rxn #38	AnalytiCon Plate #7	B8	50	0.620	0.0664	0.621	0.2
Rxn #38	AnalytiCon Plate #7	C8	50	0.585	0.0664	0.621	6.5
Rxn #38	AnalytiCon Plate #7	D8	50	0.578	0.0664	0.621	7.8
Rxn #38	AnalytiCon Plate #7	E8	50	0.458	0.0664	0.621	29.4
Rxn #38	AnalytiCon Plate #7	F8	50	0.456	0.0664	0.621	29.8
Rxn #38	AnalytiCon Plate #7	G8	50	0.550	0.0664	0.621	12.8
Rxn #38	AnalytiCon Plate #7	H8: FOC	-	0.621	0.0664	0.621	0.0
Rxn #38	AnalytiCon Plate #7	A9: NFC	-	0.066	0.0664	0.621	100.1
Rxn #38	AnalytiCon Plate #7	B9	50	0.591	0.0664	0.621	5.4
Rxn #38	AnalytiCon Plate #7	C9	50	0.580	0.0664	0.621	7.4
Rxn #38	AnalytiCon Plate #7	D9	50	0.560	0.0664	0.621	11.0
Rxn #38	AnalytiCon Plate #7	E9	50	0.578	0.0664	0.621	7.8
Rxn #38	AnalytiCon Plate #7	F9	50	0.581	0.0664	0.621	7.2
Rxn #38	AnalytiCon Plate #7	G9	50	0.585	0.0664	0.621	6.5
Rxn #38	AnalytiCon Plate #7	H9: FOC	-	0.623	0.0664	0.621	-0.4
Rxn #38	AnalytiCon Plate #7	A10: NFC	-	0.064	0.0664	0.621	100.4
Rxn #38	AnalytiCon Plate #7	B10	50	0.600	0.0664	0.621	3.8
Rxn #38	AnalytiCon Plate #7	C10	50	0.584	0.0664	0.621	6.7
Rxn #38	AnalytiCon Plate #7	D10	50	0.568	0.0664	0.621	9.6
Rxn #38	AnalytiCon Plate #7	E10	50	0.557	0.0664	0.621	11.5
Rxn #38	AnalytiCon Plate #7	F10	50	0.583	0.0664	0.621	6.9
Rxn #38	AnalytiCon Plate #7	G10	50	0.545	0.0664	0.621	13.7
Rxn #38	AnalytiCon Plate #7	H10: FOC	-	0.632	0.0664	0.621	-2.0
Rxn #38	AnalytiCon Plate #7	A11: NFC	-	0.061	0.0664	0.621	101.0
Rxn #38	AnalytiCon Plate #7	B11	50	0.597	0.0664	0.621	4.3
Rxn #38	AnalytiCon Plate #7	C11	50	0.548	0.0664	0.621	13.2
Rxn #38	AnalytiCon Plate #7	D11	50	0.526	0.0664	0.621	17.1
Rxn #38	AnalytiCon Plate #7	E11	50	0.544	0.0664	0.621	13.9
Rxn #38	AnalytiCon Plate #7	F11	50	0.578	0.0664	0.621	7.8
Rxn #38	AnalytiCon Plate #7	G11	50	0.593	0.0664	0.621	5.0
Rxn #38	AnalytiCon Plate #7	H11: FOC	-	0.649	0.0664	0.621	-5.0
Rxn #38	AnalytiCon Plate #7	A12: NFC	-	0.053	0.0664	0.621	102.4
Rxn #38	AnalytiCon Plate #7	H12: FOC	-	0.630	0.0664	0.621	-1.6
Rxn #39	AnalytiCon Plate #8	A1: NFC	-	0.080	0.0789	0.392	99.6
Rxn #39	AnalytiCon Plate #8	B1	50	0.351	0.0789	0.392	13.1
Rxn #39	AnalytiCon Plate #8	C1	50	0.310	0.0789	0.392	26.2
Rxn #39	AnalytiCon Plate #8	D1	50	0.364	0.0789	0.392	8.9
Rxn #39	AnalytiCon Plate #8	E1	50	0.351	0.0789	0.392	13.1
Rxn #39	AnalytiCon Plate #8	F1	50	0.297	0.0789	0.392	30.3
Rxn #39	AnalytiCon Plate #8	G1	50	0.363	0.0789	0.392	9.3
Rxn #39	AnalytiCon Plate #8	H1: FOC	-	0.374	0.0789	0.392	5.7
Rxn #39	AnalytiCon Plate #8	A2: NFC	-	0.080	0.0789	0.392	99.6
Rxn #39	AnalytiCon Plate #8	B2	50	0.389	0.0789	0.392	1.0
Rxn #39	AnalytiCon Plate #8	C2	50	0.338	0.0789	0.392	17.2
Rxn #39	AnalytiCon Plate #8	D2	50	0.360	0.0789	0.392	10.2
Rxn #39	AnalytiCon Plate #8	E2	50	0.386	0.0789	0.392	1.9
Rxn #39	AnalytiCon Plate #8	F2	50	0.378	0.0789	0.392	4.5
Rxn #39	AnalytiCon Plate #8	G2	50	0.386	0.0789	0.392	1.9
Rxn #39	AnalytiCon Plate #8	H2: FOC	-	0.386	0.0789	0.392	1.9
Rxn #39	AnalytiCon Plate #8	A3: NFC	-	0.088	0.0789	0.392	97.1
Rxn #39	AnalytiCon Plate #8	B3	50	0.385	0.0789	0.392	2.2
Rxn #39	AnalytiCon Plate #8	C3	50	0.369	0.0789	0.392	7.3
Rxn #39	AnalytiCon Plate #8	D3	50	0.366	0.0789	0.392	8.3
Rxn #39	AnalytiCon Plate #8	E3	50	0.358	0.0789	0.392	10.9
Rxn #39	AnalytiCon Plate #8	F3	50	0.393	0.0789	0.392	-0.3
Rxn #39	AnalytiCon Plate #8	G3	50	0.383	0.0789	0.392	2.9
Rxn #39	AnalytiCon Plate #8	H3: FOC	-	0.394	0.0789	0.392	-0.6
Rxn #39	AnalytiCon Plate #8	A4: NFC	-	0.075	0.0789	0.392	101.2
Rxn #39	AnalytiCon Plate #8	B4	50	0.371	0.0789	0.392	6.7
Rxn #39	AnalytiCon Plate #8	C4	50	0.387	0.0789	0.392	1.6
Rxn #39	AnalytiCon Plate #8	D4	50	0.390	0.0789	0.392	0.6
Rxn #39	AnalytiCon Plate #8	E4	50	0.370	0.0789	0.392	7.0
Rxn #39	AnalytiCon Plate #8	F4	50	0.391	0.0789	0.392	0.3
Rxn #39	AnalytiCon Plate #8	G4	50	0.389	0.0789	0.392	1.0
Rxn #39	AnalytiCon Plate #8	H4: FOC	-	0.395	0.0789	0.392	-1.0
Rxn #39	AnalytiCon Plate #8	A5: NFC	-	0.088	0.0789	0.392	97.1
Rxn #39	AnalytiCon Plate #8	B5: NFC	-	0.071	0.0789	0.392	102.5
Rxn #39	AnalytiCon Plate #8	C5: NFC	-	0.052	0.0789	0.392	108.6
Rxn #39	AnalytiCon Plate #8	D5: NFC	-	0.065	0.0789	0.392	104.4

S3 Table. Data from Screening of Phytochemical Library for Inhibition of KHKC Activity.

Rxn ID	Sample			Sample (ΔOD_{340nm})	Ave. No Fructose Controls (ΔOD_{340nm})	Ave. Fructose Only Controls (ΔOD_{340nm})	KHKC Inhibition (%)
	Phytochemical Plate	Well	Conc (μM)				
Rxn #39	AnalytiCon Plate #8	E5: FOC	-	0.384	0.0789	0.392	2.6
Rxn #39	AnalytiCon Plate #8	F5: FOC	-	0.405	0.0789	0.392	-4.2
Rxn #39	AnalytiCon Plate #8	G5: FOC	-	0.395	0.0789	0.392	-1.0
Rxn #39	AnalytiCon Plate #8	H5: FOC	-	0.395	0.0789	0.392	-1.0
Rxn #39	AnalytiCon Plate #8	A6: NFC	-	0.071	0.0789	0.392	102.5
Rxn #39	AnalytiCon Plate #8	B6	50	0.359	0.0789	0.392	10.5
Rxn #39	AnalytiCon Plate #8	C6	50	0.386	0.0789	0.392	1.9
Rxn #39	AnalytiCon Plate #8	D6	50	0.395	0.0789	0.392	-1.0
Rxn #39	AnalytiCon Plate #8	E6	50	0.362	0.0789	0.392	9.6
Rxn #39	AnalytiCon Plate #8	F6	50	0.390	0.0789	0.392	0.6
Rxn #39	AnalytiCon Plate #8	G6	50	0.393	0.0789	0.392	-0.3
Rxn #39	AnalytiCon Plate #8	H6: FOC	-	0.403	0.0789	0.392	-3.5
Rxn #39	AnalytiCon Plate #8	A7: NFC	-	0.075	0.0789	0.392	101.2
Rxn #39	AnalytiCon Plate #8	B7	50	0.359	0.0789	0.392	10.5
Rxn #39	AnalytiCon Plate #8	C7	50	0.387	0.0789	0.392	1.6
Rxn #39	AnalytiCon Plate #8	D7	50	0.380	0.0789	0.392	3.8
Rxn #39	AnalytiCon Plate #8	E7	50	0.389	0.0789	0.392	1.0
Rxn #39	AnalytiCon Plate #8	F7	50	0.346	0.0789	0.392	14.7
Rxn #39	AnalytiCon Plate #8	G7	50	0.339	0.0789	0.392	16.9
Rxn #39	AnalytiCon Plate #8	H7: FOC	-	0.392	0.0789	0.392	0.0
Rxn #39	AnalytiCon Plate #8	A8: NFC	-	0.082	0.0789	0.392	99.0
Rxn #39	AnalytiCon Plate #8	B8	50	0.350	0.0789	0.392	13.4
Rxn #39	AnalytiCon Plate #8	C8	50	0.393	0.0789	0.392	-0.3
Rxn #39	AnalytiCon Plate #8	D8	50	0.384	0.0789	0.392	2.6
Rxn #39	AnalytiCon Plate #8	E8	50	0.386	0.0789	0.392	1.9
Rxn #39	AnalytiCon Plate #8	F8	50	0.389	0.0789	0.392	1.0
Rxn #39	AnalytiCon Plate #8	G8	50	0.354	0.0789	0.392	12.1
Rxn #39	AnalytiCon Plate #8	H8: FOC	-	0.408	0.0789	0.392	-5.1
Rxn #39	AnalytiCon Plate #8	A9: NFC	-	0.090	0.0789	0.392	96.5
Rxn #39	AnalytiCon Plate #8	B9	50	0.371	0.0789	0.392	6.7
Rxn #39	AnalytiCon Plate #8	C9	50	0.349	0.0789	0.392	13.7
Rxn #39	AnalytiCon Plate #8	D9	50	0.400	0.0789	0.392	-2.6
Rxn #39	AnalytiCon Plate #8	E9	50	0.357	0.0789	0.392	11.2
Rxn #39	AnalytiCon Plate #8	F9	50	0.377	0.0789	0.392	4.8
Rxn #39	AnalytiCon Plate #8	G9	50	0.383	0.0789	0.392	2.9
Rxn #39	AnalytiCon Plate #8	H9: FOC	-	0.398	0.0789	0.392	-1.9
Rxn #39	AnalytiCon Plate #8	A10: NFC	-	0.094	0.0789	0.392	95.2
Rxn #39	AnalytiCon Plate #8	B10	50	0.400	0.0789	0.392	-2.6
Rxn #39	AnalytiCon Plate #8	C10	50	0.382	0.0789	0.392	3.2
Rxn #39	AnalytiCon Plate #8	D10	50	0.392	0.0789	0.392	0.0
Rxn #39	AnalytiCon Plate #8	E10	50	0.356	0.0789	0.392	11.5
Rxn #39	AnalytiCon Plate #8	F10	50	0.324	0.0789	0.392	21.7
Rxn #39	AnalytiCon Plate #8	G10	50	0.332	0.0789	0.392	19.2
Rxn #39	AnalytiCon Plate #8	H10: FOC	-	0.409	0.0789	0.392	-5.4
Rxn #39	AnalytiCon Plate #8	A11: NFC	-	0.084	0.0789	0.392	98.4
Rxn #39	AnalytiCon Plate #8	B11	50	0.334	0.0789	0.392	18.5
Rxn #39	AnalytiCon Plate #8	C11	50	0.344	0.0789	0.392	15.3
Rxn #39	AnalytiCon Plate #8	D11	50	0.309	0.0789	0.392	26.5
Rxn #39	AnalytiCon Plate #8	E11	50	0.388	0.0789	0.392	1.3
Rxn #39	AnalytiCon Plate #8	F11	50	0.381	0.0789	0.392	3.5
Rxn #39	AnalytiCon Plate #8	G11	50	0.395	0.0789	0.392	-1.0
Rxn #39	AnalytiCon Plate #8	H11: FOC	-	0.409	0.0789	0.392	-5.4
Rxn #39	AnalytiCon Plate #8	A12: NFC	-	0.089	0.0789	0.392	96.8
Rxn #39	AnalytiCon Plate #8	H12: FOC	-	0.409	0.0789	0.392	-5.4
Rxn #40	AnalytiCon Plate #9	A1: NFC	-	0.058	0.0629	0.483	101.2
Rxn #40	AnalytiCon Plate #9	B1	50	0.448	0.0629	0.483	8.3
Rxn #40	AnalytiCon Plate #9	C1	50	0.445	0.0629	0.483	9.0
Rxn #40	AnalytiCon Plate #9	D1	50	0.413	0.0629	0.483	16.7
Rxn #40	AnalytiCon Plate #9	E1	50	0.433	0.0629	0.483	11.9
Rxn #40	AnalytiCon Plate #9	F1	50	0.440	0.0629	0.483	10.2
Rxn #40	AnalytiCon Plate #9	G1	50	0.434	0.0629	0.483	11.7
Rxn #40	AnalytiCon Plate #9	H1: FOC	-	0.457	0.0629	0.483	6.2
Rxn #40	AnalytiCon Plate #9	A2: NFC	-	0.062	0.0629	0.483	100.2
Rxn #40	AnalytiCon Plate #9	B2	50	0.453	0.0629	0.483	7.1
Rxn #40	AnalytiCon Plate #9	C2	50	0.420	0.0629	0.483	15.0
Rxn #40	AnalytiCon Plate #9	D2	50	0.430	0.0629	0.483	12.6
Rxn #40	AnalytiCon Plate #9	E2	50	0.401	0.0629	0.483	19.5
Rxn #40	AnalytiCon Plate #9	F2	50	0.443	0.0629	0.483	9.5
Rxn #40	AnalytiCon Plate #9	G2	50	0.452	0.0629	0.483	7.4
Rxn #40	AnalytiCon Plate #9	H2: FOC	-	0.482	0.0629	0.483	0.2
Rxn #40	AnalytiCon Plate #9	A3: NFC	-	0.062	0.0629	0.483	100.2
Rxn #40	AnalytiCon Plate #9	B3	50	0.347	0.0629	0.483	32.4
Rxn #40	AnalytiCon Plate #9	C3	50	0.467	0.0629	0.483	3.8
Rxn #40	AnalytiCon Plate #9	D3	50	0.379	0.0629	0.483	24.8

S3 Table. Data from Screening of Phytochemical Library for Inhibition of KHKC Activity.

Rxn ID	Sample			Sample	Ave. No Fructose Controls	Ave. Fructose Only Controls	KHKC
	Phytochemical Plate	Well	Conc (μM)	(ΔOD_{340nm})	(ΔOD_{340nm})	(ΔOD_{340nm})	Inhibition (%)
Rxn #40	AnalytiCon Plate #9	E3	50	0.461	0.0629	0.483	5.2
Rxn #40	AnalytiCon Plate #9	F3	50	0.458	0.0629	0.483	6.0
Rxn #40	AnalytiCon Plate #9	G3	50	0.451	0.0629	0.483	7.6
Rxn #40	AnalytiCon Plate #9	H3: FOC	-	0.479	0.0629	0.483	1.0
Rxn #40	AnalytiCon Plate #9	A4: NFC	-	0.059	0.0629	0.483	100.9
Rxn #40	AnalytiCon Plate #9	B4	50	0.461	0.0629	0.483	5.2
Rxn #40	AnalytiCon Plate #9	C4	50	0.466	0.0629	0.483	4.0
Rxn #40	AnalytiCon Plate #9	D4	50	0.476	0.0629	0.483	1.7
Rxn #40	AnalytiCon Plate #9	E4	50	0.470	0.0629	0.483	3.1
Rxn #40	AnalytiCon Plate #9	F4	50	0.375	0.0629	0.483	25.7
Rxn #40	AnalytiCon Plate #9	G4	50	0.453	0.0629	0.483	7.1
Rxn #40	AnalytiCon Plate #9	H4: FOC	-	0.483	0.0629	0.483	0.0
Rxn #40	AnalytiCon Plate #9	A5: NFC	-	0.063	0.0629	0.483	100.0
Rxn #40	AnalytiCon Plate #9	B5: NFC	-	0.057	0.0629	0.483	101.4
Rxn #40	AnalytiCon Plate #9	C5: NFC	-	0.055	0.0629	0.483	101.9
Rxn #40	AnalytiCon Plate #9	D5: NFC	-	0.055	0.0629	0.483	101.9
Rxn #40	AnalytiCon Plate #9	E5: FOC	-	0.483	0.0629	0.483	0.0
Rxn #40	AnalytiCon Plate #9	F5: FOC	-	0.482	0.0629	0.483	0.2
Rxn #40	AnalytiCon Plate #9	G5: FOC	-	0.478	0.0629	0.483	1.2
Rxn #40	AnalytiCon Plate #9	H5: FOC	-	0.500	0.0629	0.483	-4.0
Rxn #40	AnalytiCon Plate #9	A6: NFC	-	0.065	0.0629	0.483	99.5
Rxn #40	AnalytiCon Plate #9	B6	50	0.485	0.0629	0.483	-0.5
Rxn #40	AnalytiCon Plate #9	C6	50	0.478	0.0629	0.483	1.2
Rxn #40	AnalytiCon Plate #9	D6	50	0.483	0.0629	0.483	0.0
Rxn #40	AnalytiCon Plate #9	E6	50	0.465	0.0629	0.483	4.3
Rxn #40	AnalytiCon Plate #9	F6	50	0.449	0.0629	0.483	8.1
Rxn #40	AnalytiCon Plate #9	G6	50	0.460	0.0629	0.483	5.5
Rxn #40	AnalytiCon Plate #9	H6: FOC	-	0.488	0.0629	0.483	-1.2
Rxn #40	AnalytiCon Plate #9	A7: NFC	-	0.064	0.0629	0.483	99.7
Rxn #40	AnalytiCon Plate #9	B7	50	0.485	0.0629	0.483	-0.5
Rxn #40	AnalytiCon Plate #9	C7	50	0.413	0.0629	0.483	16.7
Rxn #40	AnalytiCon Plate #9	D7	50	0.460	0.0629	0.483	5.5
Rxn #40	AnalytiCon Plate #9	E7	50	0.383	0.0629	0.483	23.8
Rxn #40	AnalytiCon Plate #9	F7	50	0.457	0.0629	0.483	6.2
Rxn #40	AnalytiCon Plate #9	G7	50	0.468	0.0629	0.483	3.6
Rxn #40	AnalytiCon Plate #9	H7: FOC	-	0.495	0.0629	0.483	-2.9
Rxn #40	AnalytiCon Plate #9	A8: NFC	-	0.071	0.0629	0.483	98.1
Rxn #40	AnalytiCon Plate #9	B8	50	0.493	0.0629	0.483	-2.4
Rxn #40	AnalytiCon Plate #9	C8	50	0.437	0.0629	0.483	10.9
Rxn #40	AnalytiCon Plate #9	D8	50	0.484	0.0629	0.483	-0.2
Rxn #40	AnalytiCon Plate #9	E8	50	0.453	0.0629	0.483	7.1
Rxn #40	AnalytiCon Plate #9	F8	50	0.469	0.0629	0.483	3.3
Rxn #40	AnalytiCon Plate #9	G8	50	0.381	0.0629	0.483	24.3
Rxn #40	AnalytiCon Plate #9	H8: FOC	-	0.491	0.0629	0.483	-1.9
Rxn #40	AnalytiCon Plate #9	A9: NFC	-	0.064	0.0629	0.483	99.7
Rxn #40	AnalytiCon Plate #9	B9	50	0.491	0.0629	0.483	-1.9
Rxn #40	AnalytiCon Plate #9	C9	50	0.464	0.0629	0.483	4.5
Rxn #40	AnalytiCon Plate #9	D9	50	0.410	0.0629	0.483	17.4
Rxn #40	AnalytiCon Plate #9	E9	50	0.492	0.0629	0.483	-2.1
Rxn #40	AnalytiCon Plate #9	F9	50	0.477	0.0629	0.483	1.4
Rxn #40	AnalytiCon Plate #9	G9	50	0.446	0.0629	0.483	8.8
Rxn #40	AnalytiCon Plate #9	H9: FOC	-	0.511	0.0629	0.483	-6.7
Rxn #40	AnalytiCon Plate #9	A10: NFC	-	0.067	0.0629	0.483	99.0
Rxn #40	AnalytiCon Plate #9	B10	50	0.484	0.0629	0.483	-0.2
Rxn #40	AnalytiCon Plate #9	C10	50	0.475	0.0629	0.483	1.9
Rxn #40	AnalytiCon Plate #9	D10	50	0.513	0.0629	0.483	-7.1
Rxn #40	AnalytiCon Plate #9	E10	50	0.477	0.0629	0.483	1.4
Rxn #40	AnalytiCon Plate #9	F10	50	0.466	0.0629	0.483	4.0
Rxn #40	AnalytiCon Plate #9	G10	50	0.481	0.0629	0.483	0.5
Rxn #40	AnalytiCon Plate #9	H10: FOC	-	0.507	0.0629	0.483	-5.7
Rxn #40	AnalytiCon Plate #9	A11: NFC	-	0.068	0.0629	0.483	98.8
Rxn #40	AnalytiCon Plate #9	B11	50	0.483	0.0629	0.483	0.0
Rxn #40	AnalytiCon Plate #9	C11	50	0.515	0.0629	0.483	-7.6
Rxn #40	AnalytiCon Plate #9	D11	50	0.508	0.0629	0.483	-6.0
Rxn #40	AnalytiCon Plate #9	E11	50	0.506	0.0629	0.483	-5.5
Rxn #40	AnalytiCon Plate #9	F11	50	0.492	0.0629	0.483	-2.1
Rxn #40	AnalytiCon Plate #9	G11	50	0.505	0.0629	0.483	-5.2
Rxn #40	AnalytiCon Plate #9	H11: FOC	-	0.507	0.0629	0.483	-5.7
Rxn #40	AnalytiCon Plate #9	A12: NFC	-	0.073	0.0629	0.483	97.6
Rxn #40	AnalytiCon Plate #9	H12: FOC	-	0.521	0.0629	0.483	-9.0
Rxn #41	AnalytiCon Plate #10	A1: NFC	-	0.075	0.0787	0.601	100.7
Rxn #41	AnalytiCon Plate #10	B1	50	0.572	0.0787	0.601	5.6
Rxn #41	AnalytiCon Plate #10	C1	50	0.487	0.0787	0.601	21.8
Rxn #41	AnalytiCon Plate #10	D1	50	0.574	0.0787	0.601	5.2

S3 Table. Data from Screening of Phytochemical Library for Inhibition of KHKC Activity.

Rxn ID	Sample			Sample	Ave. No Fructose Controls	Ave. Fructose Only Controls	KHKC
	Phytochemical Plate	Well	Conc (μM)	(ΔOD_{340nm})	(ΔOD_{340nm})	(ΔOD_{340nm})	Inhibition (%)
Rxn #41	AnalytiCon Plate #10	E1	50	0.525	0.0787	0.601	14.6
Rxn #41	AnalytiCon Plate #10	F1	50	0.523	0.0787	0.601	14.9
Rxn #41	AnalytiCon Plate #10	G1	50	0.552	0.0787	0.601	9.4
Rxn #41	AnalytiCon Plate #10	H1: FOC	-	0.571	0.0787	0.601	5.7
Rxn #41	AnalytiCon Plate #10	A2: NFC	-	0.081	0.0787	0.601	99.6
Rxn #41	AnalytiCon Plate #10	B2	50	0.603	0.0787	0.601	-0.4
Rxn #41	AnalytiCon Plate #10	C2	50	0.596	0.0787	0.601	1.0
Rxn #41	AnalytiCon Plate #10	D2	50	0.595	0.0787	0.601	1.1
Rxn #41	AnalytiCon Plate #10	E2	50	0.587	0.0787	0.601	2.7
Rxn #41	AnalytiCon Plate #10	F2	50	0.586	0.0787	0.601	2.9
Rxn #41	AnalytiCon Plate #10	G2	50	0.565	0.0787	0.601	6.9
Rxn #41	AnalytiCon Plate #10	H2: FOC	-	0.600	0.0787	0.601	0.2
Rxn #41	AnalytiCon Plate #10	A3: NFC	-	0.082	0.0787	0.601	99.4
Rxn #41	AnalytiCon Plate #10	B3	50	0.641	0.0787	0.601	-7.7
Rxn #41	AnalytiCon Plate #10	C3	50	0.633	0.0787	0.601	-6.1
Rxn #41	AnalytiCon Plate #10	D3	50	0.585	0.0787	0.601	3.1
Rxn #41	AnalytiCon Plate #10	E3	50	0.560	0.0787	0.601	7.8
Rxn #41	AnalytiCon Plate #10	F3	50	0.568	0.0787	0.601	6.3
Rxn #41	AnalytiCon Plate #10	G3	50	0.567	0.0787	0.601	6.5
Rxn #41	AnalytiCon Plate #10	H3: FOC	-	0.602	0.0787	0.601	-0.2
Rxn #41	AnalytiCon Plate #10	A4: NFC	-	0.083	0.0787	0.601	99.2
Rxn #41	AnalytiCon Plate #10	B4	50	0.600	0.0787	0.601	0.2
Rxn #41	AnalytiCon Plate #10	C4	50	0.611	0.0787	0.601	-1.9
Rxn #41	AnalytiCon Plate #10	D4	50	0.592	0.0787	0.601	1.7
Rxn #41	AnalytiCon Plate #10	E4	50	0.565	0.0787	0.601	6.9
Rxn #41	AnalytiCon Plate #10	F4	50	0.452	0.0787	0.601	28.5
Rxn #41	AnalytiCon Plate #10	G4	50	0.537	0.0787	0.601	12.3
Rxn #41	AnalytiCon Plate #10	H4: FOC	-	0.603	0.0787	0.601	-0.4
Rxn #41	AnalytiCon Plate #10	A5: NFC	-	0.077	0.0787	0.601	100.3
Rxn #41	AnalytiCon Plate #10	B5: NFC	-	0.076	0.0787	0.601	100.5
Rxn #41	AnalytiCon Plate #10	C5: NFC	-	0.072	0.0787	0.601	101.3
Rxn #41	AnalytiCon Plate #10	D5: NFC	-	0.072	0.0787	0.601	101.3
Rxn #41	AnalytiCon Plate #10	E5: FOC	-	0.592	0.0787	0.601	1.7
Rxn #41	AnalytiCon Plate #10	F5: FOC	-	0.605	0.0787	0.601	-0.8
Rxn #41	AnalytiCon Plate #10	G5: FOC	-	0.596	0.0787	0.601	1.0
Rxn #41	AnalytiCon Plate #10	H5: FOC	-	0.614	0.0787	0.601	-2.5
Rxn #41	AnalytiCon Plate #10	A6: NFC	-	0.081	0.0787	0.601	99.6
Rxn #41	AnalytiCon Plate #10	B6	50	0.584	0.0787	0.601	3.3
Rxn #41	AnalytiCon Plate #10	C6	50	0.595	0.0787	0.601	1.1
Rxn #41	AnalytiCon Plate #10	D6	50	0.626	0.0787	0.601	-4.8
Rxn #41	AnalytiCon Plate #10	E6	50	0.560	0.0787	0.601	7.8
Rxn #41	AnalytiCon Plate #10	F6	50	0.594	0.0787	0.601	1.3
Rxn #41	AnalytiCon Plate #10	G6	50	0.586	0.0787	0.601	2.9
Rxn #41	AnalytiCon Plate #10	H6: FOC	-	0.614	0.0787	0.601	-2.5
Rxn #41	AnalytiCon Plate #10	A7: NFC	-	0.081	0.0787	0.601	99.6
Rxn #41	AnalytiCon Plate #10	B7	50	0.605	0.0787	0.601	-0.8
Rxn #41	AnalytiCon Plate #10	C7	50	0.612	0.0787	0.601	-2.1
Rxn #41	AnalytiCon Plate #10	D7	50	0.512	0.0787	0.601	17.0
Rxn #41	AnalytiCon Plate #10	E7	50	0.550	0.0787	0.601	9.8
Rxn #41	AnalytiCon Plate #10	F7	50	0.576	0.0787	0.601	4.8
Rxn #41	AnalytiCon Plate #10	G7	50	0.599	0.0787	0.601	0.4
Rxn #41	AnalytiCon Plate #10	H7: FOC	-	0.609	0.0787	0.601	-1.5
Rxn #41	AnalytiCon Plate #10	A8: NFC	-	0.075	0.0787	0.601	100.7
Rxn #41	AnalytiCon Plate #10	B8	50	0.534	0.0787	0.601	12.8
Rxn #41	AnalytiCon Plate #10	C8	50	0.539	0.0787	0.601	11.9
Rxn #41	AnalytiCon Plate #10	D8	50	0.583	0.0787	0.601	3.4
Rxn #41	AnalytiCon Plate #10	E8	50	0.583	0.0787	0.601	3.4
Rxn #41	AnalytiCon Plate #10	F8	50	0.597	0.0787	0.601	0.8
Rxn #41	AnalytiCon Plate #10	G8	50	0.594	0.0787	0.601	1.3
Rxn #41	AnalytiCon Plate #10	H8: FOC	-	0.613	0.0787	0.601	-2.3
Rxn #41	AnalytiCon Plate #10	A9: NFC	-	0.078	0.0787	0.601	100.1
Rxn #41	AnalytiCon Plate #10	B9	50	0.572	0.0787	0.601	5.6
Rxn #41	AnalytiCon Plate #10	C9	50	0.461	0.0787	0.601	26.8
Rxn #41	AnalytiCon Plate #10	D9	50	0.572	0.0787	0.601	5.6
Rxn #41	AnalytiCon Plate #10	E9	50	0.583	0.0787	0.601	3.4
Rxn #41	AnalytiCon Plate #10	F9	50	0.576	0.0787	0.601	4.8
Rxn #41	AnalytiCon Plate #10	G9	50	0.578	0.0787	0.601	4.4
Rxn #41	AnalytiCon Plate #10	H9: FOC	-	0.603	0.0787	0.601	-0.4
Rxn #41	AnalytiCon Plate #10	A10: NFC	-	0.080	0.0787	0.601	99.8
Rxn #41	AnalytiCon Plate #10	B10	50	0.567	0.0787	0.601	6.5
Rxn #41	AnalytiCon Plate #10	C10	50	0.519	0.0787	0.601	15.7
Rxn #41	AnalytiCon Plate #10	D10	50	0.579	0.0787	0.601	4.2
Rxn #41	AnalytiCon Plate #10	E10	50	0.570	0.0787	0.601	5.9
Rxn #41	AnalytiCon Plate #10	F10	50	0.584	0.0787	0.601	3.3

S3 Table. Data from Screening of Phytochemical Library for Inhibition of KHKC Activity.

Rxn ID	Sample			Sample	Ave. No Fructose Controls	Ave. Fructose Only Controls	KHKC
	Phytochemical Plate	Well	Conc (μM)	(ΔOD_{340nm})	(ΔOD_{340nm})	(ΔOD_{340nm})	Inhibition (%)
Rxn #41	AnalytiCon Plate #10	G10	50	0.608	0.0787	0.601	-1.3
Rxn #41	AnalytiCon Plate #10	H10: FOC	-	0.607	0.0787	0.601	-1.1
Rxn #41	AnalytiCon Plate #10	A11: NFC	-	0.079	0.0787	0.601	99.9
Rxn #41	AnalytiCon Plate #10	B11	50	0.370	0.0787	0.601	44.2
Rxn #41	AnalytiCon Plate #10	C11	50	0.608	0.0787	0.601	-1.3
Rxn #41	AnalytiCon Plate #10	D11	50	0.588	0.0787	0.601	2.5
Rxn #41	AnalytiCon Plate #10	E11	50	0.595	0.0787	0.601	1.1
Rxn #41	AnalytiCon Plate #10	F11	50	0.583	0.0787	0.601	3.4
Rxn #41	AnalytiCon Plate #10	G11	50	0.533	0.0787	0.601	13.0
Rxn #41	AnalytiCon Plate #10	H11: FOC	-	0.629	0.0787	0.601	-5.4
Rxn #41	AnalytiCon Plate #10	A12: NFC	-	0.089	0.0787	0.601	98.0
Rxn #41	AnalytiCon Plate #10	H12: FOC	-	0.656	0.0787	0.601	-10.5
Rxn #46	AnalytiCon Plate #11	A1: NFC	-	0.064	0.0638	0.625	100.0
Rxn #46	AnalytiCon Plate #11	B1	50	0.590	0.0638	0.625	6.2
Rxn #46	AnalytiCon Plate #11	C1	50	0.501	0.0638	0.625	22.1
Rxn #46	AnalytiCon Plate #11	D1	50	0.583	0.0638	0.625	7.5
Rxn #46	AnalytiCon Plate #11	E1	50	0.568	0.0638	0.625	10.2
Rxn #46	AnalytiCon Plate #11	F1	50	0.579	0.0638	0.625	8.2
Rxn #46	AnalytiCon Plate #11	G1	50	0.561	0.0638	0.625	11.4
Rxn #46	AnalytiCon Plate #11	H1: FOC	-	0.589	0.0638	0.625	6.4
Rxn #46	AnalytiCon Plate #11	A2: NFC	-	0.067	0.0638	0.625	99.4
Rxn #46	AnalytiCon Plate #11	B2	50	0.568	0.0638	0.625	10.2
Rxn #46	AnalytiCon Plate #11	C2	50	0.641	0.0638	0.625	-2.9
Rxn #46	AnalytiCon Plate #11	D2	50	0.619	0.0638	0.625	1.1
Rxn #46	AnalytiCon Plate #11	E2	50	0.632	0.0638	0.625	-1.2
Rxn #46	AnalytiCon Plate #11	F2	50	0.545	0.0638	0.625	14.3
Rxn #46	AnalytiCon Plate #11	G2	50	0.593	0.0638	0.625	5.7
Rxn #46	AnalytiCon Plate #11	H2: FOC	-	0.620	0.0638	0.625	0.9
Rxn #46	AnalytiCon Plate #11	A3: NFC	-	0.070	0.0638	0.625	98.9
Rxn #46	AnalytiCon Plate #11	B3	50	0.614	0.0638	0.625	2.0
Rxn #46	AnalytiCon Plate #11	C3	50	0.612	0.0638	0.625	2.3
Rxn #46	AnalytiCon Plate #11	D3	50	0.638	0.0638	0.625	-2.3
Rxn #46	AnalytiCon Plate #11	E3	50	0.638	0.0638	0.625	-2.3
Rxn #46	AnalytiCon Plate #11	F3	50	0.602	0.0638	0.625	4.1
Rxn #46	AnalytiCon Plate #11	G3	50	0.605	0.0638	0.625	3.6
Rxn #46	AnalytiCon Plate #11	H3: FOC	-	0.617	0.0638	0.625	1.4
Rxn #46	AnalytiCon Plate #11	A4: NFC	-	0.056	0.0638	0.625	101.4
Rxn #46	AnalytiCon Plate #11	B4	50	0.655	0.0638	0.625	-5.3
Rxn #46	AnalytiCon Plate #11	C4	50	0.610	0.0638	0.625	2.7
Rxn #46	AnalytiCon Plate #11	D4	50	0.637	0.0638	0.625	-2.1
Rxn #46	AnalytiCon Plate #11	E4	50	0.483	0.0638	0.625	25.3
Rxn #46	AnalytiCon Plate #11	F4	50	0.472	0.0638	0.625	27.3
Rxn #46	AnalytiCon Plate #11	G4	50	0.543	0.0638	0.625	14.6
Rxn #46	AnalytiCon Plate #11	H4: FOC	-	0.623	0.0638	0.625	0.4
Rxn #46	AnalytiCon Plate #11	A5: NFC	-	0.075	0.0638	0.625	98.0
Rxn #46	AnalytiCon Plate #11	B5: NFC	-	0.065	0.0638	0.625	99.8
Rxn #46	AnalytiCon Plate #11	C5: NFC	-	0.079	0.0638	0.625	97.3
Rxn #46	AnalytiCon Plate #11	D5: NFC	-	0.062	0.0638	0.625	100.3
Rxn #46	AnalytiCon Plate #11	E5: FOC	-	0.633	0.0638	0.625	-1.4
Rxn #46	AnalytiCon Plate #11	F5: FOC	-	0.627	0.0638	0.625	-0.4
Rxn #46	AnalytiCon Plate #11	G5: FOC	-	0.626	0.0638	0.625	-0.2
Rxn #46	AnalytiCon Plate #11	H5: FOC	-	0.612	0.0638	0.625	2.3
Rxn #46	AnalytiCon Plate #11	A6: NFC	-	0.055	0.0638	0.625	101.6
Rxn #46	AnalytiCon Plate #11	B6	50	0.620	0.0638	0.625	0.9
Rxn #46	AnalytiCon Plate #11	C6	50	0.627	0.0638	0.625	-0.4
Rxn #46	AnalytiCon Plate #11	D6	50	0.652	0.0638	0.625	-4.8
Rxn #46	AnalytiCon Plate #11	E6	50	0.652	0.0638	0.625	-4.8
Rxn #46	AnalytiCon Plate #11	F6	50	0.614	0.0638	0.625	2.0
Rxn #46	AnalytiCon Plate #11	G6	50	0.599	0.0638	0.625	4.6
Rxn #46	AnalytiCon Plate #11	H6: FOC	-	0.647	0.0638	0.625	-3.9
Rxn #46	AnalytiCon Plate #11	A7: NFC	-	0.065	0.0638	0.625	99.8
Rxn #46	AnalytiCon Plate #11	B7	50	0.615	0.0638	0.625	1.8
Rxn #46	AnalytiCon Plate #11	C7	50	0.666	0.0638	0.625	-7.3
Rxn #46	AnalytiCon Plate #11	D7	50	0.607	0.0638	0.625	3.2
Rxn #46	AnalytiCon Plate #11	E7	50	0.631	0.0638	0.625	-1.1
Rxn #46	AnalytiCon Plate #11	F7	50	0.602	0.0638	0.625	4.1
Rxn #46	AnalytiCon Plate #11	G7	50	0.626	0.0638	0.625	-0.2
Rxn #46	AnalytiCon Plate #11	H7: FOC	-	0.658	0.0638	0.625	-5.9
Rxn #46	AnalytiCon Plate #11	A8: NFC	-	0.060	0.0638	0.625	100.7
Rxn #46	AnalytiCon Plate #11	B8	50	0.466	0.0638	0.625	28.3
Rxn #46	AnalytiCon Plate #11	C8	50	0.576	0.0638	0.625	8.7
Rxn #46	AnalytiCon Plate #11	D8	50	0.642	0.0638	0.625	-3.0
Rxn #46	AnalytiCon Plate #11	E8	50	0.613	0.0638	0.625	2.1
Rxn #46	AnalytiCon Plate #11	F8	50	0.499	0.0638	0.625	22.5

S3 Table. Data from Screening of Phytochemical Library for Inhibition of KHKC Activity.

Rxn ID	Sample			Sample	Ave. No Fructose Controls	Ave. Fructose Only Controls	KHKC
	Phytochemical Plate	Well	Conc (μM)	(ΔOD$_{340nm}$)	(ΔOD$_{340nm}$)	(ΔOD$_{340nm}$)	Inhibition (%)
Rxn #46	AnalytiCon Plate #11	G8	50	0.596	0.0638	0.625	5.2
Rxn #46	AnalytiCon Plate #11	H8: FOC	-	0.658	0.0638	0.625	-5.9
Rxn #46	AnalytiCon Plate #11	A9: NFC	-	0.068	0.0638	0.625	99.3
Rxn #46	AnalytiCon Plate #11	B9	50	0.629	0.0638	0.625	-0.7
Rxn #46	AnalytiCon Plate #11	C9	50	0.614	0.0638	0.625	2.0
Rxn #46	AnalytiCon Plate #11	D9	50	0.515	0.0638	0.625	19.6
Rxn #46	AnalytiCon Plate #11	E9	50	0.609	0.0638	0.625	2.9
Rxn #46	AnalytiCon Plate #11	F9	50	0.508	0.0638	0.625	20.8
Rxn #46	AnalytiCon Plate #11	G9	50	0.520	0.0638	0.625	18.7
Rxn #46	AnalytiCon Plate #11	H9: FOC	-	0.634	0.0638	0.625	-1.6
Rxn #46	AnalytiCon Plate #11	A10: NFC	-	0.052	0.0638	0.625	102.1
Rxn #46	AnalytiCon Plate #11	B10	50	0.604	0.0638	0.625	3.7
Rxn #46	AnalytiCon Plate #11	C10	50	0.594	0.0638	0.625	5.5
Rxn #46	AnalytiCon Plate #11	D10	50	0.622	0.0638	0.625	0.5
Rxn #46	AnalytiCon Plate #11	E10	50	0.614	0.0638	0.625	2.0
Rxn #46	AnalytiCon Plate #11	F10	50	0.611	0.0638	0.625	2.5
Rxn #46	AnalytiCon Plate #11	G10	50	0.602	0.0638	0.625	4.1
Rxn #46	AnalytiCon Plate #11	H10: FOC	-	0.624	0.0638	0.625	0.2
Rxn #46	AnalytiCon Plate #11	A11: NFC	-	0.072	0.0638	0.625	98.5
Rxn #46	AnalytiCon Plate #11	B11	50	0.622	0.0638	0.625	0.5
Rxn #46	AnalytiCon Plate #11	C11	50	0.582	0.0638	0.625	7.7
Rxn #46	AnalytiCon Plate #11	D11	50	0.581	0.0638	0.625	7.8
Rxn #46	AnalytiCon Plate #11	E11	50	0.580	0.0638	0.625	8.0
Rxn #46	AnalytiCon Plate #11	F11	50	0.529	0.0638	0.625	17.1
Rxn #46	AnalytiCon Plate #11	G11	50	0.581	0.0638	0.625	7.8
Rxn #46	AnalytiCon Plate #11	H11: FOC	-	0.637	0.0638	0.625	-2.1
Rxn #46	AnalytiCon Plate #11	A12: NFC	-	0.047	0.0638	0.625	103.0
Rxn #46	AnalytiCon Plate #11	H12: FOC	-	0.577	0.0638	0.625	8.6
Rxn #47	AnalytiCon Plate #12	A1: NFC	-	0.070	0.0642	0.645	99.0
Rxn #47	AnalytiCon Plate #12	B1	50	0.620	0.0642	0.645	4.3
Rxn #47	AnalytiCon Plate #12	C1	50	0.604	0.0642	0.645	7.1
Rxn #47	AnalytiCon Plate #12	D1	50	0.607	0.0642	0.645	6.5
Rxn #47	AnalytiCon Plate #12	E1	50	0.593	0.0642	0.645	9.0
Rxn #47	AnalytiCon Plate #12	F1	50	0.568	0.0642	0.645	13.3
Rxn #47	AnalytiCon Plate #12	G1	50	0.587	0.0642	0.645	10.0
Rxn #47	AnalytiCon Plate #12	H1: FOC	-	0.581	0.0642	0.645	11.0
Rxn #47	AnalytiCon Plate #12	A2: NFC	-	0.081	0.0642	0.645	97.1
Rxn #47	AnalytiCon Plate #12	B2	50	0.630	0.0642	0.645	2.6
Rxn #47	AnalytiCon Plate #12	C2	50	0.615	0.0642	0.645	5.2
Rxn #47	AnalytiCon Plate #12	D2	50	0.653	0.0642	0.645	-1.4
Rxn #47	AnalytiCon Plate #12	E2	50	0.636	0.0642	0.645	1.5
Rxn #47	AnalytiCon Plate #12	F2	50	0.522	0.0642	0.645	21.2
Rxn #47	AnalytiCon Plate #12	G2	50	0.637	0.0642	0.645	1.4
Rxn #47	AnalytiCon Plate #12	H2: FOC	-	0.632	0.0642	0.645	2.2
Rxn #47	AnalytiCon Plate #12	A3: NFC	-	0.079	0.0642	0.645	97.5
Rxn #47	AnalytiCon Plate #12	B3	50	0.651	0.0642	0.645	-1.0
Rxn #47	AnalytiCon Plate #12	C3	50	0.629	0.0642	0.645	2.8
Rxn #47	AnalytiCon Plate #12	D3	50	0.566	0.0642	0.645	13.6
Rxn #47	AnalytiCon Plate #12	E3	50	0.639	0.0642	0.645	1.0
Rxn #47	AnalytiCon Plate #12	F3	50	0.637	0.0642	0.645	1.4
Rxn #47	AnalytiCon Plate #12	G3	50	0.627	0.0642	0.645	3.1
Rxn #47	AnalytiCon Plate #12	H3: FOC	-	0.660	0.0642	0.645	-2.6
Rxn #47	AnalytiCon Plate #12	A4: NFC	-	0.060	0.0642	0.645	100.7
Rxn #47	AnalytiCon Plate #12	B4	50	0.631	0.0642	0.645	2.4
Rxn #47	AnalytiCon Plate #12	C4	50	0.653	0.0642	0.645	-1.4
Rxn #47	AnalytiCon Plate #12	D4	50	0.626	0.0642	0.645	3.3
Rxn #47	AnalytiCon Plate #12	E4	50	0.626	0.0642	0.645	3.3
Rxn #47	AnalytiCon Plate #12	F4	50	0.645	0.0642	0.645	0.0
Rxn #47	AnalytiCon Plate #12	G4	50	0.659	0.0642	0.645	-2.4
Rxn #47	AnalytiCon Plate #12	H4: FOC	-	0.668	0.0642	0.645	-4.0
Rxn #47	AnalytiCon Plate #12	A5: NFC	-	0.080	0.0642	0.645	97.3
Rxn #47	AnalytiCon Plate #12	B5: NFC	-	0.061	0.0642	0.645	100.6
Rxn #47	AnalytiCon Plate #12	C5: NFC	-	0.059	0.0642	0.645	100.9
Rxn #47	AnalytiCon Plate #12	D5: NFC	-	0.065	0.0642	0.645	99.9
Rxn #47	AnalytiCon Plate #12	E5: FOC	-	0.655	0.0642	0.645	-1.7
Rxn #47	AnalytiCon Plate #12	F5: FOC	-	0.648	0.0642	0.645	-0.5
Rxn #47	AnalytiCon Plate #12	G5: FOC	-	0.665	0.0642	0.645	-3.4
Rxn #47	AnalytiCon Plate #12	H5: FOC	-	0.662	0.0642	0.645	-2.9
Rxn #47	AnalytiCon Plate #12	A6: NFC	-	0.075	0.0642	0.645	98.1
Rxn #47	AnalytiCon Plate #12	B6	50	0.654	0.0642	0.645	-1.5
Rxn #47	AnalytiCon Plate #12	C6	50	0.655	0.0642	0.645	-1.7
Rxn #47	AnalytiCon Plate #12	D6	50	0.616	0.0642	0.645	5.0
Rxn #47	AnalytiCon Plate #12	E6	50	0.557	0.0642	0.645	15.2
Rxn #47	AnalytiCon Plate #12	F6	50	0.631	0.0642	0.645	2.4

S3 Table. Data from Screening of Phytochemical Library for Inhibition of KHKC Activity.

Rxn ID	Sample			Sample	Ave. No Fructose Controls	Ave. Fructose Only Controls	KHKC
	Phytochemical Plate	Well	Conc (μM)	(ΔOD_{340nm})	(ΔOD_{340nm})	(ΔOD_{340nm})	Inhibition (%)
Rxn #47	AnalytiCon Plate #12	G6	50	0.657	0.0642	0.645	-2.1
Rxn #47	AnalytiCon Plate #12	H6: FOC	-	0.630	0.0642	0.645	2.6
Rxn #47	AnalytiCon Plate #12	A7: NFC	-	0.062	0.0642	0.645	100.4
Rxn #47	AnalytiCon Plate #12	B7	50	0.617	0.0642	0.645	4.8
Rxn #47	AnalytiCon Plate #12	C7	50	0.603	0.0642	0.645	7.2
Rxn #47	AnalytiCon Plate #12	D7	50	0.630	0.0642	0.645	2.6
Rxn #47	AnalytiCon Plate #12	E7	50	0.660	0.0642	0.645	-2.6
Rxn #47	AnalytiCon Plate #12	F7	50	0.584	0.0642	0.645	10.5
Rxn #47	AnalytiCon Plate #12	G7	50	0.571	0.0642	0.645	12.7
Rxn #47	AnalytiCon Plate #12	H7: FOC	-	0.648	0.0642	0.645	-0.5
Rxn #47	AnalytiCon Plate #12	A8: NFC	-	0.064	0.0642	0.645	100.0
Rxn #47	AnalytiCon Plate #12	B8	50	0.637	0.0642	0.645	1.4
Rxn #47	AnalytiCon Plate #12	C8	50	0.579	0.0642	0.645	11.4
Rxn #47	AnalytiCon Plate #12	D8	50	0.568	0.0642	0.645	13.3
Rxn #47	AnalytiCon Plate #12	E8	50	0.583	0.0642	0.645	10.7
Rxn #47	AnalytiCon Plate #12	F8	50	0.558	0.0642	0.645	15.0
Rxn #47	AnalytiCon Plate #12	G8	50	0.620	0.0642	0.645	4.3
Rxn #47	AnalytiCon Plate #12	H8: FOC	-	0.654	0.0642	0.645	-1.5
Rxn #47	AnalytiCon Plate #12	A9: NFC	-	0.049	0.0642	0.645	102.6
Rxn #47	AnalytiCon Plate #12	B9	50	0.612	0.0642	0.645	5.7
Rxn #47	AnalytiCon Plate #12	C9	50	0.622	0.0642	0.645	4.0
Rxn #47	AnalytiCon Plate #12	D9	50	0.610	0.0642	0.645	6.0
Rxn #47	AnalytiCon Plate #12	E9	50	0.612	0.0642	0.645	5.7
Rxn #47	AnalytiCon Plate #12	F9	50	0.616	0.0642	0.645	5.0
Rxn #47	AnalytiCon Plate #12	G9	50	0.622	0.0642	0.645	4.0
Rxn #47	AnalytiCon Plate #12	H9: FOC	-	0.652	0.0642	0.645	-1.2
Rxn #47	AnalytiCon Plate #12	A10: NFC	-	0.045	0.0642	0.645	103.3
Rxn #47	AnalytiCon Plate #12	B10	50	0.615	0.0642	0.645	5.2
Rxn #47	AnalytiCon Plate #12	C10	50	0.616	0.0642	0.645	5.0
Rxn #47	AnalytiCon Plate #12	D10	50	0.617	0.0642	0.645	4.8
Rxn #47	AnalytiCon Plate #12	E10	50	0.616	0.0642	0.645	5.0
Rxn #47	AnalytiCon Plate #12	F10	50	0.609	0.0642	0.645	6.2
Rxn #47	AnalytiCon Plate #12	G10	50	0.570	0.0642	0.645	12.9
Rxn #47	AnalytiCon Plate #12	H10: FOC	-	0.635	0.0642	0.645	1.7
Rxn #47	AnalytiCon Plate #12	A11: NFC	-	0.053	0.0642	0.645	101.9
Rxn #47	AnalytiCon Plate #12	B11	50	0.586	0.0642	0.645	10.2
Rxn #47	AnalytiCon Plate #12	C11	50	0.609	0.0642	0.645	6.2
Rxn #47	AnalytiCon Plate #12	D11	50	0.596	0.0642	0.645	8.4
Rxn #47	AnalytiCon Plate #12	E11	50	0.613	0.0642	0.645	5.5
Rxn #47	AnalytiCon Plate #12	F11	50	0.596	0.0642	0.645	8.4
Rxn #47	AnalytiCon Plate #12	G11	50	0.596	0.0642	0.645	8.4
Rxn #47	AnalytiCon Plate #12	H11: FOC	-	0.646	0.0642	0.645	-0.2
Rxn #47	AnalytiCon Plate #12	A12: NFC	-	0.060	0.0642	0.645	100.7
Rxn #47	AnalytiCon Plate #12	H12: FOC	-	0.598	0.0642	0.645	8.1
Rxn #48	AnalytiCon Plate #13	A1: NFC	-	0.043	0.0515	0.560	101.7
Rxn #48	AnalytiCon Plate #13	B1	50	0.540	0.0515	0.560	3.9
Rxn #48	AnalytiCon Plate #13	C1	50	0.547	0.0515	0.560	2.6
Rxn #48	AnalytiCon Plate #13	D1	50	0.540	0.0515	0.560	3.9
Rxn #48	AnalytiCon Plate #13	E1	50	0.544	0.0515	0.560	3.1
Rxn #48	AnalytiCon Plate #13	F1	50	0.540	0.0515	0.560	3.9
Rxn #48	AnalytiCon Plate #13	G1	50	0.529	0.0515	0.560	6.1
Rxn #48	AnalytiCon Plate #13	H1: FOC	-	0.537	0.0515	0.560	4.5
Rxn #48	AnalytiCon Plate #13	A2: NFC	-	0.059	0.0515	0.560	98.5
Rxn #48	AnalytiCon Plate #13	B2	50	0.523	0.0515	0.560	7.3
Rxn #48	AnalytiCon Plate #13	C2	50	0.532	0.0515	0.560	5.5
Rxn #48	AnalytiCon Plate #13	D2	50	0.547	0.0515	0.560	2.6
Rxn #48	AnalytiCon Plate #13	E2	50	0.543	0.0515	0.560	3.3
Rxn #48	AnalytiCon Plate #13	F2	50	0.554	0.0515	0.560	1.2
Rxn #48	AnalytiCon Plate #13	G2	50	0.492	0.0515	0.560	13.4
Rxn #48	AnalytiCon Plate #13	H2: FOC	-	0.560	0.0515	0.560	0.0
Rxn #48	AnalytiCon Plate #13	A3: NFC	-	0.059	0.0515	0.560	98.5
Rxn #48	AnalytiCon Plate #13	B3	50	0.498	0.0515	0.560	12.2
Rxn #48	AnalytiCon Plate #13	C3	50	0.540	0.0515	0.560	3.9
Rxn #48	AnalytiCon Plate #13	D3	50	0.543	0.0515	0.560	3.3
Rxn #48	AnalytiCon Plate #13	E3	50	0.461	0.0515	0.560	19.5
Rxn #48	AnalytiCon Plate #13	F3	50	0.544	0.0515	0.560	3.1
Rxn #48	AnalytiCon Plate #13	G3	50	0.545	0.0515	0.560	2.9
Rxn #48	AnalytiCon Plate #13	H3: FOC	-	0.572	0.0515	0.560	-2.4
Rxn #48	AnalytiCon Plate #13	A4: NFC	-	0.056	0.0515	0.560	99.1
Rxn #48	AnalytiCon Plate #13	B4	50	0.558	0.0515	0.560	0.4
Rxn #48	AnalytiCon Plate #13	C4	50	0.543	0.0515	0.560	3.3
Rxn #48	AnalytiCon Plate #13	D4	50	0.548	0.0515	0.560	2.4
Rxn #48	AnalytiCon Plate #13	E4	50	0.538	0.0515	0.560	4.3
Rxn #48	AnalytiCon Plate #13	F4	50	0.559	0.0515	0.560	0.2

S3 Table. Data from Screening of Phytochemical Library for Inhibition of KHKC Activity.

Rxn ID	Sample			Sample (ΔOD_{340nm})	Ave. No Fructose Controls (ΔOD_{340nm})	Ave. Fructose Only Controls (ΔOD_{340nm})	KHKC Inhibition (%)
	Phytochemical Plate	Well	Conc (μM)				
Rxn #48	AnalytiCon Plate #13	G4	50	0.496	0.0515	0.560	12.6
Rxn #48	AnalytiCon Plate #13	H4: FOC	-	0.561	0.0515	0.560	-0.2
Rxn #48	AnalytiCon Plate #13	A5: NFC	-	0.050	0.0515	0.560	100.3
Rxn #48	AnalytiCon Plate #13	B5: NFC	-	0.052	0.0515	0.560	99.9
Rxn #48	AnalytiCon Plate #13	C5: NFC	-	0.050	0.0515	0.560	100.3
Rxn #48	AnalytiCon Plate #13	D5: NFC	-	0.058	0.0515	0.560	98.7
Rxn #48	AnalytiCon Plate #13	E5: FOC	-	0.566	0.0515	0.560	-1.2
Rxn #48	AnalytiCon Plate #13	F5: FOC	-	0.559	0.0515	0.560	0.2
Rxn #48	AnalytiCon Plate #13	G5: FOC	-	0.561	0.0515	0.560	-0.2
Rxn #48	AnalytiCon Plate #13	H5: FOC	-	0.564	0.0515	0.560	-0.8
Rxn #48	AnalytiCon Plate #13	A6: NFC	-	0.049	0.0515	0.560	100.5
Rxn #48	AnalytiCon Plate #13	B6	50	0.534	0.0515	0.560	5.1
Rxn #48	AnalytiCon Plate #13	C6	50	0.496	0.0515	0.560	12.6
Rxn #48	AnalytiCon Plate #13	D6	50	0.564	0.0515	0.560	-0.8
Rxn #48	AnalytiCon Plate #13	E6	50	0.559	0.0515	0.560	0.2
Rxn #48	AnalytiCon Plate #13	F6	50	0.562	0.0515	0.560	-0.4
Rxn #48	AnalytiCon Plate #13	G6	50	0.242	0.0515	0.560	62.5
Rxn #48	AnalytiCon Plate #13	H6: FOC	-	0.571	0.0515	0.560	-2.2
Rxn #48	AnalytiCon Plate #13	A7: NFC	-	0.051	0.0515	0.560	100.1
Rxn #48	AnalytiCon Plate #13	B7	50	0.568	0.0515	0.560	-1.6
Rxn #48	AnalytiCon Plate #13	C7	50	0.559	0.0515	0.560	0.2
Rxn #48	AnalytiCon Plate #13	D7	50	0.564	0.0515	0.560	-0.8
Rxn #48	AnalytiCon Plate #13	E7	50	0.565	0.0515	0.560	-1.0
Rxn #48	AnalytiCon Plate #13	F7	50	0.545	0.0515	0.560	2.9
Rxn #48	AnalytiCon Plate #13	G7	50	0.551	0.0515	0.560	1.8
Rxn #48	AnalytiCon Plate #13	H7: FOC	-	0.548	0.0515	0.560	2.4
Rxn #48	AnalytiCon Plate #13	A8: NFC	-	0.053	0.0515	0.560	99.7
Rxn #48	AnalytiCon Plate #13	B8	50	0.554	0.0515	0.560	1.2
Rxn #48	AnalytiCon Plate #13	C8	50	0.588	0.0515	0.560	-5.5
Rxn #48	AnalytiCon Plate #13	D8	50	0.521	0.0515	0.560	7.7
Rxn #48	AnalytiCon Plate #13	E8	50	0.552	0.0515	0.560	1.6
Rxn #48	AnalytiCon Plate #13	F8	50	0.522	0.0515	0.560	7.5
Rxn #48	AnalytiCon Plate #13	G8	50	0.541	0.0515	0.560	3.7
Rxn #48	AnalytiCon Plate #13	H8: FOC	-	0.560	0.0515	0.560	0.0
Rxn #48	AnalytiCon Plate #13	A9: NFC	-	0.047	0.0515	0.560	100.9
Rxn #48	AnalytiCon Plate #13	B9	50	0.554	0.0515	0.560	1.2
Rxn #48	AnalytiCon Plate #13	C9	50	0.567	0.0515	0.560	-1.4
Rxn #48	AnalytiCon Plate #13	D9	50	0.566	0.0515	0.560	-1.2
Rxn #48	AnalytiCon Plate #13	E9	50	0.557	0.0515	0.560	0.6
Rxn #48	AnalytiCon Plate #13	F9	50	0.565	0.0515	0.560	-1.0
Rxn #48	AnalytiCon Plate #13	G9	50	0.588	0.0515	0.560	-5.5
Rxn #48	AnalytiCon Plate #13	H9: FOC	-	0.569	0.0515	0.560	-1.8
Rxn #48	AnalytiCon Plate #13	A10: NFC	-	0.048	0.0515	0.560	100.7
Rxn #48	AnalytiCon Plate #13	B10	50	0.534	0.0515	0.560	5.1
Rxn #48	AnalytiCon Plate #13	C10	50	0.560	0.0515	0.560	0.0
Rxn #48	AnalytiCon Plate #13	D10	50	0.568	0.0515	0.560	-1.6
Rxn #48	AnalytiCon Plate #13	E10	50	0.529	0.0515	0.560	6.1
Rxn #48	AnalytiCon Plate #13	F10	50	0.557	0.0515	0.560	0.6
Rxn #48	AnalytiCon Plate #13	G10	50	0.559	0.0515	0.560	0.2
Rxn #48	AnalytiCon Plate #13	H10: FOC	-	0.574	0.0515	0.560	-2.8
Rxn #48	AnalytiCon Plate #13	A11: NFC	-	0.047	0.0515	0.560	100.9
Rxn #48	AnalytiCon Plate #13	B11	50	0.567	0.0515	0.560	-1.4
Rxn #48	AnalytiCon Plate #13	C11	50	0.556	0.0515	0.560	0.8
Rxn #48	AnalytiCon Plate #13	D11	50	0.575	0.0515	0.560	-2.9
Rxn #48	AnalytiCon Plate #13	E11	50	0.587	0.0515	0.560	-5.3
Rxn #48	AnalytiCon Plate #13	F11	50	0.591	0.0515	0.560	-6.1
Rxn #48	AnalytiCon Plate #13	G11	50	0.569	0.0515	0.560	-1.8
Rxn #48	AnalytiCon Plate #13	H11: FOC	-	0.594	0.0515	0.560	-6.7
Rxn #48	AnalytiCon Plate #13	A12: NFC	-	0.051	0.0515	0.560	100.1
Rxn #48	AnalytiCon Plate #13	H12: FOC	-	0.614	0.0515	0.560	-10.6
Rxn #49	AnalytiCon Plate #14	A1: NFC	-	0.046	0.0498	0.568	100.7
Rxn #49	AnalytiCon Plate #14	B1	50	0.524	0.0498	0.568	8.5
Rxn #49	AnalytiCon Plate #14	C1	50	0.488	0.0498	0.568	15.4
Rxn #49	AnalytiCon Plate #14	D1	50	0.495	0.0498	0.568	14.1
Rxn #49	AnalytiCon Plate #14	E1	50	0.552	0.0498	0.568	3.1
Rxn #49	AnalytiCon Plate #14	F1	50	0.555	0.0498	0.568	2.5
Rxn #49	AnalytiCon Plate #14	G1	50	0.475	0.0498	0.568	17.9
Rxn #49	AnalytiCon Plate #14	H1: FOC	-	0.518	0.0498	0.568	9.6
Rxn #49	AnalytiCon Plate #14	A2: NFC	-	0.044	0.0498	0.568	101.1
Rxn #49	AnalytiCon Plate #14	B2	50	0.516	0.0498	0.568	10.0
Rxn #49	AnalytiCon Plate #14	C2	50	0.532	0.0498	0.568	6.9
Rxn #49	AnalytiCon Plate #14	D2	50	0.548	0.0498	0.568	3.9
Rxn #49	AnalytiCon Plate #14	E2	50	0.572	0.0498	0.568	-0.8
Rxn #49	AnalytiCon Plate #14	F2	50	0.530	0.0498	0.568	7.3

S3 Table. Data from Screening of Phytochemical Library for Inhibition of KHKC Activity.

Rxn ID	Sample			Sample	Ave. No Fructose Controls	Ave. Fructose Only Controls	KHKC
	Phytochemical Plate	Well	Conc (μM)	(ΔOD_{340nm})	(ΔOD_{340nm})	(ΔOD_{340nm})	Inhibition (%)
Rxn #49	AnalytiCon Plate #14	G2	50	0.555	0.0498	0.568	2.5
Rxn #49	AnalytiCon Plate #14	H2: FOC	-	0.562	0.0498	0.568	1.2
Rxn #49	AnalytiCon Plate #14	A3: NFC	-	0.050	0.0498	0.568	100.0
Rxn #49	AnalytiCon Plate #14	B3	50	0.544	0.0498	0.568	4.6
Rxn #49	AnalytiCon Plate #14	C3	50	0.498	0.0498	0.568	13.5
Rxn #49	AnalytiCon Plate #14	D3	50	0.567	0.0498	0.568	0.2
Rxn #49	AnalytiCon Plate #14	E3	50	0.478	0.0498	0.568	17.4
Rxn #49	AnalytiCon Plate #14	F3	50	0.566	0.0498	0.568	0.4
Rxn #49	AnalytiCon Plate #14	G3	50	0.553	0.0498	0.568	2.9
Rxn #49	AnalytiCon Plate #14	H3: FOC	-	0.551	0.0498	0.568	3.3
Rxn #49	AnalytiCon Plate #14	A4: NFC	-	0.039	0.0498	0.568	102.1
Rxn #49	AnalytiCon Plate #14	B4	50	0.484	0.0498	0.568	16.2
Rxn #49	AnalytiCon Plate #14	C4	50	0.533	0.0498	0.568	6.8
Rxn #49	AnalytiCon Plate #14	D4	50	0.571	0.0498	0.568	-0.6
Rxn #49	AnalytiCon Plate #14	E4	50	0.565	0.0498	0.568	0.6
Rxn #49	AnalytiCon Plate #14	F4	50	0.505	0.0498	0.568	12.2
Rxn #49	AnalytiCon Plate #14	G4	50	0.555	0.0498	0.568	2.5
Rxn #49	AnalytiCon Plate #14	H4: FOC	-	0.591	0.0498	0.568	-4.4
Rxn #49	AnalytiCon Plate #14	A5: NFC	-	0.046	0.0498	0.568	100.7
Rxn #49	AnalytiCon Plate #14	B5: NFC	-	0.050	0.0498	0.568	100.0
Rxn #49	AnalytiCon Plate #14	C5: NFC	-	0.050	0.0498	0.568	100.0
Rxn #49	AnalytiCon Plate #14	D5: NFC	-	0.052	0.0498	0.568	99.6
Rxn #49	AnalytiCon Plate #14	E5: FOC	-	0.576	0.0498	0.568	-1.5
Rxn #49	AnalytiCon Plate #14	F5: FOC	-	0.571	0.0498	0.568	-0.6
Rxn #49	AnalytiCon Plate #14	G5: FOC	-	0.581	0.0498	0.568	-2.5
Rxn #49	AnalytiCon Plate #14	H5: FOC	-	0.585	0.0498	0.568	-3.3
Rxn #49	AnalytiCon Plate #14	A6: NFC	-	0.042	0.0498	0.568	101.5
Rxn #49	AnalytiCon Plate #14	B6	50	0.490	0.0498	0.568	15.1
Rxn #49	AnalytiCon Plate #14	C6	50	0.378	0.0498	0.568	36.7
Rxn #49	AnalytiCon Plate #14	D6	50	0.572	0.0498	0.568	-0.8
Rxn #49	AnalytiCon Plate #14	E6	50	0.504	0.0498	0.568	12.4
Rxn #49	AnalytiCon Plate #14	F6	50	0.576	0.0498	0.568	-1.5
Rxn #49	AnalytiCon Plate #14	G6	50	0.555	0.0498	0.568	2.5
Rxn #49	AnalytiCon Plate #14	H6: FOC	-	0.560	0.0498	0.568	1.5
Rxn #49	AnalytiCon Plate #14	A7: NFC	-	0.060	0.0498	0.568	98.0
Rxn #49	AnalytiCon Plate #14	B7	50	0.555	0.0498	0.568	2.5
Rxn #49	AnalytiCon Plate #14	C7	50	0.481	0.0498	0.568	16.8
Rxn #49	AnalytiCon Plate #14	D7	50	0.550	0.0498	0.568	3.5
Rxn #49	AnalytiCon Plate #14	E7	50	0.545	0.0498	0.568	4.4
Rxn #49	AnalytiCon Plate #14	F7	50	0.559	0.0498	0.568	1.7
Rxn #49	AnalytiCon Plate #14	G7	50	0.571	0.0498	0.568	-0.6
Rxn #49	AnalytiCon Plate #14	H7: FOC	-	0.589	0.0498	0.568	-4.1
Rxn #49	AnalytiCon Plate #14	A8: NFC	-	0.050	0.0498	0.568	100.0
Rxn #49	AnalytiCon Plate #14	B8	50	0.434	0.0498	0.568	25.9
Rxn #49	AnalytiCon Plate #14	C8	50	0.459	0.0498	0.568	21.0
Rxn #49	AnalytiCon Plate #14	D8	50	0.547	0.0498	0.568	4.1
Rxn #49	AnalytiCon Plate #14	E8	50	0.549	0.0498	0.568	3.7
Rxn #49	AnalytiCon Plate #14	F8	50	0.566	0.0498	0.568	0.4
Rxn #49	AnalytiCon Plate #14	G8	50	0.547	0.0498	0.568	4.1
Rxn #49	AnalytiCon Plate #14	H8: FOC	-	0.553	0.0498	0.568	2.9
Rxn #49	AnalytiCon Plate #14	A9: NFC	-	0.054	0.0498	0.568	99.2
Rxn #49	AnalytiCon Plate #14	B9	50	0.538	0.0498	0.568	5.8
Rxn #49	AnalytiCon Plate #14	C9	50	0.550	0.0498	0.568	3.5
Rxn #49	AnalytiCon Plate #14	D9	50	0.540	0.0498	0.568	5.4
Rxn #49	AnalytiCon Plate #14	E9	50	0.532	0.0498	0.568	6.9
Rxn #49	AnalytiCon Plate #14	F9	50	0.562	0.0498	0.568	1.2
Rxn #49	AnalytiCon Plate #14	G9	50	0.514	0.0498	0.568	10.4
Rxn #49	AnalytiCon Plate #14	H9: FOC	-	0.541	0.0498	0.568	5.2
Rxn #49	AnalytiCon Plate #14	A10: NFC	-	0.056	0.0498	0.568	98.8
Rxn #49	AnalytiCon Plate #14	B10	50	0.551	0.0498	0.568	3.3
Rxn #49	AnalytiCon Plate #14	C10	50	0.510	0.0498	0.568	11.2
Rxn #49	AnalytiCon Plate #14	D10	50	0.551	0.0498	0.568	3.3
Rxn #49	AnalytiCon Plate #14	E10	50	0.451	0.0498	0.568	22.6
Rxn #49	AnalytiCon Plate #14	F10	50	0.551	0.0498	0.568	3.3
Rxn #49	AnalytiCon Plate #14	G10	50	0.556	0.0498	0.568	2.3
Rxn #49	AnalytiCon Plate #14	H10: FOC	-	0.527	0.0498	0.568	7.9
Rxn #49	AnalytiCon Plate #14	A11: NFC	-	0.053	0.0498	0.568	99.4
Rxn #49	AnalytiCon Plate #14	B11	50	0.547	0.0498	0.568	4.1
Rxn #49	AnalytiCon Plate #14	C11	50	0.542	0.0498	0.568	5.0
Rxn #49	AnalytiCon Plate #14	D11	50	0.568	0.0498	0.568	0.0
Rxn #49	AnalytiCon Plate #14	E11	50	0.548	0.0498	0.568	3.9
Rxn #49	AnalytiCon Plate #14	F11	50	0.482	0.0498	0.568	16.6
Rxn #49	AnalytiCon Plate #14	G11	50	0.513	0.0498	0.568	10.6
Rxn #49	AnalytiCon Plate #14	H11: FOC	-	0.536	0.0498	0.568	6.2

S3 Table. Data from Screening of Phytochemical Library for Inhibition of KHKC Activity.

Rxn ID	Sample			Sample	Ave. No Fructose Controls	Ave. Fructose Only Controls	KHKC
	Phytochemical Plate	Well	Conc (µM)	(ΔOD_{340nm})	(ΔOD_{340nm})	(ΔOD_{340nm})	Inhibition (%)
Rxn #49	AnalytiCon Plate #14	A12: NFC	-	0.055	0.0498	0.568	99.0
Rxn #49	AnalytiCon Plate #14	H12: FOC	-	0.535	0.0498	0.568	6.4
Rxn #50	AnalytiCon Plate #15	A1: NFC	-	0.079	0.0741	0.628	99.1
Rxn #50	AnalytiCon Plate #15	B1	50	0.540	0.0741	0.628	15.9
Rxn #50	AnalytiCon Plate #15	C1	50	0.571	0.0741	0.628	10.3
Rxn #50	AnalytiCon Plate #15	D1	50	0.543	0.0741	0.628	15.3
Rxn #50	AnalytiCon Plate #15	E1	50	0.589	0.0741	0.628	7.0
Rxn #50	AnalytiCon Plate #15	F1	50	0.581	0.0741	0.628	8.5
Rxn #50	AnalytiCon Plate #15	G1	50	0.595	0.0741	0.628	6.0
Rxn #50	AnalytiCon Plate #15	H1: FOC	-	0.563	0.0741	0.628	11.7
Rxn #50	AnalytiCon Plate #15	A2: NFC	-	0.078	0.0741	0.628	99.3
Rxn #50	AnalytiCon Plate #15	B2	50	0.626	0.0741	0.628	0.4
Rxn #50	AnalytiCon Plate #15	C2	50	0.604	0.0741	0.628	4.3
Rxn #50	AnalytiCon Plate #15	D2	50	0.592	0.0741	0.628	6.5
Rxn #50	AnalytiCon Plate #15	E2	50	0.600	0.0741	0.628	5.1
Rxn #50	AnalytiCon Plate #15	F2	50	0.540	0.0741	0.628	15.9
Rxn #50	AnalytiCon Plate #15	G2	50	0.599	0.0741	0.628	5.2
Rxn #50	AnalytiCon Plate #15	H2: FOC	-	0.620	0.0741	0.628	1.4
Rxn #50	AnalytiCon Plate #15	A3: NFC	-	0.093	0.0741	0.628	96.6
Rxn #50	AnalytiCon Plate #15	B3	50	0.594	0.0741	0.628	6.1
Rxn #50	AnalytiCon Plate #15	C3	50	0.584	0.0741	0.628	7.9
Rxn #50	AnalytiCon Plate #15	D3	50	0.609	0.0741	0.628	3.4
Rxn #50	AnalytiCon Plate #15	E3	50	0.570	0.0741	0.628	10.5
Rxn #50	AnalytiCon Plate #15	F3	50	0.569	0.0741	0.628	10.7
Rxn #50	AnalytiCon Plate #15	G3	50	0.619	0.0741	0.628	1.6
Rxn #50	AnalytiCon Plate #15	H3: FOC	-	0.640	0.0741	0.628	-2.2
Rxn #50	AnalytiCon Plate #15	A4: NFC	-	0.082	0.0741	0.628	98.6
Rxn #50	AnalytiCon Plate #15	B4	50	0.625	0.0741	0.628	0.5
Rxn #50	AnalytiCon Plate #15	C4	50	0.620	0.0741	0.628	1.4
Rxn #50	AnalytiCon Plate #15	D4	50	0.621	0.0741	0.628	1.3
Rxn #50	AnalytiCon Plate #15	E4	50	0.625	0.0741	0.628	0.5
Rxn #50	AnalytiCon Plate #15	F4	50	0.618	0.0741	0.628	1.8
Rxn #50	AnalytiCon Plate #15	G4	50	0.620	0.0741	0.628	1.4
Rxn #50	AnalytiCon Plate #15	H4: FOC	-	0.646	0.0741	0.628	-3.2
Rxn #50	AnalytiCon Plate #15	A5: NFC	-	0.084	0.0741	0.628	98.2
Rxn #50	AnalytiCon Plate #15	B5: NFC	-	0.072	0.0741	0.628	100.4
Rxn #50	AnalytiCon Plate #15	C5: NFC	-	0.072	0.0741	0.628	100.4
Rxn #50	AnalytiCon Plate #15	D5: NFC	-	0.072	0.0741	0.628	100.4
Rxn #50	AnalytiCon Plate #15	E5: FOC	-	0.622	0.0741	0.628	1.1
Rxn #50	AnalytiCon Plate #15	F5: FOC	-	0.626	0.0741	0.628	0.4
Rxn #50	AnalytiCon Plate #15	G5: FOC	-	0.656	0.0741	0.628	-5.1
Rxn #50	AnalytiCon Plate #15	H5: FOC	-	0.638	0.0741	0.628	-1.8
Rxn #50	AnalytiCon Plate #15	A6: NFC	-	0.069	0.0741	0.628	100.9
Rxn #50	AnalytiCon Plate #15	B6	50	0.606	0.0741	0.628	4.0
Rxn #50	AnalytiCon Plate #15	C6	50	0.614	0.0741	0.628	2.5
Rxn #50	AnalytiCon Plate #15	D6	50	0.613	0.0741	0.628	2.7
Rxn #50	AnalytiCon Plate #15	E6	50	0.602	0.0741	0.628	4.7
Rxn #50	AnalytiCon Plate #15	F6	50	0.586	0.0741	0.628	7.6
Rxn #50	AnalytiCon Plate #15	G6	50	0.608	0.0741	0.628	3.6
Rxn #50	AnalytiCon Plate #15	H6: FOC	-	0.620	0.0741	0.628	1.4
Rxn #50	AnalytiCon Plate #15	A7: NFC	-	0.072	0.0741	0.628	100.4
Rxn #50	AnalytiCon Plate #15	B7	50	0.564	0.0741	0.628	11.6
Rxn #50	AnalytiCon Plate #15	C7	50	0.623	0.0741	0.628	0.9
Rxn #50	AnalytiCon Plate #15	D7	50	0.553	0.0741	0.628	13.5
Rxn #50	AnalytiCon Plate #15	E7	50	0.631	0.0741	0.628	-0.5
Rxn #50	AnalytiCon Plate #15	F7	50	0.622	0.0741	0.628	1.1
Rxn #50	AnalytiCon Plate #15	G7	50	0.624	0.0741	0.628	0.7
Rxn #50	AnalytiCon Plate #15	H7: FOC	-	0.648	0.0741	0.628	-3.6
Rxn #50	AnalytiCon Plate #15	A8: NFC	-	0.065	0.0741	0.628	101.6
Rxn #50	AnalytiCon Plate #15	B8	50	0.622	0.0741	0.628	1.1
Rxn #50	AnalytiCon Plate #15	C8	50	0.625	0.0741	0.628	0.5
Rxn #50	AnalytiCon Plate #15	D8	50	0.635	0.0741	0.628	-1.3
Rxn #50	AnalytiCon Plate #15	E8	50	0.629	0.0741	0.628	-0.2
Rxn #50	AnalytiCon Plate #15	F8	50	0.633	0.0741	0.628	-0.9
Rxn #50	AnalytiCon Plate #15	G8	50	0.612	0.0741	0.628	2.9
Rxn #50	AnalytiCon Plate #15	H8: FOC	-	0.651	0.0741	0.628	-4.2
Rxn #50	AnalytiCon Plate #15	A9: NFC	-	0.075	0.0741	0.628	99.8
Rxn #50	AnalytiCon Plate #15	B9	50	0.574	0.0741	0.628	9.7
Rxn #50	AnalytiCon Plate #15	C9	50	0.651	0.0741	0.628	-4.2
Rxn #50	AnalytiCon Plate #15	D9	50	0.617	0.0741	0.628	2.0
Rxn #50	AnalytiCon Plate #15	E9	50	0.630	0.0741	0.628	-0.4
Rxn #50	AnalytiCon Plate #15	F9	50	0.630	0.0741	0.628	-0.4
Rxn #50	AnalytiCon Plate #15	G9	50	0.636	0.0741	0.628	-1.4
Rxn #50	AnalytiCon Plate #15	H9: FOC	-	0.650	0.0741	0.628	-4.0

S3 Table. Data from Screening of Phytochemical Library for Inhibition of KHKC Activity.

Rxn ID	Sample			Sample	Ave. No Fructose Controls	Ave. Fructose Only Controls	KHKC
	Phytochemical Plate	Well	Conc (μM)	(ΔOD_{340nm})	(ΔOD_{340nm})	(ΔOD_{340nm})	Inhibition (%)
Rxn #50	AnalytiCon Plate #15	A10: NFC	-	0.067	0.0741	0.628	101.3
Rxn #50	AnalytiCon Plate #15	B10	50	0.524	0.0741	0.628	18.8
Rxn #50	AnalytiCon Plate #15	C10	50	0.623	0.0741	0.628	0.9
Rxn #50	AnalytiCon Plate #15	D10	50	0.622	0.0741	0.628	1.1
Rxn #50	AnalytiCon Plate #15	E10	50	0.623	0.0741	0.628	0.9
Rxn #50	AnalytiCon Plate #15	F10	50	0.607	0.0741	0.628	3.8
Rxn #50	AnalytiCon Plate #15	G10	50	0.605	0.0741	0.628	4.2
Rxn #50	AnalytiCon Plate #15	H10: FOC	-	0.636	0.0741	0.628	-1.4
Rxn #50	AnalytiCon Plate #15	A11: NFC	-	0.072	0.0741	0.628	100.4
Rxn #50	AnalytiCon Plate #15	B11	50	0.633	0.0741	0.628	-0.9
Rxn #50	AnalytiCon Plate #15	C11	50	0.636	0.0741	0.628	-1.4
Rxn #50	AnalytiCon Plate #15	D11	50	0.632	0.0741	0.628	-0.7
Rxn #50	AnalytiCon Plate #15	E11	50	0.626	0.0741	0.628	0.4
Rxn #50	AnalytiCon Plate #15	F11	50	0.627	0.0741	0.628	0.2
Rxn #50	AnalytiCon Plate #15	G11	50	0.663	0.0741	0.628	-6.3
Rxn #50	AnalytiCon Plate #15	H11: FOC	-	0.640	0.0741	0.628	-2.2
Rxn #50	AnalytiCon Plate #15	A12: NFC	-	0.059	0.0741	0.628	102.7
Rxn #50	AnalytiCon Plate #15	H12: FOC	-	0.636	0.0741	0.628	-1.4
Rxn #51	AnalytiCon Plate #16	A1: NFC	-	0.103	0.0833	0.632	96.4
Rxn #51	AnalytiCon Plate #16	B1	50	0.590	0.0833	0.632	7.7
Rxn #51	AnalytiCon Plate #16	C1	50	0.546	0.0833	0.632	15.7
Rxn #51	AnalytiCon Plate #16	D1	50	0.645	0.0833	0.632	-2.4
Rxn #51	AnalytiCon Plate #16	E1	50	0.635	0.0833	0.632	-0.5
Rxn #51	AnalytiCon Plate #16	F1	50	0.626	0.0833	0.632	1.1
Rxn #51	AnalytiCon Plate #16	G1	50	0.650	0.0833	0.632	-3.3
Rxn #51	AnalytiCon Plate #16	H1: FOC	-	0.642	0.0833	0.632	-1.8
Rxn #51	AnalytiCon Plate #16	A2: NFC	-	0.095	0.0833	0.632	97.9
Rxn #51	AnalytiCon Plate #16	B2	50	0.644	0.0833	0.632	-2.2
Rxn #51	AnalytiCon Plate #16	C2	50	0.622	0.0833	0.632	1.8
Rxn #51	AnalytiCon Plate #16	D2	50	0.651	0.0833	0.632	-3.5
Rxn #51	AnalytiCon Plate #16	E2	50	0.642	0.0833	0.632	-1.8
Rxn #51	AnalytiCon Plate #16	F2	50	0.609	0.0833	0.632	4.2
Rxn #51	AnalytiCon Plate #16	G2	50	0.629	0.0833	0.632	0.5
Rxn #51	AnalytiCon Plate #16	H2: FOC	-	0.618	0.0833	0.632	2.6
Rxn #51	AnalytiCon Plate #16	A3: NFC	-	0.116	0.0833	0.632	94.0
Rxn #51	AnalytiCon Plate #16	B3	50	0.577	0.0833	0.632	10.0
Rxn #51	AnalytiCon Plate #16	C3	50	0.608	0.0833	0.632	4.4
Rxn #51	AnalytiCon Plate #16	D3	50	0.612	0.0833	0.632	3.6
Rxn #51	AnalytiCon Plate #16	E3	50	0.614	0.0833	0.632	3.3
Rxn #51	AnalytiCon Plate #16	F3	50	0.557	0.0833	0.632	13.7
Rxn #51	AnalytiCon Plate #16	G3	50	0.608	0.0833	0.632	4.4
Rxn #51	AnalytiCon Plate #16	H3: FOC	-	0.629	0.0833	0.632	0.5
Rxn #51	AnalytiCon Plate #16	A4: NFC	-	0.106	0.0833	0.632	95.9
Rxn #51	AnalytiCon Plate #16	B4	50	0.619	0.0833	0.632	2.4
Rxn #51	AnalytiCon Plate #16	C4	50	0.574	0.0833	0.632	10.6
Rxn #51	AnalytiCon Plate #16	D4	50	0.600	0.0833	0.632	5.8
Rxn #51	AnalytiCon Plate #16	E4	50	0.624	0.0833	0.632	1.5
Rxn #51	AnalytiCon Plate #16	F4	50	0.606	0.0833	0.632	4.7
Rxn #51	AnalytiCon Plate #16	G4	50	0.607	0.0833	0.632	4.6
Rxn #51	AnalytiCon Plate #16	H4: FOC	-	0.634	0.0833	0.632	-0.4
Rxn #51	AnalytiCon Plate #16	A5: NFC	-	0.117	0.0833	0.632	93.9
Rxn #51	AnalytiCon Plate #16	B5: NFC	-	0.071	0.0833	0.632	102.2
Rxn #51	AnalytiCon Plate #16	C5: NFC	-	0.073	0.0833	0.632	101.9
Rxn #51	AnalytiCon Plate #16	D5: NFC	-	0.067	0.0833	0.632	103.0
Rxn #51	AnalytiCon Plate #16	E5: FOC	-	0.635	0.0833	0.632	-0.5
Rxn #51	AnalytiCon Plate #16	F5: FOC	-	0.635	0.0833	0.632	-0.5
Rxn #51	AnalytiCon Plate #16	G5: FOC	-	0.634	0.0833	0.632	-0.4
Rxn #51	AnalytiCon Plate #16	H5: FOC	-	0.637	0.0833	0.632	-0.9
Rxn #51	AnalytiCon Plate #16	A6: NFC	-	0.070	0.0833	0.632	102.4
Rxn #51	AnalytiCon Plate #16	B6	50	0.620	0.0833	0.632	2.2
Rxn #51	AnalytiCon Plate #16	C6	50	0.577	0.0833	0.632	10.0
Rxn #51	AnalytiCon Plate #16	D6	50	0.619	0.0833	0.632	2.4
Rxn #51	AnalytiCon Plate #16	E6	50	0.635	0.0833	0.632	-0.5
Rxn #51	AnalytiCon Plate #16	F6	50	0.651	0.0833	0.632	-3.5
Rxn #51	AnalytiCon Plate #16	G6	50	0.628	0.0833	0.632	0.7
Rxn #51	AnalytiCon Plate #16	H6: FOC	-	0.639	0.0833	0.632	-1.3
Rxn #51	AnalytiCon Plate #16	A7: NFC	-	0.087	0.0833	0.632	99.3
Rxn #51	AnalytiCon Plate #16	B7	50	0.604	0.0833	0.632	5.1
Rxn #51	AnalytiCon Plate #16	C7	50	0.628	0.0833	0.632	0.7
Rxn #51	AnalytiCon Plate #16	D7	50	0.651	0.0833	0.632	-3.5
Rxn #51	AnalytiCon Plate #16	E7	50	0.650	0.0833	0.632	-3.3
Rxn #51	AnalytiCon Plate #16	F7	50	0.611	0.0833	0.632	3.8
Rxn #51	AnalytiCon Plate #16	G7	50	0.632	0.0833	0.632	0.0
Rxn #51	AnalytiCon Plate #16	H7: FOC	-	0.614	0.0833	0.632	3.3

S3 Table. Data from Screening of Phytochemical Library for Inhibition of KHKC Activity.

Rxn ID	Sample			Sample	Ave. No Fructose Controls	Ave. Fructose Only Controls	KHKC
	Phytochemical Plate	Well	Conc (μM)	(ΔOD_{340nm})	(ΔOD_{340nm})	(ΔOD_{340nm})	Inhibition (%)
Rxn #51	AnalytiCon Plate #16	A8: NFC	-	0.079	0.0833	0.632	100.8
Rxn #51	AnalytiCon Plate #16	B8	50	0.609	0.0833	0.632	4.2
Rxn #51	AnalytiCon Plate #16	C8	50	0.644	0.0833	0.632	-2.2
Rxn #51	AnalytiCon Plate #16	D8	50	0.542	0.0833	0.632	16.4
Rxn #51	AnalytiCon Plate #16	E8	50	0.604	0.0833	0.632	5.1
Rxn #51	AnalytiCon Plate #16	F8	50	0.616	0.0833	0.632	2.9
Rxn #51	AnalytiCon Plate #16	G8	50	0.633	0.0833	0.632	-0.2
Rxn #51	AnalytiCon Plate #16	H8: FOC	-	0.634	0.0833	0.632	-0.4
Rxn #51	AnalytiCon Plate #16	A9: NFC	-	0.063	0.0833	0.632	103.7
Rxn #51	AnalytiCon Plate #16	B9	50	0.659	0.0833	0.632	-4.9
Rxn #51	AnalytiCon Plate #16	C9	50	0.619	0.0833	0.632	2.4
Rxn #51	AnalytiCon Plate #16	D9	50	0.653	0.0833	0.632	-3.8
Rxn #51	AnalytiCon Plate #16	E9	50	0.592	0.0833	0.632	7.3
Rxn #51	AnalytiCon Plate #16	F9	50	0.633	0.0833	0.632	-0.2
Rxn #51	AnalytiCon Plate #16	G9	50	0.618	0.0833	0.632	2.6
Rxn #51	AnalytiCon Plate #16	H9: FOC	-	0.655	0.0833	0.632	-4.2
Rxn #51	AnalytiCon Plate #16	A10: NFC	-	0.070	0.0833	0.632	102.4
Rxn #51	AnalytiCon Plate #16	B10	50	0.579	0.0833	0.632	9.7
Rxn #51	AnalytiCon Plate #16	C10	50	0.621	0.0833	0.632	2.0
Rxn #51	AnalytiCon Plate #16	D10	50	0.612	0.0833	0.632	3.6
Rxn #51	AnalytiCon Plate #16	E10	50	0.639	0.0833	0.632	-1.3
Rxn #51	AnalytiCon Plate #16	F10	50	0.621	0.0833	0.632	2.0
Rxn #51	AnalytiCon Plate #16	G10	50	0.616	0.0833	0.632	2.9
Rxn #51	AnalytiCon Plate #16	H10: FOC	-	0.598	0.0833	0.632	6.2
Rxn #51	AnalytiCon Plate #16	A11: NFC	-	0.068	0.0833	0.632	102.8
Rxn #51	AnalytiCon Plate #16	B11	50	0.604	0.0833	0.632	5.1
Rxn #51	AnalytiCon Plate #16	C11	50	0.595	0.0833	0.632	6.7
Rxn #51	AnalytiCon Plate #16	D11	50	0.626	0.0833	0.632	1.1
Rxn #51	AnalytiCon Plate #16	E11	50	0.583	0.0833	0.632	8.9
Rxn #51	AnalytiCon Plate #16	F11	50	0.622	0.0833	0.632	1.8
Rxn #51	AnalytiCon Plate #16	G11	50	0.635	0.0833	0.632	-0.5
Rxn #51	AnalytiCon Plate #16	H11: FOC	-	0.603	0.0833	0.632	5.3
Rxn #51	AnalytiCon Plate #16	A12: NFC	-	0.065	0.0833	0.632	103.3
Rxn #51	AnalytiCon Plate #16	H12: FOC	-	0.577	0.0833	0.632	10.0
Rxn #52	AnalytiCon Plate #17	A1: NFC	-	0.068	0.0598	0.561	98.4
Rxn #52	AnalytiCon Plate #17	B1	50	0.509	0.0598	0.561	10.4
Rxn #52	AnalytiCon Plate #17	C1	50	0.465	0.0598	0.561	19.2
Rxn #52	AnalytiCon Plate #17	D1	50	0.522	0.0598	0.561	7.8
Rxn #52	AnalytiCon Plate #17	E1	50	0.519	0.0598	0.561	8.4
Rxn #52	AnalytiCon Plate #17	F1	50	0.535	0.0598	0.561	5.2
Rxn #52	AnalytiCon Plate #17	G1	50	0.520	0.0598	0.561	8.2
Rxn #52	AnalytiCon Plate #17	H1: FOC	-	0.524	0.0598	0.561	7.4
Rxn #52	AnalytiCon Plate #17	A2: NFC	-	0.070	0.0598	0.561	98.0
Rxn #52	AnalytiCon Plate #17	B2	50	0.573	0.0598	0.561	-2.4
Rxn #52	AnalytiCon Plate #17	C2	50	0.499	0.0598	0.561	12.4
Rxn #52	AnalytiCon Plate #17	D2	50	0.559	0.0598	0.561	0.4
Rxn #52	AnalytiCon Plate #17	E2	50	0.512	0.0598	0.561	9.8
Rxn #52	AnalytiCon Plate #17	F2	50	0.547	0.0598	0.561	2.8
Rxn #52	AnalytiCon Plate #17	G2	50	0.405	0.0598	0.561	31.1
Rxn #52	AnalytiCon Plate #17	H2: FOC	-	0.556	0.0598	0.561	1.0
Rxn #52	AnalytiCon Plate #17	A3: NFC	-	0.064	0.0598	0.561	99.2
Rxn #52	AnalytiCon Plate #17	B3	50	0.565	0.0598	0.561	-0.8
Rxn #52	AnalytiCon Plate #17	C3	50	0.503	0.0598	0.561	11.6
Rxn #52	AnalytiCon Plate #17	D3	50	0.539	0.0598	0.561	4.4
Rxn #52	AnalytiCon Plate #17	E3	50	0.550	0.0598	0.561	2.2
Rxn #52	AnalytiCon Plate #17	F3	50	0.562	0.0598	0.561	-0.2
Rxn #52	AnalytiCon Plate #17	G3	50	0.458	0.0598	0.561	20.6
Rxn #52	AnalytiCon Plate #17	H3: FOC	-	0.565	0.0598	0.561	-0.8
Rxn #52	AnalytiCon Plate #17	A4: NFC	-	0.064	0.0598	0.561	99.2
Rxn #52	AnalytiCon Plate #17	B4	50	0.474	0.0598	0.561	17.4
Rxn #52	AnalytiCon Plate #17	C4	50	0.454	0.0598	0.561	21.3
Rxn #52	AnalytiCon Plate #17	D4	50	0.533	0.0598	0.561	5.6
Rxn #52	AnalytiCon Plate #17	E4	50	0.563	0.0598	0.561	-0.4
Rxn #52	AnalytiCon Plate #17	F4	50	0.551	0.0598	0.561	2.0
Rxn #52	AnalytiCon Plate #17	G4	50	0.553	0.0598	0.561	1.6
Rxn #52	AnalytiCon Plate #17	H4: FOC	-	0.574	0.0598	0.561	-2.6
Rxn #52	AnalytiCon Plate #17	A5: NFC	-	0.054	0.0598	0.561	101.2
Rxn #52	AnalytiCon Plate #17	B5: NFC	-	0.063	0.0598	0.561	99.4
Rxn #52	AnalytiCon Plate #17	C5: NFC	-	0.052	0.0598	0.561	101.6
Rxn #52	AnalytiCon Plate #17	D5: NFC	-	0.061	0.0598	0.561	99.8
Rxn #52	AnalytiCon Plate #17	E5: FOC	-	0.575	0.0598	0.561	-2.8
Rxn #52	AnalytiCon Plate #17	F5: FOC	-	0.570	0.0598	0.561	-1.8
Rxn #52	AnalytiCon Plate #17	G5: FOC	-	0.562	0.0598	0.561	-0.2
Rxn #52	AnalytiCon Plate #17	H5: FOC	-	0.564	0.0598	0.561	-0.6

S3 Table. Data from Screening of Phytochemical Library for Inhibition of KHKC Activity.

Rxn ID	Sample			Sample	Ave. No Fructose Controls	Ave. Fructose Only Controls	KHKC
	Phytochemical Plate	Well	Conc (μM)	(ΔOD_{340nm})	(ΔOD_{340nm})	(ΔOD_{340nm})	Inhibition (%)
Rxn #52	AnalytiCon Plate #17	A6: NFC	-	0.060	0.0598	0.561	100.0
Rxn #52	AnalytiCon Plate #17	B6	50	0.573	0.0598	0.561	-2.4
Rxn #52	AnalytiCon Plate #17	C6	50	0.496	0.0598	0.561	13.0
Rxn #52	AnalytiCon Plate #17	D6	50	0.408	0.0598	0.561	30.5
Rxn #52	AnalytiCon Plate #17	E6	50	0.519	0.0598	0.561	8.4
Rxn #52	AnalytiCon Plate #17	F6	50	0.585	0.0598	0.561	-4.8
Rxn #52	AnalytiCon Plate #17	G6	50	0.545	0.0598	0.561	3.2
Rxn #52	AnalytiCon Plate #17	H6: FOC	-	0.564	0.0598	0.561	-0.6
Rxn #52	AnalytiCon Plate #17	A7: NFC	-	0.062	0.0598	0.561	99.6
Rxn #52	AnalytiCon Plate #17	B7	50	0.578	0.0598	0.561	-3.4
Rxn #52	AnalytiCon Plate #17	C7	50	0.500	0.0598	0.561	12.2
Rxn #52	AnalytiCon Plate #17	D7	50	0.548	0.0598	0.561	2.6
Rxn #52	AnalytiCon Plate #17	E7	50	0.541	0.0598	0.561	4.0
Rxn #52	AnalytiCon Plate #17	F7	50	0.522	0.0598	0.561	7.8
Rxn #52	AnalytiCon Plate #17	G7	50	0.576	0.0598	0.561	-3.0
Rxn #52	AnalytiCon Plate #17	H7: FOC	-	0.554	0.0598	0.561	1.4
Rxn #52	AnalytiCon Plate #17	A8: NFC	-	0.056	0.0598	0.561	100.8
Rxn #52	AnalytiCon Plate #17	B8	50	0.569	0.0598	0.561	-1.6
Rxn #52	AnalytiCon Plate #17	C8	50	0.511	0.0598	0.561	10.0
Rxn #52	AnalytiCon Plate #17	D8	50	0.599	0.0598	0.561	-7.6
Rxn #52	AnalytiCon Plate #17	E8	50	0.566	0.0598	0.561	-1.0
Rxn #52	AnalytiCon Plate #17	F8	50	0.565	0.0598	0.561	-0.8
Rxn #52	AnalytiCon Plate #17	G8	50	0.417	0.0598	0.561	28.7
Rxn #52	AnalytiCon Plate #17	H8: FOC	-	0.586	0.0598	0.561	-5.0
Rxn #52	AnalytiCon Plate #17	A9: NFC	-	0.060	0.0598	0.561	100.0
Rxn #52	AnalytiCon Plate #17	B9	50	0.582	0.0598	0.561	-4.2
Rxn #52	AnalytiCon Plate #17	C9	50	0.481	0.0598	0.561	16.0
Rxn #52	AnalytiCon Plate #17	D9	50	0.571	0.0598	0.561	-2.0
Rxn #52	AnalytiCon Plate #17	E9	50	0.566	0.0598	0.561	-1.0
Rxn #52	AnalytiCon Plate #17	F9	50	0.524	0.0598	0.561	7.4
Rxn #52	AnalytiCon Plate #17	G9	50	0.563	0.0598	0.561	-0.4
Rxn #52	AnalytiCon Plate #17	H9: FOC	-	0.566	0.0598	0.561	-1.0
Rxn #52	AnalytiCon Plate #17	A10: NFC	-	0.052	0.0598	0.561	101.6
Rxn #52	AnalytiCon Plate #17	B10	50	0.480	0.0598	0.561	16.2
Rxn #52	AnalytiCon Plate #17	C10	50	0.464	0.0598	0.561	19.4
Rxn #52	AnalytiCon Plate #17	D10	50	0.556	0.0598	0.561	1.0
Rxn #52	AnalytiCon Plate #17	E10	50	0.528	0.0598	0.561	6.6
Rxn #52	AnalytiCon Plate #17	F10	50	0.547	0.0598	0.561	2.8
Rxn #52	AnalytiCon Plate #17	G10	50	0.557	0.0598	0.561	0.8
Rxn #52	AnalytiCon Plate #17	H10: FOC	-	0.542	0.0598	0.561	3.8
Rxn #52	AnalytiCon Plate #17	A11: NFC	-	0.055	0.0598	0.561	101.0
Rxn #52	AnalytiCon Plate #17	B11	50	0.517	0.0598	0.561	8.8
Rxn #52	AnalytiCon Plate #17	C11	50	0.486	0.0598	0.561	15.0
Rxn #52	AnalytiCon Plate #17	D11	50	0.534	0.0598	0.561	5.4
Rxn #52	AnalytiCon Plate #17	E11	50	0.524	0.0598	0.561	7.4
Rxn #52	AnalytiCon Plate #17	F11	50	0.561	0.0598	0.561	0.0
Rxn #52	AnalytiCon Plate #17	G11	50	0.549	0.0598	0.561	2.4
Rxn #52	AnalytiCon Plate #17	H11: FOC	-	0.575	0.0598	0.561	-2.8
Rxn #52	AnalytiCon Plate #17	A12: NFC	-	0.056	0.0598	0.561	100.8
Rxn #52	AnalytiCon Plate #17	H12: FOC	-	0.554	0.0598	0.561	1.4
Rxn #53	AnalytiCon Plate #18	A1: NFC	-	0.099	0.0772	0.533	95.2
Rxn #53	AnalytiCon Plate #18	B1	50	0.487	0.0772	0.533	10.1
Rxn #53	AnalytiCon Plate #18	C1	50	0.534	0.0772	0.533	-0.2
Rxn #53	AnalytiCon Plate #18	D1	50	0.572	0.0772	0.533	-8.6
Rxn #53	AnalytiCon Plate #18	E1	50	0.562	0.0772	0.533	-6.4
Rxn #53	AnalytiCon Plate #18	F1	50	0.574	0.0772	0.533	-9.0
Rxn #53	AnalytiCon Plate #18	G1	50	0.605	0.0772	0.533	-15.8
Rxn #53	AnalytiCon Plate #18	H1: FOC	-	0.554	0.0772	0.533	-4.6
Rxn #53	AnalytiCon Plate #18	A2: NFC	-	0.065	0.0772	0.533	102.7
Rxn #53	AnalytiCon Plate #18	B2	50	0.624	0.0772	0.533	-20.0
Rxn #53	AnalytiCon Plate #18	C2	50	0.587	0.0772	0.533	-11.8
Rxn #53	AnalytiCon Plate #18	D2	50	0.574	0.0772	0.533	-9.0
Rxn #53	AnalytiCon Plate #18	E2	50	0.561	0.0772	0.533	-6.1
Rxn #53	AnalytiCon Plate #18	F2	50	0.582	0.0772	0.533	-10.8
Rxn #53	AnalytiCon Plate #18	G2	50	0.530	0.0772	0.533	0.7
Rxn #53	AnalytiCon Plate #18	H2: FOC	-	0.557	0.0772	0.533	-5.3
Rxn #53	AnalytiCon Plate #18	A3: NFC	-	0.073	0.0772	0.533	100.9
Rxn #53	AnalytiCon Plate #18	B3	50	0.534	0.0772	0.533	-0.2
Rxn #53	AnalytiCon Plate #18	C3	50	0.576	0.0772	0.533	-9.4
Rxn #53	AnalytiCon Plate #18	D3	50	0.511	0.0772	0.533	4.8
Rxn #53	AnalytiCon Plate #18	E3	50	0.507	0.0772	0.533	5.7
Rxn #53	AnalytiCon Plate #18	F3	50	0.595	0.0772	0.533	-13.6
Rxn #53	AnalytiCon Plate #18	G3	50	0.556	0.0772	0.533	-5.0
Rxn #53	AnalytiCon Plate #18	H3: FOC	-	0.564	0.0772	0.533	-6.8

S3 Table. Data from Screening of Phytochemical Library for Inhibition of KHKC Activity.

Rxn ID	Sample			Sample	Ave. No Fructose Controls	Ave. Fructose Only Controls	KHKC
	Phytochemical Plate	Well	Conc (μM)	(ΔOD_{340nm})	(ΔOD_{340nm})	(ΔOD_{340nm})	Inhibition (%)
Rxn #53	AnalytiCon Plate #18	A4: NFC	-	0.101	0.0772	0.533	94.8
Rxn #53	AnalytiCon Plate #18	B4	50	0.566	0.0772	0.533	-7.2
Rxn #53	AnalytiCon Plate #18	C4	50	0.563	0.0772	0.533	-6.6
Rxn #53	AnalytiCon Plate #18	D4	50	0.573	0.0772	0.533	-8.8
Rxn #53	AnalytiCon Plate #18	E4	50	0.471	0.0772	0.533	13.6
Rxn #53	AnalytiCon Plate #18	F4	50	0.583	0.0772	0.533	-11.0
Rxn #53	AnalytiCon Plate #18	G4	50	0.517	0.0772	0.533	3.5
Rxn #53	AnalytiCon Plate #18	H4: FOC	-	0.220	0.0772	0.533	68.7
Rxn #53	AnalytiCon Plate #18	A5: NFC	-	0.083	0.0772	0.533	98.7
Rxn #53	AnalytiCon Plate #18	B5: NFC	-	0.072	0.0772	0.533	101.1
Rxn #53	AnalytiCon Plate #18	C5: NFC	-	0.069	0.0772	0.533	101.8
Rxn #53	AnalytiCon Plate #18	D5: NFC	-	0.067	0.0772	0.533	102.2
Rxn #53	AnalytiCon Plate #18	E5: FOC	-	0.583	0.0772	0.533	-11.0
Rxn #53	AnalytiCon Plate #18	F5: FOC	-	0.566	0.0772	0.533	-7.2
Rxn #53	AnalytiCon Plate #18	G5: FOC	-	0.582	0.0772	0.533	-10.8
Rxn #53	AnalytiCon Plate #18	H5: FOC	-	0.558	0.0772	0.533	-5.5
Rxn #53	AnalytiCon Plate #18	A6: NFC	-	0.077	0.0772	0.533	100.0
Rxn #53	AnalytiCon Plate #18	B6	50	0.546	0.0772	0.533	-2.9
Rxn #53	AnalytiCon Plate #18	C6	50	0.561	0.0772	0.533	-6.1
Rxn #53	AnalytiCon Plate #18	D6	50	0.589	0.0772	0.533	-12.3
Rxn #53	AnalytiCon Plate #18	E6	50	0.588	0.0772	0.533	-12.1
Rxn #53	AnalytiCon Plate #18	F6	50	0.571	0.0772	0.533	-8.3
Rxn #53	AnalytiCon Plate #18	G6	50	0.519	0.0772	0.533	3.1
Rxn #53	AnalytiCon Plate #18	H6: FOC	-	0.575	0.0772	0.533	-9.2
Rxn #53	AnalytiCon Plate #18	A7: NFC	-	0.064	0.0772	0.533	102.9
Rxn #53	AnalytiCon Plate #18	B7	50	0.596	0.0772	0.533	-13.8
Rxn #53	AnalytiCon Plate #18	C7	50	0.533	0.0772	0.533	0.0
Rxn #53	AnalytiCon Plate #18	D7	50	0.593	0.0772	0.533	-13.2
Rxn #53	AnalytiCon Plate #18	E7	50	0.639	0.0772	0.533	-23.3
Rxn #53	AnalytiCon Plate #18	F7	50	0.605	0.0772	0.533	-15.8
Rxn #53	AnalytiCon Plate #18	G7	50	0.580	0.0772	0.533	-10.3
Rxn #53	AnalytiCon Plate #18	H7: FOC	-	0.570	0.0772	0.533	-8.1
Rxn #53	AnalytiCon Plate #18	A8: NFC	-	0.063	0.0772	0.533	103.1
Rxn #53	AnalytiCon Plate #18	B8	50	0.592	0.0772	0.533	-12.9
Rxn #53	AnalytiCon Plate #18	C8	50	0.551	0.0772	0.533	-3.9
Rxn #53	AnalytiCon Plate #18	D8	50	0.576	0.0772	0.533	-9.4
Rxn #53	AnalytiCon Plate #18	E8	50	0.581	0.0772	0.533	-10.5
Rxn #53	AnalytiCon Plate #18	F8	50	0.585	0.0772	0.533	-11.4
Rxn #53	AnalytiCon Plate #18	G8	50	0.587	0.0772	0.533	-11.8
Rxn #53	AnalytiCon Plate #18	H8: FOC	-	0.572	0.0772	0.533	-8.6
Rxn #53	AnalytiCon Plate #18	A9: NFC	-	0.075	0.0772	0.533	100.5
Rxn #53	AnalytiCon Plate #18	B9	50	0.575	0.0772	0.533	-9.2
Rxn #53	AnalytiCon Plate #18	C9	50	0.537	0.0772	0.533	-0.9
Rxn #53	AnalytiCon Plate #18	D9	50	0.556	0.0772	0.533	-5.0
Rxn #53	AnalytiCon Plate #18	E9	50	0.576	0.0772	0.533	-9.4
Rxn #53	AnalytiCon Plate #18	F9	50	0.575	0.0772	0.533	-9.2
Rxn #53	AnalytiCon Plate #18	G9	50	0.580	0.0772	0.533	-10.3
Rxn #53	AnalytiCon Plate #18	H9: FOC	-	0.588	0.0772	0.533	-12.1
Rxn #53	AnalytiCon Plate #18	A10: NFC	-	0.081	0.0772	0.533	99.2
Rxn #53	AnalytiCon Plate #18	B10	50	0.605	0.0772	0.533	-15.8
Rxn #53	AnalytiCon Plate #18	C10	50	0.504	0.0772	0.533	6.4
Rxn #53	AnalytiCon Plate #18	D10	50	0.547	0.0772	0.533	-3.1
Rxn #53	AnalytiCon Plate #18	E10	50	0.547	0.0772	0.533	-3.1
Rxn #53	AnalytiCon Plate #18	F10	50	0.563	0.0772	0.533	-6.6
Rxn #53	AnalytiCon Plate #18	G10	50	0.551	0.0772	0.533	-3.9
Rxn #53	AnalytiCon Plate #18	H10: FOC	-	0.555	0.0772	0.533	-4.8
Rxn #53	AnalytiCon Plate #18	A11: NFC	-	0.068	0.0772	0.533	102.0
Rxn #53	AnalytiCon Plate #18	B11	50	0.570	0.0772	0.533	-8.1
Rxn #53	AnalytiCon Plate #18	C11	50	0.464	0.0772	0.533	15.1
Rxn #53	AnalytiCon Plate #18	D11	50	0.549	0.0772	0.533	-3.5
Rxn #53	AnalytiCon Plate #18	E11	50	0.575	0.0772	0.533	-9.2
Rxn #53	AnalytiCon Plate #18	F11	50	0.543	0.0772	0.533	-2.2
Rxn #53	AnalytiCon Plate #18	G11	50	0.565	0.0772	0.533	-7.0
Rxn #53	AnalytiCon Plate #18	H11: FOC	-	0.547	0.0772	0.533	-3.1
Rxn #53	AnalytiCon Plate #18	A12: NFC	-	0.101	0.0772	0.533	94.8
Rxn #53	AnalytiCon Plate #18	H12: FOC	-	0.550	0.0772	0.533	-3.7
Rxn #54	AnalytiCon Plate #19	A1: NFC	-	0.047	0.0518	0.587	100.9
Rxn #54	AnalytiCon Plate #19	B1	50	0.475	0.0518	0.587	20.9
Rxn #54	AnalytiCon Plate #19	C1	50	0.558	0.0518	0.587	5.4
Rxn #54	AnalytiCon Plate #19	D1	50	0.551	0.0518	0.587	6.7
Rxn #54	AnalytiCon Plate #19	E1	50	0.439	0.0518	0.587	27.7
Rxn #54	AnalytiCon Plate #19	F1	50	0.549	0.0518	0.587	7.1
Rxn #54	AnalytiCon Plate #19	G1	50	0.541	0.0518	0.587	8.6
Rxn #54	AnalytiCon Plate #19	H1: FOC	-	0.538	0.0518	0.587	9.2

S3 Table. Data from Screening of Phytochemical Library for Inhibition of KHKC Activity.

Rxn ID	Sample			Sample	Ave. No Fructose Controls	Ave. Fructose Only Controls	KHKC
	Phytochemical Plate	Well	Conc (μM)	(ΔOD_{340nm})	(ΔOD_{340nm})	(ΔOD_{340nm})	Inhibition (%)
Rxn #54	AnalytiCon Plate #19	A2: NFC	-	0.048	0.0518	0.587	100.7
Rxn #54	AnalytiCon Plate #19	B2	50	0.584	0.0518	0.587	0.6
Rxn #54	AnalytiCon Plate #19	C2	50	0.529	0.0518	0.587	10.8
Rxn #54	AnalytiCon Plate #19	D2	50	0.549	0.0518	0.587	7.1
Rxn #54	AnalytiCon Plate #19	E2	50	0.562	0.0518	0.587	4.7
Rxn #54	AnalytiCon Plate #19	F2	50	0.469	0.0518	0.587	22.0
Rxn #54	AnalytiCon Plate #19	G2	50	0.462	0.0518	0.587	23.4
Rxn #54	AnalytiCon Plate #19	H2: FOC	-	0.592	0.0518	0.587	-0.9
Rxn #54	AnalytiCon Plate #19	A3: NFC	-	0.055	0.0518	0.587	99.4
Rxn #54	AnalytiCon Plate #19	B3	50	0.590	0.0518	0.587	-0.6
Rxn #54	AnalytiCon Plate #19	C3	50	0.597	0.0518	0.587	-1.9
Rxn #54	AnalytiCon Plate #19	D3	50	0.622	0.0518	0.587	-6.5
Rxn #54	AnalytiCon Plate #19	E3	50	0.750	0.0518	0.587	-30.5
Rxn #54	AnalytiCon Plate #19	F3	50	0.580	0.0518	0.587	1.3
Rxn #54	AnalytiCon Plate #19	G3	50	0.574	0.0518	0.587	2.4
Rxn #54	AnalytiCon Plate #19	H3: FOC	-	0.573	0.0518	0.587	2.6
Rxn #54	AnalytiCon Plate #19	A4: NFC	-	0.054	0.0518	0.587	99.6
Rxn #54	AnalytiCon Plate #19	B4	50	0.567	0.0518	0.587	3.7
Rxn #54	AnalytiCon Plate #19	C4	50	0.592	0.0518	0.587	-0.9
Rxn #54	AnalytiCon Plate #19	D4	50	0.597	0.0518	0.587	-1.9
Rxn #54	AnalytiCon Plate #19	E4	50	0.632	0.0518	0.587	-8.4
Rxn #54	AnalytiCon Plate #19	F4	50	0.545	0.0518	0.587	7.8
Rxn #54	AnalytiCon Plate #19	G4	50	0.604	0.0518	0.587	-3.2
Rxn #54	AnalytiCon Plate #19	H4: FOC	-	0.606	0.0518	0.587	-3.6
Rxn #54	AnalytiCon Plate #19	A5: NFC	-	0.047	0.0518	0.587	100.9
Rxn #54	AnalytiCon Plate #19	B5: NFC	-	0.049	0.0518	0.587	100.5
Rxn #54	AnalytiCon Plate #19	C5: NFC	-	0.046	0.0518	0.587	101.1
Rxn #54	AnalytiCon Plate #19	D5: NFC	-	0.048	0.0518	0.587	100.7
Rxn #54	AnalytiCon Plate #19	E5: FOC	-	0.618	0.0518	0.587	-5.8
Rxn #54	AnalytiCon Plate #19	F5: FOC	-	0.577	0.0518	0.587	1.9
Rxn #54	AnalytiCon Plate #19	G5: FOC	-	0.573	0.0518	0.587	2.6
Rxn #54	AnalytiCon Plate #19	H5: FOC	-	0.579	0.0518	0.587	1.5
Rxn #54	AnalytiCon Plate #19	A6: NFC	-	0.089	0.0518	0.587	93.0
Rxn #54	AnalytiCon Plate #19	B6	50	0.593	0.0518	0.587	-1.1
Rxn #54	AnalytiCon Plate #19	C6	50	0.619	0.0518	0.587	-6.0
Rxn #54	AnalytiCon Plate #19	D6	50	0.591	0.0518	0.587	-0.7
Rxn #54	AnalytiCon Plate #19	E6	50	0.596	0.0518	0.587	-1.7
Rxn #54	AnalytiCon Plate #19	F6	50	0.612	0.0518	0.587	-4.7
Rxn #54	AnalytiCon Plate #19	G6	50	0.607	0.0518	0.587	-3.7
Rxn #54	AnalytiCon Plate #19	H6: FOC	-	0.612	0.0518	0.587	-4.7
Rxn #54	AnalytiCon Plate #19	A7: NFC	-	0.048	0.0518	0.587	100.7
Rxn #54	AnalytiCon Plate #19	B7	50	0.575	0.0518	0.587	2.2
Rxn #54	AnalytiCon Plate #19	C7	50	0.580	0.0518	0.587	1.3
Rxn #54	AnalytiCon Plate #19	D7	50	0.616	0.0518	0.587	-5.4
Rxn #54	AnalytiCon Plate #19	E7	50	0.536	0.0518	0.587	9.5
Rxn #54	AnalytiCon Plate #19	F7	50	0.614	0.0518	0.587	-5.0
Rxn #54	AnalytiCon Plate #19	G7	50	0.575	0.0518	0.587	2.2
Rxn #54	AnalytiCon Plate #19	H7: FOC	-	0.600	0.0518	0.587	-2.4
Rxn #54	AnalytiCon Plate #19	A8: NFC	-	0.050	0.0518	0.587	100.3
Rxn #54	AnalytiCon Plate #19	B8	50	0.572	0.0518	0.587	2.8
Rxn #54	AnalytiCon Plate #19	C8	50	0.607	0.0518	0.587	-3.7
Rxn #54	AnalytiCon Plate #19	D8	50	0.625	0.0518	0.587	-7.1
Rxn #54	AnalytiCon Plate #19	E8	50	0.616	0.0518	0.587	-5.4
Rxn #54	AnalytiCon Plate #19	F8	50	0.550	0.0518	0.587	6.9
Rxn #54	AnalytiCon Plate #19	G8	50	0.581	0.0518	0.587	1.1
Rxn #54	AnalytiCon Plate #19	H8: FOC	-	0.610	0.0518	0.587	-4.3
Rxn #54	AnalytiCon Plate #19	A9: NFC	-	0.050	0.0518	0.587	100.3
Rxn #54	AnalytiCon Plate #19	B9	50	0.560	0.0518	0.587	5.0
Rxn #54	AnalytiCon Plate #19	C9	50	0.611	0.0518	0.587	-4.5
Rxn #54	AnalytiCon Plate #19	D9	50	0.618	0.0518	0.587	-5.8
Rxn #54	AnalytiCon Plate #19	E9	50	0.572	0.0518	0.587	2.8
Rxn #54	AnalytiCon Plate #19	F9	50	0.578	0.0518	0.587	1.7
Rxn #54	AnalytiCon Plate #19	G9	50	0.537	0.0518	0.587	9.3
Rxn #54	AnalytiCon Plate #19	H9: FOC	-	0.612	0.0518	0.587	-4.7
Rxn #54	AnalytiCon Plate #19	A10: NFC	-	0.054	0.0518	0.587	99.6
Rxn #54	AnalytiCon Plate #19	B10	50	0.466	0.0518	0.587	22.6
Rxn #54	AnalytiCon Plate #19	C10	50	0.592	0.0518	0.587	-0.9
Rxn #54	AnalytiCon Plate #19	D10	50	0.533	0.0518	0.587	10.1
Rxn #54	AnalytiCon Plate #19	E10	50	0.582	0.0518	0.587	0.9
Rxn #54	AnalytiCon Plate #19	F10	50	0.602	0.0518	0.587	-2.8
Rxn #54	AnalytiCon Plate #19	G10	50	0.613	0.0518	0.587	-4.9
Rxn #54	AnalytiCon Plate #19	H10: FOC	-	0.623	0.0518	0.587	-6.7
Rxn #54	AnalytiCon Plate #19	A11: NFC	-	0.043	0.0518	0.587	101.6
Rxn #54	AnalytiCon Plate #19	B11	50	0.497	0.0518	0.587	16.8

S3 Table. Data from Screening of Phytochemical Library for Inhibition of KHKC Activity.

Rxn ID	Sample			Sample	Ave. No Fructose Controls	Ave. Fructose Only Controls	KHKC
	Phytochemical Plate	Well	Conc (μM)	(ΔOD_{340nm})	(ΔOD_{340nm})	(ΔOD_{340nm})	Inhibition (%)
Rxn #54	AnalytiCon Plate #19	C11	50	0.522	0.0518	0.587	12.1
Rxn #54	AnalytiCon Plate #19	D11	50	0.636	0.0518	0.587	-9.2
Rxn #54	AnalytiCon Plate #19	E11	50	0.578	0.0518	0.587	1.7
Rxn #54	AnalytiCon Plate #19	F11	50	0.572	0.0518	0.587	2.8
Rxn #54	AnalytiCon Plate #19	G11	50	0.555	0.0518	0.587	6.0
Rxn #54	AnalytiCon Plate #19	H11: FOC	-	0.615	0.0518	0.587	-5.2
Rxn #54	AnalytiCon Plate #19	A12: NFC	-	0.049	0.0518	0.587	100.5
Rxn #54	AnalytiCon Plate #19	H12: FOC	-	0.611	0.0518	0.587	-4.5
Rxn #55	AnalytiCon Plate #20	A1: NFC	-	0.068	0.0641	0.579	99.2
Rxn #55	AnalytiCon Plate #20	B1	50	0.516	0.0641	0.579	12.2
Rxn #55	AnalytiCon Plate #20	C1	50	0.581	0.0641	0.579	-0.4
Rxn #55	AnalytiCon Plate #20	D1	50	0.556	0.0641	0.579	4.5
Rxn #55	AnalytiCon Plate #20	E1	50	0.529	0.0641	0.579	9.7
Rxn #55	AnalytiCon Plate #20	F1	50	0.541	0.0641	0.579	7.4
Rxn #55	AnalytiCon Plate #20	G1	50	0.559	0.0641	0.579	3.9
Rxn #55	AnalytiCon Plate #20	H1: FOC	-	0.551	0.0641	0.579	5.4
Rxn #55	AnalytiCon Plate #20	A2: NFC	-	0.068	0.0641	0.579	99.2
Rxn #55	AnalytiCon Plate #20	B2	50	0.447	0.0641	0.579	25.6
Rxn #55	AnalytiCon Plate #20	C2	50	0.426	0.0641	0.579	29.7
Rxn #55	AnalytiCon Plate #20	D2	50	0.571	0.0641	0.579	1.6
Rxn #55	AnalytiCon Plate #20	E2	50	0.544	0.0641	0.579	6.8
Rxn #55	AnalytiCon Plate #20	F2	50	0.456	0.0641	0.579	23.9
Rxn #55	AnalytiCon Plate #20	G2	50	0.526	0.0641	0.579	10.3
Rxn #55	AnalytiCon Plate #20	H2: FOC	-	0.563	0.0641	0.579	3.1
Rxn #55	AnalytiCon Plate #20	A3: NFC	-	0.053	0.0641	0.579	102.2
Rxn #55	AnalytiCon Plate #20	B3	50	0.471	0.0641	0.579	21.0
Rxn #55	AnalytiCon Plate #20	C3	50	0.575	0.0641	0.579	0.8
Rxn #55	AnalytiCon Plate #20	D3	50	0.486	0.0641	0.579	18.1
Rxn #55	AnalytiCon Plate #20	E3	50	0.559	0.0641	0.579	3.9
Rxn #55	AnalytiCon Plate #20	F3	50	0.485	0.0641	0.579	18.3
Rxn #55	AnalytiCon Plate #20	G3	50	0.437	0.0641	0.579	27.6
Rxn #55	AnalytiCon Plate #20	H3: FOC	-	0.563	0.0641	0.579	3.1
Rxn #55	AnalytiCon Plate #20	A4: NFC	-	0.068	0.0641	0.579	99.2
Rxn #55	AnalytiCon Plate #20	B4	50	0.538	0.0641	0.579	8.0
Rxn #55	AnalytiCon Plate #20	C4	50	0.512	0.0641	0.579	13.0
Rxn #55	AnalytiCon Plate #20	D4	50	0.569	0.0641	0.579	1.9
Rxn #55	AnalytiCon Plate #20	E4	50	0.554	0.0641	0.579	4.9
Rxn #55	AnalytiCon Plate #20	F4	50	0.565	0.0641	0.579	2.7
Rxn #55	AnalytiCon Plate #20	G4	50	0.505	0.0641	0.579	14.4
Rxn #55	AnalytiCon Plate #20	H4: FOC	-	0.579	0.0641	0.579	0.0
Rxn #55	AnalytiCon Plate #20	A5: NFC	-	0.066	0.0641	0.579	99.6
Rxn #55	AnalytiCon Plate #20	B5: NFC	-	0.048	0.0641	0.579	103.1
Rxn #55	AnalytiCon Plate #20	C5: NFC	-	0.054	0.0641	0.579	102.0
Rxn #55	AnalytiCon Plate #20	D5: NFC	-	0.051	0.0641	0.579	102.5
Rxn #55	AnalytiCon Plate #20	E5: FOC	-	0.580	0.0641	0.579	-0.2
Rxn #55	AnalytiCon Plate #20	F5: FOC	-	0.596	0.0641	0.579	-3.3
Rxn #55	AnalytiCon Plate #20	G5: FOC	-	0.588	0.0641	0.579	-1.7
Rxn #55	AnalytiCon Plate #20	H5: FOC	-	0.601	0.0641	0.579	-4.3
Rxn #55	AnalytiCon Plate #20	A6: NFC	-	0.068	0.0641	0.579	99.2
Rxn #55	AnalytiCon Plate #20	B6	50	0.550	0.0641	0.579	5.6
Rxn #55	AnalytiCon Plate #20	C6	50	0.571	0.0641	0.579	1.6
Rxn #55	AnalytiCon Plate #20	D6	50	0.552	0.0641	0.579	5.2
Rxn #55	AnalytiCon Plate #20	E6	50	0.552	0.0641	0.579	5.2
Rxn #55	AnalytiCon Plate #20	F6	50	0.587	0.0641	0.579	-1.6
Rxn #55	AnalytiCon Plate #20	G6	50	0.554	0.0641	0.579	4.9
Rxn #55	AnalytiCon Plate #20	H6: FOC	-	0.593	0.0641	0.579	-2.7
Rxn #55	AnalytiCon Plate #20	A7: NFC	-	0.078	0.0641	0.579	97.3
Rxn #55	AnalytiCon Plate #20	B7	50	0.471	0.0641	0.579	21.0
Rxn #55	AnalytiCon Plate #20	C7	50	0.539	0.0641	0.579	7.8
Rxn #55	AnalytiCon Plate #20	D7	50	0.552	0.0641	0.579	5.2
Rxn #55	AnalytiCon Plate #20	E7	50	0.612	0.0641	0.579	-6.4
Rxn #55	AnalytiCon Plate #20	F7	50	0.521	0.0641	0.579	11.3
Rxn #55	AnalytiCon Plate #20	G7	50	0.512	0.0641	0.579	13.0
Rxn #55	AnalytiCon Plate #20	H7: FOC	-	0.579	0.0641	0.579	0.0
Rxn #55	AnalytiCon Plate #20	A8: NFC	-	0.057	0.0641	0.579	101.4
Rxn #55	AnalytiCon Plate #20	B8	50	0.537	0.0641	0.579	8.2
Rxn #55	AnalytiCon Plate #20	C8	50	0.506	0.0641	0.579	14.2
Rxn #55	AnalytiCon Plate #20	D8	50	0.492	0.0641	0.579	16.9
Rxn #55	AnalytiCon Plate #20	E8	50	0.457	0.0641	0.579	23.7
Rxn #55	AnalytiCon Plate #20	F8	50	0.588	0.0641	0.579	-1.7
Rxn #55	AnalytiCon Plate #20	G8	50	0.552	0.0641	0.579	5.2
Rxn #55	AnalytiCon Plate #20	H8: FOC	-	0.584	0.0641	0.579	-1.0
Rxn #55	AnalytiCon Plate #20	A9: NFC	-	0.059	0.0641	0.579	101.0
Rxn #55	AnalytiCon Plate #20	B9	50	0.495	0.0641	0.579	16.3

S3 Table. Data from Screening of Phytochemical Library for Inhibition of KHKC Activity.

Rxn ID	Sample			Sample	Ave. No Fructose Controls	Ave. Fructose Only Controls	KHKC
	Phytochemical Plate	Well	Conc (μM)	(ΔOD_{340nm})	(ΔOD_{340nm})	(ΔOD_{340nm})	Inhibition (%)
Rxn #55	AnalytiCon Plate #20	C9	50	0.553	0.0641	0.579	5.0
Rxn #55	AnalytiCon Plate #20	D9	50	0.586	0.0641	0.579	-1.4
Rxn #55	AnalytiCon Plate #20	E9	50	0.488	0.0641	0.579	17.7
Rxn #55	AnalytiCon Plate #20	F9	50	0.589	0.0641	0.579	-1.9
Rxn #55	AnalytiCon Plate #20	G9	50	0.551	0.0641	0.579	5.4
Rxn #55	AnalytiCon Plate #20	H9: FOC	-	0.565	0.0641	0.579	2.7
Rxn #55	AnalytiCon Plate #20	A10: NFC	-	0.078	0.0641	0.579	97.3
Rxn #55	AnalytiCon Plate #20	B10	50	0.561	0.0641	0.579	3.5
Rxn #55	AnalytiCon Plate #20	C10	50	0.505	0.0641	0.579	14.4
Rxn #55	AnalytiCon Plate #20	D10	50	0.487	0.0641	0.579	17.9
Rxn #55	AnalytiCon Plate #20	E10	50	0.565	0.0641	0.579	2.7
Rxn #55	AnalytiCon Plate #20	F10	50	0.559	0.0641	0.579	3.9
Rxn #55	AnalytiCon Plate #20	G10	50	0.553	0.0641	0.579	5.0
Rxn #55	AnalytiCon Plate #20	H10: FOC	-	0.595	0.0641	0.579	-3.1
Rxn #55	AnalytiCon Plate #20	A11: NFC	-	0.086	0.0641	0.579	95.7
Rxn #55	AnalytiCon Plate #20	B11	50	0.516	0.0641	0.579	12.2
Rxn #55	AnalytiCon Plate #20	C11	50	0.485	0.0641	0.579	18.3
Rxn #55	AnalytiCon Plate #20	D11	50	0.565	0.0641	0.579	2.7
Rxn #55	AnalytiCon Plate #20	E11	50	0.549	0.0641	0.579	5.8
Rxn #55	AnalytiCon Plate #20	F11	50	0.517	0.0641	0.579	12.0
Rxn #55	AnalytiCon Plate #20	G11	50	0.552	0.0641	0.579	5.2
Rxn #55	AnalytiCon Plate #20	H11: FOC	-	0.553	0.0641	0.579	5.0
Rxn #55	AnalytiCon Plate #20	A12: NFC	-	0.059	0.0641	0.579	101.0
Rxn #55	AnalytiCon Plate #20	H12: FOC	-	0.537	0.0641	0.579	8.2

AnalytiCon: Phytochemicals from MEGx library (AnalytiCon Discovery GmbH, Potsdam, Germany) were selected and purchased by Amway Corp.
KHKC: ketohexokinase isoform C. FOC: fructose only control. NFC: no fructose control. OD: optical density.
