# Supplementary material for: Bioactivity-Guided Identification of Botanical Inhibitors of Ketohexokinase
Source: PLoS One. 2016 Jun 20;11(6):e0157458. doi: 10.1371/journal.pone.0157458 (PMC4913896; doi:10.1371/journal.pone.0157458)
Supplement: S4 Table — AnalytiCon: Phytochemicals from MEGx library (AnalytiCon Discovery GmbH, Potsdam, Germany) were selected and purchased by Amway Corp. IC50: half maximal inhibitory concentration. OD: optical density. *KHKC IC50s were calculated using nonlinear regression (three parameters) in GraphPad Prism 5.03. To generate a best fit, an upper concentration (10,000 μg/mL at 100% inhibition) and a lower concentration (0.001 μg/mL at 0% inhibition) were added. (PDF) [file pone.0157458.s004.pdf]

S4 Table. Data from Titrations of Candidate Phytochemicals for Inhibition of KHKC Activity.

Rxn ID	Rxn Well	Sample Phytochemical Plate	Sample Well	Sample Conc (μM)	Sample (ΔOD_{340nm})	Ave. No Fructose Controls (ΔOD_{340nm})	Ave. Fructose Only Controls (ΔOD_{340nm})	KHKC Inhibition (%)	*KHKC IC_{50} (μM)
Rxn #44	A1	AnalytiCon Plate #1	D10	106.58	0.284	0.118	0.682	70.6	3.48
Rxn #44	B1	AnalytiCon Plate #1	D10	79.93	0.312	0.118	0.682	65.6	
Rxn #44	C1	AnalytiCon Plate #1	D10	53.29	0.252	0.118	0.682	76.2	
Rxn #44	D1	AnalytiCon Plate #1	D10	26.64	0.262	0.118	0.682	74.5	
Rxn #44	E1	AnalytiCon Plate #1	D10	13.32	0.294	0.118	0.682	68.8	
Rxn #44	F1	AnalytiCon Plate #1	D10	5.33	0.379	0.118	0.682	53.7	
Rxn #44	G1	AnalytiCon Plate #1	D10	2.66	0.514	0.118	0.682	29.8	
Rxn #44	H1	AnalytiCon Plate #1	D10	0.05	0.707	0.118	0.682	-4.4	
Rxn #44	A2	AnalytiCon Plate #1	G7	110.48	0.249	0.118	0.682	76.8	10.33
Rxn #44	B2	AnalytiCon Plate #1	G7	82.86	0.225	0.118	0.682	81.0	
Rxn #44	C2	AnalytiCon Plate #1	G7	55.24	0.230	0.118	0.682	80.1	
Rxn #44	D2	AnalytiCon Plate #1	G7	27.62	0.302	0.118	0.682	67.4	
Rxn #44	E2	AnalytiCon Plate #1	G7	13.81	0.354	0.118	0.682	58.2	
Rxn #44	F2	AnalytiCon Plate #1	G7	5.52	0.530	0.118	0.682	27.0	
Rxn #44	G2	AnalytiCon Plate #1	G7	2.76	0.586	0.118	0.682	17.0	
Rxn #44	H2	AnalytiCon Plate #1	G7	0.06	0.715	0.118	0.682	-5.9	
Rxn #44	A3	AnalytiCon Plate #2	F8	71.7	0.288	0.118	0.682	69.9	29.45
Rxn #44	B3	AnalytiCon Plate #2	F8	53.78	0.320	0.118	0.682	64.2	
Rxn #44	C3	AnalytiCon Plate #2	F8	35.85	0.383	0.118	0.682	53.0	
Rxn #44	D3	AnalytiCon Plate #2	F8	17.93	0.474	0.118	0.682	36.9	
Rxn #44	E3	AnalytiCon Plate #2	F8	8.96	0.540	0.118	0.682	25.2	
Rxn #44	F3	AnalytiCon Plate #2	F8	3.59	0.631	0.118	0.682	9.0	
Rxn #44	G3	AnalytiCon Plate #2	F8	1.79	0.626	0.118	0.682	9.9	
Rxn #44	H3	AnalytiCon Plate #2	F8	0.04	0.712	0.118	0.682	-5.3	
Rxn #44	A4	AnalytiCon Plate #2	B8	83.7	0.223	0.118	0.682	81.4	11.29
Rxn #44	B4	AnalytiCon Plate #2	B8	62.78	0.222	0.118	0.682	81.6	
Rxn #44	C4	AnalytiCon Plate #2	B8	41.85	0.243	0.118	0.682	77.8	
Rxn #44	D4	AnalytiCon Plate #2	B8	20.93	0.354	0.118	0.682	58.2	
Rxn #44	E4	AnalytiCon Plate #2	B8	10.46	0.425	0.118	0.682	45.6	
Rxn #44	F4	AnalytiCon Plate #2	B8	4.19	0.506	0.118	0.682	31.2	
Rxn #44	G4	AnalytiCon Plate #2	B8	2.09	0.579	0.118	0.682	18.3	
Rxn #44	H4	AnalytiCon Plate #2	B8	0.04	0.701	0.118	0.682	-3.4	
Rxn #44	A5	AnalytiCon Plate #2	C8	73.05	0.454	0.118	0.682	40.4	1.00
Rxn #44	B5	AnalytiCon Plate #2	C8	54.79	0.348	0.118	0.682	59.2	
Rxn #44	C5	AnalytiCon Plate #2	C8	36.53	0.387	0.118	0.682	52.3	
Rxn #44	D5	AnalytiCon Plate #2	C8	18.26	0.349	0.118	0.682	59.0	
Rxn #44	E5	AnalytiCon Plate #2	C8	9.13	0.349	0.118	0.682	59.0	
Rxn #44	F5	AnalytiCon Plate #2	C8	3.65	0.392	0.118	0.682	51.4	
Rxn #44	G5	AnalytiCon Plate #2	C8	1.83	0.439	0.118	0.682	43.1	
Rxn #44	H5	AnalytiCon Plate #2	C8	0.04	0.700	0.118	0.682	-3.2	
Rxn #44	A6	AnalytiCon Plate #2	G10	122.8	0.286	0.118	0.682	70.2	0.65
Rxn #44	B6	AnalytiCon Plate #2	G10	92.1	0.252	0.118	0.682	76.2	
Rxn #44	C6	AnalytiCon Plate #2	G10	61.4	0.223	0.118	0.682	81.4	
Rxn #44	D6	AnalytiCon Plate #2	G10	30.7	0.170	0.118	0.682	90.8	
Rxn #44	E6	AnalytiCon Plate #2	G10	15.35	0.173	0.118	0.682	90.2	
Rxn #44	F6	AnalytiCon Plate #2	G10	6.14	0.221	0.118	0.682	81.7	
Rxn #44	G6	AnalytiCon Plate #2	G10	3.07	0.299	0.118	0.682	67.9	
Rxn #44	H6	AnalytiCon Plate #2	G10	0.06	0.665	0.118	0.682	3.0	
Rxn #44	A7	AnalytiCon Plate #10	B11	92.45	0.290	0.118	0.682	69.5	5.70
Rxn #44	B7	AnalytiCon Plate #10	B11	69.34	0.261	0.118	0.682	74.6	
Rxn #44	C7	AnalytiCon Plate #10	B11	46.23	0.239	0.118	0.682	78.5	
Rxn #44	D7	AnalytiCon Plate #10	B11	23.11	0.271	0.118	0.682	72.9	
Rxn #44	E7	AnalytiCon Plate #10	B11	11.56	0.356	0.118	0.682	57.8	
Rxn #44	F7	AnalytiCon Plate #10	B11	4.62	0.458	0.118	0.682	39.7	
Rxn #44	G7	AnalytiCon Plate #10	B11	2.31	0.562	0.118	0.682	21.3	
Rxn #44	H7	AnalytiCon Plate #10	B11	0.05	0.721	0.118	0.682	-6.9	
Rxn #44	A10	No Fructose Control			0.118	0.118	0.682	100.0	
Rxn #44	B10	No Fructose Control			0.111	0.118	0.682	101.2	
Rxn #44	C10	No Fructose Control			0.130	0.118	0.682	97.9	
Rxn #44	D10	No Fructose Control			0.106	0.118	0.682	102.1	
Rxn #44	E10	No Fructose Control			0.109	0.118	0.682	101.6	
Rxn #44	F10	No Fructose Control			0.135	0.118	0.682	97.0	
Rxn #44	G10	No Fructose Control			0.122	0.118	0.682	99.3	
Rxn #44	H10	No Fructose Control			0.112	0.118	0.682	101.1	
Rxn #44	A11	Fructose Only Control			0.690	0.118	0.682	-1.4	
Rxn #44	B11	Fructose Only Control			0.678	0.118	0.682	0.7	
Rxn #44	C11	Fructose Only Control			0.669	0.118	0.682	2.3	
Rxn #44	D11	Fructose Only Control			0.691	0.118	0.682	-1.6	
Rxn #44	E11	Fructose Only Control			0.675	0.118	0.682	1.2	
Rxn #44	F11	Fructose Only Control			0.698	0.118	0.682	-2.8	
Rxn #44	G11	Fructose Only Control			0.693	0.118	0.682	-2.0	
Rxn #44	H11	Fructose Only Control			0.662	0.118	0.682	3.5	
Rxn #45	A1	AnalytiCon Plate #3	E1	105.5	0.337	0.111	0.628	56.3	8.21
Rxn #45	B1	AnalytiCon Plate #3	E1	79.13	0.325	0.111	0.628	58.6	
Rxn #45	C1	AnalytiCon Plate #3	E1	52.75	0.272	0.111	0.628	68.9	
Rxn #45	D1	AnalytiCon Plate #3	E1	26.38	0.296	0.111	0.628	64.2	
Rxn #45	E1	AnalytiCon Plate #3	E1	13.19	0.341	0.111	0.628	55.5	
Rxn #45	F1	AnalytiCon Plate #3	E1	5.28	0.448	0.111	0.628	34.8	
Rxn #45	G1	AnalytiCon Plate #3	E1	2.64	0.511	0.111	0.628	22.6	
Rxn #45	H1	AnalytiCon Plate #3	E1	0.05	0.589	0.111	0.628	7.5	
Rxn #45	A2	AnalytiCon Plate #3	D7	71	0.289	0.111	0.628	65.6	31.24

S4 Table. Data from Titrations of Candidate Phytochemicals for Inhibition of KHKC Activity.

Rxn		Sample			Sample	Ave. No Fructose Controls	Ave. Fructose Only Controls	KHKC	*KHKC IC_{50}
ID	Well	Phytochemical Plate	Well	Conc (μM)	(ΔOD_{340nm})	(ΔOD_{340nm})	(ΔOD_{340nm})	Inhibition (%)	(μM)
Rxn #45	B2	AnalytiCon Plate #3	D7	53.25	0.327	0.111	0.628	58.2	
Rxn #45	C2	AnalytiCon Plate #3	D7	35.5	0.349	0.111	0.628	54.0	
Rxn #45	D2	AnalytiCon Plate #3	D7	17.75	0.430	0.111	0.628	38.3	
Rxn #45	E2	AnalytiCon Plate #3	D7	8.88	0.429	0.111	0.628	38.5	
Rxn #45	F2	AnalytiCon Plate #3	D7	3.55	0.523	0.111	0.628	20.3	
Rxn #45	G2	AnalytiCon Plate #3	D7	1.78	0.568	0.111	0.628	11.6	
Rxn #45	H2	AnalytiCon Plate #3	D7	0.04	0.602	0.111	0.628	5.0	
Rxn #45	A3	AnalytiCon Plate #4	F3	67.2	0.312	0.111	0.628	61.1	16.13
Rxn #45	B3	AnalytiCon Plate #4	F3	50.4	0.307	0.111	0.628	62.1	
Rxn #45	C3	AnalytiCon Plate #4	F3	33.6	0.290	0.111	0.628	65.4	
Rxn #45	D3	AnalytiCon Plate #4	F3	16.8	0.378	0.111	0.628	48.4	
Rxn #45	E3	AnalytiCon Plate #4	F3	8.4	0.424	0.111	0.628	39.5	
Rxn #45	F3	AnalytiCon Plate #4	F3	3.36	0.529	0.111	0.628	19.1	
Rxn #45	G3	AnalytiCon Plate #4	F3	1.68	0.581	0.111	0.628	9.1	
Rxn #45	H3	AnalytiCon Plate #4	F3	0.03	0.641	0.111	0.628	-2.5	
Rxn #45	A4	AnalytiCon Plate #4	C9	84.65	0.355	0.111	0.628	52.8	0.19
Rxn #45	B4	AnalytiCon Plate #4	C9	63.49	0.354	0.111	0.628	53.0	
Rxn #45	C4	AnalytiCon Plate #4	C9	42.33	0.334	0.111	0.628	56.9	
Rxn #45	D4	AnalytiCon Plate #4	C9	21.16	0.291	0.111	0.628	65.2	
Rxn #45	E4	AnalytiCon Plate #4	C9	10.58	0.206	0.111	0.628	81.6	
Rxn #45	F4	AnalytiCon Plate #4	C9	4.23	0.204	0.111	0.628	82.0	
Rxn #45	G4	AnalytiCon Plate #4	C9	2.12	0.267	0.111	0.628	69.8	
Rxn #45	H4	AnalytiCon Plate #4	C9	0.04	0.616	0.111	0.628	2.3	
Rxn #45	A5	AnalytiCon Plate #4	F2	75.65	0.268	0.111	0.628	69.6	1.49
Rxn #45	B5	AnalytiCon Plate #4	F2	56.74	0.203	0.111	0.628	82.2	
Rxn #45	C5	AnalytiCon Plate #4	F2	37.83	0.211	0.111	0.628	80.7	
Rxn #45	D5	AnalytiCon Plate #4	F2	18.91	0.225	0.111	0.628	77.9	
Rxn #45	E5	AnalytiCon Plate #4	F2	9.46	0.212	0.111	0.628	80.5	
Rxn #45	F5	AnalytiCon Plate #4	F2	3.78	0.309	0.111	0.628	61.7	
Rxn #45	G5	AnalytiCon Plate #4	F2	1.89	0.395	0.111	0.628	45.1	
Rxn #45	H5	AnalytiCon Plate #4	F2	0.04	0.651	0.111	0.628	-4.4	
Rxn #45	A6	AnalytiCon Plate #5	G10	130.25	0.196	0.111	0.628	83.6	7.77
Rxn #45	B6	AnalytiCon Plate #5	G10	97.69	0.188	0.111	0.628	85.1	
Rxn #45	C6	AnalytiCon Plate #5	G10	65.13	0.189	0.111	0.628	84.9	
Rxn #45	D6	AnalytiCon Plate #5	G10	32.56	0.230	0.111	0.628	77.0	
Rxn #45	E6	AnalytiCon Plate #5	G10	16.28	0.276	0.111	0.628	68.1	
Rxn #45	F6	AnalytiCon Plate #5	G10	6.51	0.413	0.111	0.628	41.6	
Rxn #45	G6	AnalytiCon Plate #5	G10	3.26	0.487	0.111	0.628	27.3	
Rxn #45	H6	AnalytiCon Plate #5	G10	0.07	0.623	0.111	0.628	1.0	
Rxn #45	A7	AnalytiCon Plate #6	E1	53.15	0.322	0.111	0.628	59.2	21.52
Rxn #45	B7	AnalytiCon Plate #6	E1	39.86	0.317	0.111	0.628	60.2	
Rxn #45	C7	AnalytiCon Plate #6	E1	26.58	0.341	0.111	0.628	55.5	
Rxn #45	D7	AnalytiCon Plate #6	E1	13.29	0.406	0.111	0.628	42.9	
Rxn #45	E7	AnalytiCon Plate #6	E1	6.64	0.454	0.111	0.628	33.7	
Rxn #45	F7	AnalytiCon Plate #6	E1	2.66	0.567	0.111	0.628	11.8	
Rxn #45	G7	AnalytiCon Plate #6	E1	1.33	0.624	0.111	0.628	0.8	
Rxn #45	H7	AnalytiCon Plate #6	E1	0.03	0.595	0.111	0.628	6.4	
Rxn #45	A10	No Fructose Control			0.142	0.111	0.628	94.0	
Rxn #45	B10	No Fructose Control			0.109	0.111	0.628	100.4	
Rxn #45	C10	No Fructose Control			0.105	0.111	0.628	101.2	
Rxn #45	D10	No Fructose Control			0.105	0.111	0.628	101.2	
Rxn #45	E10	No Fructose Control			0.090	0.111	0.628	104.1	
Rxn #45	F10	No Fructose Control			0.107	0.111	0.628	100.8	
Rxn #45	G10	No Fructose Control			0.101	0.111	0.628	101.9	
Rxn #45	H10	No Fructose Control			0.128	0.111	0.628	96.7	
Rxn #45	A11	Fructose Only Control			0.645	0.111	0.628	-3.3	
Rxn #45	B11	Fructose Only Control			0.622	0.111	0.628	1.2	
Rxn #45	C11	Fructose Only Control			0.636	0.111	0.628	-1.5	
Rxn #45	D11	Fructose Only Control			0.627	0.111	0.628	0.2	
Rxn #45	E11	Fructose Only Control			0.598	0.111	0.628	5.8	
Rxn #45	F11	Fructose Only Control			0.631	0.111	0.628	-0.6	
Rxn #45	G11	Fructose Only Control			0.634	0.111	0.628	-1.2	
Rxn #45	H11	Fructose Only Control			0.634	0.111	0.628	-1.2	
Rxn #56	A1	AnalytiCon Plate #13	G6	123.8	0.230	0.0795	0.609	71.6	58.36
Rxn #56	B1	AnalytiCon Plate #13	G6	92.85	0.271	0.0795	0.609	63.8	
Rxn #56	C1	AnalytiCon Plate #13	G6	61.9	0.353	0.0795	0.609	48.3	
Rxn #56	D1	AnalytiCon Plate #13	G6	30.95	0.406	0.0795	0.609	38.3	
Rxn #56	E1	AnalytiCon Plate #13	G6	15.48	0.470	0.0795	0.609	26.3	
Rxn #56	F1	AnalytiCon Plate #13	G6	6.19	0.524	0.0795	0.609	16.1	
Rxn #56	G1	AnalytiCon Plate #13	G6	3.1	0.565	0.0795	0.609	8.3	
Rxn #56	H1	AnalytiCon Plate #13	G6	0.06	0.580	0.0795	0.609	5.5	
Rxn #56	A2	No Fructose Control			0.086	0.0795	0.609	98.8	
Rxn #56	B2	No Fructose Control			0.077	0.0795	0.609	100.5	
Rxn #56	C2	No Fructose Control			0.081	0.0795	0.609	99.7	
Rxn #56	D2	No Fructose Control			0.076	0.0795	0.609	100.7	
Rxn #56	E2	No Fructose Control			0.080	0.0795	0.609	99.9	
Rxn #56	F2	No Fructose Control			0.078	0.0795	0.609	100.3	
Rxn #56	G2	No Fructose Control			0.076	0.0795	0.609	100.7	
Rxn #56	H2	No Fructose Control			0.082	0.0795	0.609	99.5	
Rxn #56	A10	Fructose Only Control			0.618	0.0795	0.609	-1.7	
Rxn #56	B10	Fructose Only Control			0.611	0.0795	0.609	-0.4	

S4 Table. Data from Titrations of Candidate Phytochemicals for Inhibition of KHKC Activity.

Rxn		Sample			Sample	Ave. No Fructose Controls	Ave. Fructose Only Controls	KHKC	*KHKC IC_{50}
ID	Well	Phytochemical Plate	Well	Conc (μM)	(ΔOD_{340nm})	(ΔOD_{340nm})	(ΔOD_{340nm})	Inhibition (%)	(μM)
Rxn #56	C10	Fructose Only Control			0.610	0.0795	0.609	-0.2	
Rxn #56	D10	Fructose Only Control			0.569	0.0795	0.609	7.6	
Rxn #56	E10	Fructose Only Control			0.611	0.0795	0.609	-0.4	
Rxn #56	F10	Fructose Only Control			0.612	0.0795	0.609	-0.6	
Rxn #56	G10	Fructose Only Control			0.618	0.0795	0.609	-1.7	
Rxn #56	H10	Fructose Only Control			0.621	0.0795	0.609	-2.3	

AnalytiCon: Phytochemicals from MEGx library (AnalytiCon Discovery GmbH, Potsdam, Germany) were selected and purchased by Amway Corp.

IC_{50}: half maximal inhibitory concentration. OD: optical density.

*KHKC IC_{50}s were calculated using nonlinear regression (three parameters) in GraphPad Prism 5.03. To generate a best fit, an upper concentration (10,000 μg/mL at 100% inhibition) and a lower concentration (0.001 μg/mL at 0% inhibition) were added.
