# Supplementary material for: Bioactivity-Guided Identification of Botanical Inhibitors of Ketohexokinase
Source: PLoS One. 2016 Jun 20;11(6):e0157458. doi: 10.1371/journal.pone.0157458 (PMC4913896; doi:10.1371/journal.pone.0157458)
Supplement: S5 Table — IC50: half maximal inhibitory concentration. OD: optical density. *KHKC IC50s were calculated using nonlinear regression (three parameters) in GraphPad Prism 5.03. To generate a best fit, an upper concentration (10,000 μg/mL at 100% inhibition) and a lower concentration (0.001 μg/mL at 0% inhibition) were added. (PDF) [file pone.0157458.s005.pdf]

S5 Table. Data from Titrations of Top Botanical Candidates for Inhibition of KHKC Activity.

Rxn		Botanical Extract					Sample	Ave. No Fructose Controls	Ave. Fructose Only Controls	KHKC	*KHKC IC_{50}
ID	Well	Genus	Species	SampleID	Lot #	Conc (μg/mL)	(ΔOD_{340nm})	(ΔOD_{340nm})	(ΔOD_{340nm})	Inhibition (%)	(μg/mL)
Rxn #65	A1	*Angelica*	*archangelica*	1	1	200	0.242	0.0665	0.532	62.3	90.38
Rxn #65	B1	*Angelica*	*archangelica*	1	1	100	0.301	0.0665	0.532	49.6	
Rxn #65	C1	*Angelica*	*archangelica*	1	1	75	0.342	0.0665	0.532	40.8	
Rxn #65	D1	*Angelica*	*archangelica*	1	1	50	0.393	0.0665	0.532	29.9	
Rxn #65	E1	*Angelica*	*archangelica*	1	1	25	0.456	0.0665	0.532	16.3	
Rxn #65	F1	*Angelica*	*archangelica*	1	1	10	0.519	0.0665	0.532	2.8	
Rxn #65	G1	*Angelica*	*archangelica*	1	1	1	0.588	0.0665	0.532	-12.0	
Rxn #65	H1	*Angelica*	*archangelica*	1	1	0.1	0.603	0.0665	0.532	-15.3	
Rxn #65	A2	*Angelica*	*archangelica*	1	1	200	0.231	0.0665	0.532	64.7	95.55
Rxn #65	B2	*Angelica*	*archangelica*	1	1	100	0.297	0.0665	0.532	50.5	
Rxn #65	C2	*Angelica*	*archangelica*	1	1	75	0.350	0.0665	0.532	39.1	
Rxn #65	D2	*Angelica*	*archangelica*	1	1	50	0.396	0.0665	0.532	29.2	
Rxn #65	E2	*Angelica*	*archangelica*	1	1	25	0.475	0.0665	0.532	12.2	
Rxn #65	F2	*Angelica*	*archangelica*	1	1	10	0.533	0.0665	0.532	-0.2	
Rxn #65	G2	*Angelica*	*archangelica*	1	1	1	0.577	0.0665	0.532	-9.7	
Rxn #65	H2	*Angelica*	*archangelica*	1	1	0.1	0.595	0.0665	0.532	-13.5	
Rxn #65	A3	*Angelica*	*archangelica*	1	1	200	0.242	0.0665	0.532	62.3	98.60
Rxn #65	B3	*Angelica*	*archangelica*	1	1	100	0.304	0.0665	0.532	49.0	
Rxn #65	C3	*Angelica*	*archangelica*	1	1	75	0.353	0.0665	0.532	38.5	
Rxn #65	D3	*Angelica*	*archangelica*	1	1	50	0.403	0.0665	0.532	27.7	
Rxn #65	E3	*Angelica*	*archangelica*	1	1	25	0.486	0.0665	0.532	9.9	
Rxn #65	F3	*Angelica*	*archangelica*	1	1	10	0.535	0.0665	0.532	-0.6	
Rxn #65	G3	*Angelica*	*archangelica*	1	1	1	0.593	0.0665	0.532	-13.1	
Rxn #65	H3	*Angelica*	*archangelica*	1	1	0.1	0.598	0.0665	0.532	-14.2	
Rxn #65	A10	No Fructose Control					0.062	0.0665	0.532	101.0	
Rxn #65	B10	No Fructose Control					0.072	0.0665	0.532	98.8	
Rxn #65	C10	No Fructose Control					0.070	0.0665	0.532	99.2	
Rxn #65	D10	No Fructose Control					0.067	0.0665	0.532	99.9	
Rxn #65	E10	No Fructose Control					0.068	0.0665	0.532	99.7	
Rxn #65	F10	No Fructose Control					0.067	0.0665	0.532	99.9	
Rxn #65	G10	No Fructose Control					0.072	0.0665	0.532	98.8	
Rxn #65	H10	No Fructose Control					0.054	0.0665	0.532	102.7	
Rxn #65	A11	Fructose Only Control					0.476	0.0665	0.532	12.0	
Rxn #65	B11	Fructose Only Control					0.486	0.0665	0.532	9.9	
Rxn #65	C11	Fructose Only Control					0.537	0.0665	0.532	-1.1	
Rxn #65	D11	Fructose Only Control					0.562	0.0665	0.532	-6.4	
Rxn #65	E11	Fructose Only Control					0.568	0.0665	0.532	-7.7	
Rxn #65	F11	Fructose Only Control					0.571	0.0665	0.532	-8.4	
Rxn #65	G11	Fructose Only Control					0.546	0.0665	0.532	-3.0	
Rxn #65	H11	Fructose Only Control					0.511	0.0665	0.532	4.5	
Rxn #66	A1	*Scutellaria*	*baicalensis*	4	1	200	0.254	0.0699	0.463	53.2	117.00
Rxn #66	B1	*Scutellaria*	*baicalensis*	4	1	100	0.292	0.0699	0.463	43.5	
Rxn #66	C1	*Scutellaria*	*baicalensis*	4	1	75	0.312	0.0699	0.463	38.4	
Rxn #66	D1	*Scutellaria*	*baicalensis*	4	1	50	0.337	0.0699	0.463	32.1	
Rxn #66	E1	*Scutellaria*	*baicalensis*	4	1	25	0.386	0.0699	0.463	19.6	
Rxn #66	F1	*Scutellaria*	*baicalensis*	4	1	10	0.430	0.0699	0.463	8.4	
Rxn #66	G1	*Scutellaria*	*baicalensis*	4	1	1	0.479	0.0699	0.463	-4.1	
Rxn #66	H1	*Scutellaria*	*baicalensis*	4	1	0.1	0.497	0.0699	0.463	-8.6	
Rxn #66	A2	*Scutellaria*	*baicalensis*	4	1	200	0.248	0.0699	0.463	54.7	120.60
Rxn #66	B2	*Scutellaria*	*baicalensis*	4	1	100	0.280	0.0699	0.463	46.6	
Rxn #66	C2	*Scutellaria*	*baicalensis*	4	1	75	0.315	0.0699	0.463	37.6	
Rxn #66	D2	*Scutellaria*	*baicalensis*	4	1	50	0.327	0.0699	0.463	34.6	
Rxn #66	E2	*Scutellaria*	*baicalensis*	4	1	25	0.375	0.0699	0.463	22.4	
Rxn #66	F2	*Scutellaria*	*baicalensis*	4	1	10	0.417	0.0699	0.463	11.7	
Rxn #66	G2	*Scutellaria*	*baicalensis*	4	1	1	0.453	0.0699	0.463	2.5	
Rxn #66	H2	*Scutellaria*	*baicalensis*	4	1	0.1	0.477	0.0699	0.463	-3.6	
Rxn #66	A3	*Scutellaria*	*baicalensis*	4	1	200	0.258	0.0699	0.463	52.1	126.20
Rxn #66	B3	*Scutellaria*	*baicalensis*	4	1	100	0.287	0.0699	0.463	44.8	
Rxn #66	C3	*Scutellaria*	*baicalensis*	4	1	75	0.305	0.0699	0.463	40.2	
Rxn #66	D3	*Scutellaria*	*baicalensis*	4	1	50	0.341	0.0699	0.463	31.0	
Rxn #66	E3	*Scutellaria*	*baicalensis*	4	1	25	0.374	0.0699	0.463	22.6	
Rxn #66	F3	*Scutellaria*	*baicalensis*	4	1	10	0.432	0.0699	0.463	7.9	
Rxn #66	G3	*Scutellaria*	*baicalensis*	4	1	1	0.454	0.0699	0.463	2.3	
Rxn #66	H3	*Scutellaria*	*baicalensis*	4	1	0.1	0.477	0.0699	0.463	-3.6	
Rxn #66	A4	*Petroselinum*	*crispum*	6	1	200	0.264	0.0699	0.463	50.6	185.50
Rxn #66	B4	*Petroselinum*	*crispum*	6	1	100	0.320	0.0699	0.463	36.4	
Rxn #66	C4	*Petroselinum*	*crispum*	6	1	75	0.350	0.0699	0.463	28.7	
Rxn #66	D4	*Petroselinum*	*crispum*	6	1	50	0.386	0.0699	0.463	19.6	
Rxn #66	E4	*Petroselinum*	*crispum*	6	1	25	0.403	0.0699	0.463	15.3	
Rxn #66	F4	*Petroselinum*	*crispum*	6	1	10	0.455	0.0699	0.463	2.0	
Rxn #66	G4	*Petroselinum*	*crispum*	6	1	1	0.460	0.0699	0.463	0.8	
Rxn #66	H4	*Petroselinum*	*crispum*	6	1	0.1	0.475	0.0699	0.463	-3.1	
Rxn #66	A5	*Petroselinum*	*crispum*	6	1	200	0.257	0.0699	0.463	52.4	190.10
Rxn #66	B5	*Petroselinum*	*crispum*	6	1	100	0.336	0.0699	0.463	32.3	
Rxn #66	C5	*Petroselinum*	*crispum*	6	1	75	0.342	0.0699	0.463	30.8	
Rxn #66	D5	*Petroselinum*	*crispum*	6	1	50	0.375	0.0699	0.463	22.4	
Rxn #66	E5	*Petroselinum*	*crispum*	6	1	25	0.408	0.0699	0.463	14.0	
Rxn #66	F5	*Petroselinum*	*crispum*	6	1	10	0.443	0.0699	0.463	5.1	
Rxn #66	G5	*Petroselinum*	*crispum*	6	1	1	0.451	0.0699	0.463	3.1	
Rxn #66	H5	*Petroselinum*	*crispum*	6	1	0.1	0.473	0.0699	0.463	-2.5	
Rxn #66	A6	*Petroselinum*	*crispum*	6	1	200	0.253	0.0699	0.463	53.4	180.70
Rxn #66	B6	*Petroselinum*	*crispum*	6	1	100	0.334	0.0699	0.463	32.8	
Rxn #66	C6	*Petroselinum*	*crispum*	6	1	75	0.343	0.0699	0.463	30.5	
Rxn #66	D6	*Petroselinum*	*crispum*	6	1	50	0.375	0.0699	0.463	22.4	

S5 Table. Data from Titrations of Top Botanical Candidates for Inhibition of KHKC Activity.

Rxn		Botanical Extract					Sample	Ave. No Fructose Controls	Ave. Fructose Only Controls	KHKC	*KHKC IC_{50}
ID	Well	Genus	Species	SampleID	Lot #	Conc (µg/mL)	(ΔOD_{340nm})	(ΔOD_{340nm})	(ΔOD_{340nm})	Inhibition (%)	(µg/mL)
Rxn #66	E6	*Petroselinum*	*crispum*	6	1	25	0.416	0.0699	0.463	12.0	
Rxn #66	F6	*Petroselinum*	*crispum*	6	1	10	0.442	0.0699	0.463	5.3	
Rxn #66	G6	*Petroselinum*	*crispum*	6	1	1	0.464	0.0699	0.463	-0.3	
Rxn #66	H6	*Petroselinum*	*crispum*	6	1	0.1	0.476	0.0699	0.463	-3.3	
Rxn #66	A10	No Fructose Control					0.076	0.0699	0.463	98.4	
Rxn #66	B10	No Fructose Control					0.067	0.0699	0.463	100.7	
Rxn #66	C10	No Fructose Control					0.069	0.0699	0.463	100.2	
Rxn #66	D10	No Fructose Control					0.070	0.0699	0.463	100.0	
Rxn #66	E10	No Fructose Control					0.059	0.0699	0.463	102.8	
Rxn #66	F10	No Fructose Control					0.070	0.0699	0.463	100.0	
Rxn #66	G10	No Fructose Control					0.072	0.0699	0.463	99.5	
Rxn #66	H10	No Fructose Control					0.076	0.0699	0.463	98.4	
Rxn #66	A11	Fructose Only Control					0.459	0.0699	0.463	1.0	
Rxn #66	B11	Fructose Only Control					0.468	0.0699	0.463	-1.3	
Rxn #66	C11	Fructose Only Control					0.458	0.0699	0.463	1.3	
Rxn #66	D11	Fructose Only Control					0.456	0.0699	0.463	1.8	
Rxn #66	E11	Fructose Only Control					0.458	0.0699	0.463	1.3	
Rxn #66	F11	Fructose Only Control					0.462	0.0699	0.463	0.3	
Rxn #66	G11	Fructose Only Control					0.465	0.0699	0.463	-0.5	
Rxn #66	H11	Fructose Only Control					0.474	0.0699	0.463	-2.8	
Rxn #67	A1	*Garcinia*	*mangostana*	8	1	200	0.168	0.0699	0.525	78.4	18.55
Rxn #67	B1	*Garcinia*	*mangostana*	8	1	100	0.170	0.0699	0.525	78.0	
Rxn #67	C1	*Garcinia*	*mangostana*	8	1	75	0.175	0.0699	0.525	76.9	
Rxn #67	D1	*Garcinia*	*mangostana*	8	1	50	0.200	0.0699	0.525	71.4	
Rxn #67	E1	*Garcinia*	*mangostana*	8	1	25	0.277	0.0699	0.525	54.5	
Rxn #67	F1	*Garcinia*	*mangostana*	8	1	10	0.414	0.0699	0.525	24.4	
Rxn #67	G1	*Garcinia*	*mangostana*	8	1	1	0.554	0.0699	0.525	-6.4	
Rxn #67	H1	*Garcinia*	*mangostana*	8	1	0.1	0.586	0.0699	0.525	-13.4	
Rxn #67	A2	*Garcinia*	*mangostana*	8	1	200	0.163	0.0699	0.525	79.5	18.27
Rxn #67	B2	*Garcinia*	*mangostana*	8	1	100	0.155	0.0699	0.525	81.3	
Rxn #67	C2	*Garcinia*	*mangostana*	8	1	75	0.182	0.0699	0.525	75.4	
Rxn #67	D2	*Garcinia*	*mangostana*	8	1	50	0.195	0.0699	0.525	72.5	
Rxn #67	E2	*Garcinia*	*mangostana*	8	1	25	0.270	0.0699	0.525	56.0	
Rxn #67	F2	*Garcinia*	*mangostana*	8	1	10	0.406	0.0699	0.525	26.1	
Rxn #67	G2	*Garcinia*	*mangostana*	8	1	1	0.550	0.0699	0.525	-5.5	
Rxn #67	H2	*Garcinia*	*mangostana*	8	1	0.1	0.569	0.0699	0.525	-9.7	
Rxn #67	A3	*Garcinia*	*mangostana*	8	1	200	0.146	0.0699	0.525	83.3	19.38
Rxn #67	B3	*Garcinia*	*mangostana*	8	1	100	0.162	0.0699	0.525	79.8	
Rxn #67	C3	*Garcinia*	*mangostana*	8	1	75	0.177	0.0699	0.525	76.5	
Rxn #67	D3	*Garcinia*	*mangostana*	8	1	50	0.203	0.0699	0.525	70.8	
Rxn #67	E3	*Garcinia*	*mangostana*	8	1	25	0.275	0.0699	0.525	54.9	
Rxn #67	F3	*Garcinia*	*mangostana*	8	1	10	0.413	0.0699	0.525	24.6	
Rxn #67	G3	*Garcinia*	*mangostana*	8	1	1	0.558	0.0699	0.525	-7.3	
Rxn #67	H3	*Garcinia*	*mangostana*	8	1	0.1	0.578	0.0699	0.525	-11.6	
Rxn #67	A10	No Fructose Control					0.075	0.0699	0.525	98.9	
Rxn #67	B10	No Fructose Control					0.070	0.0699	0.525	100.0	
Rxn #67	C10	No Fructose Control					0.069	0.0699	0.525	100.2	
Rxn #67	D10	No Fructose Control					0.066	0.0699	0.525	100.9	
Rxn #67	E10	No Fructose Control					0.071	0.0699	0.525	99.8	
Rxn #67	F10	No Fructose Control					0.068	0.0699	0.525	100.4	
Rxn #67	G10	No Fructose Control					0.068	0.0699	0.525	100.4	
Rxn #67	H10	No Fructose Control					0.072	0.0699	0.525	99.5	
Rxn #67	A11	Fructose Only Control					0.479	0.0699	0.525	10.1	
Rxn #67	B11	Fructose Only Control					0.495	0.0699	0.525	6.6	
Rxn #67	C11	Fructose Only Control					0.521	0.0699	0.525	0.9	
Rxn #67	D11	Fructose Only Control					0.531	0.0699	0.525	-1.3	
Rxn #67	E11	Fructose Only Control					0.537	0.0699	0.525	-2.6	
Rxn #67	F11	Fructose Only Control					0.537	0.0699	0.525	-2.6	
Rxn #67	G11	Fructose Only Control					0.533	0.0699	0.525	-1.8	
Rxn #67	H11	Fructose Only Control					0.567	0.0699	0.525	-9.2	
Rxn #63	A1	*Angelica*	*archangelica*	1	2	200	0.378	0.0602	0.494	26.7	510.90
Rxn #63	B1	*Angelica*	*archangelica*	1	2	100	0.442	0.0602	0.494	12.0	
Rxn #63	C1	*Angelica*	*archangelica*	1	2	75	0.452	0.0602	0.494	9.7	
Rxn #63	D1	*Angelica*	*archangelica*	1	2	50	0.469	0.0602	0.494	5.8	
Rxn #63	E1	*Angelica*	*archangelica*	1	2	25	0.448	0.0602	0.494	10.6	
Rxn #63	F1	*Angelica*	*archangelica*	1	2	10	0.503	0.0602	0.494	-2.1	
Rxn #63	G1	*Angelica*	*archangelica*	1	2	1	0.522	0.0602	0.494	-6.5	
Rxn #63	H1	*Angelica*	*archangelica*	1	2	0.1	0.539	0.0602	0.494	-10.4	
Rxn #63	A2	*Angelica*	*archangelica*	1	2	200	0.384	0.0602	0.494	25.4	521.10
Rxn #63	B2	*Angelica*	*archangelica*	1	2	100	0.429	0.0602	0.494	15.0	
Rxn #63	C2	*Angelica*	*archangelica*	1	2	75	0.441	0.0602	0.494	12.2	
Rxn #63	D2	*Angelica*	*archangelica*	1	2	50	0.467	0.0602	0.494	6.2	
Rxn #63	E2	*Angelica*	*archangelica*	1	2	25	0.477	0.0602	0.494	3.9	
Rxn #63	F2	*Angelica*	*archangelica*	1	2	10	0.487	0.0602	0.494	1.6	
Rxn #63	G2	*Angelica*	*archangelica*	1	2	1	0.509	0.0602	0.494	-3.5	
Rxn #63	H2	*Angelica*	*archangelica*	1	2	0.1	0.535	0.0602	0.494	-9.5	
Rxn #63	A3	*Angelica*	*archangelica*	1	2	200	0.377	0.0602	0.494	27.0	484.70
Rxn #63	B3	*Angelica*	*archangelica*	1	2	100	0.420	0.0602	0.494	17.1	
Rxn #63	C3	*Angelica*	*archangelica*	1	2	75	0.436	0.0602	0.494	13.4	
Rxn #63	D3	*Angelica*	*archangelica*	1	2	50	0.463	0.0602	0.494	7.1	
Rxn #63	E3	*Angelica*	*archangelica*	1	2	25	0.474	0.0602	0.494	4.6	
Rxn #63	F3	*Angelica*	*archangelica*	1	2	10	0.486	0.0602	0.494	1.8	
Rxn #63	G3	*Angelica*	*archangelica*	1	2	1	0.508	0.0602	0.494	-3.2	
Rxn #63	H3	*Angelica*	*archangelica*	1	2	0.1	0.527	0.0602	0.494	-7.6	

S5 Table. Data from Titrations of Top Botanical Candidates for Inhibition of KHKC Activity.

Rxn ID	Rxn Well	Botanical Extract Genus	Species	SampleID	Lot #	Conc (μg/mL)	Sample (ΔOD_{340nm})	Ave. No Fructose Controls (ΔOD_{340nm})	Ave. Fructose Only Controls (ΔOD_{340nm})	KHKC Inhibition (%)	*KHKC IC_{50} (μg/mL)
Rxn #63	A4	*Scutellaria*	*baicalensis*	2	2	200	0.128	0.0602	0.494	84.4	22.83
Rxn #63	B4	*Scutellaria*	*baicalensis*	2	2	100	0.180	0.0602	0.494	72.4	
Rxn #63	C4	*Scutellaria*	*baicalensis*	2	2	75	0.203	0.0602	0.494	67.1	
Rxn #63	D4	*Scutellaria*	*baicalensis*	2	2	50	0.233	0.0602	0.494	60.2	
Rxn #63	E4	*Scutellaria*	*baicalensis*	2	2	25	0.256	0.0602	0.494	54.9	
Rxn #63	F4	*Scutellaria*	*baicalensis*	2	2	10	0.352	0.0602	0.494	32.7	
Rxn #63	G4	*Scutellaria*	*baicalensis*	2	2	1	0.483	0.0602	0.494	2.5	
Rxn #63	H4	*Scutellaria*	*baicalensis*	2	2	0.1	0.511	0.0602	0.494	-3.9	
Rxn #63	A5	*Scutellaria*	*baicalensis*	2	2	200	0.151	0.0602	0.494	79.1	22.21
Rxn #63	B5	*Scutellaria*	*baicalensis*	2	2	100	0.179	0.0602	0.494	72.6	
Rxn #63	C5	*Scutellaria*	*baicalensis*	2	2	75	0.206	0.0602	0.494	66.4	
Rxn #63	D5	*Scutellaria*	*baicalensis*	2	2	50	0.219	0.0602	0.494	63.4	
Rxn #63	E5	*Scutellaria*	*baicalensis*	2	2	25	0.273	0.0602	0.494	50.9	
Rxn #63	F5	*Scutellaria*	*baicalensis*	2	2	10	0.347	0.0602	0.494	33.9	
Rxn #63	G5	*Scutellaria*	*baicalensis*	2	2	1	0.481	0.0602	0.494	3.0	
Rxn #63	H5	*Scutellaria*	*baicalensis*	2	2	0.1	0.513	0.0602	0.494	-4.4	
Rxn #63	A6	*Scutellaria*	*baicalensis*	2	2	200	0.130	0.0602	0.494	83.9	25.04
Rxn #63	B6	*Scutellaria*	*baicalensis*	2	2	100	0.179	0.0602	0.494	72.6	
Rxn #63	C6	*Scutellaria*	*baicalensis*	2	2	75	0.202	0.0602	0.494	67.3	
Rxn #63	D6	*Scutellaria*	*baicalensis*	2	2	50	0.233	0.0602	0.494	60.2	
Rxn #63	E6	*Scutellaria*	*baicalensis*	2	2	25	0.263	0.0602	0.494	53.3	
Rxn #63	F6	*Scutellaria*	*baicalensis*	2	2	10	0.372	0.0602	0.494	28.1	
Rxn #63	G6	*Scutellaria*	*baicalensis*	2	2	1	0.496	0.0602	0.494	-0.5	
Rxn #63	H6	*Scutellaria*	*baicalensis*	2	2	0.1	0.510	0.0602	0.494	-3.7	
Rxn #63	A7	*Petroselinum*	*crispum*	3	2	200	0.307	0.0602	0.494	43.1	272.00
Rxn #63	B7	*Petroselinum*	*crispum*	3	2	100	0.381	0.0602	0.494	26.0	
Rxn #63	C7	*Petroselinum*	*crispum*	3	2	75	0.405	0.0602	0.494	20.5	
Rxn #63	D7	*Petroselinum*	*crispum*	3	2	50	0.437	0.0602	0.494	13.1	
Rxn #63	E7	*Petroselinum*	*crispum*	3	2	25	0.466	0.0602	0.494	6.5	
Rxn #63	F7	*Petroselinum*	*crispum*	3	2	10	0.484	0.0602	0.494	2.3	
Rxn #63	G7	*Petroselinum*	*crispum*	3	2	1	0.502	0.0602	0.494	-1.8	
Rxn #63	H7	*Petroselinum*	*crispum*	3	2	0.1	0.511	0.0602	0.494	-3.9	
Rxn #63	A8	*Petroselinum*	*crispum*	3	2	200	0.320	0.0602	0.494	40.1	305.30
Rxn #63	B8	*Petroselinum*	*crispum*	3	2	100	0.392	0.0602	0.494	23.5	
Rxn #63	C8	*Petroselinum*	*crispum*	3	2	75	0.425	0.0602	0.494	15.9	
Rxn #63	D8	*Petroselinum*	*crispum*	3	2	50	0.440	0.0602	0.494	12.4	
Rxn #63	E8	*Petroselinum*	*crispum*	3	2	25	0.473	0.0602	0.494	4.8	
Rxn #63	F8	*Petroselinum*	*crispum*	3	2	10	0.493	0.0602	0.494	0.2	
Rxn #63	G8	*Petroselinum*	*crispum*	3	2	1	0.511	0.0602	0.494	-3.9	
Rxn #63	H8	*Petroselinum*	*crispum*	3	2	0.1	0.511	0.0602	0.494	-3.9	
Rxn #63	A9	*Petroselinum*	*crispum*	3	2	200	0.304	0.0602	0.494	43.8	285.20
Rxn #63	B9	*Petroselinum*	*crispum*	3	2	100	0.390	0.0602	0.494	24.0	
Rxn #63	C9	*Petroselinum*	*crispum*	3	2	75	0.404	0.0602	0.494	20.7	
Rxn #63	D9	*Petroselinum*	*crispum*	3	2	50	0.433	0.0602	0.494	14.1	
Rxn #63	E9	*Petroselinum*	*crispum*	3	2	25	0.464	0.0602	0.494	6.9	
Rxn #63	F9	*Petroselinum*	*crispum*	3	2	10	0.485	0.0602	0.494	2.1	
Rxn #63	G9	*Petroselinum*	*crispum*	3	2	1	0.497	0.0602	0.494	-0.7	
Rxn #63	H9	*Petroselinum*	*crispum*	3	2	0.1	0.497	0.0602	0.494	-0.7	
Rxn #63	A10		No Fructose Control				0.061	0.0602	0.494	99.8	
Rxn #63	B10		No Fructose Control				0.067	0.0602	0.494	98.4	
Rxn #63	C10		No Fructose Control				0.062	0.0602	0.494	99.6	
Rxn #63	D10		No Fructose Control				0.054	0.0602	0.494	101.4	
Rxn #63	E10		No Fructose Control				0.055	0.0602	0.494	101.2	
Rxn #63	F10		No Fructose Control				0.061	0.0602	0.494	99.8	
Rxn #63	G10		No Fructose Control				0.061	0.0602	0.494	99.8	
Rxn #63	H10		No Fructose Control				0.061	0.0602	0.494	99.8	
Rxn #63	A11		Fructose Only Control				0.476	0.0602	0.494	4.1	
Rxn #63	B11		Fructose Only Control				0.495	0.0602	0.494	-0.2	
Rxn #63	C11		Fructose Only Control				0.504	0.0602	0.494	-2.3	
Rxn #63	D11		Fructose Only Control				0.491	0.0602	0.494	0.7	
Rxn #63	E11		Fructose Only Control				0.477	0.0602	0.494	3.9	
Rxn #63	F11		Fructose Only Control				0.509	0.0602	0.494	-3.5	
Rxn #63	G11		Fructose Only Control				0.504	0.0602	0.494	-2.3	
Rxn #63	H11		Fructose Only Control				0.497	0.0602	0.494	-0.7	
Rxn #64	A1	*Garcinia*	*mangostana*	4	2	200	0.185	0.0714	0.389	64.2	27.64
Rxn #64	B1	*Garcinia*	*mangostana*	4	2	100	0.161	0.0714	0.389	71.8	
Rxn #64	C1	*Garcinia*	*mangostana*	4	2	75	0.178	0.0714	0.389	66.4	
Rxn #64	D1	*Garcinia*	*mangostana*	4	2	50	0.227	0.0714	0.389	51.0	
Rxn #64	E1	*Garcinia*	*mangostana*	4	2	25	0.272	0.0714	0.389	36.8	
Rxn #64	F1	*Garcinia*	*mangostana*	4	2	10	0.404	0.0714	0.389	-4.7	
Rxn #64	G1	*Garcinia*	*mangostana*	4	2	1	0.502	0.0714	0.389	-35.6	
Rxn #64	H1	*Garcinia*	*mangostana*	4	2	0.1	0.540	0.0714	0.389	-47.5	
Rxn #64	A2	*Garcinia*	*mangostana*	4	2	200	0.173	0.0714	0.389	68.0	25.39
Rxn #64	B2	*Garcinia*	*mangostana*	4	2	100	0.170	0.0714	0.389	69.0	
Rxn #64	C2	*Garcinia*	*mangostana*	4	2	75	0.167	0.0714	0.389	69.9	
Rxn #64	D2	*Garcinia*	*mangostana*	4	2	50	0.198	0.0714	0.389	60.1	
Rxn #64	E2	*Garcinia*	*mangostana*	4	2	25	0.271	0.0714	0.389	37.2	
Rxn #64	F2	*Garcinia*	*mangostana*	4	2	10	0.391	0.0714	0.389	-0.6	
Rxn #64	G2	*Garcinia*	*mangostana*	4	2	1	0.500	0.0714	0.389	-34.9	
Rxn #64	H2	*Garcinia*	*mangostana*	4	2	0.1	0.526	0.0714	0.389	-43.1	
Rxn #64	A3	*Garcinia*	*mangostana*	4	2	200	0.177	0.0714	0.389	66.8	29.53
Rxn #64	B3	*Garcinia*	*mangostana*	4	2	100	0.170	0.0714	0.389	69.0	
Rxn #64	C3	*Garcinia*	*mangostana*	4	2	75	0.194	0.0714	0.389	61.4	
Rxn #64	D3	*Garcinia*	*mangostana*	4	2	50	0.225	0.0714	0.389	51.6	

S5 Table. Data from Titrations of Top Botanical Candidates for Inhibition of KHKC Activity.

Rxn ID	Rxn Well	Botanical Extract Genus	Species	SampleID	Lot #	Conc (μg/mL)	Sample (ΔOD_{340nm})	Ave. No Fructose Controls (ΔOD_{340nm})	Ave. Fructose Only Controls (ΔOD_{340nm})	KHKC Inhibition (%)	*KHKC IC_{50} (μg/mL)
Rxn #64	E3	*Garcinia*	*mangostana*	4	2	25	0.281	0.0714	0.389	34.0	
Rxn #64	F3	*Garcinia*	*mangostana*	4	2	10	0.405	0.0714	0.389	-5.0	
Rxn #64	G3	*Garcinia*	*mangostana*	4	2	1	0.514	0.0714	0.389	-39.4	
Rxn #64	H3	*Garcinia*	*mangostana*	4	2	0.1	0.537	0.0714	0.389	-46.6	
Rxn #64	A10	No Fructose Control					0.274	0.0714	0.389	36.2	
Rxn #64	B10	No Fructose Control					0.363	0.0714	0.389	8.2	
Rxn #64	C10	No Fructose Control					0.415	0.0714	0.389	-8.2	
Rxn #64	D10	No Fructose Control					0.440	0.0714	0.389	-16.1	
Rxn #64	E10	No Fructose Control					0.423	0.0714	0.389	-10.7	
Rxn #64	F10	No Fructose Control					0.417	0.0714	0.389	-8.8	
Rxn #64	G10	No Fructose Control					0.415	0.0714	0.389	-8.2	
Rxn #64	H10	No Fructose Control					0.368	0.0714	0.389	6.6	
Rxn #64	A11	Fructose Only Control					0.082	0.0714	0.389	96.7	
Rxn #64	B11	Fructose Only Control					0.076	0.0714	0.389	98.6	
Rxn #64	C11	Fructose Only Control					0.071	0.0714	0.389	100.1	
Rxn #64	D11	Fructose Only Control					0.070	0.0714	0.389	100.4	
Rxn #64	E11	Fructose Only Control					0.070	0.0714	0.389	100.4	
Rxn #64	F11	Fructose Only Control					0.065	0.0714	0.389	102.0	
Rxn #64	G11	Fructose Only Control					0.064	0.0714	0.389	102.3	
Rxn #64	H11	Fructose Only Control					0.073	0.0714	0.389	99.5	

IC_{50}: half maximal inhibitory concentration. OD: optical density.

*KHKC IC_{50}s were calculated using nonlinear regression (three parameters) in GraphPad Prism 5.03. To generate a best fit, an upper concentration (10,000 μg/mL at 100% inhibition) and a lower concentration (0.001 μg/mL at 0% inhibition) were added.
