# Supplementary material for: Bioactivity-Guided Identification of Botanical Inhibitors of Ketohexokinase
Source: PLoS One. 2016 Jun 20;11(6):e0157458. doi: 10.1371/journal.pone.0157458 (PMC4913896; doi:10.1371/journal.pone.0157458)
Supplement: S6 Table — IC50: half maximal inhibitory concentration. OD: optical density. *ATP Depletion IC50s were calculated using nonlinear regression (three parameters) in GraphPad Prism 5.03. To generate a best fit, an upper concentration (10,000 μg/mL at 100% inhibition) and a lower concentration (0.001 μg/mL at 0% inhibition) were added. (PDF) [file pone.0157458.s006.pdf]

S6 Table. Data from Titrations of Top Botanical Candidates for Inhibition of Fructose-Induced ATP Depletion.

Rxn		Botanical Extract					Sample	Ave. No Fructose Controls	Ave. Fructose Only Controls	ATP Depletion	*ATP Depletion IC_{50}
ID	Well	Genus	Species	SampleID	Lot #	Conc (μg/mL)	(ΔOD_{570nm})	(ΔOD_{570nm})	(ΔOD_{570nm})	(%)	(μg/mL)
ATP #12	A1	No Fructose Control					0.147	0.139	1.068	99.1	
ATP #12	B1	No Fructose Control					0.133	0.139	1.068	100.6	
ATP #12	C1	No Fructose Control					0.065	0.139	1.068	108.0	
ATP #12	D1	No Fructose Control					0.142	0.139	1.068	99.7	
ATP #12	E1	No Fructose Control					0.221	0.139	1.068	91.2	
ATP #12	F1	No Fructose Control					0.134	0.139	1.068	100.5	
ATP #12	G1	No Fructose Control					0.163	0.139	1.068	97.4	
ATP #12	H1	No Fructose Control					0.114	0.139	1.068	102.7	
ATP #12	A2	Fructose Only Control					1.093	0.139	1.068	-2.7	
ATP #12	B2	Fructose Only Control					1.055	0.139	1.068	1.4	
ATP #12	C2	Fructose Only Control					0.981	0.139	1.068	9.4	
ATP #12	D2	Fructose Only Control					1.063	0.139	1.068	0.5	
ATP #12	E2	Fructose Only Control					1.162	0.139	1.068	-10.1	
ATP #12	F2	Fructose Only Control					1.061	0.139	1.068	0.8	
ATP #12	G2	Fructose Only Control					1.062	0.139	1.068	0.6	
ATP #12	H2	Fructose Only Control					0.927	0.139	1.068	15.2	
ATP #12	A3	*Angelica*	*archangelica*	1	1	500	-0.234	0.139	1.068	140.2	60.17
ATP #12	B3	*Angelica*	*archangelica*	1	1	250	-0.182	0.139	1.068	134.6	
ATP #12	C3	*Angelica*	*archangelica*	1	1	125	0.434	0.139	1.068	68.2	
ATP #12	D3	*Angelica*	*archangelica*	1	1	50	0.357	0.139	1.068	76.5	
ATP #12	E3	*Angelica*	*archangelica*	1	1	25	1.044	0.139	1.068	2.6	
ATP #12	F3	*Angelica*	*archangelica*	1	1	10	1.189	0.139	1.068	-13.0	
ATP #12	G3	*Angelica*	*archangelica*	1	1	1	1.118	0.139	1.068	-5.4	
ATP #12	H3	*Angelica*	*archangelica*	1	1	0.1	1.096	0.139	1.068	-3.0	
ATP #12	A4	*Angelica*	*archangelica*	1	1	500	0.06	0.139	1.068	108.5	85.98
ATP #12	B4	*Angelica*	*archangelica*	1	1	250	0.071	0.139	1.068	107.3	
ATP #12	C4	*Angelica*	*archangelica*	1	1	125	0.437	0.139	1.068	67.9	
ATP #12	D4	*Angelica*	*archangelica*	1	1	50	0.605	0.139	1.068	49.8	
ATP #12	E4	*Angelica*	*archangelica*	1	1	25	1.096	0.139	1.068	-3.0	
ATP #12	F4	*Angelica*	*archangelica*	1	1	10	1.219	0.139	1.068	-16.3	
ATP #12	G4	*Angelica*	*archangelica*	1	1	1	1.172	0.139	1.068	-11.2	
ATP #12	H4	*Angelica*	*archangelica*	1	1	0.1	0.801	0.139	1.068	28.7	
ATP #12	A5	*Angelica*	*archangelica*	1	1	500	-0.129	0.139	1.068	128.8	77.49
ATP #12	B5	*Angelica*	*archangelica*	1	1	250	-0.009	0.139	1.068	115.9	
ATP #12	C5	*Angelica*	*archangelica*	1	1	125	0.409	0.139	1.068	70.9	
ATP #12	D5	*Angelica*	*archangelica*	1	1	50	0.725	0.139	1.068	36.9	
ATP #12	E5	*Angelica*	*archangelica*	1	1	25	0.953	0.139	1.068	12.4	
ATP #12	F5	*Angelica*	*archangelica*	1	1	10	1.041	0.139	1.068	2.9	
ATP #12	G5	*Angelica*	*archangelica*	1	1	1	1.112	0.139	1.068	-4.7	
ATP #12	H5	*Angelica*	*archangelica*	1	1	0.1	1.049	0.139	1.068	2.0	
ATP #13	A1	No Fructose Control					-0.694	-0.653	0.561	103.4	
ATP #13	B1	No Fructose Control					-0.68	-0.653	0.561	102.2	
ATP #13	C1	No Fructose Control					-0.719	-0.653	0.561	105.4	
ATP #13	D1	No Fructose Control					-0.74	-0.653	0.561	107.2	
ATP #13	E1	No Fructose Control					-0.593	-0.653	0.561	95.1	
ATP #13	F1	No Fructose Control					-0.609	-0.653	0.561	96.4	
ATP #13	G1	No Fructose Control					-0.582	-0.653	0.561	94.2	
ATP #13	H1	No Fructose Control					-0.607	-0.653	0.561	96.2	
ATP #13	A2	Fructose Only Control					0.524	-0.653	0.561	3.0	
ATP #13	B2	Fructose Only Control					0.593	-0.653	0.561	-2.6	
ATP #13	C2	Fructose Only Control					0.528	-0.653	0.561	2.7	
ATP #13	D2	Fructose Only Control					0.54	-0.653	0.561	1.7	
ATP #13	E2	Fructose Only Control					0.614	-0.653	0.561	-4.4	
ATP #13	F2	Fructose Only Control					0.609	-0.653	0.561	-4.0	
ATP #13	G2	Fructose Only Control					0.516	-0.653	0.561	3.7	
ATP #13	H2	Fructose Only Control					0.728	-0.653	0.561	-13.8	
ATP #13	A3	*Scutellaria*	*baicalensis*	4	1	500	-0.469	-0.653	0.561	84.8	66.59
ATP #13	B3	*Scutellaria*	*baicalensis*	4	1	250	-0.553	-0.653	0.561	91.8	
ATP #13	C3	*Scutellaria*	*baicalensis*	4	1	125	-0.109	-0.653	0.561	55.2	
ATP #13	D3	*Scutellaria*	*baicalensis*	4	1	50	-0.091	-0.653	0.561	53.7	
ATP #13	E3	*Scutellaria*	*baicalensis*	4	1	25	0.281	-0.653	0.561	23.1	
ATP #13	F3	*Scutellaria*	*baicalensis*	4	1	10	0.379	-0.653	0.561	15.0	
ATP #13	G3	*Scutellaria*	*baicalensis*	4	1	1	0.512	-0.653	0.561	4.0	
ATP #13	H3	*Scutellaria*	*baicalensis*	4	1	0.1	0.502	-0.653	0.561	4.9	
ATP #13	A4	*Scutellaria*	*baicalensis*	4	1	500	-0.536	-0.653	0.561	90.4	74.15
ATP #13	B4	*Scutellaria*	*baicalensis*	4	1	250	-0.504	-0.653	0.561	87.7	
ATP #13	C4	*Scutellaria*	*baicalensis*	4	1	125	-0.085	-0.653	0.561	53.2	
ATP #13	D4	*Scutellaria*	*baicalensis*	4	1	50	-0.08	-0.653	0.561	52.8	
ATP #13	E4	*Scutellaria*	*baicalensis*	4	1	25	0.298	-0.653	0.561	21.7	
ATP #13	F4	*Scutellaria*	*baicalensis*	4	1	10	0.447	-0.653	0.561	9.4	
ATP #13	G4	*Scutellaria*	*baicalensis*	4	1	1	0.491	-0.653	0.561	5.8	
ATP #13	H4	*Scutellaria*	*baicalensis*	4	1	0.1	0.532	-0.653	0.561	2.4	
ATP #13	A5	*Scutellaria*	*baicalensis*	4	1	500	-0.534	-0.653	0.561	90.2	79.27
ATP #13	B5	*Scutellaria*	*baicalensis*	4	1	250	-0.582	-0.653	0.561	94.2	
ATP #13	C5	*Scutellaria*	*baicalensis*	4	1	125	-0.156	-0.653	0.561	59.1	
ATP #13	D5	*Scutellaria*	*baicalensis*	4	1	50	0.107	-0.653	0.561	37.4	
ATP #13	E5	*Scutellaria*	*baicalensis*	4	1	25	0.258	-0.653	0.561	25.0	
ATP #13	F5	*Scutellaria*	*baicalensis*	4	1	10	0.383	-0.653	0.561	14.7	
ATP #13	G5	*Scutellaria*	*baicalensis*	4	1	1	0.521	-0.653	0.561	3.3	
ATP #13	H5	*Scutellaria*	*baicalensis*	4	1	0.1	0.527	-0.653	0.561	2.8	
ATP #13	A6	*Petroselinum*	*crispum*	6	1	500	-0.342	-0.653	0.561	74.4	131.90
ATP #13	B6	*Petroselinum*	*crispum*	6	1	250	-0.516	-0.653	0.561	88.7	
ATP #13	C6	*Petroselinum*	*crispum*	6	1	125	0.205	-0.653	0.561	29.3	
ATP #13	D6	*Petroselinum*	*crispum*	6	1	50	0.199	-0.653	0.561	29.8	
ATP #13	E6	*Petroselinum*	*crispum*	6	1	25	0.398	-0.653	0.561	13.4	
ATP #13	F6	*Petroselinum*	*crispum*	6	1	10	0.361	-0.653	0.561	16.5	
ATP #13	G6	*Petroselinum*	*crispum*	6	1	1	0.604	-0.653	0.561	-3.5	
ATP #13	H6	*Petroselinum*	*crispum*	6	1	0.1	0.643	-0.653	0.561	-6.8	
ATP #13	A7	*Petroselinum*	*crispum*	6	1	500	-0.296	-0.653	0.561	70.6	228.50
ATP #13	B7	*Petroselinum*	*crispum*	6	1	250	-0.326	-0.653	0.561	73.1	
ATP #13	C7	*Petroselinum*	*crispum*	6	1	125	0.086	-0.653	0.561	39.1	
ATP #13	D7	*Petroselinum*	*crispum*	6	1	50	0.172	-0.653	0.561	32.0	
ATP #13	E7	*Petroselinum*	*crispum*	6	1	25	0.406	-0.653	0.561	12.8	

S6 Table. Data from Titrations of Top Botanical Candidates for Inhibition of Fructose-Induced ATP Depletion.

Rxn		Botanical Extract					Sample	Ave. No Fructose Controls	Ave. Fructose Only Controls	ATP Depletion	*ATP Depletion IC$_{50}$
ID	Well	Genus	Species	SampleID	Lot #	Conc (μg/mL)	(ΔOD_{570nm})	(ΔOD_{570nm})	(ΔOD_{570nm})	(%)	(μg/mL)
ATP #13	F7	*Petroselinum*	*crispum*	6	1	10	0.462	-0.653	0.561	8.2	
ATP #13	G7	*Petroselinum*	*crispum*	6	1	1	0.136	-0.653	0.561	35.0	
ATP #13	H7	*Petroselinum*	*crispum*	6	1	0.1	0.285	-0.653	0.561	22.7	
ATP #13	A8	*Petroselinum*	*crispum*	6	1	500	-0.24	-0.653	0.561	66.0	87.74
ATP #13	B8	*Petroselinum*	*crispum*	6	1	250	-0.334	-0.653	0.561	73.7	
ATP #13	C8	*Petroselinum*	*crispum*	6	1	125	0.135	-0.653	0.561	35.1	
ATP #13	D8	*Petroselinum*	*crispum*	6	1	50	0.294	-0.653	0.561	22.0	
ATP #13	E8	*Petroselinum*	*crispum*	6	1	25	0.407	-0.653	0.561	12.7	
ATP #13	F8	*Petroselinum*	*crispum*	6	1	10	0.544	-0.653	0.561	1.4	
ATP #13	G8	*Petroselinum*	*crispum*	6	1	1	1.023	-0.653	0.561	-38.1	
ATP #13	H8	*Petroselinum*	*crispum*	6	1	0.1	1.004	-0.653	0.561	-36.5	
ATP #13	A9	*Garcinia*	*mangostana*	8	1	500	-0.507	-0.653	0.561	88.0	27.46
ATP #13	B9	*Garcinia*	*mangostana*	8	1	250	-0.328	-0.653	0.561	73.2	
ATP #13	C9	*Garcinia*	*mangostana*	8	1	125	-0.457	-0.653	0.561	83.9	
ATP #13	D9	*Garcinia*	*mangostana*	8	1	50	-0.443	-0.653	0.561	82.7	
ATP #13	E9	*Garcinia*	*mangostana*	8	1	25	0.311	-0.653	0.561	20.6	
ATP #13	F9	*Garcinia*	*mangostana*	8	1	10	0.463	-0.653	0.561	8.1	
ATP #13	G9	*Garcinia*	*mangostana*	8	1	1	0.704	-0.653	0.561	-11.8	
ATP #13	H9	*Garcinia*	*mangostana*	8	1	0.1	0.898	-0.653	0.561	-27.8	
ATP #13	A10	*Garcinia*	*mangostana*	8	1	500	-0.55	-0.653	0.561	91.5	32.91
ATP #13	B10	*Garcinia*	*mangostana*	8	1	250	-0.382	-0.653	0.561	77.7	
ATP #13	C10	*Garcinia*	*mangostana*	8	1	125	-0.584	-0.653	0.561	94.3	
ATP #13	D10	*Garcinia*	*mangostana*	8	1	50	-0.411	-0.653	0.561	80.1	
ATP #13	E10	*Garcinia*	*mangostana*	8	1	25	0.201	-0.653	0.561	29.7	
ATP #13	F10	*Garcinia*	*mangostana*	8	1	10	0.474	-0.653	0.561	7.2	
ATP #13	G10	*Garcinia*	*mangostana*	8	1	1	0.55	-0.653	0.561	0.9	
ATP #13	H10	*Garcinia*	*mangostana*	8	1	0.1	0.499	-0.653	0.561	5.1	
ATP #13	A11	*Garcinia*	*mangostana*	8	1	500	-0.321	-0.653	0.561	72.7	30.78
ATP #13	B11	*Garcinia*	*mangostana*	8	1	250	-0.507	-0.653	0.561	88.0	
ATP #13	C11	*Garcinia*	*mangostana*	8	1	125	-0.552	-0.653	0.561	91.7	
ATP #13	D11	*Garcinia*	*mangostana*	8	1	50	-0.307	-0.653	0.561	71.5	
ATP #13	E11	*Garcinia*	*mangostana*	8	1	25	0.188	-0.653	0.561	30.7	
ATP #13	F11	*Garcinia*	*mangostana*	8	1	10	0.449	-0.653	0.561	9.2	
ATP #13	G11	*Garcinia*	*mangostana*	8	1	1	0.608	-0.653	0.561	-3.9	
ATP #13	H11	*Garcinia*	*mangostana*	8	1	0.1	0.562	-0.653	0.561	-0.1	
ATP #21	A1	No Fructose Control					0.189	0.231	1.39	103.6	
ATP #21	B1	No Fructose Control					0.166	0.231	1.39	105.6	
ATP #21	C1	No Fructose Control					0.178	0.231	1.39	104.6	
ATP #21	D1	No Fructose Control					0.179	0.231	1.39	104.5	
ATP #21	E1	No Fructose Control					0.285	0.231	1.39	95.3	
ATP #21	F1	No Fructose Control					0.327	0.231	1.39	91.7	
ATP #21	G1	No Fructose Control					0.243	0.231	1.39	99.0	
ATP #21	H1	No Fructose Control					0.279	0.231	1.39	95.9	
ATP #21	A2	Fructose Only Control					1.354	0.231	1.39	3.1	
ATP #21	B2	Fructose Only Control					1.375	0.231	1.39	1.3	
ATP #21	C2	Fructose Only Control					1.420	0.231	1.39	-2.6	
ATP #21	D2	Fructose Only Control					1.380	0.231	1.39	0.9	
ATP #21	E2	Fructose Only Control					1.457	0.231	1.39	-5.8	
ATP #21	F2	Fructose Only Control					1.406	0.231	1.39	-1.4	
ATP #21	G2	Fructose Only Control					1.368	0.231	1.39	1.9	
ATP #21	H2	Fructose Only Control					1.359	0.231	1.39	2.7	
ATP #21	A3	*Angelica*	*archangelica*	1	2	500	0.880	0.231	1.39	44.0	772.20
ATP #21	B3	*Angelica*	*archangelica*	1	2	250	1.066	0.231	1.39	28.0	
ATP #21	C3	*Angelica*	*archangelica*	1	2	125	1.258	0.231	1.39	11.4	
ATP #21	D3	*Angelica*	*archangelica*	1	2	50	1.313	0.231	1.39	6.6	
ATP #21	E3	*Angelica*	*archangelica*	1	2	25	1.343	0.231	1.39	4.1	
ATP #21	F3	*Angelica*	*archangelica*	1	2	10	1.377	0.231	1.39	1.1	
ATP #21	G3	*Angelica*	*archangelica*	1	2	1	1.359	0.231	1.39	2.7	
ATP #21	H3	*Angelica*	*archangelica*	1	2	0.1	1.369	0.231	1.39	1.8	
ATP #21	A4	*Angelica*	*archangelica*	1	2	500	0.816	0.231	1.39	49.5	510.20
ATP #21	B4	*Angelica*	*archangelica*	1	2	250	1.124	0.231	1.39	23.0	
ATP #21	C4	*Angelica*	*archangelica*	1	2	125	1.272	0.231	1.39	10.2	
ATP #21	D4	*Angelica*	*archangelica*	1	2	50	1.311	0.231	1.39	6.8	
ATP #21	E4	*Angelica*	*archangelica*	1	2	25	1.407	0.231	1.39	-1.5	
ATP #21	F4	*Angelica*	*archangelica*	1	2	10	1.421	0.231	1.39	-2.7	
ATP #21	G4	*Angelica*	*archangelica*	1	2	1	1.381	0.231	1.39	0.8	
ATP #21	H4	*Angelica*	*archangelica*	1	2	0.1	1.883	0.231	1.39	-42.5	
ATP #21	A5	*Angelica*	*archangelica*	1	2	500	0.929	0.231	1.39	39.8	1052.00
ATP #21	B5	*Angelica*	*archangelica*	1	2	250	1.204	0.231	1.39	16.0	
ATP #21	C5	*Angelica*	*archangelica*	1	2	125	1.145	0.231	1.39	21.1	
ATP #21	D5	*Angelica*	*archangelica*	1	2	50	1.243	0.231	1.39	12.7	
ATP #21	E5	*Angelica*	*archangelica*	1	2	25	1.284	0.231	1.39	9.1	
ATP #21	F5	*Angelica*	*archangelica*	1	2	10	1.296	0.231	1.39	8.1	
ATP #21	G5	*Angelica*	*archangelica*	1	2	1	1.481	0.231	1.39	-7.9	
ATP #21	H5	*Angelica*	*archangelica*	1	2	0.1	1.292	0.231	1.39	8.5	
ATP #21	A6	*Scutellaria*	*baicalensis*	2	2	500	0.241	0.231	1.39	99.1	26.11
ATP #21	B6	*Scutellaria*	*baicalensis*	2	2	250	0.211	0.231	1.39	101.7	
ATP #21	C6	*Scutellaria*	*baicalensis*	2	2	125	0.244	0.231	1.39	98.9	
ATP #21	D6	*Scutellaria*	*baicalensis*	2	2	50	0.530	0.231	1.39	74.2	
ATP #21	E6	*Scutellaria*	*baicalensis*	2	2	25	0.849	0.231	1.39	46.7	
ATP #21	F6	*Scutellaria*	*baicalensis*	2	2	10	1.133	0.231	1.39	22.2	
ATP #21	G6	*Scutellaria*	*baicalensis*	2	2	1	1.311	0.231	1.39	6.8	
ATP #21	H6	*Scutellaria*	*baicalensis*	2	2	0.1	1.414	0.231	1.39	-2.1	
ATP #21	A7	*Scutellaria*	*baicalensis*	2	2	500	0.287	0.231	1.39	95.2	29.55
ATP #21	B7	*Scutellaria*	*baicalensis*	2	2	250	0.259	0.231	1.39	97.6	
ATP #21	C7	*Scutellaria*	*baicalensis*	2	2	125	0.304	0.231	1.39	93.7	
ATP #21	D7	*Scutellaria*	*baicalensis*	2	2	50	0.567	0.231	1.39	71.0	
ATP #21	E7	*Scutellaria*	*baicalensis*	2	2	25	0.898	0.231	1.39	42.5	
ATP #21	F7	*Scutellaria*	*baicalensis*	2	2	10	1.130	0.231	1.39	22.4	
ATP #21	G7	*Scutellaria*	*baicalensis*	2	2	1	1.331	0.231	1.39	5.1	
ATP #21	H7	*Scutellaria*	*baicalensis*	2	2	0.1	1.325	0.231	1.39	5.6	
ATP #21	A8	*Scutellaria*	*baicalensis*	2	2	500	0.251	0.231	1.39	98.3	28.91
ATP #21	B8	*Scutellaria*	*baicalensis*	2	2	250	0.261	0.231	1.39	97.4	

S6 Table. Data from Titrations of Top Botanical Candidates for Inhibition of Fructose-Induced ATP Depletion.

Rxn		Botanical Extract					Sample	Ave. No Fructose Controls	Ave. Fructose Only Controls	ATP Depletion	*ATP Depletion IC_{50}
ID	Well	Genus	Species	SampleID	Lot #	Conc (μg/mL)	(ΔOD_{570nm})	(ΔOD_{570nm})	(ΔOD_{570nm})	(%)	(μg/mL)
ATP #21	C8	*Scutellaria*	*baicalensis*	2	2	125	0.269	0.231	1.39	96.7	
ATP #21	D8	*Scutellaria*	*baicalensis*	2	2	50	0.582	0.231	1.39	69.7	
ATP #21	E8	*Scutellaria*	*baicalensis*	2	2	25	0.905	0.231	1.39	41.8	
ATP #21	F8	*Scutellaria*	*baicalensis*	2	2	10	1.143	0.231	1.39	21.3	
ATP #21	G8	*Scutellaria*	*baicalensis*	2	2	1	1.400	0.231	1.39	-0.9	
ATP #21	H8	*Scutellaria*	*baicalensis*	2	2	0.1	1.387	0.231	1.39	0.3	
ATP #21	A9	*Petroselinum*	*crispum*	3	2	500	0.515	0.231	1.39	75.5	223.30
ATP #21	B9	*Petroselinum*	*crispum*	3	2	250	0.524	0.231	1.39	74.7	
ATP #21	C9	*Petroselinum*	*crispum*	3	2	125	1.039	0.231	1.39	30.3	
ATP #21	D9	*Petroselinum*	*crispum*	3	2	50	1.133	0.231	1.39	22.2	
ATP #21	E9	*Petroselinum*	*crispum*	3	2	25	1.204	0.231	1.39	16.0	
ATP #21	F9	*Petroselinum*	*crispum*	3	2	10	1.202	0.231	1.39	16.2	
ATP #21	G9	*Petroselinum*	*crispum*	3	2	1	1.214	0.231	1.39	15.2	
ATP #21	H9	*Petroselinum*	*crispum*	3	2	0.1	1.192	0.231	1.39	17.1	
ATP #21	A10	*Petroselinum*	*crispum*	3	2	500	0.496	0.231	1.39	77.1	215.60
ATP #21	B10	*Petroselinum*	*crispum*	3	2	250	0.533	0.231	1.39	73.9	
ATP #21	C10	*Petroselinum*	*crispum*	3	2	125	1.049	0.231	1.39	29.4	
ATP #21	D10	*Petroselinum*	*crispum*	3	2	50	1.134	0.231	1.39	22.1	
ATP #21	E10	*Petroselinum*	*crispum*	3	2	25	1.122	0.231	1.39	23.1	
ATP #21	F10	*Petroselinum*	*crispum*	3	2	10	1.178	0.231	1.39	18.3	
ATP #21	G10	*Petroselinum*	*crispum*	3	2	1	1.247	0.231	1.39	12.3	
ATP #21	H10	*Petroselinum*	*crispum*	3	2	0.1	1.228	0.231	1.39	14.0	
ATP #21	A11	*Petroselinum*	*crispum*	3	2	500	0.482	0.231	1.39	78.3	214.30
ATP #21	B11	*Petroselinum*	*crispum*	3	2	250	0.484	0.231	1.39	78.2	
ATP #21	C11	*Petroselinum*	*crispum*	3	2	125	0.815	0.231	1.39	49.6	
ATP #21	D11	*Petroselinum*	*crispum*	3	2	50	0.942	0.231	1.39	38.7	
ATP #21	E11	*Petroselinum*	*crispum*	3	2	25	0.964	0.231	1.39	36.8	
ATP #21	F11	*Petroselinum*	*crispum*	3	2	10	0.985	0.231	1.39	34.9	
ATP #21	G11	*Petroselinum*	*crispum*	3	2	1	0.885	0.231	1.39	43.6	
ATP #21	H11	*Petroselinum*	*crispum*	3	2	0.1	0.867	0.231	1.39	45.1	
ATP #22	A1		No Fructose Control				-0.616	-0.591	0.884	101.7	
ATP #22	B1		No Fructose Control				-0.601	-0.591	0.884	100.7	
ATP #22	C1		No Fructose Control				-0.588	-0.591	0.884	99.8	
ATP #22	D1		No Fructose Control				-0.583	-0.591	0.884	99.5	
ATP #22	E1		No Fructose Control				-0.468	-0.591	0.884	91.7	
ATP #22	F1		No Fructose Control				-0.656	-0.591	0.884	104.4	
ATP #22	G1		No Fructose Control				-0.771	-0.591	0.884	112.2	
ATP #22	H1		No Fructose Control				-0.623	-0.591	0.884	102.2	
ATP #22	A2		Fructose Only Control				0.889	-0.591	0.884	-0.3	
ATP #22	B2		Fructose Only Control				0.901	-0.591	0.884	-1.2	
ATP #22	C2		Fructose Only Control				0.940	-0.591	0.884	-3.8	
ATP #22	D2		Fructose Only Control				0.921	-0.591	0.884	-2.5	
ATP #22	E2		Fructose Only Control				0.874	-0.591	0.884	0.7	
ATP #22	F2		Fructose Only Control				0.889	-0.591	0.884	-0.3	
ATP #22	G2		Fructose Only Control				0.735	-0.591	0.884	10.1	
ATP #22	H2		Fructose Only Control				0.773	-0.591	0.884	7.5	
ATP #22	A3	*Garcinia*	*mangostana*	4	2	500	-0.148	-0.591	0.884	70.0	69.20
ATP #22	B3	*Garcinia*	*mangostana*	4	2	250	-0.239	-0.591	0.884	76.1	
ATP #22	C3	*Garcinia*	*mangostana*	4	2	125	-0.100	-0.591	0.884	66.7	
ATP #22	D3	*Garcinia*	*mangostana*	4	2	50	0.162	-0.591	0.884	48.9	
ATP #22	E3	*Garcinia*	*mangostana*	4	2	25	0.606	-0.591	0.884	18.8	
ATP #22	F3	*Garcinia*	*mangostana*	4	2	10	0.855	-0.591	0.884	2.0	
ATP #22	G3	*Garcinia*	*mangostana*	4	2	1	0.810	-0.591	0.884	5.0	
ATP #22	H3	*Garcinia*	*mangostana*	4	2	0.1	0.799	-0.591	0.884	5.8	
ATP #22	A4	*Garcinia*	*mangostana*	4	2	500	-0.164	-0.591	0.884	71.1	61.05
ATP #22	B4	*Garcinia*	*mangostana*	4	2	250	-0.260	-0.591	0.884	77.6	
ATP #22	C4	*Garcinia*	*mangostana*	4	2	125	-0.155	-0.591	0.884	70.4	
ATP #22	D4	*Garcinia*	*mangostana*	4	2	50	0.133	-0.591	0.884	50.9	
ATP #22	E4	*Garcinia*	*mangostana*	4	2	25	0.597	-0.591	0.884	19.5	
ATP #22	F4	*Garcinia*	*mangostana*	4	2	10	0.815	-0.591	0.884	4.7	
ATP #22	G4	*Garcinia*	*mangostana*	4	2	1	0.811	-0.591	0.884	4.9	
ATP #22	H4	*Garcinia*	*mangostana*	4	2	0.1	0.842	-0.591	0.884	2.8	
ATP #22	A5	*Garcinia*	*mangostana*	4	2	500	-0.112	-0.591	0.884	67.5	59.19
ATP #22	B5	*Garcinia*	*mangostana*	4	2	250	-0.147	-0.591	0.884	69.9	
ATP #22	C5	*Garcinia*	*mangostana*	4	2	125	-0.075	-0.591	0.884	65.0	
ATP #22	D5	*Garcinia*	*mangostana*	4	2	50	0.078	-0.591	0.884	54.6	
ATP #22	E5	*Garcinia*	*mangostana*	4	2	25	0.424	-0.591	0.884	31.2	
ATP #22	F5	*Garcinia*	*mangostana*	4	2	10	0.808	-0.591	0.884	5.2	
ATP #22	G5	*Garcinia*	*mangostana*	4	2	1	0.689	-0.591	0.884	13.2	
ATP #22	H5	*Garcinia*	*mangostana*	4	2	0.1	0.734	-0.591	0.884	10.2	

IC_{50}: half maximal inhibitory concentration. OD: optical density.

*ATP Depletion IC_{50}s were calculated using nonlinear regression (three parameters) in GraphPad Prism 5.03. To generate a best fit, an upper concentration (10,000 μg/mL at 100% inhibition) and a lower concentration (0.001 μg/mL at 0% inhibition) were added.
