# Supplementary material for: Bioactivity-Guided Identification of Botanical Inhibitors of Ketohexokinase
Source: PLoS One. 2016 Jun 20;11(6):e0157458. doi: 10.1371/journal.pone.0157458 (PMC4913896; doi:10.1371/journal.pone.0157458)
Supplement: S7 Table — IC50: half maximal inhibitory concentration. OD: optical density. TG: triglycerides. *TG IC50s were calculated using nonlinear regression (three parameters) in GraphPad Prism 5.03. To generate a best fit, an upper concentration (10,000 μg/mL at 100% inhibition) and a lower concentration (0.001 μg/mL at 0% inhibition) were added. (PDF) [file pone.0157458.s007.pdf]

S7 Table. Data from Titrations of Top Botanical Candidates for Inhibition of Fructose-induced Elevation in TG Levels.

Botanical Extract					TG (mg/dL)	TG (mg/dL)	ΔTG	TG Inhibition	*TG IC_{50}
Genus	Species	SampleID	Lot #	Conc (µg/mL)	(Fructose Only Controls)	(No Fructose Controls)	(mg/dL)	(%)	(µg/mL)
Angelica	*archangelica*	1	1	500	36	39	-3	107.0	37.93
Angelica	*archangelica*	1	1	200	44	42	2	95.3	
Angelica	*archangelica*	1	1	100	56	42	14	67.4	
Angelica	*archangelica*	1	1	50	57	46	11	74.4	
Angelica	*archangelica*	1	1	25	75	44	31	27.9	
Angelica	*archangelica*	1	1	12.5	82	51	31	27.9	
Angelica	*archangelica*	1	1	0.6	99	48	51	-18.6	
Angelica	*archangelica*	1	1	0.3	95	55	40	7.0	
Angelica	*archangelica*	1	1	0.1	103	53	50	-16.3	
Angelica	*archangelica*	1	1	0	96	53	43	0.0	
Angelica	*archangelica*	1	1	500	40	41	-1	102.5	146.50
Angelica	*archangelica*	1	1	200	42	44	-2	105.0	
Angelica	*archangelica*	1	1	100	61	39	22	45.0	
Angelica	*archangelica*	1	1	50	68	34	34	15.0	
Angelica	*archangelica*	1	1	25	78	45	33	17.5	
Angelica	*archangelica*	1	1	12.5	92	46	46	-15.0	
Angelica	*archangelica*	1	1	0.6	93	48	45	-12.5	
Angelica	*archangelica*	1	1	0.3	89	51	38	5.0	
Angelica	*archangelica*	1	1	0.1	99	50	49	-22.5	
Angelica	*archangelica*	1	1	0	95	55	40	0.0	
Scutellaria	*baicalensis*	4	1	500	42	46	-4	109.3	74.79
Scutellaria	*baicalensis*	4	1	200	40	46	-6	114.0	
Scutellaria	*baicalensis*	4	1	100	56	42	14	67.4	
Scutellaria	*baicalensis*	4	1	50	68	44	24	44.2	
Scutellaria	*baicalensis*	4	1	25	81	45	36	16.3	
Scutellaria	*baicalensis*	4	1	12.5	86	45	41	4.7	
Scutellaria	*baicalensis*	4	1	0.6	81	40	41	4.7	
Scutellaria	*baicalensis*	4	1	0.3	94	39	55	-27.9	
Scutellaria	*baicalensis*	4	1	0.1	99	46	53	-23.3	
Scutellaria	*baicalensis*	4	1	0	89	46	43	0.0	
Scutellaria	*baicalensis*	4	1	500	42	42	0	100.0	30.44
Scutellaria	*baicalensis*	4	1	200	44	42	2	96.5	
Scutellaria	*baicalensis*	4	1	100	38	45	-7	112.3	
Scutellaria	*baicalensis*	4	1	50	56	46	10	82.5	
Scutellaria	*baicalensis*	4	1	25	75	47	28	50.9	
Scutellaria	*baicalensis*	4	1	12.5	85	48	37	35.1	
Scutellaria	*baicalensis*	4	1	0.6	89	51	38	33.3	
Scutellaria	*baicalensis*	4	1	0.3	99	52	47	17.5	
Scutellaria	*baicalensis*	4	1	0.1	103	49	54	5.3	
Scutellaria	*baicalensis*	4	1	0	106	49	57	0.0	
Petroselinum	*crispum*	6	1	500	46	42	4	92.0	414.00
Petroselinum	*crispum*	6	1	200	65	46	19	62.0	
Petroselinum	*crispum*	6	1	100	78	44	34	32.0	
Petroselinum	*crispum*	6	1	50	90	45	45	10.0	
Petroselinum	*crispum*	6	1	25	91	51	40	20.0	
Petroselinum	*crispum*	6	1	12.5	89	52	37	26.0	
Petroselinum	*crispum*	6	1	0.6	96	46	50	0.0	
Petroselinum	*crispum*	6	1	0.3	94	44	50	0.0	
Petroselinum	*crispum*	6	1	0.1	102	52	50	0.0	
Petroselinum	*crispum*	6	1	0	89	39	50	0.0	
Petroselinum	*crispum*	6	1	500	44	42	2	95.7	385.50
Petroselinum	*crispum*	6	1	200	50	38	12	73.9	
Petroselinum	*crispum*	6	1	100	68	50	18	60.9	
Petroselinum	*crispum*	6	1	50	88	46	42	8.7	
Petroselinum	*crispum*	6	1	25	86	44	42	8.7	
Petroselinum	*crispum*	6	1	12.5	88	50	38	17.4	
Petroselinum	*crispum*	6	1	0.6	89	56	33	28.3	
Petroselinum	*crispum*	6	1	0.3	99	62	37	19.6	
Petroselinum	*crispum*	6	1	0.1	102	65	37	19.6	
Petroselinum	*crispum*	6	1	0	104	58	46	0.0	
Garcinia	*mangostana*	8	1	500	39	46	-7	111.9	101.80
Garcinia	*mangostana*	8	1	200	38	41	-3	105.1	
Garcinia	*mangostana*	8	1	100	56	52	4	93.2	
Garcinia	*mangostana*	8	1	50	78	47	31	47.5	
Garcinia	*mangostana*	8	1	25	89	46	43	27.1	
Garcinia	*mangostana*	8	1	12.5	101	55	46	22.0	
Garcinia	*mangostana*	8	1	0.6	106	53	53	10.2	
Garcinia	*mangostana*	8	1	0.3	96	52	44	25.4	
Garcinia	*mangostana*	8	1	0.1	103	56	47	20.3	
Garcinia	*mangostana*	8	1	0	101	42	59	0.0	
Garcinia	*mangostana*	8	1	500	42	43	-1	101.7	44.12
Garcinia	*mangostana*	8	1	200	45	45	0	100.0	
Garcinia	*mangostana*	8	1	100	56	51	5	91.5	
Garcinia	*mangostana*	8	1	50	66	51	15	74.6	
Garcinia	*mangostana*	8	1	25	74	44	30	49.2	
Garcinia	*mangostana*	8	1	12.5	105	48	57	3.4	
Garcinia	*mangostana*	8	1	0.6	104	48	56	5.1	
Garcinia	*mangostana*	8	1	0.3	99	49	50	15.3	
Garcinia	*mangostana*	8	1	0.1	106	51	55	6.8	
Garcinia	*mangostana*	8	1	0	110	51	59	0.0	

S7 Table. Data from Titrations of Top Botanical Candidates for Inhibition of Fructose-induced Elevation in TG Levels.

Botanical Extract					TG (mg/dL)	TG (mg/dL)	ΔTG	TG Inhibition	*TG IC_{50}
Genus	Species	SampleID	Lot #	Conc (μg/mL)	(Fructose Only Controls)	(No Fructose Controls)	(mg/dL)	(%)	(μg/mL)
Angelica	*archangelica*	1	2	500	39	43	-4	105.8	75.67
Angelica	*archangelica*	1	2	200	44	39	5	92.8	
Angelica	*archangelica*	1	2	100	59	45	14	79.7	
Angelica	*archangelica*	1	2	50	62	41	21	69.6	
Angelica	*archangelica*	1	2	25	89	42	47	31.9	
Angelica	*archangelica*	1	2	12.5	95	41	54	21.7	
Angelica	*archangelica*	1	2	0.6	94	40	54	21.7	
Angelica	*archangelica*	1	2	0.3	89	38	51	26.1	
Angelica	*archangelica*	1	2	0.1	99	38	61	11.6	
Angelica	*archangelica*	1	2	0	105	36	69	0.0	
Angelica	*archangelica*	1	2	500	41	43	-2	103.6	41.96
Angelica	*archangelica*	1	2	200	46	48	-2	103.6	
Angelica	*archangelica*	1	2	100	49	44	5	91.1	
Angelica	*archangelica*	1	2	50	66	46	20	64.3	
Angelica	*archangelica*	1	2	25	79	47	32	42.9	
Angelica	*archangelica*	1	2	12.5	99	51	48	14.3	
Angelica	*archangelica*	1	2	0.6	98	53	45	19.6	
Angelica	*archangelica*	1	2	0.3	94	39	55	1.8	
Angelica	*archangelica*	1	2	0.1	106	38	68	-21.4	
Angelica	*archangelica*	1	2	0	110	54	56	0.0	
Scutellaria	*baicalensis*	2	2	500	46	42	4	93.5	19.49
Scutellaria	*baicalensis*	2	2	200	45	45	0	100.0	
Scutellaria	*baicalensis*	2	2	100	49	44	5	91.9	
Scutellaria	*baicalensis*	2	2	50	59	40	19	69.4	
Scutellaria	*baicalensis*	2	2	25	58	45	13	79.0	
Scutellaria	*baicalensis*	2	2	12.5	83	42	41	33.9	
Scutellaria	*baicalensis*	2	2	0.6	91	41	50	19.4	
Scutellaria	*baicalensis*	2	2	0.3	95	43	52	16.1	
Scutellaria	*baicalensis*	2	2	0.1	101	43	58	6.5	
Scutellaria	*baicalensis*	2	2	0	101	39	62	0.0	
Scutellaria	*baicalensis*	2	2	500	42	41	1	98.4	13.10
Scutellaria	*baicalensis*	2	2	200	51	39	12	80.3	
Scutellaria	*baicalensis*	2	2	100	51	46	5	91.8	
Scutellaria	*baicalensis*	2	2	50	46	42	4	93.4	
Scutellaria	*baicalensis*	2	2	25	59	41	18	70.5	
Scutellaria	*baicalensis*	2	2	12.5	79	45	34	44.3	
Scutellaria	*baicalensis*	2	2	0.6	95	51	44	27.9	
Scutellaria	*baicalensis*	2	2	0.3	101	49	52	14.8	
Scutellaria	*baicalensis*	2	2	0.1	100	42	58	4.9	
Scutellaria	*baicalensis*	2	2	0	99	38	61	0.0	
Petroselinum	*crispum*	3	2	500	51	41	10	82.1	240.80
Petroselinum	*crispum*	3	2	200	58	45	13	76.8	
Petroselinum	*crispum*	3	2	100	66	38	28	50.0	
Petroselinum	*crispum*	3	2	50	98	38	60	-7.1	
Petroselinum	*crispum*	3	2	25	92	35	57	-1.8	
Petroselinum	*crispum*	3	2	12.5	102	46	56	0.0	
Petroselinum	*crispum*	3	2	0.6	101	42	59	-5.4	
Petroselinum	*crispum*	3	2	0.3	97	51	46	17.9	
Petroselinum	*crispum*	3	2	0.1	106	50	56	0.0	
Petroselinum	*crispum*	3	2	0	105	49	56	0.0	
Petroselinum	*crispum*	3	2	500	66	40	26	60.6	291.30
Petroselinum	*crispum*	3	2	200	76	39	37	43.9	
Petroselinum	*crispum*	3	2	100	86	36	50	24.2	
Petroselinum	*crispum*	3	2	50	97	42	55	16.7	
Petroselinum	*crispum*	3	2	25	97	44	53	19.7	
Petroselinum	*crispum*	3	2	12.5	102	48	54	18.2	
Petroselinum	*crispum*	3	2	0.6	106	48	58	12.1	
Petroselinum	*crispum*	3	2	0.3	110	46	64	3.0	
Petroselinum	*crispum*	3	2	0.1	116	49	67	-1.5	
Petroselinum	*crispum*	3	2	0	112	46	66	0.0	
Garcinia	*mangostana*	4	2	500	44	38	6	89.5	22.53
Garcinia	*mangostana*	4	2	200	44	46	-2	103.5	
Garcinia	*mangostana*	4	2	100	51	42	9	84.2	
Garcinia	*mangostana*	4	2	50	59	42	17	70.2	
Garcinia	*mangostana*	4	2	25	65	41	24	57.9	
Garcinia	*mangostana*	4	2	12.5	83	44	39	31.6	
Garcinia	*mangostana*	4	2	0.6	93	44	49	14.0	
Garcinia	*mangostana*	4	2	0.3	102	47	55	3.5	
Garcinia	*mangostana*	4	2	0.1	104	43	61	-7.0	
Garcinia	*mangostana*	4	2	0	100	43	57	0.0	
Garcinia	*mangostana*	4	2	500	46	41	5	90.4	12.42
Garcinia	*mangostana*	4	2	200	39	44	-5	109.6	
Garcinia	*mangostana*	4	2	100	47	51	-4	107.7	
Garcinia	*mangostana*	4	2	50	49	49	0	100.0	
Garcinia	*mangostana*	4	2	25	56	46	10	80.8	
Garcinia	*mangostana*	4	2	12.5	79	52	27	48.1	
Garcinia	*mangostana*	4	2	0.6	78	48	30	42.3	
Garcinia	*mangostana*	4	2	0.3	92	48	44	15.4	
Garcinia	*mangostana*	4	2	0.1	100	49	51	1.9	
Garcinia	*mangostana*	4	2	0	101	49	52	0.0	

S7 Table. Data from Titrations of Top Botanical Candidates for Inhibition of Fructose-induced Elevation in TG Levels.

Botanical Extract					TG (mg/dL)	TG (mg/dL)	ΔTG	TG Inhibition	*TG IC_{50}
Genus	Species	SampleID	Lot #	Conc (μg/mL)	(Fructose Only Controls)	(No Fructose Controls)	(mg/dL)	(%)	(μg/mL)

IC_{50}: half maximal inhibitory concentration. OD: optical density. TG: triglycerides.

*TG IC_{50}s were calculated using nonlinear regression (three parameters) in GraphPad Prism 5.03. To generate a best fit, an upper concentration (10,000 μg/mL at 100% inhibition) and a lower concentration (0.001 μg/mL at 0% inhibition) were added.
