# Supplementary material for: Bioactivity-Guided Identification of Botanical Inhibitors of Ketohexokinase
Source: PLoS One. 2016 Jun 20;11(6):e0157458. doi: 10.1371/journal.pone.0157458 (PMC4913896; doi:10.1371/journal.pone.0157458)
Supplement: S8 Table — IC50: half maximal inhibitory concentration. OD: optical density. UA: uric acid. *UA IC50s were calculated using nonlinear regression (three parameters) in GraphPad Prism 5.03. To generate a best fit, an upper concentration (10,000 μg/mL at 100% inhibition) and a lower concentration (0.001 μg/mL at 0% inhibition) were added. (PDF) [file pone.0157458.s008.pdf]

S8 Table. Data from Titrations of Top Botanical Candidates for Inhibition of Fructose-induced Elevation in UA Levels.

Botanical Extract					UA (mg/dL)	UA (mg/dL)	ΔUA	UA Inhibition	*UA IC_{50}
Genus	Species	SampleID	Lot #	Conc (μg/mL)	(Fructose Only Controls)	(No Fructose Controls)	(mg/dL)	(%)	(μg/mL)
Angelica	*archangelica*	1	2	500	0.95	1.06	-0.11	121.2	1.56
Angelica	*archangelica*	1	2	200	1.13	0.95	0.18	65.4	
Angelica	*archangelica*	1	2	100	1.23	0.98	0.25	51.9	
Angelica	*archangelica*	1	2	50	1.25	1.25	0	100.0	
Angelica	*archangelica*	1	2	25	1.38	1.32	0.06	88.5	
Angelica	*archangelica*	1	2	12.5	1.48	1.21	0.27	48.1	
Angelica	*archangelica*	1	2	0.6	1.75	1.21	0.54	-3.8	
Angelica	*archangelica*	1	2	0.3	1.85	1.2	0.65	-25.0	
Angelica	*archangelica*	1	2	0.1	1.82	1.06	0.76	-46.2	
Angelica	*archangelica*	1	2	0	2.02	1.5	0.52	0.0	
Angelica	*archangelica*	1	2	500	1.06	1.06	0	100.0	33.35
Angelica	*archangelica*	1	2	200	1.15	0.95	0.2	80.0	
Angelica	*archangelica*	1	2	100	1.28	0.98	0.3	70.0	
Angelica	*archangelica*	1	2	50	1.45	1.25	0.2	80.0	
Angelica	*archangelica*	1	2	25	1.86	1.32	0.54	46.0	
Angelica	*archangelica*	1	2	12.5	2.06	1.21	0.85	15.0	
Angelica	*archangelica*	1	2	0.6	2.16	1.21	0.95	5.0	
Angelica	*archangelica*	1	2	0.3	2.08	1.2	0.88	12.0	
Angelica	*archangelica*	1	2	0.1	2.1	1.06	1.04	-4.0	
Angelica	*archangelica*	1	2	0	2.15	1.15	1	0.0	
Scutellaria	*baicalensis*	2	2	500	1.11	1.21	-0.1	109.3	51.01
Scutellaria	*baicalensis*	2	2	200	1.31	1.06	0.25	76.6	
Scutellaria	*baicalensis*	2	2	100	1.23	1.12	0.11	89.7	
Scutellaria	*baicalensis*	2	2	50	1.46	1.11	0.35	67.3	
Scutellaria	*baicalensis*	2	2	25	1.67	1.14	0.53	50.5	
Scutellaria	*baicalensis*	2	2	12.5	1.66	1.15	0.51	52.3	
Scutellaria	*baicalensis*	2	2	0.6	1.86	1.01	0.85	20.6	
Scutellaria	*baicalensis*	2	2	0.3	1.86	1.23	0.63	41.1	
Scutellaria	*baicalensis*	2	2	0.1	2.11	1.28	0.83	22.4	
Scutellaria	*baicalensis*	2	2	0	2.13	1.06	1.07	0.0	
Scutellaria	*baicalensis*	2	2	500	1.1	1.19	-0.09	108.1	0.22
Scutellaria	*baicalensis*	2	2	200	1.08	1.16	-0.08	107.2	
Scutellaria	*baicalensis*	2	2	100	1.07	1.09	-0.02	101.8	
Scutellaria	*baicalensis*	2	2	50	1.19	1.11	0.08	92.8	
Scutellaria	*baicalensis*	2	2	25	1.36	1.19	0.17	84.7	
Scutellaria	*baicalensis*	2	2	12.5	1.46	1.23	0.23	79.3	
Scutellaria	*baicalensis*	2	2	0.6	1.51	1.29	0.22	80.2	
Scutellaria	*baicalensis*	2	2	0.3	1.91	1.26	0.65	41.4	
Scutellaria	*baicalensis*	2	2	0.1	2.06	1.23	0.83	25.2	
Scutellaria	*baicalensis*	2	2	0	2.23	1.12	1.11	0.0	
Petroselinum	*crispum*	3	2	500	1.01	1.1	-0.09	112.0	31.28
Petroselinum	*crispum*	3	2	200	1.06	1.02	0.04	94.7	
Petroselinum	*crispum*	3	2	100	1.15	0.95	0.2	73.3	
Petroselinum	*crispum*	3	2	50	0.99	0.85	0.14	81.3	
Petroselinum	*crispum*	3	2	25	1.02	0.99	0.03	96.0	
Petroselinum	*crispum*	3	2	12.5	1.29	1.01	0.28	62.7	
Petroselinum	*crispum*	3	2	0.6	1.35	1.02	0.33	56.0	
Petroselinum	*crispum*	3	2	0.3	1.36	1.12	0.24	68.0	
Petroselinum	*crispum*	3	2	0.1	1.56	1.11	0.45	40.0	
Petroselinum	*crispum*	3	2	0	1.86	1.11	0.75	0.0	
Petroselinum	*crispum*	3	2	500	1.16	1.06	0.1	90.9	26.37
Petroselinum	*crispum*	3	2	200	1.19	1.09	0.1	90.9	
Petroselinum	*crispum*	3	2	100	1.29	1.16	0.13	88.2	
Petroselinum	*crispum*	3	2	50	1.49	1.12	0.37	66.4	
Petroselinum	*crispum*	3	2	25	1.56	1.11	0.45	59.1	
Petroselinum	*crispum*	3	2	12.5	1.79	1.11	0.68	38.2	
Petroselinum	*crispum*	3	2	0.6	1.85	1.16	0.69	37.3	
Petroselinum	*crispum*	3	2	0.3	2.21	1.23	0.98	10.9	
Petroselinum	*crispum*	3	2	0.1	2.25	1.22	1.03	6.4	
Petroselinum	*crispum*	3	2	0	2.29	1.19	1.1	0.0	
Garcinia	*mangostana*	4	2	500	1.03	0.85	0.18	70.0	0.65
Garcinia	*mangostana*	4	2	200	1.13	1.13	0	100.0	
Garcinia	*mangostana*	4	2	100	1.23	1.15	0.08	86.7	
Garcinia	*mangostana*	4	2	50	1.25	1.21	0.04	93.3	
Garcinia	*mangostana*	4	2	25	1.19	1.28	-0.09	115.0	
Garcinia	*mangostana*	4	2	12.5	1.35	1.21	0.14	76.7	
Garcinia	*mangostana*	4	2	0.6	1.49	1.16	0.33	45.0	
Garcinia	*mangostana*	4	2	0.3	1.48	1.16	0.32	46.7	
Garcinia	*mangostana*	4	2	0.1	1.59	1.12	0.47	21.7	
Garcinia	*mangostana*	4	2	0	1.76	1.16	0.6	0.0	
Garcinia	*mangostana*	4	2	500	0.98	1.06	-0.08	108.4	6.85
Garcinia	*mangostana*	4	2	200	0.97	0.99	-0.02	102.1	
Garcinia	*mangostana*	4	2	100	1.06	1.16	-0.1	110.5	
Garcinia	*mangostana*	4	2	50	1.02	1.1	-0.08	108.4	
Garcinia	*mangostana*	4	2	25	1.15	1.09	0.06	93.7	
Garcinia	*mangostana*	4	2	12.5	1.28	1.15	0.13	86.3	
Garcinia	*mangostana*	4	2	0.6	1.49	1.13	0.36	62.1	
Garcinia	*mangostana*	4	2	0.3	1.52	1.2	0.32	66.3	
Garcinia	*mangostana*	4	2	0.1	1.66	1.09	0.57	40.0	
Garcinia	*mangostana*	4	2	0	2.09	1.14	0.95	0.0	

S8 Table. Data from Titrations of Top Botanical Candidates for Inhibition of Fructose-induced Elevation in UA Levels.

Botanical Extract					UA (mg/dL)	UA (mg/dL)	ΔUA	UA Inhibition	*UA IC_{50}
Genus	Species	SampleID	Lot #	Conc (μg/mL)	(Fructose Only Controls)	(No Fructose Controls)	(mg/dL)	(%)	(μg/mL)

IC_{50}: half maximal inhibitory concentration. OD: optical density. UA: uric acid.

*UA IC_{50}s were calculated using nonlinear regression (three parameters) in GraphPad Prism 5.03. To generate a best fit, an upper concentration (10,000 μg/mL at 100% inhibition) and a lower concentration (0.001 μg/mL at 0% inhibition) were added.
